# Extended-release ketamine tablets for treatment-resistant depression: a randomized placebo-controlled phase 2 trial

**DOI:** 10.1038/s41591-024-03063-x

**Published:** 2024-06-24

**Authors:** Paul Glue, Colleen Loo, Johnson Fam, Hsien-Yuan Lane, Allan H. Young, Peter Surman, BEDROC study investigators, Nick Glozier, Paul Fitzgerald, Dennis Liu, Shanthi Sharma, Jennifer Grunfeld, David Barton, Malcolm Hopwood, Wayne Miles, Mike Williams, Simon Carson, Johnson Fam, Phern-Chern Tor, Hsien-Yuan Lane, Chun-Hsin Chen, Yu-Jui Huang

**Affiliations:** 1 University of Otago, Dunedin, New Zealand; 2 Black Dog Institute & University of New South Wales, Sydney, New South Wales Australia; 3 George Institute for Global Health, Sydney, New South Wales Australia; 4 National University of Singapore, Singapore, Singapore; 5 China Medical University, Taichung, Taiwan; 6 China Medical University Hospital, Taichung, Taiwan; 7 Institute of Psychiatry, Psychology, and Neuroscience, King’s College London, London, UK; 8 Douglas Pharmaceuticals, Auckland, New Zealand; 9 Brain and Mind Centre, University of Sydney, Sydney, New South Wales Australia; 10 Australian National University College of Health and Medicine, Canberra, Australian Capital Territory Australia; 11 Lyell McEwin Hospital, Adelaide, South Australia Australia; 12 Gold Coast University Hospital, Southport, Queensland Australia; 13 Peninsula Therapeutic & Research Group, Melbourne, Victoria Australia; 14 Neurocentrix, Melbourne, Victoria Australia; 15 Ramsay Clinic Albert Road, University of Melbourne, Melbourne, Victoria Australia; 16 North Shore Hospital, Auckland, New Zealand; 17 Lakeland Clinical Trials & Anteris Clinical Research, Rotorua, New Zealand; 18 Southern Clinical Trials, Christchurch, New Zealand; 19 National University Hospital Singapore, Singapore, Singapore; 20 Institute of Mental Health, Singapore, Singapore; 21 China Medical University Hospital, Taichung City, Taiwan; 22 Taipei Municipal Wanfang Hospital, Taipei City, Taiwan; 23 Taipei Medical University Hospital, Taipei City, Taiwan

**Keywords:** Drug development, Drug delivery

## Abstract

Ketamine has rapid-onset antidepressant activity in patients with treatment-resistant major depression (TRD). The safety and tolerability of racemic ketamine may be improved if given orally, as an extended-release tablet (R-107), compared with other routes of administration. In this phase 2 multicenter clinical trial, male and female adult patients with TRD and Montgomery–Asberg Depression Rating Scale (MADRS) scores ≥20 received open-label R-107 tablets 120 mg per day for 5 days and were assessed on day 8 (enrichment phase). On day 8, responders (MADRS scores ≤12 and reduction ≥50%) were randomized on a 1:1:1:1:1 basis to receive double-blind R-107 doses of 30, 60, 120 or 180 mg, or placebo, twice weekly for a further 12 weeks. Nonresponders on day 8 exited the study. The primary endpoint was least square mean change in MADRS for each active treatment compared with placebo at 13 weeks, starting with the 180 mg dose, using a fixed sequence step-down closed test procedure. Between May 2019 and August 2021, 329 individuals were screened for eligibility, 231 entered the open-label enrichment phase (days 1–8) and 168 responders were randomized to double-blind treatment. The primary objective was met; the least square mean difference of MADRS score for the 180 mg tablet group and placebo was −6.1 (95% confidence interval 1.0 to 11.16, *P* = 0.019) at 13 weeks. Relapse rates during double-blind treatment showed a dose response from 70.6% for placebo to 42.9% for 180 mg. Tolerability was excellent, with no changes in blood pressure, minimal reports of sedation and minimal dissociation. The most common adverse events were headache, dizziness and anxiety. During the randomized phase of the study, most patient dosing occurred at home. R-107 tablets were effective, safe and well tolerated in a patient population with TRD, enriched for initial response to R-107 tablets. ClinicalTrials.gov registration: ACTRN12618001042235.

## Abstract (web summary)

A phase 2 dose-finding study of an extended-release tablet formulation of ketamine in patients with treatment-resistant depression shows that this formulation overcomes many of the limitations associated with the use of intravenous or intranasal ketamine formulations.

## Main

Over the past two decades, there has been a growing evidence base demonstrating the rapid-onset antidepressant properties of ketamine in patients with treatment-resistant depression (TRD). The majority of published research has been with off-label use of racemic ketamine^1^, most commonly administered intravenously, with a more recent regulatory approval of intranasal esketamine for TRD^2^. Only 2/72 treatment arms in published randomized controlled trials for TRD involved oral dosing^1^. Ketamine and esketamine can be effectively administered via multiple routes, with higher doses associated with greater improvement in depression compared with lower doses^1^. Published dose ranges and bioavailability vary by formulation and route of administration^3^.

The pharmacology of ketamine relating to its antidepressant activity has been linked to several of its metabolites, including norketamine and the hydronorketamines^4,5^. After oral dosing, pharmacokinetic exposure to norketamine and the hydronorketamines is considerably more prolonged than exposure to ketamine^6^. Furthermore, ketamine is still active as an antidepressant even when dosed by routes where bioavailability of parent ketamine is low^7^. A synthesis of these observations suggests that ketamine may be acting as a prodrug, where its antidepressant activity is substantially due to its metabolites. A meta-analysis of ketamine formulations identified that formulations that maximize first-pass metabolism of ketamine and delay time to maximum concentrations were better tolerated (less dissociation) and safer (less blood pressure change) than formulations that lack those characteristics^8^. We hypothesized that an extended-release tablet formulation of ketamine could be an effective and well-tolerated treatment option for patients with TRD. Details of the formulation and its pharmacokinetic profile have been published^9,10^. Due to its pronged absorption phase, it undergoes extensive first-pass metabolism, and its absolute bioavailability is <20% (ref. ^8^). In this Article, we report on a multicenter phase 2 study of the extended-release ketamine tablets (R-107) in patients with TRD.

**Fig. 1:**
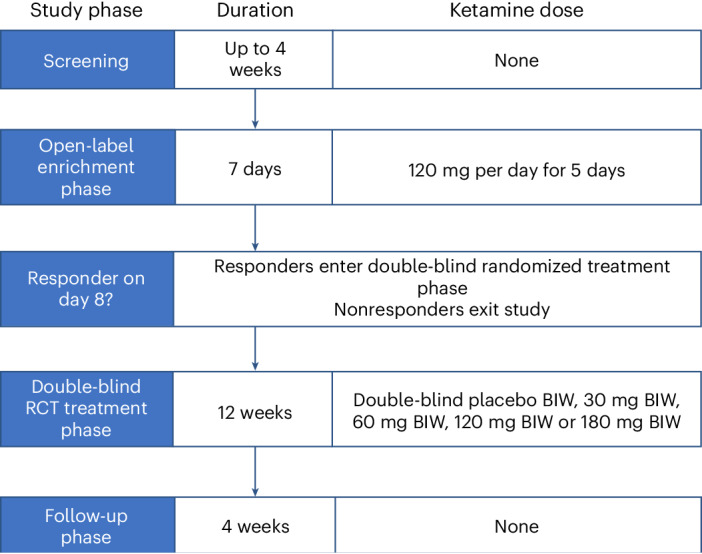
BEDROC study design. BIW, twice-weekly dosing; RCT, randomized controlled trial.

The study design for this proof-of-concept trial is shown in Fig. 1. We chose this design owing to observations that acute antidepressant clinical trials in non-TRD depression have high failure rates (inability to separate clinical response between active and placebo arms), as high as 50% (refs. ^11,12^). Failure rates can be reduced by using an enrichment design, in which nonresponders to acute treatment are excluded, followed by a subsequent relapse-prevention phase in treatment responders^13^; study failure rates using this design are as low as 25%. Temple has described this strategy as a type of predictive enrichment, producing a more treatment-responsive sample and, thus, increasing effect size^14^. A similar design was used in Daly’s esketamine randomized withdrawal study^15^. We included a dose-finding component in our double-blind relapse prevention phase as it was unclear what the effective oral dose range might be.

## Results

**Fig. 2:**
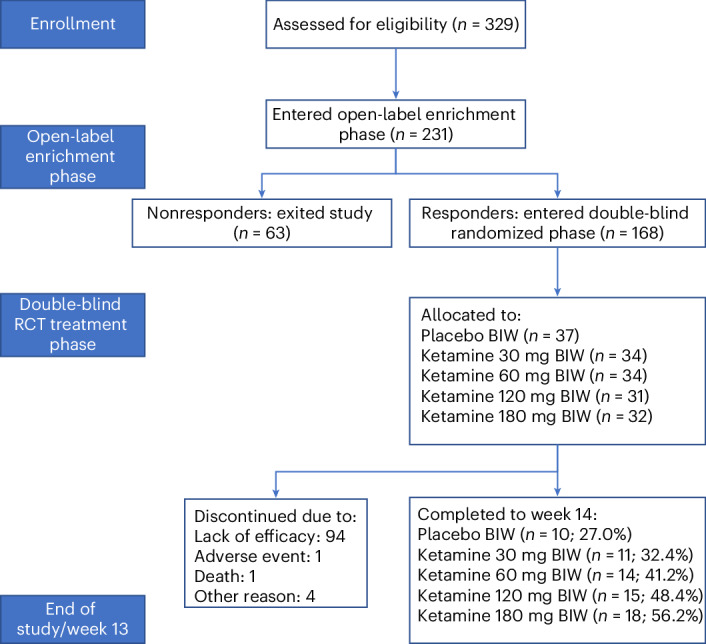
BEDROC patient disposition. CONSORT diagram indicating patient numbers and disposition throughout the trial. BIW, twice-weekly dosing; RCT, randomized controlled trial.

Between May 2019 and August 2021, 329 individuals were screened for eligibility, 231 entered the open-label enrichment phase (days 1–5). At day 8 assessment, 132/231 (57.1%) of participants were in remission, and 168/231 (72.7%) were responders. After exclusion of nonresponders, the 168 responders were randomized to double-blind treatment (see CONSORT diagram in Fig. 2). Participant demographic details are provided in Table 1. Mean pretreatment Montgomery–Asberg Depression Rating Scale (MADRS) scores were approximately 30, and mean number of failed antidepressant trials was approximately 4.8 (Table 1). By the end of the study (day 92), 100 participants had discontinued, of whom 94 were for lack of efficacy as defined by an MADRS total score of ≥22 (placebo, 26; 30 mg, 22; 60 mg, 19; 120 mg, 16; 180 mg, 11) (Fig. 2). The proportion of participants who completed the study ranged from 29.7% in the placebo arm through to 56.2% for the 180 mg dose arm, with higher proportions of completers associated with higher R-107 doses. Treatment compliance was high, with almost all participants (96.4%) reported to have compliance of 80% or more (at home and in clinic).

**Table 1:** Baseline characteristics of study participants enrolled in the randomized double-blind phase

		Placebo	R-107 30 mg	R-107 60 mg	R-107 120 mg	R-107 180 mg	*P* value for difference between treatment groups
*n*		37	34	34	31	32	N/A
Age, years	Mean (s.d.)	43.7 (15.43)	44.6 (12.89)	42.5 (15.80)	47.2 (13.80)	46.8 (11.90)	0.6063
	Median (IQR)	42 (31–57)	45.5 (34.75–51.5)	40.5 (28.5–53.75)	48 (33.5–60)	46 (36–56.25)	N/A
Sex (M/F)		22/15	18/16	18/16	13/18	21/11	0.4096
Number of prior depressive episodes	Mean (s.d.)	3.9 (7.42)	3.2 (4.33)	3.1 (3.21)	4.4 (4.67)	1.8 (1.59)	0.3526
	Median (IQR)	1 (1–3)	1 (1–3)	1 (1–4.5)	2 (1–4)	1 (1–2)	N/A
Treatment resistance—mean number of past failed ADs in this episode	Mean (SD)	4.8 (2.84)	5.0 (2.74)	4.6 (2.70)	4.8 (3.39)	4.7 (2.74)	0.9871
	Median (IQR)	4 (3–5.75)	5 (2.5–6)	4 (2.25–6)	3.5 (2–5.75)	4 (3–6)	N/A
Failed ECT pre-study (%)		2 (5.4%)	4 (11.8%)	1 (2.9%)	5 (16.1%)	2 (6.2%)	0.3372
Number taking ADs pre-study entry (%)		34 (91.9%)	32 (94.1%)	31 (91.2%)	31 (100.0%)	28 (87.5%)	0.361
Day 1 MADRS score	Mean (SD)	30.2 (4.48)	29.9 (4.14)	29.3 (5.80)	31.4 (5.19)	29.9 (4.61)	0.5111
	Median (IQR)	30 (27–34)	29.5 (27.25–31.75)	29 (25.25–31.75)	31 (28–34)	29 (27.5–34)	N/A

*P* values are calculated using Fisher’s exact test for the number of prior depressive episodes, failed ECT pre-study, and number taking ADs pre-study entry; and analysis of variance (ANOVA) for age and day 1 MADRS score. The ANOVA was based on regressing the variable (treatment resistance, MADRS and so on) on the dose group.

AD, antidepressant; ECT, electroconvulsive therapy; IQR, interquartile range; N/A, not available.

### Primary outcome

Estimated marginal mean reductions at days 36, 64 and 92 are presented in Extended Data Table 1. Numerically, greater mean reductions in the MADRS total score from baseline to day 92 were observed in all treatment groups compared with placebo (R-107 30 mg: 1.9 (95% confidence interval (CI) −3.08 to 6.92), *P* = 0.450; 60 mg: 0.7 (95% CI −4.32 to 5.70), *P* = 0.785; 120 mg: 4.5 (95% CI −0.60 to 9.69), *P* = 0.083). The largest reduction was in the 180 mg treatment group: 6.1 (95% CI 1.00 to 11.16; *P* = 0.019), and this result was statistically significant. Mean (standard deviation, s.d.) reductions in MADRS scores by treatment group are presented in Table 2. The 120 mg and 180 mg dose groups had lower mean reductions (<10 points) compared with lower-dose groups. Compared with placebo, numerically greater reductions in day 92 MADRS scores (95% CI) were observed for females (−10.1 (−18.7 to −1.5)) compared with males (−4.2 (−10.8 to 2.4)), patients younger than 65 years (−6.9 (−12.3 to −1.6)) compared with patients 65 years and older (0.1 (−23.4 to 23.7)), those taking antidepressants (−6.5 (−12.5 to −0.6)) compared with those not taking antidepressants (−2.5 (−12.6 to 7.7)), and those with greater than median body weight (−7.1 (−14.0 to −0.1)) compared with those below median body weight (−5.3 (−13.1 to 2.5)).

**Table 2:** Estimated marginal mean (95% CI) reduction in MADRS scores from baseline on days 8, 36, 64 and 92, by treatment group, in the double-blind treatment phase, with last observation carried forward approach

Day	Statistics	Placebo (*N* = 37)	R-107 30 mg (*N* = 34)	R-107 60 mg (*N* = 34)	R-107 120 mg (*N* = 31)	R-107 180 mg (*N* = 32)
Day 8	Mean (95% CI)	22.6 (21.63 to 23.62)	22.1 (21.09 to 23.17)	23.0 (21.99 to 24.08)	22.1 (20.97 to 23.16)	22.3 (21.25 to 23.39)
Day 15	Mean (95% CI)	17.8 (15.04 to 20.49)	17.4 (14.55 to 20.23)	16.5 (13.62 to 19.31)	16.1 (13.15 to 19.13)	20.5 (17.53 to 23.39)
Day 22	Mean (95% CI)	14.6 (11.50 to 17.65)	15.0 (11.77 to 18.18)	12.6 (9.39 to 15.81)	14.3 (10.88 to 17.63)	18.9 (15.64 to 22.25)
Day 29	Mean (95% CI)	14.8 (11.46 to 18.19)	13.0 (9.50 to 16.52)	11.5 (7.94 to 14.97)	13.5 (9.77 to 17.16)	18.0 (14.43 to 21.66)
Day 36	Mean (95% CI)	12.8 (9.28 to 16.28)	12.9 (9.26 to 16.56)	10.8 (7.17 to 14.49)	13.2 (9.31 to 17.00)	15.9 (12.15 to 19.67)
Day 64	Mean (95% CI)	8.4 (5.01 to 11.79)	10.5 (6.99 to 14.07)	8.9 (5.40 to 12.49)	12.4 (8.66 to 16.12)	15.4 (11.73 to 19.03)
Day 92	Mean (95% CI)	8.0 (4.49 to 11.41)	9.9 (6.26 to 13.48)	8.6 (5.02 to 12.26)	12.5 (8.69 to 16.30)	14.0 (10.31 to 17.75)

### Secondary efficacy outcomes

During the open-label enrichment phase (days 1–8), there was mean reduction in MADRS total score of 18.5 points (95% CI 17.37 to 19.69) at day 8. A total of 132 participants (57.1%) of the 231 enrolled in the enrichment phase achieved remission with as MADRS total score ≤10 at day 8. A total of 168 participants (72.7%) of the 231 patients enrolled in the enrichment phase achieved a response to treatment, defined as ≥50% reduction from baseline in MADRS score at day 8.

Rates of remission and response at week 13 were numerically greater for the active treatment arms compared with placebo; however, these were not statistically significant (remission) or were significant for only the 120 mg dose group for treatment response (48% versus 24.3%, *P* = 0.046; Extended Data Tables 2 and 3). Compared with baseline, Clinical Global Improvement-Severity (CGI-S) scores improved in participants randomized to ketamine; however, this was not statistically significant compared with placebo. With the exception of the 60 mg dose group, the 120 mg and 180 mg ketamine dose groups had higher probability of improvement in depression severity from the subject’s perspective, using the Patient Global Impression-Improvement (PGI-I) scale, compared with the placebo group (OR (95% CI) 30 mg: 0.52 (0.09 to 2.78); 60 mg: 1.62 (0.36 to 7.42); 120 mg: 0.28 (0.06 to 1.25); 180 mg: 0.82 (0.19 to 3.51) (where ‘OR’ is ‘odds ratio’ and ORs <1 signify higher probabilities for the active treatment group for lower categories compared with the placebo group; Extended Data Table 4).

**Fig. 3:**
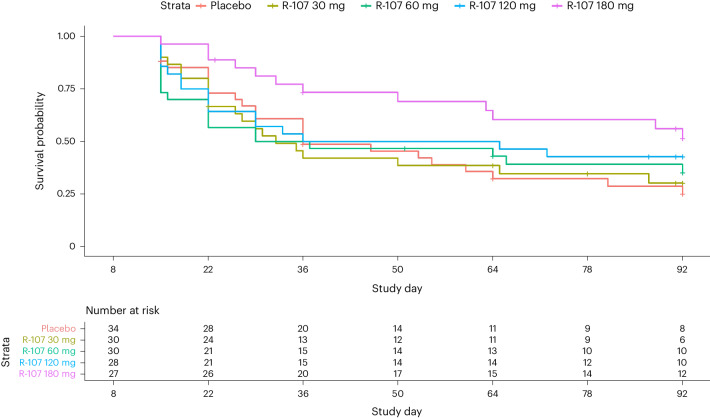
Kaplan–Meier analysis of the percentage of patients remaining in the trial in the enrichment phase of BEDROC stratified by dose group. The number of patients at risk at days 8, 22, 36, 50, 64, 78 and 92 is presented in the table below the figure. The difference in the restricted mean survival time for the 180 mg treatment group was statistically significantly greater compared with the placebo group (19.0 (95% CI 4.9 to 33.1)).

Temporal trends in relapse and numbers of patients in each dose group are shown in the Kaplan–Meier plot in Fig. 3. The majority of relapses occurred within the first 4 weeks of double-blind treatment. The median relapse time after randomization increased with higher R-107 doses (placebo: 45 days; 30 mg: 28 days; 60 mg: 56 days; 120 mg: 64 days; and 180 mg: >85 days). The difference in the restricted mean survival time for the 180 mg treatment group was statistically significantly greater compared with the placebo group (19.0 (95% CI 4.9 to 33.1)).

### Safety outcomes

Adverse events were rated predose and postdose before leaving clinic, and on scheduled telephone calls. During the open-label enrichment phase, the most common adverse events included dizziness, headache, dissociation, feeling abnormal, fatigue and nausea. Twenty-six participants (11.6%) reported dissociation. Mean dissociation (Clinician-Administered Dissociative States Scale, CADSS) scores were <3 for all participants throughout this phase. Mean blood pressure changes after 5 days of open-label 120 mg daily dosing in the enrichment phase were systolic and diastolic blood pressure changes of −1.2 mmHg and −0.1 mmHg, respectively.

The most common side effects reported in the double-blind treatment phase are presented in Table 3. The majority of these were of mild intensity (131 subjects; 56.7%) or moderate intensity (42 subjects; 18.2%). Mean CADSS scores were <1 point at all visits during the double-blind phase of the study. Sedation of mild severity was reported by a total of five participants (30 mg, *n* = 4; 120 mg, *n* = 1). Mean CADSS scores were <1 point at all time points during this phase of the study. Mean ratings of cystitis symptoms using the BPIC-SS questionnaire remained less than 3 points throughout the study, out of a maximum of 38, with no differences between placebo and 180 mg dose groups.

**Table 3:** Treatment-emergent adverse events occurring in >10% of study participants in any dose arm enrolled in the randomized double-blind phase

	Placebo (*n* = 37)	R-107 30 mg (*n* = 34)	R-107 60 mg (*n* = 34)	R-107 120 mg (*n* = 31)	R-107 180 mg (*n* = 32)	*P* value for difference between dose groups
Headache	6 (16.2%)	10 (29.4%)	11 (32.4%)	6 (19.4%)	6 (18.8%)	0.4314
Dizziness	3 (8.1%)	4 (11.8%)	5 (14.7%)	5 (16.1%)	9 (28.1%)	0.2475
Anxiety	2 (5.4%)	3 (8.8%)	1 (2.9%)	6 (19.4%)	6 (18.8%)	0.0992
Depression	2 (5.4%)	4 (11.8%)	5 (14.7%)	2 (6.5%)	3 (9.4%)	0.7087
Dissociation	0 (0.0%)	1 (2.9%)	1 (2.9%)	2 (6.5%)	5 (15.6%)	0.0518
Nausea	3 (8.1%)	3 (8.8%)	2 (5.9%)	3 (9.7%)	5 (15.6%)	0.7683
Feeling abnormal	2 (5.4%)	5 (14.7%)	1 (2.9%)	2 (6.5%)	3 (9.4%)	0.4702
Fatigue	3 (8.1%)	1 (2.9%)	0 (0.0%)	4 (12.9%)	2 (6.2%)	0.1944
URTI	4 (10.8%)	3 (8.8%)	2 (5.9%)	2 (6.5%)	3 (9.4%)	0.9627

The *P* values were calculated using Fisher’s exact test for the differences between dose groups.

URTI, upper respiratory tract infection.

During double-blind treatment, there were ten severe adverse events in eight participants: severe headache (30 mg and 60 mg dose groups); severe depression (120 mg and 180 mg dose groups); completed suicide at day 42 in a 65-year-old male (180 mg dose group); noncardiac chest pain (60 mg dose group); nausea (30 mg dose group); intervertebral disc protrusion (120 mg dose group); and nephrolithiasis and ureterolithiasis (in one participant in the placebo group). Five subjects experienced serious adverse events (SAEs): three participants in the 180 mg group (wound dehiscence (*n* = 1), suicidal ideation (*n* = 1) and completed suicide (*n* = 1); one participant in the 60 mg group had noncardiac chest pain; and one participant in the placebo group had a urinary calculus. None of the SAEs was considered treatment related (the suicide was considered by the site principal investigator to be due to the disease under study), and all SAEs resolved except for the completed suicide.

There were no changes of note in safety laboratory tests, urinalyses, vital signs, body weights or electrocardiograms (ECGs). There were no changes of note in Brief Psychiatric Rating Scale (BPRS+) or Montreal Cognitive Assessment scores during the study.

## Discussion

In this study, 231 patients with TRD were treated with R-107 120 mg per day for 5 days, and 168 (72.7%) were included as an enriched responder population who were randomized to a range of double-blind R-107 doses or placebo for the next 12 weeks. In this double-blind phase, the 180 mg dose given twice weekly showed statistically significant and clinically meaningful improvement in depressive symptoms based on MADRS score compared with placebo, with a group-treatment difference of 6.1. Side effects commonly observed in clinical trials of injected or intranasal ketamine (for example, dissociation, sedation and increased blood pressure) were minimal, and overall tolerability was good. Most patient dosing during the double-blind phase occurred at home.

Acute placebo-controlled antidepressant clinical trials in non-TRD patients have high failure rates, up to 50% (refs. ^11,12^). Study failure rates in patients with TRD may be similarly high (47%), based on the proportion of industry-funded studies of ketamine or esketamine registered on clinicaltrials.gov between 2010 and 2022, where no results have been published. As discussed in the introduction, failure rates (inability to separate responses between active and placebo arms) can be reduced by using an enrichment design to remove treatment nonresponders, before a double-blind relapse-prevention phase^13^, and is consistent with a regulatory guidance on enrichment designs^16^. Failure rate across all studies using this design was 25% (ref. ^13^). We included a dose-finding component in the double-blind phase of the present study as it was not clear what the effective oral dose range might be. The R-107 dose used in the enrichment phase (120 mg daily for 5 days) was based on observations from case reports from patients with pain and TRD receiving continuous ketamine infusions for 5 days, who reported mood improvements occurring by 24–72 h (ref. ^17^). The tablet formulation’s sustained exposure to norketamine over 24 h after once-daily dosing provided a similar prolonged pharmacokinetic exposure^9^. Ketamine dosing was open-label during the enrichment phase; therefore, the high remission (57.1%) and response (72.7%) rates for participants during this phase have to be considered cautiously due to likely expectation effects^18^. During the double-blind treatment phase, clear dose responses were observed, for the proportion of patients relapsing and median time to relapse, and there were dose-related trends for reductions in the MADRS total score. Most relapses in the 0–120 mg dose groups occurred within 1 month of randomization (Fig. 3). Only the mean between-group treatment difference between the 180 mg and placebo groups (−6.1) was statistically significant, and this value exceeds the minimum clinically important difference threshold for antidepressants reported in the literature^19^.

The relapse rates between weeks 2 and 13 in patients randomized to the placebo and 180 mg dose groups (70.3% and 43.7% respectively) are both higher than those reported in a meta-analysis of relapse-prevention studies of antidepressants in non-TRD patients^13^ and in patients with TRD enrolled in an esketamine randomized withdrawal study^15^ (Extended Data Table 5). This could be due to the much shorter duration of open-label dosing in the present study (5 days) compared with 16 weeks in patients with TRD^15^, and a mean of 16.4 weeks in non-TRD patients with depression^13^. These longer dosing periods before randomized withdrawal could select for stable responders, which would reduce subsequent relapse rates.

Many of the secondary efficacy outcome variables also showed dose-related trends compared with placebo; however, these were not statistically significant, presumably because of small dose group sizes, which may have reduced statistical power.

Commonly reported adverse events during the open-label enrichment phase included dizziness, headache, dissociation, feeling abnormal, fatigue and nausea. The intensity of dissociation in the 26 participants (11.6%) who reported this adverse event was minor, as demonstrated by mean CADSS scores of 3 or less for all participants. The most common side effects reported in the double-blind relapse-prevention phase were headache, dizziness, anxiety, depressed mood and dissociation (Table 3), most of which were mild to moderate in intensity. Other notable differences from adverse events commonly reported after administration of ketamine or esketamine^20^ were the absence of cardiovascular side effects, especially relating to increased blood pressure, low rates of dissociation and also very low rates of sedation. Mean ratings of cystitis symptoms using the bladder pain/interstitial cystitis symptom score (BPIC-SS) questionnaire remained less than 3 points throughout the study, out of a maximum of 38, with no differences between placebo and 180 mg dose groups.

Another common concern about most currently available ketamine and esketamine formulations is the risk of diversion and abuse^21^. The extended-release ketamine tablets used in this study are exceptionally hard and difficult to shatter, due to annealing of polyethylene oxide during their manufacturing process^10^. This property may make this formulation less likely to be diverted for abuse, due to difficulty in manipulation of the tablets. We were not aware of any participants reporting craving for the tablets, and only one participant was removed from the study for lack of compliance. Most of the dosing of double-blind tablets after day 8 occurred at home rather than in clinic, and clinic visits were brief, which participants anecdotally reported to be convenient. These attributes potentially improve scalability of ketamine use in the community, due to reduced need for in-clinic monitoring, and would also reduce costs associated with clinic visits.

There are several important limitations to the trial. The study design (enrichment followed by relapse prevention) was intended to reduce risk of study failure^13^. Because this type of design eliminates nonresponders before randomization, this strategy is likely to overestimate population levels of treatment response to R-107, and future unenriched clinical trials are needed to address this issue. There are relatively few data for efficacy and tolerability after oral ketamine dosing compared with intravenous or intranasal dosing, and it is not possible to directly compare the present study’s findings with studies using nonoral routes of administration. This study included both participants established on antidepressants (*n* = 165) as well as those who were not on antidepressants (*n* = 60). Secondary analyses did not show differences in the acute (open-label phase) response to ketamine (the mean (95% CI) reduction in MADRS score for those taking an antidepressant was −19.2 versus −16.6 for those not taking an antidepressant (−2.6 (−5.19 to 0.02)). During the double-blind phase, there was a small but statistically significant greater reduction in MADRS scores at day 92 in patients taking antidepressants than those not respectively, −6.5 (−12.5 to −0.6) versus −2.5 (−12.6 to 7.7). Further larger studies are required to determine if these two populations respond differently to oral ketamine. Also, the protocol did not require patients to start a new antidepressant at the time of starting study medication, as this design would have complicated interpretation of this intervention.

In conclusion, extended-release R-107 tablets were effective, safe and well tolerated in an enriched patient population with TRD. Use of an extended-release oral dosage ketamine formulation may be advantageous compared with intranasal or intravenous dosing, in terms of reduced intensity of dissociation, lower risk of abuse, reduced frequency and intensity of sedative and cardiovascular side effects, and improved convenience for administration in the community.

## Methods

### Study design and oversight

This phase 2 multicenter clinical trial recruited participants from 20 psychiatric clinics in New Zealand, Australia, Singapore and Taiwan. The trial design included an initial 1-week open-label enrichment phase to exclude nonresponders, followed by a 12-week double-blind relapse prevention phase in participants who were treatment responders in the enrichment phase (Fig. 1). The trial was conducted in accordance with the ethical principles stated in the Declaration of Helsinki and Good Clinical Practice quality standards, and is reported in accordance with the CONSORT 2010 statement. The protocol, consent forms and associated documents were approved by local or national ethics committees. A copy of the protocol and statistical analysis plan are included with Supplementary Information. This study was prospectively registered (ACTRN12618001042235).

### Patients

We screened adult self-reported male and female patients (18–80 years) with DSM-5 major depressive disorder that was treatment resistant. This was defined as lack of clinically meaningful improvement despite the use of adequate doses of at least two antidepressant agents, derived from the group(s) of commonly used first-line treatment, prescribed for adequate duration. Adequate dose was defined as the minimum therapeutic dose as per the product label or maximum tolerated dose, and adequate duration was defined as a minimum duration of 6 weeks. Patients who provided written informed consent were eligible to enter screening. Patients’ depression scores, assessed using MADRS^22^, were 20 or higher during screening. Any concurrent antidepressant medication had to be at stable dosage ≥4 weeks before study entry, and during the study. Key exclusion criteria included having severe medical disorders, contraindications to the use of ketamine, clinically important findings on physical examination, safety laboratory tests or ECGs, serious risk for suicide, recent history of alcohol or drug abuse, or a history of bipolar disorder, schizophrenia or severe personality disorder. Detailed inclusion and exclusion criteria can be found in the study protocol (Supplementary Information).

### Trial procedures, randomization and blinding

Patients who met eligibility criteria and completed screening received open-label R-107 tablets 120 mg per day for 5 days (days 1–5; enrichment phase). On day 8, dosing responders (MADRS scores ≤12 and reduction ≥50% from baseline) were randomized on a 1:1:1:1:1 basis to receive double-blind R-107 doses of 30, 60, 120 or 180 mg, or placebo, twice weekly for 12 weeks; nonresponders exited the study. Each dose administered during the double-blind phase comprised three tablets that could contain 0, 30 or 60 mg R-107, to make up the allocated dose. Active and placebo tablets dispensed during the trial were identical in appearance. Randomization was by an automated integrated web response system. All patients, and all people involved in the conduct of the clinical trial were blinded to treatment allocation. During the double-blind relapse prevention phase, there were weekly clinic visits up to week 6, and clinic visits every 4 weeks up to 13 weeks. Medication compliance was monitored by participants completing a dosing diary that they brought to clinic visits for checking, plus return of investigational product containers. Participants also received scheduled phone checks from investigators at the study sites to enquire about compliance; during these calls, patients were asked if they had experienced any adverse events. Patients who relapsed during double-blind treatment (MADRS ≥22) were withdrawn from the study and could enter an open-label extension study.

### Dose justification

The open-label R-107 used in the day 1–5 enrichment phase had previously shown onset of antidepressant activity by day 2 of dosing in a pilot study of R-107 in patients with TRD^9^. This method of dosing was intended to provide continuous exposure to ketamine metabolites and to recreate exposures that would occur in a continuous ketamine infusion paradigm previously reported to have rapid onset antidepressant effects^17^. Doses used in the double-blind phase were intended to cover the range of oral doses reported to be active in a review of oral ketamine for depression^23^.

### Endpoints

The primary efficacy endpoint was the change in MADRS total score from baseline (day 1) to day 92 (week 13). This was evaluated with analysis of covariance, with dose as a factor and baseline MADRS as a covariate. Time to relapse was another efficacy measure. Other efficacy measures included the PGI-I and CGI-S scales^24^. Safety assessments included safety laboratory tests (hematology and biochemistry), ECGs, Montreal Cognitive Assessment^25^ and verbal fluency tests, Columbia Suicide Severity Rating Scale^26^, BPIC-SS^27^ and the four-item positive symptom subscale of BPRS+ (ref. ^28^). Tolerability assessments included reported adverse events and CADSS (dissociation)^29^ scores.

### Sample size and statistical analysis

The sample size calculation was based on the superiority of R-107 to placebo by a magnitude of six MADRS units, using an s.d. of change in MADRS of 7.5 units, a two-sided type 1 error of 0.05 and a power of 80%. A closed testing procedure was assumed whereby each dose group was compared with the placebo group in descending dose order, and 26 subjects per group were required. Allowing for a 13% dropout rate and an attrition rate of 25% during the enrichment open-label phase, approximately 200 subjects were required initially in order to have 150 subjects randomized to five treatment groups at the start of the double-blind randomized treatment phase.

The primary endpoint, change in MADRS total score from baseline (day 1) to day 92, was analyzed using analysis of covariance. The analysis was based on differences in MADRS total scores at day 92 from day 1 MADRS total score, with dose as factor and baseline MADRS total score as a covariate. Missing values for the day 92 MADRS total scores were imputed from the last available MADRS total score using a last observation carried forward approach, under the assumption that this was a conservative imputation (it was assumed that more relapses would occur in the placebo group, and that relapsed subjects would have deteriorated further had they remained in the study, so this imputation method was conservative in terms of the estimation of a treatment effect). This ensured the main analysis of the primary endpoint was not left unanalyzable due to high relapse rates in some groups. Time to relapse (defined as an MADRS score ≥22) was evaluated by Kaplan–Meier analysis, with restricted mean survival time calculated for each treatment group and differences compared with the placebo group.

### Reporting summary

Further information on research design is available in the Nature Portfolio Reporting Summary linked to this article.

## Online content

Any methods, additional references, Nature Portfolio reporting summaries, source data, extended data, supplementary information, acknowledgements, peer review information; details of author contributions and competing interests; and statements of data and code availability are available at 10.1038/s41591-024-03063-x.

### Supplementary information

Supplementary Information Clinical study protocol.

Reporting Summary

## Figures and Tables

**Extended Data Table 1:**
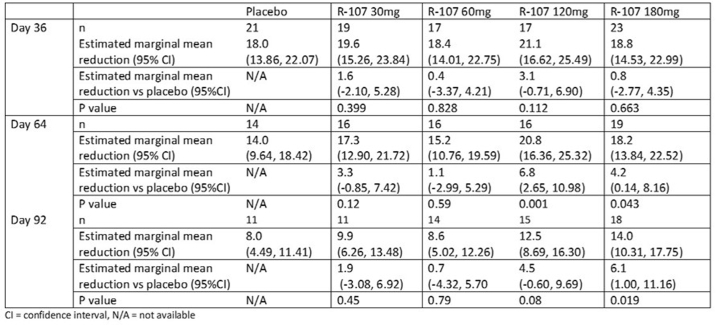
Estimated marginal mean (95% CI) reduction in MADRS scores on Days 36, 64 and 92, by treatment group, in the double-blind treatment phase with MMRM

**Extended Data Table 2:**
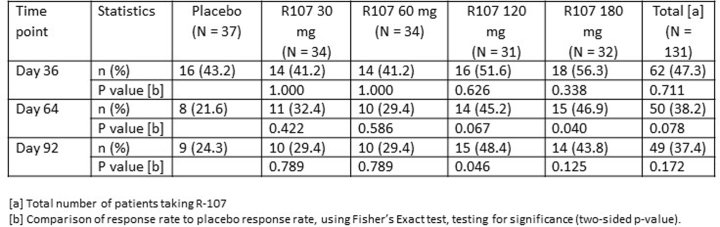
Patient response rates on Days 36, 64 and 92

**Extended Data Table 3:**
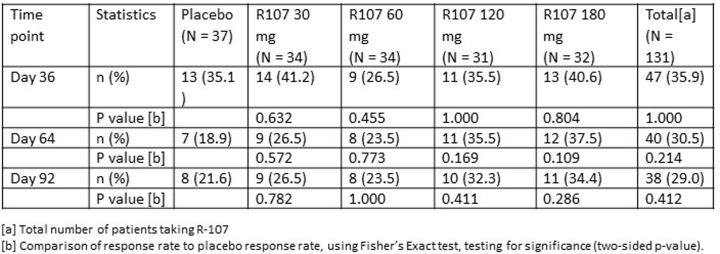
Patient remission rates on Days 36, 64 and 92

**Extended Data Table 4:**
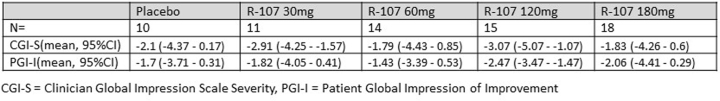
Change in CGI-S and PGI-I between Day 1 and Day 92 in patients who completed BEDROC

**Extended Data Table 5:**
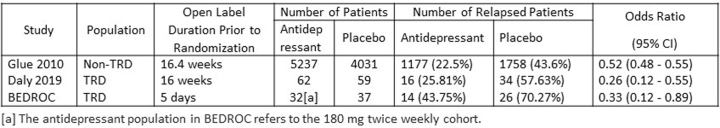
Comparison of relapse odds ratios in relapse prevention antidepressant trials in TRD and non-TRD patient populations

## Data Availability

Deidentified individual participant data and the data dictionary will be made available 24 months after publication. Proposals with specific aims and an analysis plan should be directed to P.S.

## References

[1] Nikolin, S. et al. Ketamine for the treatment of major depression: a systematic review and meta-analysis. *EClinicalMedicine* **62**, 102127 (2023). 37593223 10.1016/j.eclinm.2023.102127 PMC10430179

[2] Kim, J., Farchione, T., Potter, A., Chen, Q. & Temple, R. Esketamine for treatment-resistant depression—first FDA-approved antidepressant in a new class. *N. Engl. J. Med.* **381**, 1–4 (2019). 31116916 10.1056/NEJMp1903305

[3] McIntyre, R. S. et al. Synthesizing the evidence for ketamine and esketamine in treatment-resistant depression: an international expert opinion on the available evidence and implementation. *Am. J. Psychiatry* **178**, 383–399 (2021). 33726522 10.1176/appi.ajp.2020.20081251 PMC9635017

[4] Highland, J. N. et al. Hydroxynorketamines: pharmacology and potential therapeutic applications. *Pharm. Rev.* **73**, 763–791 (2021). 33674359 10.1124/pharmrev.120.000149 PMC7938660

[5] Paul, R. K. et al. (*R*,*S*)-Ketamine metabolites (*R*,*S*)-norketamine and (*2S*,*6S*)-hydroxynorketamine increase the mammalian target of rapamycin function. *Anesthesiology* **121**, 149–159 (2014). 10.1097/ALN.0000000000000285 PMC4061505 24936922

[6] Hasan, M. et al. Chiral pharmacokinetics and metabolite profile of prolonged-release ketamine tablets in healthy human subjects. *Anesthesiology* **135**, 326–339 (2021). 34019627 10.1097/ALN.0000000000003829

[7] Domany, Y. et al. Repeated oral ketamine for out-patient treatment of resistant depression: randomised, double-blind, placebo-controlled, proof-of-concept study. *Br. J. Psychiatry* **214**, 20–26 (2019). 30246667 10.1192/bjp.2018.196

[8] Glue, P., Russell, B. & Medlicott, N. J. Influence of formulation and route of administration on ketamine’s safety and tolerability: systematic review. *Eur. J. Clin. Pharm.* **77**, 671–676 (2021). 10.1007/s00228-020-03047-z 33210159

[9] Glue, P. et al. Ascending‐dose study of controlled‐release ketamine tablets in healthy volunteers: pharmacokinetics, pharmacodynamics, safety, and tolerability. *J. Clin. Pharm.* **60**, 751–757 (2020). 10.1002/jcph.1573 32065415

[10] Glue, P. W., Medlicott, N. J. & Surman, P. W. Extended release pharmaceutical formulation and methods of treatment. US Patent No. 11,471,416 (2022).

[11] Khan, A., Mar, K. F. & Brown, W. A. The conundrum of depression clinical trials: one size does not fit all. *Int. Clin. Psychopharm.* **33**, 239–248 (2018). 10.1097/YIC.0000000000000229 PMC6078483 29939890

[12] Khin, N. A., Chen, Y. F., Yang, Y., Yang, P. & Laughren, T. P. Exploratory analyses of efficacy data from major depressive disorder trials submitted to the US Food and Drug Administration in support of new drug applications. *J. Clin. Psychiatry* **72**, 464–472 (2011). 21527123 10.4088/JCP.10m06191

[13] Glue, P., Donovan, M. R., Kolluri, S. & Emir, B. Meta-analysis of relapse prevention antidepressant trials in depressive disorders. *Austral. NZ J. Psychiatry* **44**, 697–705 (2010). 10.3109/00048671003705441 20636190

[14] Temple, R. Enrichment of clinical study populations. *Clin. Pharmacol. Ther.* **88**, 774–778 (2010). 20944560 10.1038/clpt.2010.233

[15] Daly, E. J. et al. Efficacy of esketamine nasal spray plus oral antidepressant treatment for relapse prevention in patients with treatment-resistant depression: a randomized clinical trial. *JAMA Psychiatry* **76**, 893–903 (2019). 31166571 10.1001/jamapsychiatry.2019.1189 PMC6551577

[16] *Enrichment Strategies for Clinical Trials to Support Determination of Effectiveness of Human Drugs and Biological Products Guidance for Industry* (US FDA, 2019); www.fda.gov/media/121320/download

[17] Correll, G. E. & Futter, G. E. Two case studies of patients with major depressive disorder given low-dose (subanesthetic) ketamine infusions. *Pain Med* **7**, 92–95 (2006). 16533209 10.1111/j.1526-4637.2006.00101.x

[18] Brown, W. A. Expectation, the placebo effect and the response to treatment. *Rhode Isl. Med J.* **98**, 19–21 (2015). 25938400

[19] Melander, H., Salmonson, T., Abadie, E. & van Zwieten-Boot, B. A regulatory Apologia—a review of placebo-controlled studies in regulatory submissions of new-generation antidepressants. *Eur. Neuropsychopharm.* **18**, 623–627 (2008). 10.1016/j.euroneuro.2008.06.003 18621509

[20] Short, B., Fong, J., Galvez, V., Shelker, W. & Loo, C. K. Side-effects associated with ketamine use in depression: a systematic review. *Lancet Psychiatry* **5**, 65–78 (2018). 28757132 10.1016/S2215-0366(17)30272-9

[21] Sanacora, G. et al. Balancing the promise and risks of ketamine treatment for mood disorders. *Neuropsychopharmacology* **42**, 1179–1181 (2017). 27640324 10.1038/npp.2016.193 PMC5437876

[22] Montgomery, S. A. & Åsberg, M. A new depression scale designed to be sensitive to change. *Br. J. Psychiatry* **134**, 382–389 (1979). 444788 10.1192/bjp.134.4.382

[23] Schoevers, R. A., Chaves, T. V., Balukova, S. M. & Kortekaas, R. Oral ketamine for the treatment of pain and treatment-resistant depression. *Br. J. Psychiatry* **208**, 108–113 (2016). 26834167 10.1192/bjp.bp.115.165498

[24] Busner, J. & Targum, S. D. The clinical global impressions scale: applying a research tool in clinical practice. *Psychiatry* **4**, 28–37 (2007). 20526405 PMC2880930

[25] Blair, M. et al. Depressive symptoms negatively impact Montreal Cognitive Assessment performance: a memory clinic experience. *Can. J. Neurol. Sci.* **43**, 513–517 (2016). 26842678 10.1017/cjn.2015.399

[26] Posner, K. et al. *Columbia-Suicide Severity Rating Scale (C-SSRS)* 10 (Columbia University Medical Center, 2008).

[27] Humphrey, L. et al. The bladder pain/interstitial cystitis symptom score: development, validation, and identification of a cut score. *Eur. Urol.* **61**, 271–279 (2012). 22050826 10.1016/j.eururo.2011.10.004

[28] Overall, J. E. & Beller, S. A. The Brief Psychiatric Rating Scale (BPRS) in geropsychiatric research: I. Factor structure on an inpatient unit. *J. Gerontol.* **39**, 187–193 (1984). 6699374 10.1093/geronj/39.2.187

[29] Bremner, J. D. et al. Measurement of dissociative states with the clinician-administered dissociative states scale (CADSS). *J. Trauma Stress* **11**, 125–136 (1998). 9479681 10.1023/A:1024465317902

