# Supplementary material for: Extended-release ketamine tablets for treatment-resistant depression: a randomized placebo-controlled phase 2 trial
Source: Nat Med. 2024 Jun 24;30(7):2004–9. doi: 10.1038/s41591-024-03063-x (PMC11271411; doi:10.1038/s41591-024-03063-x)
Supplement: Supplementary file 1 — Clinical study protocol. [file 41591_2024_3063_MOESM1_ESM.pdf]

nature medicine

Supplementary information https://doi.org/10.1038/s41591-024-03063-x

Extended-release ketamine tablets for treatment-resistant depression: a randomized placebo-controlled phase 2 trial

In the format provided by the authors and unedited

SUPPLEMENTARY INFORMATION:
CLINICAL STUDY PROTOCOL

STUDY TITLE:

A Phase 2a Proof-of-Concept Study of R-107 for the Treatment of Refractory Major Depressive Disorder.

STUDY DESIGN:	2a
TARGET DISEASE AREA:	Major Depressive Disorder
DRUG SUBSTANCE	R-107
DURATION OF STUDY:	20 Weeks
PROTOCOL AUTHOR:	Subash Muniswamaiah, Clintec

Date of protocol: Final, 20 NOV 2020

SPONSOR

Douglas Pharmaceuticals Limited.
2 Te Pai Place
Henderson
Auckland, 0610
New Zealand
Tel.: +64 9 835 0660
Fax: +64 9 835 0665

Confidentiality Statement
This document is confidential and the property of Douglas Pharmaceuticals Ltd. No part of it may be transmitted, reproduced, published or used by any person/s without prior written authorisation from Douglas Pharmaceuticals Ltd.

Confidential © 2020 Clintec All Rights Reserved

ADMINISTRATIVE STRUCTURE

**Sponsor:**	Douglas Pharmaceuticals Limited<br>2 Te Pai Place<br>Henderson<br>Auckland, 0610<br>New Zealand<br>Tel.: +64 9 835 0660<br>Fax: +64 9 835 0665
**Sponsor's Authorised Representative:**	Blair Dickman<br>Senior Clinical Research Manager<br>Douglas Pharmaceuticals Ltd<br>2 Te Pai Place<br>Auckland 0610<br>New Zealand<br>clinical@douglas.co.nz
**Contract Research Organization (CRO):**	Clintec<br>133 Finnieston St.,<br>Glasgow, G3 8HB,<br>Scotland, United Kingdom<br>Tel: +44 (0) 141 226 1120<br>Fax: +44 (0) 141 248 8993<br>Email: 1489DOU_BEDROC@clintec.com
**CRO's Project Manager:**	Kylie Tarascio<br>Project Manager<br>Clintec International Pty Ltd<br>Level 8, 201 Pacific Highway<br>St Leonard's, NSW, 2065<br>Australia<br>Tel: +61 (02) 90168100<br>Email: ktarascio@clintec.com
**Protocol Author:**	Subash Muniswamaiah, Clintec

Confidential © 2017 Clintec All Rights Reserved

SPONSOR SIGNATURE PAGE

I have read this protocol and I agree to conduct the study as described, in compliance with Good Clinical Practice (ICH E6, R2), the Declaration of Helsinki and the current rules and regulations in force in the countries in which the study is being conducted.

I agree to maintain a list of appropriately qualified persons to whom I shall delegate significant trial-related duties. I shall ensure that all persons assisting me with the trial are adequately informed about the protocol and any amendments, and their trial-related duties and functions.

____

Signature of Sponsors' authorised signatory

Name: ____________________________

Date: ____________________________

Confidential © 2017 Clintec All Rights Reserved

INVESTIGATOR SIGNATURE PAGE

I have read and understood this protocol and I agree to conduct the study as described, in compliance with Good Clinical Practice (ICH E6, R2), the Declaration of Helsinki, and the rules and regulations in force in this country at present.

I agree to maintain a list of appropriately qualified persons to whom I shall delegate significant trial-related duties. I shall ensure that all persons assisting me with the trial are adequately informed about the protocol and any amendments, and their trial-related duties and functions. I also acknowledge that the Sponsor has the right to discontinue this study at any time.

Signature of Investigator
Name:
Date:

Confidential © 2017 Clintec All Rights Reserved

CRO SIGNATURE PAGE

I have read this protocol and I agree to conduct the study as described, in compliance with Good Clinical Practice (ICH E6, R2), the Declaration of Helsinki and the current rules and regulations in force in the countries in which the study is being conducted.

Signature of CRO's authorised signatory
Name:
Date:

Confidential © 2017 Clintec All Rights Reserved

BIOSTATISTICIAN SIGNATURE PAGE

I have read this protocol and I agree to conduct the study as described, in compliance with Good Clinical Practice (ICH E6, R2), the Declaration of Helsinki and the rules and regulations in force in this country at present.

Signature of CRO Biostatistician
Name:
Date:

Confidential © 2017 Clintec All Rights Reserved

<table>
<tr><th colspan="2">SYNOPSIS</th></tr>
<tr><td>STUDY TITLE:</td><td>Phase 2a Proof-of-Concept Study of R-107 for the Treatment of Refractory Major Depressive Disorder</td></tr>
<tr><td>STUDY OBJECTIVES:</td><td>Primary Objective

To evaluate the efficacy of extended release (ER) R-107 tablets (30 mg, 60 mg, 120 mg, 180 mg) as measured by the change in Montgomery-Asberg Depression Rating Scale (MADRS) score from baseline (Day 1) to Day 92 in subjects with Diagnostic and Statistical Manual of Mental Disorders, 5th edition (DSM-5) Treatment-Resistant Depression (TRD) who have responded to 1 weeks' treatment with R-107 120 mg tablets.

Secondary Objectives

• To evaluate the efficacy of ER R-107 tablets (30 mg, 60 mg, 120 mg, 180 mg) as measured by the change in MADRS total score from baseline (Day 1) to Day 36 and to Day 64 in subjects with TRD who have responded to 1 weeks' treatment with R-107 120 mg tablets;
• To assess the effect of R-107 tablets compared to placebo on response at Day 36, Day 64 and Day 92, where response is defined as ≥50% reduction from baseline (Day 1) in MADRS total score;
• To assess the effect of R-107 tablets compared to placebo on remission at Day 36, Day 64 and Day 92, where remission is defined as MADRS total score ≤10;
• To assess the effect of R-107 tablets compared to placebo on the severity of illness using the Clinician Global Impression – Severity (CGI-S) and the Patient Global Impression – Improvement (PGI-I);
• To evaluate the pharmacokinetics (PK) of R-107 tablets in subjects with TRD;
• To investigate the dose-response pattern for R-107 with respect to the primary efficacy outcome (MADRS) and safety outcomes.

Exploratory Objectives:

• Subject perspective of global improvement in TRD from baseline as measured by the Patient Global Impression-Improvement (PGI-I);
• Impact on health status as assessed using the EuroQol-5D, 5-level version (EQ-5D-5L) questionnaire;
• Subject perspective on impaired functioning during the double-blind phase of the study using the Work and Social Adjustment Scale (WSAS).
• To assess the effect of R-107 tablets compared to placebo on delaying relapse during the double-blind randomisation phase, where relapse is defined as MADRS total score ≥ 22.

Safety Objectives:

Part 1: Enrichment Open-label Phase
To evaluate the safety, tolerability and Psychiatric Pharmacodynamics (PD) of 120 mg of R-107 tablets in an open-label enrichment treatment phase in a population of 200 subjects meeting inclusion criteria.
• Adverse Events (AEs);
• Effect on heart rate, blood pressure, respiratory rate, temperature and blood oxygen saturation (SpO2);
• Effect on laboratory evaluations and physical examination.

Part 2: Randomised Double-Blind Treatment Phase
To evaluate the safety, tolerability and Psychiatric Pharmacodynamics (PD) of R-107 tablets in a treatment-enriched subject population with ketamine responsive TRD.
• AEs;</td></tr>
</table>

Confidential © 2017 Clintec All Rights Reserved

**SYNOPSIS**	
	• Effect on heart rate, blood pressure, respiratory rate, temperature and blood oxygen saturation (SpO2);<br>• Effect on laboratory evaluations and physical examination;<br>• Effects on suicidal ideation/behaviour measured by the Electronic Columbia Suicide Severity Rating Scale (eC-SSRS);<br>• Psychosis-like side effects by using a five-item positive symptom scale of the Brief Psychiatric Rating Scale (BPRS) consisting of: grandiosity, suspiciousness, hallucinations, unusual thought content, and conceptual disorganization;<br>• Effects on dissociative symptoms using the Clinician-Administered Dissociative States Scale (CADSS);<br>• Potential treatment-emergent symptoms of cystitis using the Bladder Pain/ Interstitial Cystitis Symptom Score (BPIC-SS);<br>• Effects on aspects of cognitive functioning (attention and memory), as assessed by the Montreal Cognitive Assessment (MoCA), Verbal Fluency (category and letter) and Symbol Digit Modalities Test (SDMT).
**STUDY DURATION:**	Approximately 20 Weeks.
**STUDY BACKGROUND:**	Major depressive disorder impairs socio-occupational functioning and increases suicide risk, adverse sequelae of other common comorbid medical conditions (e.g., cardiovascular disease, type 2 diabetes, and obesity), and mortality. Currently prescribed antidepressant treatments target the monoamine system only improves depressive symptoms in about 50% of the subjects (Undurraga, 2011). Subjects with Treatment Resistant Disease (TRD) have lower productivity, higher medical comorbidities, and more suicide attempts than those with Major Depressive Disorder (MDD) (Conway et al. 2017). TRD typically refers to depressive episodes that do not respond adequately to 2 or more trials of antidepressant monotherapy (Conway et al. 2017).<br><br>Despite the many treatment options available for clinically depressed patients, there is a need for more effective treatments, particularly for TRD and specifically for therapeutic options that lead to more rapid symptom resolution (Schwartz, 2016).<br><br>Since 2006, research on the use of ketamine at subanaesthetic doses (0.5 mg/kg) administered over a 40-minute intravenous (IV) infusion has demonstrated rapid onset of antidepressant effects on patients with TRD. Activation of synaptic plasticity by increasing brain-derived neurotrophic factor (BDNF) translation and secretion, as well as via glycogen synthase kinase-3 (GSK-3) inhibition are the possible mechanisms by which ketamine induces an antidepressant effect (Schwartz, 2016).<br><br>Ketamine has been shown in published clinical studies to exert antidepressant effects in subjects with TRD that are both rapid (in terms of onset) and durable (Muller et al. 2016). These studies generally involve single doses of ketamine given as an IV infusion over 40 minutes. The purpose of this study is to investigate the efficacy of extended release (ER) Ketamine in tablet form as a treatment for Treatment Resistant Major Depressive Disorder.
**OVERALL STUDY DESIGN:**	This is a multi-centre, Phase 2a study, which incorporates a 1-week enrichment open-label phase followed by randomised, double-blind, placebo-controlled phase, to investigate R-107 (30 mg, 60 mg, 120 mg or 180 mg) versus placebo in TRD subjects who respond to the 1-week enrichment open-label phase.<br><br>Each subject will participate in up to 4 phases:<br><br>• Phase 1: A screening phase of up to 4 weeks (Day -28 to -1);<br>• Phase 2: An enrichment phase: open-label treatment phase for 1 week (to identify responders for the enrichment population in the subsequent double-

Confidential © 2017 Clintec All Rights Reserved

**SYNOPSIS**	
	blind treatment phase);<br>• Phase 3: A double-blind treatment phase for 12 weeks (Day 8-92);<br>• Phase 4: A 4-week post-treatment (follow-up) phase.<br><br>The treatment section of the study will comprise of two parts:<br><br>**Part 1**: Enrichment open-label phase<br>• Approximately 200 subjects will be screened and selected into the enrichment phase consisting of an open-label treatment phase of 5 doses of R-107 at 120 mg (Days 1-5).<br>**Part 2**: Double-blind randomised treatment<br>• 150 subjects having a positive clinical response (≥50% reduction of their baseline Montgomery-Asberg Depression Rating Scale [MADRS] total score which is ≤12) in Part 1 will be randomised into 5 arms within the Part 2 double-blind treatment phase (Day 8-92).<br><br>Subjects will be screened from Day -28 to -1 prior to dose administration.<br>Study evaluations will be performed throughout the study as per the Study Schedule.<br><br>All participating subjects who have had at least one dose of R-107/ placebo will be followed up for safety for 4 weeks after their last dose.<br><br>**Part 1:** On Day 1, approximately 200 subjects will receive a single dose of R-107 120 mg (2 x 60 mg) and will complete procedures as detailed. Subjects will return to the study site on Days 2-5 for 4 additional single doses of R-107 120 mg. Subjects will remain in the study site for 4 hours post-dose on Day 1 and will complete procedures as detailed. Subjects will be given the telephone number of the study site which they are encouraged to call if they experience any adverse events on the remaining study days.<br><br>Subjects will return to the study site on Day 8 for evaluation of their response to treatment with the open-label R-107 120 mg for 5 days and randomisation if the response criteria is met.<br><br>**Part 2:** Following the enrichment open-label phase, 150 subjects with a clinical response, as demonstrated by a ≥50% reduction of their MADRS total score which is ≤12, will enter into Part 2 of the study. The subjects will be randomised on Day 8 into double-blind treatment with either placebo, R-107 30 mg, R-107 60 mg, R-107 120 mg or R-107 180 mg (n=30/treatment). A pre-dose blood sample will be obtained on Day 8 prior to study drug administration. Subjects will be dispensed tablets for their corresponding randomised R-107 dose or placebo followed by the completion of the safety and efficacy assessments including blood sample collection to assess PK at 4- and 24-hours post-dose.<br><br>Subjects will be followed up for safety at each visit and full complement of study safety parameters, laboratory assessments, procedures, and evaluations will be performed at the primary endpoint conclusion on Day 92 or at a subject's early study termination.<br><br>All adverse events and special events, whether serious or non-serious, will be reported from the time a signed and dated informed consent form (ICF) is obtained until completion of the subject's last study-related procedure (which may include contact for follow-up of safety).<br><br>The duration of the subject's participation will be approximately 20 weeks. The end of study will occur when the last subject in the study completes his/her last study assessment.<br><br>Subjects who withdraw or are withdrawn from the study prior to Day 92 may be eligible to enter a separate open-label extension (OLE) study, if the reason for

Confidential © 2017 Clintec All Rights Reserved

**SYNOPSIS**	
	withdrawal was a relapse of depressive symptoms during the double-blind treatment phase of the study. Subjects withdrawn because of safety or tolerability issues may also be eligible to enter a separate OLE study, if the issues were unrelated to R-107. The blind will not be broken for subjects withdrawing prematurely.
**STUDY POPULATION and SIZE:**	Approximately 200 subjects in the enrichment phase (Days 1-5), to allow randomisation of 150 subjects into the 5 arms of the double-blind phase (Days 8-92).
**INCLUSION CRITERIA**	The initial recruitment approach will be carried out by a registered nurse who will assist the Investigator in obtaining written informed consent and checking inclusion and exclusion criteria.<br><br>Subjects must meet all the following criteria:<br>1. Provision of written informed consent prior to any study specific procedures;<br>2. Subjects aged 18-80 years inclusive at the time of enrolment (screening visit);<br>3. Diagnosed with MDD as per Diagnostic and Statistical Manual of Mental Disorders, 5th edition (DSM-5) and Mini-International Neuropsychiatric Interview (MINI) 7.0.2 without psychotic features for at least three months prior to screening (comorbid anxiety disorders are also acceptable);<br>4. Montgomery Asberg Depression Rating Scale (MADRS) total score of ≥20 at screening and baseline;<br>5. Treatment resistance in major depression (TRD) defined as lack of clinically meaningful improvement despite the use of adequate doses of at least two antidepressant agents, derived from the group(s) of commonly used first line treatment, prescribed for adequate duration with adequate affirmation of treatment adherence[1]<br>6. Montreal Cognitive Assessment (MoCA) score ≥26 assessing cognitive function;<br>7. Psychotropic medication and/or psychotherapy is stable (i.e. no change of drugs or drug dose or visit schedule within 28 days prior to Day 1);<br>8. Subjects must weigh at least 50kg and have a Body Mass Index (BMI) between 18 and 40 kg/m^2 inclusive;<br>9. Subjects of childbearing potential (SOCBP) must use a highly effective form of birth control (confirmed by the Investigator). Highly effective forms of birth control include:<br>• True sexual abstinence (defined as refraining from sexual intercourse for the duration of the study and a minimum of 30 days following the last dose of study drug);<br>• Oral, intravaginal, or transdermal combined (oestrogen and progestogen containing) hormonal contraception associated with inhibition of ovulation;<br>• Oral, injectable, or implantable progestogen-only hormonal contraception associated with inhibition of ovulation;<br>• Intrauterine device (IUD)<br>• Intrauterine hormone-releasing system (IUS);<br>• Bilateral tubal occlusion;<br>• Vasectomised partner (provided that the partner is the sole sexual partner of the participant with childbearing potential and that the vasectomised partner has received medical assessment of the surgical success).

[1] Note: 'Lack of clinically meaningful improvement' is defined as failure of treatment to produce response or remission for patients and is determined after detailed clinical interview with the patient. In addition, patients are required to have depression of at least moderate severity, based on a MADRS score of 20 or greater.
'Adequate doses' is defined as the minimum therapeutic dose as per the product label OR maximum tolerated dose.
'Adequate duration' is defined as a minimum duration of 6 weeks.
'Adequate affirmation of treatment adherence' is affirmation sought during routine clinical interview (Fekadu, 2009).

Confidential © 2017 Clintec All Rights Reserved

**SYNOPSIS**	
	SOCBP are defined as subjects with a uterus who are NOT either permanently sterilized (hysterectomy, bilateral oophorectomy, or bilateral salpingectomy), or who are NOT postmenopausal. Subjects will be considered post-menopausal if they have been amenorrhoeic for 12 months prior to the planned date of randomisation without an alternative medical cause.<br><br>The following age-specific requirements apply:<br>• <50 years old would be considered postmenopausal if they have been amenorrhoeic for 12 months or more following cessation of exogenous hormonal treatment and follicle stimulating hormone (FSH) levels in the postmenopausal range (FSH level > 40 mIU/mL);<br>• ≥50 years old would be considered postmenopausal if they have been amenorrhoeic for 12 months or more following cessation of all exogenous hormonal treatment.<br>*Rhythm methods will not be considered as highly effective methods of birth control.*<br>10. Subjects with the ability to produce sperm must use an adequate method of contraception (condom or condom with spermicide depending on local regulations) from the first dose of Investigational Medicinal Product (IMP) until 30 days after their last dose. Subjects with a partner or partners who is (are) not of childbearing potential are exempt of these requirements;<br>11. Subjects with the ability to produce sperm must not donate sperm for at least 30 days post-dose of the last study treatment;<br>12. A subject with ability of producing eggs (ova, oocytes) must agree not to donate eggs for the purposes of assisted reproduction during the study and for at least 3 months after receiving the last dose of study drug;<br>13. Ability and willingness to attend the necessary visits to the study centre;<br>14. Ability to read and write;<br>15. Subject must be willing and able to adhere to the prohibitions and restrictions specified in the protocol;<br>16. Able to swallow tablets.<br>**Entry to Double-blind phase of the study at Day 8**<br>1. Subjects with a clinical response as demonstrated by ≥50% reduction of their MADRS total score which is ≤12 will enter into Part 2 of the study.
**EXCLUSION CRITERIA**	Subjects must NOT meet any of the following exclusion criteria to be enrolled:<br>1. Any significant disease or disorder (e.g., cardiovascular, pulmonary, gastrointestinal, hepatic, renal, neurological, musculoskeletal, endocrine, metabolic, malignant, psychiatric, major physical impairment) which, in the opinion of the Investigator, may either put the subject at risk because of participation in the study, or may influence the results of the study, or the subject's ability to participate in the study;<br>2. Contraindication to the use of R-107, e.g., any condition in which a significant elevation of blood pressure would be hazardous, such as decompensated heart failure, severe or poorly controlled hypertension (blood pressure systolic ≥160 or diastolic ≥90, tested on 2 or more occasions); within last 3 months, recent myocardial infarction, stroke, cerebral haemorrhage; myasthenia gravis; known allergy, hypersensitivity, or intolerance to ketamine or its excipients;<br>3. History of neurodegenerative disorder e.g. Alzheimer's disease, vascular dementia or Parkinson's disease;<br>4. Any clinically significant abnormal findings in physical examination, vital signs, haematology, clinical chemistry, or urinalysis during screening and at baseline, which in the opinion of the Investigator, may put the subject at risk because of their participation in the study, or may influence the results of the

Confidential © 2017 Clintec All Rights Reserved

SYNOPSIS	
	study, or the subject's ability to complete entire duration of the study.<br>5. Any electrocardiogram (ECG) abnormality obtained during the screening period that in Investigator's judgement may put the subject at risk or negatively affect the outcome of the study;<br>6. Subjects are excluded if they have any of the following:<br>• A history of known immunodeficiency disorder including a positive test for human immunodeficiency virus, HIV-1 or HIV-2;<br>• Positive hepatitis B surface antigen, or positive hepatitis C virus antibody serology, or a positive medical history for hepatitis B or C. Subjects with a history of hepatitis B vaccination without history of hepatitis B are allowed to enrol.<br><br>7. Hepatic Insufficiency: Alanine aminotransferase (ALT) or aspartate aminotransferase (AST) level ≥2 times the upper limit of normal (ULN) confirmed by repeated testing during screening period;<br>8. Pregnant, breastfeeding, or lactating subjects (subjects of childbearing potential must have a negative serum pregnancy test at screening and a negative urine pregnancy test on Day 1);<br>9. Significant current risk of suicide:<br>• As assessed by eC-SSRS (baseline), or<br>• Serious risk for suicide, as assessed by the evaluating study clinician; a serious suicide risk will be considered:<br>(a) an inability to control suicide impulses or imminent or unacceptably high risk of suicide in the Investigator's judgment; or<br>(b) a recent history of suicidal behaviour, which is defined as either one or more suicide attempts (or interrupted suicide attempts) in the 12 months before study entry; or<br>(c) history of serious suicidal behaviour, defined as one or more suicide attempts (or interrupted attempts) in the last 3 years with a potential lethality judged by the evaluating study clinician to have possibly resulted in serious injury or death.<br>10. History of alcohol or drug abuse within the past year, which may compromise the study data interpretation as judged by Investigator or Study Physician;<br>11. Subject has a positive test result(s) for drugs of abuse (including barbiturates, methadone, opiates, cocaine, phencyclidine, and amphetamine/methamphetamine) at screening or pre-dose on Day 1. In addition to the drugs of abuse previously mentioned, cannabinoids will also be tested on Day 1;<br>• Subjects that have a positive test result at screening due to prescribed opiates may be permitted to continue the screening phase if the prohibited medication is discontinued at least 1 week or 5 half-lives, whichever is longer, before the first dose of study medication. Provided the Day 1 pre-dose test for drugs of abuse result is negative, the subject may be enrolled. Retesting is not permitted for positive test result(s) from non-prescription use of drugs of abuse.<br>• Subjects who have a positive test result at screening due to prescribed psychostimulants (e.g., amphetamine, methylphenidate, etc.) taken for an indication other than MDD are permitted to continue to take this medication during the study.<br>• A positive test result for cannabinoids pre-dose on Day 1 is exclusionary.<br>12. Any clinically significant infection or febrile illness in the five days prior to dosing Day 1;<br>13. Past or current history of schizophrenia, bipolar disorder, ongoing severe personality disorder, ongoing post-traumatic stress disorder, intellectual disability or severe obsessive-compulsive disorder;<br>14. History of abuse of ketamine or phencyclidine;<br>15. Electroconvulsive therapy, transcranial magnetic stimulation, vagal nerve stimulation, deep brain stimulation or other brain stimulation treatment within

Confidential © 2017 Clintec All Rights Reserved

**SYNOPSIS**	
	the past 4 weeks or currently used as either an acute or maintenance treatment of depression;<br>16. Receipt of any investigational product within 30 days or 5 half-lives prior to dosing; whichever is longer;<br>17. Subject has a history of malignancy within 5 years before screening (exceptions are squamous and basal cell carcinomas of the skin and carcinoma in situ of the cervix, or malignancy that in the opinion of the Investigator, with concurrence with the Sponsor's medical monitor, is considered cured with minimal risk of recurrence);<br>18. Subjects should not consume grapefruit, grapefruit juice or Seville oranges for 72 hours before R-107 administration and during the study;<br>19. Subject has received any disallowed therapies as noted in the protocol (Restricted Medication), Pre-study and Concomitant Therapy before the specific time relative to the planned first dose of study drug;<br>20. Subject has had major surgery, (e.g., requiring general anaesthesia) within 2 weeks before screening, or will not have fully recovered from surgery, or has surgery planned during the time the subject is expected to participate in the study. Note: subjects with planned surgical procedures to be conducted under local anaesthesia may participate;<br>21. Employees of the clinical study centre or family members (first-degree relatives) of such individuals or anyone involved in the planning and/or conduct of the study;<br>22. Subjects who do not consent to their General Practitioner being contacted prior to the commencement of the study, if necessary, about their medical history or after the study about any adverse results or reactions;<br>23. Subjects who, in the opinion of the Investigator, do not understand the information and procedures of the study, or would not be compliant with them (in particular the study restrictions and risks involved).<br><br>Investigators should ensure that all study enrolment criteria have been met at screening. If a subject's status changes (including laboratory results or receipt of additional medical records) after screening but before the first dose of study drug is given such that they no longer meet all eligibility criteria, then the subject should be excluded from participation in the study.
**Treatment Groups**	**Part 1:** Approximately 200 TRD subjects to receive open-label R-107 120 mg (2 x 60 mg) oral tablets per day for 5 days. Enrolment to Part 1 of the study will continue until 150 responders enter Part 2 of the study.<br><br>**Part 2:** 150 subjects responding to the open-label Part 1 portion will be randomised to one of 5 treatment groups of 30 subjects each (placebo, R-107 30 mg, R-107 60 mg, R-107 120 mg and R-107 180 mg). Study drug will then be taken orally twice per week for 12 consecutive weeks.
**Endpoint Measures**	**MADRS**<br><br>Montgomery-Asberg Depression Rating Scale (MADRS), a validated and reliable clinician assessed ten-item diagnostic questionnaire used to measure the severity of depressive episodes in subjects with mood disorders. The questionnaire includes questions on the following symptoms: 1. Apparent sadness 2. Reported sadness 3. Inner tension 4. Reduced sleep 5. Reduced appetite 6. Concentration difficulties 7. Lassitude 8. Inability to feel 9. Pessimistic thoughts 10. Suicidal thoughts. Each item yields a score of 0 to 6. The overall score ranges from 0 to 60. Higher MADRS score indicates more severe depression.<br><br>In depression, ‘response’ is commonly defined as a >50% reduction in the initial symptom score and remission is typically defined as a total score of ≤10. The primary efficacy evaluation will be the change from baseline in the MADRS total score in each period in the double-blind treatment phase.

Confidential © 2017 Clintec All Rights Reserved

SYNOPSIS	
	**PGI-I**<br><br>Patient Global Impression-Improvement Score (PGI-I), a 7-point scale that requires the subject to assess how much their illness has improved or worsened relative to a baseline state at the beginning of the intervention. Possible ratings are:<br><br>1. Very much improved<br>2. Much improved<br>3. Minimally improved<br>4. No change<br>5. Minimally worse<br>6. Much worse<br>7. Very much worse<br><br>**CGI-S**<br><br>The clinical global impression-severity score (CGI-S) is a well-established rating tool for many psychiatric disorders that can be utilised quickly in a clinical environment. The CGI-S provides an overall clinical summary measure, considering all available information including symptoms, subject history and psychological circumstances. The CGI evaluates the severity of psychopathology from 1 to 7.<br><br>**PGI-S**<br><br>The Patient Global Impression scales (PGI-S) will provide an overall patient-rated summary measure that assesses the severity of the subject's depression.<br><br>**EQ-5D-5L**<br><br>The EQ-5D is a standardized 2-part instrument for use as a measure of health outcome, primarily designed for self-completion by respondents. The EQ-5D-5L descriptive system comprises the following 5 dimensions: mobility, self-care, usual activities, pain/discomfort and anxiety/depression. Each dimension has 5 levels: no problems, slight problems, moderate problems, severe problems, and extreme problems. The descriptive system can be represented as a health state. The second part is the EQ-VAS, a visual analogue scale on which respondents are asked to rate their current state of health on a scale between 0 and 100.<br><br>**WSAS**<br><br>The Work and Social Adjustment Scale (WSAS) is a simple, reliable and valid measure of impaired functioning. It is a sensitive and useful outcome measure offering the potential for readily interpretable comparisons across studies and disorders.<br><br>**Pharmacokinetic Assessments**<br><br>A total of 9 PK samples will be obtained on Days 8, 9, 64, 65, 92 and 93 using a sparse sampling strategy, for population PK analysis<br><br>**CADSS**<br><br>Clinician-Administered Dissociative States Scale (CADSS), a 19-item scale with subject-rated items which are administered by a clinician. It is an instrument for the measurement of present-state dissociative symptoms.<br><br>**eC-SSRS**<br><br>The electronic Columbia-Suicide Severity Rating Scale (eC-SSRS) is a suicidal ideation rating scale created to evaluate suicidality (Baseline and 'since last visit'). It is a computer-automated, patient-reported version of the C-SSRS, in which the user's response to a question prompts and shows the appropriate follow-up questions (if any). It rates an individual's degree of suicidal ideation with "yes" or "no" questions revolving around thoughts about wanting to be dead and actions the

Confidential © 2017 Clintec All Rights Reserved

SYNOPSIS	
	individual may have actually done, or preparations made in an attempt of suicide. Each question addresses a different component of the respondent's suicide ideation severity.<br>**Safety Monitoring:**<br>• Evaluations of adverse events, concomitant medications, physical examination findings, vital signs (oral body temperature, pulse rate [radial], respiratory rate, blood pressure and blood oxygen saturation), body weight and 12-lead ECGs will be performed throughout the study to monitor subject safety.<br>• Safety laboratory tests includes haematology, serum chemistry and urinalysis assessments.<br>**BPRS**<br>The Brief Psychiatric Rating Scale (BPRS) is a rating scale to measure positive psychiatric symptoms such as grandiosity, suspiciousness, hallucinations, unusual thought content and conceptual disorganisation. Each symptom is rated 1-7 where it will be administered to assess treatment-emergent psychotic symptoms.<br>**BPIC-SS**<br>Bladder Pain/ Interstitial Cystitis Symptom Score (BPIC-SS) a systematic scoring questionnaire to assess a subject's bladder pain or cystitis symptoms. It records 5 questions scored 0-5 for never to always, 2 questions scored 0-4 for not at all to a great deal, and 1 global question recording the worst bladder pain in the last 7 days from 0-10. It provides a total scoring range from 0-38.<br>**Verbal Fluency (category and letter)**<br>Verbal fluency tests are a psychological test in which participants have to produce as many words as possible from a category or a letter in a given time (usually 60 seconds). This category can be semantic, including objects such as animals or fruits. Letter fluency will be assessed by Controlled Oral Word Association Test. Performance measure is the total number of correct non-repetitive words produced in 60 seconds.<br>**SDMT**<br>SDMT detects cognitive impairment and is used by clinicians to screen for organic cerebral dysfunction in both children (eight years and older) and adults. The SDMT is sensitive to detecting presence of brain damage and also changes in cognitive functioning over time and in response to treatment. The SDMT involves a simple substitution task that normal children and adults can easily perform. Using a reference key, the test taker has 90 seconds to pair specific numbers with given geometric figures.<br>**MoCA**<br>MoCA is a brief cognitive screening tool for Mild Cognitive Impairment. It was validated in the setting of mild cognitive impairment and has subsequently been adopted in numerous other settings clinically. The MoCA test is a one-page 30-point test administered in approximately 10 minutes. Different versions of the test should be used at different study visits in order to avoid learning effects. MoCA scores range between 0 and 30. A score of 26 or over is considered to be normal. Thirty items assessing multiple cognitive domains are contained in the MoCA:<br>• short-term memory (5 points);<br>• visuospatial abilities via clock drawing (3 points), and a cube copy task (1 point);<br>• executive functioning via an adaptation of Trail Making Test Part B (1 point), phonemic fluency (1 point), and verbal abstraction (2 points);<br>• attention, concentration, and working memory via target detection (1 point),

Confidential © 2017 Clintec All Rights Reserved

SYNOPSIS	
	serial subtraction (3 points), digits forward (1 point), and digits backward (1 point);<br>• language via confrontation naming with low-familiarity animals (3 points), and repetition of complex sentences (2 points);<br>• orientation to time and place (6 points).<br><br>Different versions of the tool are available and should be used at different visits in order to avoid learning effects. The administration sequence of the MoCA assessment will be from Version 8.1 to Version 8.2 to Version 8.3, back to Version 8.1 and so on. The version of the MoCA assessment used at the specified study visit should differ from and will depend on the last version of MoCA administered to the subject, in order to identify any emerging cerebral dysfunction, including short-term memory and visuospatial processing.<br><br>**Screening Assessments**<br>At screening the subjects will be evaluated for diagnosis of MDD and TRD by MINI 7.0.2. Additionally, TRD assessment will be performed using Maudsley Staging Method (MSM).<br>**MINI version 7.0.2**<br>MINI is used to assess the 17 most common psychiatric disorders in DSM-III-R, DSM-IV and DSM-5 and ICD-10. The MINI is designed as a brief structured diagnostic interview to meet the need for a short but accurate structured psychiatric interview for multicentre clinical trials and epidemiology studies and to be used as a first step in outcome tracking in non-research clinical settings. The MINI is a structured interview in which patients are asked to answer questions "Yes" or "No" (e.g., "Were you ever depressed or down, or felt sad, empty or hopeless most of the day, nearly every day, for two weeks?"). The MINI is designed to map onto diagnoses defined by the DSM-5. For the current study the following modules will be completed:<br>• Major Depressive Episode (Module A)<br>• Suicidality (Module B)<br>• Manic Episode (Module C)<br>• Any Psychotic disorders (Module K)<br>• Alcohol use disorder (Module I)<br>• Substance use disorder (Module J)<br>**MSM**<br>MSM was developed to support the effort to better understand and stage TRD. The first and the core dimension is treatment failure. The MSM incorporates severity (dimension 2) and duration of the depressive episode (dimension 3) as important dimensions to quantify treatment-resistance. The MSM defines treatment-resistance as: failure to attain significant level of improvement (equated with clinical remission) from an accurately defined depressive episode following treatment with an antidepressant medication given at an adequate (minimum effective) dose for a minimum of six weeks.
**PLANNED STATISTICAL ANALYSIS:**	The primary analysis of efficacy will be performed on the change in MADRS from baseline to Day 92 and will be assessed with Mixed Model Repeated Measures (MMRM) method, with treatment, time and the interaction of treatment with time as fixed effects, subject as random effect and baseline MADRS as a

Confidential © 2017 Clintec All Rights Reserved

**SYNOPSIS**	
	covariate. The least square mean differences between each active treatment with placebo will be presented along with a 95% confidence interval. The four treatment comparisons from the primary efficacy analysis at Day 92 are:<br>• R-107 180 mg vs. placebo<br>• R-107 120 mg vs. placebo<br>• R-107 60 mg vs. placebo<br>• R-107 30 mg vs. placebo<br><br>To control family-wise type I error, a fixed closed test procedure will be used for hypothesis testing. Starting with the highest dose of R-107, 180 mg will be compared with placebo as the first step. Only if this comparison is statistically significant at the 2-sided 5% level will R-107 120 mg be compared with placebo. Only if the R-107 120 mg dose group is statistically significant to placebo will R-107 60 mg be compared with placebo. Only if the R-107 60 mg dose group is statistically significant to placebo will R-107 30 mg be compared with placebo. A 2-sided significance level of 5% will be used for each comparison. The primary analysis will be performed using the Full Analysis Set with sensitivity analysis performed using the Per Protocol population, as supportive analysis.
**STUDY ETHICS:**	This study will be conducted according to the principles of the Declaration of Helsinki, ICH-GCP and in compliance with all relevant guidelines and Ethics Committee / Regulatory approval specific to each country.
**STUDY SPONSOR:**	Douglas Pharmaceuticals Limited<br>2 Te Pai Place<br>Henderson Auckland, 0610<br>New Zealand

Confidential © 2017 Clintec All Rights Reserved

TABLE OF CONTENTS

1 INTRODUCTION 24
1.1 Background of the Study 24
1.2 Study Rationale 25
1.3 Risk-Benefit Assessment 26
2 STUDY OBJECTIVES AND ENDPOINTS 30
2.1 Objectives 30
2.1.1 Primary Objectives 30
2.1.2 Secondary Objectives 30
2.1.3 Exploratory Objectives 30
2.1.4 Safety Objectives 30
2.2 Endpoints 31
2.2.1 Primary Endpoints 31
2.2.2 Secondary Endpoints 31
2.2.3 Exploratory Endpoints 31
2.2.4 Safety Endpoints 32
3 INVESTIGATIONAL PLAN 33
3.1 Overall Study Design 33
3.2 Study Flow Chart and Timelines 35
4 STUDY POPULATION 40
4.1 Subject Selection 40
4.2 Inclusion Criteria 40
4.3 Exclusion Criteria 42
4.4 Discontinuation of Study Medication 45
4.5 Withdrawal of Subjects 45
4.6 Ethical and Regulatory Considerations 46
4.6.1 Study-Specific Design Considerations 46
4.6.2 Declaration of Helsinki and ICH-GCP 47
4.6.3 Protocol Amendments 47
4.7 Protocol Deviations 47
4.8 Informed Consent 48
4.9 Subject Information 49
5 DESCRIPTION OF STUDY MEDICATION 50
5.1 Characterisation of Study Medication 50
5.2 Packaging, Labelling and Storage 51
5.3 Dispensing 51
5.4 Drug Accountability 52
6 STUDY DRUG TREATMENT 53
6.1 Drug Administration 53
6.2 Concomitant Indications and Therapy 53
6.3 Rescue Medication 55

Confidential © 2017 Clintec All Rights Reserved

7 STUDY DATA COLLECTION 57
7.1 Study Visits 57
7.1.1 Screening 57
7.1.2 Day 1 Visit 58
7.1.3 Open-Label Treatment Phase - Days 2-5 59
7.1.4 Early Termination 59
7.1.5 Randomisation 60
7.1.6 Randomised, Double-blind Treatment Phase 60
7.1.7 Treatment Phase - Check-out Visits 62
7.1.8 Safety Follow-up Visit 62
7.2 Study Assessments 63
7.2.1 Written Informed Consent 63
7.2.2 Demography 63
7.2.3 Physical Examination 64
7.2.4 Vital Signs 64
7.3 Laboratory Assessments 64
7.3.1 Haematology Tests 64
7.3.2 Serum Chemistry 65
7.3.3 Urinalysis Tests 65
7.3.4 Serum Pregnancy Test 65
7.3.5 Pregnancy Urine Test 65
7.3.6 FSH Test 65
7.3.7 Hepatitis and HIV 65
7.4 Efficacy Assessments 65
7.4.1 MADRS 65
7.4.2 PGI-I 66
7.4.3 PGI-S 66
7.4.4 CGI-S 66
7.4.5 EQ-5D-5L 67
7.4.6 WSAS 67
7.5 Safety Assessments 67
7.5.1 BPRS 67
7.5.2 CADSS 67
7.5.3 eC-SSRS 68
7.5.4 BPIC-SS 68
7.5.5 Verbal Fluency (category and letter) 68
7.5.6 SDMT 68
7.5.7 Electrocardiogram (ECG) 68
7.5.8 MoCA 69
7.6 Screening Assessments 69
7.6.1 MINI version 7.0.2 69
7.6.2 MSM 70
7.7 Pharmacokinetic (PK) Assessments 70
8 ADVERSE EVENTS 72
8.1 Documentation and Reporting of AEs 72
8.2 Follow-up of Adverse Events 74
8.3 Serious Adverse Events 74
8.4 Pregnancy Reporting 75

Confidential © 2017 Clintec All Rights Reserved

8.5 Significant Safety Issues and Urgent Safety Measures. 76
8.6 Disease-Related Events 77
8.7 Special Reporting 77
8.8 Data Safety Monitoring Committee 78
8.9 Medical monitoring 79
9 DATA HANDLING, RECORDING and QUALITY ASSURANCE 80
9.1 Electronic Case Record Forms 80
9.2 Source Documentation 80
9.3 Data Handling and Record Keeping 81
9.4 Monitoring 81
9.5 Data Management 82
9.5.1 Responsibilities 82
9.5.2 Data collection and validation 82
9.5.3 Data coding 82
9.6 Quality Assurance 83
10 DATA ANALYSIS AND STATISTICAL CONSIDERATIONS 84
10.1 Determination of Sample Size 84
10.2 Statistical Methods 84
10.2.1 Analysis Populations 84
10.2.2 Randomization Scheme 85
10.2.3 Number of Subjects Per Site 85
10.2.4 Primary Endpoint 85
10.2.5 Secondary Endpoints 86
10.2.6 Exploratory Endpoints 87
10.2.7 Safety Endpoints 87
10.2.8 General Considerations 88
10.3 Presentation of Results 88
10.4 Handling of Missing Data 88
11 STUDY ADMINISTRATIVE PROCEDURES 89
11.1 Regulatory Authority Approval 89
11.2 Confidentiality of Subjects 89
11.3 Delegation of Investigator Responsibilities 89
11.4 Central Laboratory 89
11.5 Coding and Decoding of subject data 89
11.5.1 Randomisation 89
11.5.2 Blinding 90
11.5.3 Unblinding 90
11.6 Insurance and Financial Arrangements 91
11.7 Study Termination and Site Closure 91
11.8 Archiving and Record Retention 92
11.9 Study Report and Publication Policy 93
12 REFERENCES 94
13 APPENDICES 97
13.1 Appendix 1 MADRS 97

Confidential © 2017 Clintec All Rights Reserved

13.2 Appendix 2 PGI-I ... 101
13.3 Appendix 3 PGI-S ... 102
13.4 Appendix 4 CGI-S ... 103
13.5 Appendix 5 EQ-5D-5L ... 104
13.6 Appendix 6 WSAS ... 107
13.7 Appendix 7 BPRS ... 108
13.8 Appendix 8 CADSS ... 112
13.9 Appendix 9 eC-SSRS ... 117
13.10 Appendix 10 BPIC-SS ... 140
13.11 Appendix 11 Verbal Fluency (Category and letter) ... 141
13.12 Appendix 12 Symbol Digit Modalities Test ... 145
13.13 Appendix 13 Mini-International Neuropsychiatric Interview (version 7.0.2) ... 147
13.14 Appendix 14 Maudsley Staging Method ... 166
13.15 Appendix 15 Montreal Cognitive Assessment ... 167
13.16 Appendix 16 Declaration of Helsinki ... 183

List of Tables

Table 1: Study Schedule – Part 1 (Enrichment Open-Label R-107 120 mg QD x 5D) ... 37
Table 2: Study Schedule – Part 2 (Randomisation, double-blind treatments) ... 39
Table 3: Study Placebo for Ketamine 60 mg Extended Release Tablet Composition: ... 50
Table 4: Study Drug (Ketamine 30 mg Extended Release Tablet) Composition: ... 50
Table 5: Study Drug (Ketamine 60 mg Extended Release Tablet) Composition: ... 51
Table 6: Study Medication Supplies ... 52
Table 7: Concomitant Therapies ... 54
Table 8: Approximate Volume of Blood to be Collected from each subject. ... 70

List of Figures

Figure 1: Study Timeline ... 35
Figure 2: Subject Visit and Dosing Schedule ... 35
Figure 3: Subject Visit and Telephone Follow-up Schedule (representative). ... 36
Figure 4: Structural Formula of Ketamine. ... 50

Confidential © 2017 Clintec All Rights Reserved

ABBREVIATIONS

AE	Adverse Event
BMI	Body Mass Index
BP	Blood Pressure
BPIC-SS	Bladder Pain/Interstitial Cystitis Symptom Score
BPRS+	Brief Psychiatric Rating Scale Positive Symptom subscale
CADSS	Clinician-Administered Dissociative States Scale
CBT	Cognitive Behavioural Therapy
CGI-S	Clinician Global Impression – Severity Score
CRF	Case Report Form
CRO	Contract Research Organization
DRE	Disease-related event
DSM-5	Diagnostic and Statistical Manual of Mental Disorders, 5th edition
EC	Ethics Committee
ECG	Electrocardiogram
eCRF	Electronic Case Report Forms
eC-SSRS	Electronic Columbia-Suicide Severity Rating Scale
ECT	Electroconvulsive therapy
EDC	Electronic Data Capture.
EOS	End of Study Visit
ER	Extended Release
FDA	Food and Drug Administration
FSH	Follicle Stimulating Hormone
GCP	Good Clinical Practice
HIV	Human immunodeficiency virus
ICF	Informed Consent Form
ICH	International conference on harmonization of technical requirements for registration of pharmaceuticals for human use
IMP	Investigational Medicinal Product
IRB	Institutional Review Board
IUD	Intrauterine Device
MADRS	Montgomery-Asberg Depression Rating Scale

Confidential © 2017 Clintec All Rights Reserved

MedDRA	Medical Dictionary for Regulatory Activities
MDD	Major Depressive Disorder
MINI	Mini-International Neuropsychiatric Interview
MoCA	Montreal Cognitive Assessment
MSM	Maudsley Staging Method
N	Sample Size
PD	Pharmacodynamics
PGI-I	Patient Global Impression - Improvement Score
PK	Pharmacokinetics
PGI-S	Patient Global Impression - Severity Score
PTE	Pre-Treatment Event
QD	Daily
REC	Research Ethics Committee
SAE	Serious Adverse Event
SD	Standard Deviation
SDMT	Symbol Digit Modalities Test
SIS	Subject Information Sheet
SOCBP	Subject of Child-Bearing Potential
SOP	Standard Operating Procedure
TEAE	Treatment Emergent Adverse Event
TRD	Treatment-Resistant Depression
ULN	Upper Limit of Normal
WHO	World Health Organization
WSAS	Work and Social Adjustment Scale

Confidential © 2017 Clintec All Rights Reserved

1 INTRODUCTION

1.1 Background of the Study

Approximately 350 million people are affected by Unipolar major depressive disorder (MDD) globally. MDD is one of the leading causes of disability, associated with harmful consequences to the well-being of individuals affected as well as the society (Marcus, WHO; Papakostas and Ionescu, 2015). MDD impairs socio-occupational functioning and increases suicide risk, adverse sequelae of other common comorbid medical conditions (e.g., cardiovascular disease, type 2 diabetes, and obesity), and mortality. There are numerous pharmaceutical products available to treat depression, as well as non-pharmacological therapies (ego, Cognitive Behavioural Therapy [CBT] and physical treatments such as Electroconvulsive Therapy [ECT]) Preston, 2013; Trivedi et al., 2006). Currently prescribed antidepressant treatments targeting the monoamine system only improves depressive symptoms in about 50% of the patients (Undurraga, 2011). Furthermore, in patients who had already failed to respond after two or more antidepressant treatments at adequate doses and duration (i.e., treatment-resistant depression, TRD), the rates are significantly lower (Malhi and Byrow, 2016). As a result, patients are exposed to long courses of anti-depressant treatments which prove ineffective. Currently approved antidepressants with action on the monoamine system have a longer time to onset of response, 6–12 weeks (Schwartz, Murrough and Iosifescu, 2016).

Of patients with MDD, approximately 10% to 30% do not improve or show only a partial response (coupled with functional impairment, poor quality of life, suicidal ideation, and a high relapse rate) to available treatments (Al-Harbi, 2012). Patients with TRD have lower productivity, higher medical comorbidities, and more suicide attempts than those with MDD (Conway et al. 2017). TRD typically refers to depressive episodes that do not respond adequately to 2 or more trials of antidepressant monotherapy (Conway et al. 2017). However, the definition for treatment failure (in terms of number of trials failed) has not been standardized (Conway et al. 2017; Rush et al. 2006), and treatment resistance is sometimes conceptualized as a disease continuum with staging systems based on the number and types of treatment that failed to resolve the depressive episode(s). Defining treatment resistance is further complicated by the lack of consensus in describing acute antidepressant responses (Conway et al. 2017). Standard clinical practice for obtaining a treatment history for patients who may be treatment resistant is the use of a clinical interview in conjunction with a review of a patient's medical record. The general treatment strategy for patients failing an initial treatment with an antidepressant medication is as follows:

- Switching treatment (e.g., to a different antidepressant, CBT, ECT, repetitive transcranial magnetic stimulation)

OR

- Addition of a treatment to an ineffective antidepressant (i.e., "augmentation"; either medication or psychotherapy) regimen

These options are generally considered to be comparable and multiple practice guidelines, including those of the American Psychiatric Association (APA), suggest both switching and

Confidential © 2017 Clintec All Rights Reserved

augmentation approaches as acceptable treatment options for patients with TRD (American Psychiatric Association 2010). Furthermore, in patients with established TRD, the APA recognizes ECT as the most effective form of therapy. ECT, while considered effective with both high response and remission rates, requires patients to undergo anaesthesia for the procedure and is associated with cognitive side effects. A short-term course of ECT typically consists of 6 to 12 treatments, administered 2 to 3 times per week until an effect is achieved.

Despite the many treatment options available for clinically depressed patients, there is a need for more effective treatments, particularly for TRD and specifically for therapeutic options that lead to more rapid symptom resolution (Schwartz, Murrough and Iosifescu, 2016).

Recent advances have begun to explore this common, yet debilitating illness. Available reports indicate a specific region of brain that is responsible for depression, as well as neuronal atrophy, including loss of synapses in MDD and rodent chronic stress models (Manji, Drevets and Charney, 2001; Price and Drevets, 2012). Converging lines of evidence suggest that MDD is associated with abnormalities in glutamatergic synaptic transmission resulting in loss of synaptic plasticity in mood and emotion circuits (Kavalali, 2012; Sanacora et al., 2008). Ketamine is an ionotropic glutamatergic N-methyl-d-aspartate receptor antagonist, on the WHO Essential Medicine List used as an anaesthetic agent and prescribed off-label for treating chronic pain. Since 2006, research on the use of ketamine at subanaesthetic doses (0.5 mg/kg) administered over a 40-minute intravenous (IV) infusion has demonstrated rapid onset of antidepressant effects on patients with TRD. Activation of synaptic plasticity by increasing brain-derived neurotrophic factor (BDNF) translation and secretion, as well as via glycogen synthase kinase-3 (GSK-3) inhibition are the possible mechanisms by which ketamine induces an antidepressant effect (Schwartz, Murrough and Iosifescu, 2016).

Ketamine has been shown in published clinical studies to exert antidepressant effects in patients with TRD that are both rapid (in terms of onset) and durable (Muller et al. 2016). These studies generally involve single doses of ketamine given as an intravenous infusion over 40 minutes. Intravenous ketamine, though, is rapidly cleared from the body (terminal half-life of 2.5 h). Despite its relatively short half-life, the antidepressant effects of IV ketamine persist for 3 to 7 days in a majority of patients. This durability of mood response after single-dose administration of ketamine suggests that ketamine initiates a cascade of events that is sustained following metabolism and excretion of the drug (Zarate and Machado-Vieira 2017; Sanacora and Schatzberg, 2015). One potential mechanism involves phosphorylation of mechanistic target of rapamycin (mTOR), which is increased with both ketamine and norketamine administration (Paul et al. 2014). Other groups have identified another ketamine metabolite, hydroxynorketamine, as another possible active metabolite (Zarate and Machado-Vieira 2017).

1.2 Study Rationale

Douglas Pharmaceuticals Ltd is aiming to solve the unmet need in the treatment of TRD by developing R-107, an extended release (ER) oral dosage formulation of ketamine. R-107 is designed to prolong the plasma concentrations of ketamine. R-107 contains both stereoisomers of ketamine (R- and S-) as a racemic mixture. The elimination half-life following administration of R-107 is approximately 8 h (Study ZPS-603), compared with the terminal elimination half-life after intravenous administration of 2.5 h. The apparent absorption rate constant (ka) is much slower than the elimination rate constant (ke), demonstrating “flip-flop”

Confidential © 2017 Clintec All Rights Reserved

kinetics [ka << ke] for ketamine when administered as R-107. Additional benefits of oral dosing of ketamine include reduced or absent reports of dissociation post-dose (these are marked after iv dosing) and the convenience of oral dosing compared with administering ketamine in a 40-minute iv infusion. Study ZPS-603 has preliminary open-label efficacy data in a cohort of subjects with TRD. Five subjects with TRD, whose pre-study Montgomery-Asberg Depression Rating Scale (MADRS) scores were at least 20, received single and multiple doses of R-107 ranging from 60 mg to 240 mg, administered every 12 h for 72 h. At 96 h, all 5 subjects had > 50% reduction in MADRS scores (pre-dose mean = 25; 96 h mean = 7.6; mean percent change = 69.6%). Adverse events (AE) in this cohort, which involved daily doses up to 240 mg by Day 4 in all subjects, included dissociation of mild intensity (1 subject) and headache (2 subjects). Three of these 5 subjects subsequently completed an open-label extension (OLE) treatment period, where they received R-107 (120 mg-240 mg once or twice weekly) for 3 months. Their mean MADRS score at the end of this 3-month period was 2.7.

Based upon the preceding data, Douglas Pharmaceuticals proposes R-107 would provide a meaningful clinical benefit relative to alternative treatments for TRD, such as ECT, in that it does not require hospitalization (consequent to anaesthesia as required for ECT) for treatment to be administered. Furthermore, R-107 appears to be effective when dosed once or twice weekly, demonstrating improved convenience of administration compared with ECT.

1.3 Risk-Benefit Assessment

Treatment with investigational drugs are associated with the inherent risk of both anticipated and unanticipated adverse outcomes. The known risks associated with the off-label use of ketamine in depression have been considered in the design of this study. The protocol has been designed in such a way to mitigate or completely avoid these risks. The benefits associated with use of ketamine outweigh the risks. With the use of sub-anaesthetic dose of ketamine, the risk of associated AEs is substantially lowered. The study will actively monitor subjects for associated risks as follows:

- Capturing clinically relevant treatment emergent adverse events (TEAEs) of special interest will be examined separately grouped in the following categories: drug abuse, including overdose, dependence, withdrawal, inadvertent or accidental exposure and medication error involving R-107;
- Electronic Columbia-Suicide Severity Rating Scale (eC-SSRS) will be performed to assess suicidal ideation and behaviour;
- Clinician-Administered Dissociative States Scale (CADSS) will be administered to assess treatment-emergent dissociative symptoms;
- Brief Psychiatric Rating Scale Positive Symptom subscale (BPRS+) will be administered to assess treatment-emergent psychotic symptoms;
- Bladder Pain/Interstitial Cystitis Symptom Score (BPIC-SS) to identify symptoms of cystitis;
- Montreal Cognitive Assessment (MoCA) will be administered to assess cognitive function;

Confidential © 2017 Clintec All Rights Reserved

- Evaluations of AEs, concomitant medication, physical examination findings, vital signs (oral body temperature, pulse rate [radial], respiratory rate, blood pressure and blood oxygen saturation), body weight and 12-lead electrocardiogram (ECG) throughout the study.

Managing the risk of overdose:

To manage the risk of overdose during the study, subjects will be evaluated for suicidal ideation before and during the study to minimise potential for overdosing. In addition to site visits, subjects will receive telephone calls and be asked to attend Pharmacy visits to collect the study drug, as specified in Section 3. The additional contact between visits allows the Investigator to encourage compliance and document any AEs the subject may experience outside of the site visits.

On days 36 and 64, subjects will be dispensed 3 doses of study drug to be taken at home as instructed, with one follow up telephone call. On days 50 and 78, subjects will be asked to visit the Pharmacy to receive 4 doses of study drug to be taken at home as instructed, with one follow up telephone call. The following will be the maximum total dosage of study drug dispensed to subjects in each treatment arm, at any one site/ Pharmacy visit:

Treatment arm	Maximum total dosage of study drug dispensed on days 36 and 64 to be taken at home	Maximum total dosage of study drug dispensed on days 50 and 78 to be taken at home
• R-107 30 mg	90 mg	120 mg
• R-107 60 mg	180 mg	240 mg
• R107 120 mg	360 mg	480 mg
• R-107 180 mg	540 mg	720 mg

For more details, see Section 3 for the overall study design, Section 5.3 for dispensing and Section 6.1 for drug administration procedures.

In the event of overdose, subjects will be advised to seek immediate assistance by dialling the relevant local emergency number to access medical care. Symptoms of ketamine overdose include:

- Neurological (impaired consciousness, confusion, drowsiness, dizziness, nystagmus, agitation, hallucinations)
- Urological (dysuria)
- Cardiovascular (hypertension, tachycardia, chest pain, palpitations)
- Gastrointestinal (abdominal pain, nausea, vomiting)
- Respiratory (dyspnoea)
- Psychiatric (acute psychosis).

Subjects will receive clinical care, e.g. in the emergency department of a local hospital until symptoms have resolved. Symptomatic improvement has been reported to occur within 2-6

Confidential © 2017 Clintec All Rights Reserved

hours (Ng *et al.,* 2010). Subjects who have overdosed on study drug will be referred for psychiatric consultation and will be withdrawn from the study.

Managing subjects who demonstrate a significant reduction in MoCA scores:

The MoCA questionnaire is used as a measure of cognitive function and is assessed from screening to end of treatment. At screening, subjects must have a MoCA score of ≥26 to be included in this study. MoCA is further assessed at Day 8, 36, 64, 92, early termination (if applicable) and follow up. Subjects who show a reduction in scores of ≥2 points in the MoCA, together with subjective reporting of cognitive side effects, will be reviewed by the consultant clinical neuropsychologist. This review will involve consultation with the site principal investigator, including review of the subject's cognitive assessments (MoCA, Verbal Fluency and SDMT). Following this review, the subject will be referred for a detailed neuropsychological assessment, locally, if required.

R-107 is reasonably anticipated to provide a meaningful clinical benefit relative to alternative treatments for TRD, such as ECT, in that it does not require hospitalization (consequent to anaesthesia as required for ECT) for treatment to be administered. Furthermore, R-107 appears to be effective when dosed once or twice weekly, demonstrating improved convenience of administration compared with ECT. Study ZPS-603 with R-107 did not indicate changes of clinical significance in vital signs, ECG, safety laboratory tests or urinalyses in any subjects. The only AE to show dose-related increases in frequency was dissociation of mild intensity, in subjects dosed with 240 mg. No participants reported suicidal ideation at any time and there was no evidence of increased arousal or behavioural activation.

This study also incorporates guidelines as per European Union Good Clinical Practice (GCP) (International Conference on Harmonisation of Technical Requirements for Registration of Pharmaceuticals for Human Use; ICH), the United States Code of Federal Regulations on the Protection of Human Subjects (21 CFR Part 50), the Declaration of Helsinki and its amendments, all applicable standard operating procedures and local regulatory requirements.

It can be considered unethical to conduct placebo-controlled studies in MDD due to the potential risk of irreversible harm resulting from lack of treatment. However, the inclusion criteria states subjects must have the indication of interest, MDD. Moreover, subjects must have treatment resistance in major depression (TRD), defined as lack of clinically meaningful improvement despite the use of adequate doses of at least two antidepressant agents, derived from the group(s) of commonly used first line treatment, prescribed for adequate duration with adequate affirmation of treatment adherence[2]. Subjects enrolled will be resistant to standard available treatment and so the use of placebo-control in this study could be thought of as ethical as the subject is not being denied access to a standard used treatment that can effectively treat their depression. This is a key-point when considering the ethical implications of this study design.

[2] Note: 'Lack of clinically meaningful improvement' is defined as failure of treatment to produce response or remission for patients and is determined after detailed clinical interview with the patient. In addition, patients are required to have depression of at least moderate severity, based on a MADRS score of 20 or greater.
'Adequate doses' is defined as the minimum therapeutic dose as per the product label OR maximum tolerated dose.
'Adequate duration' is defined as a minimum duration of 6 weeks.
'Adequate affirmation of treatment adherence' is affirmation sought during routine clinical interview (Fekadu, 2009).

Confidential © 2017 Clintec All Rights Reserved

Adequate precautions will be implemented to ensure subject safety in the study. The safety assessments throughout the study are frequent and extensive. Potential subjects will be fully informed of the risks and requirements of the study and, during the study, subjects will be given any new information that may affect their decision to continue participation. Subjects will be informed that their consent to participate in the study is voluntary and may be withdrawn at any time with no reason given and without penalty or loss of benefits to which they would otherwise be entitled. Subjects may participate in the study only if they have capacity to give consent and after fully understanding the potential risks and benefits and giving written informed consent.

Confidential © 2017 Clintec All Rights Reserved

2 STUDY OBJECTIVES AND ENDPOINTS

2.1 Objectives

2.1.1 Primary Objectives

- To evaluate the efficacy of ER R-107 tablets (30 mg, 60 mg, 120 mg, 180 mg) as measured by the change in MADRS total score from baseline (Day 1) to Day 92 in subjects with Diagnostic and Statistical Manual of Mental Disorders, 5th edition (DSM-5) TRD who have responded to 1 weeks' treatment with R-107 120 mg tablets.

2.1.2 Secondary Objectives

- To evaluate the efficacy of ER R-107 tablets (30 mg, 60 mg, 120 mg, 180 mg) as measured by the change in MADRS total score from baseline (Day 1) to Day 36 and to Day 64 in subjects with TRD who have responded to 1 weeks' treatment with R-107 120 mg tablets;
- To assess the effect of R-107 tablets compared to placebo on response at Day 36, Day 64 and Day 92, where response is defined as ≥50% reduction from baseline (Day 1) in MADRS total score;
- To assess the effect of R-107 tablets compared to placebo on remission at Day 36, Day 64 and Day 92, where remission is defined as MADRS total score ≤10;
- To assess the effect of R-107 tablets compared to placebo on the severity of illness using the Clinician Global Impression – Severity (CGI-S) and the Patient Global Impression – Improvement (PGI-I)
- To evaluate the pharmacokinetics (PK) of R-107 tablets in subjects with TRD.
- To investigate the dose-response pattern for R-107 with respect to the primary efficacy outcome (MADRS) and safety outcomes

2.1.3 Exploratory Objectives

- Subject perspective of global improvement in TRD from baseline as measured by the Patient Global Impression-Improvement (PGI-I);
- Impact on health status as assessed using the EuroQol-5D, 5-level version (EQ-5D-5L) questionnaire;
- Subject perspective on impaired functioning during the double-blind phase of the study using the Work and Social Adjustment Scale (WSAS).
- To assess the effect of R-107 tablets compared to placebo on delaying relapse during the double-blind randomisation phase, where relapse is defined as MADRS total score ≥ 22.

2.1.4 Safety Objectives

Part 1: Enrichment Open-label Phase

Confidential © 2017 Clintec All Rights Reserved

To evaluate the safety, tolerability and Psychiatric Pharmacodynamics (PD) of 120 mg of R-107 tablets in an open-label enrichment treatment phase in a population of 200 subjects meeting inclusion criteria.

- Adverse Events (AEs);
- Effect on heart rate, BP, respiratory rate, temperature and blood oxygen saturation (SpO2);
- Effect on laboratory evaluations and physical examination;

Part 2: Randomised Double-Blind Treatment Phase

To evaluate the safety, tolerability and Psychiatric PDs of R-107 tablets at 30 mg, 60 mg, 120 mg and 180 mg, in a treatment-enriched subject population with ketamine responsive TRD.

- Adverse Events (AEs);
- Effect on heart rate, blood pressure (BP), respiratory rate, temperature and blood oxygen saturation (SpO2);
- Effect on laboratory evaluations and physical examination;
- Effects on suicidal ideation/behaviour measured by the eC-SSRS;
- Psychosis-like side effects by using a four-item positive symptom subscale of the BPRS+ consisting of: suspiciousness, hallucinations, unusual thought content, and conceptual disorganization;
- Effects on dissociative symptoms using the CADSS;
- Potential treatment-emergent symptoms of cystitis using the BPIC-SS;
- Effects on aspects of cognitive functioning (attention and memory), as assessed by the MoCA, Verbal Fluency (category and letter) and Symbol Digit Modalities Test (SDMT).

2.2 Endpoints

2.2.1 Primary Endpoints

- The primary efficacy evaluation for the study will be the change in MADRS total score as measured by the change from baseline (Day 1 pre-dose) to the end of the double-blind randomised phase, planned at Day 92.

2.2.2 Secondary Endpoints

- Change from baseline (Day 1 pre-dose) in MADRS total score to Day 36 and Day 64;
- Proportion of subjects with response at Day 36, Day 64, and Day 92, where response is defined as ≥50% reduction from baseline in MADRS total score;
- Proportion of subjects in remission at Day 36, Day 64, and Day 92, where remission is defined as MADRS total score of ≤10;
- Change from baseline to the final drug administering visit (Day 92) of the CGI-S;
- Change from baseline to the final drug administering visit (Day 92) of the PGI-S;
- PK parameters (CL/F; Vd/F) from the population PK analysis of sparse plasma samples obtained on Days 8-9, 64-65, and 92-93.

2.2.3 Exploratory Endpoints

- PGI-I to assess improvement of disease from the subject's perspective;

Confidential © 2017 Clintec All Rights Reserved

- Change in the EQ-5D-5L (EQ-VAS) score from baseline (Day 1) to the final drug administering visit (Day 92);
- Change from baseline (Day 1) to the final drug administering visit (Day 92) in the WSAS score.
- Proportion of relapsed subjects (MADRS ≥ 22) at Day 36, Day 64 and Day 92;
- Time to relapse (defined as MADRS ≥ 22);

2.2.4 Safety Endpoints

- Reported adverse events;
- Summary of vital signs (pulse rate (radial), systolic and diastolic BP, respiratory rate, oral body temperature, and blood oxygen saturation), laboratory parameters and physical examination;
- Change from baseline in eC-SSRS, CADSS, BPRS+ and BPIC-SS scores at Day 8, Day 36, Day 64 and Day 92;
- Change from baseline to the final drug administering visit (Day 92) in cognitive functioning – MoCA, Verbal Fluency (category and letter) and SDMT scores.

Confidential © 2017 Clintec All Rights Reserved

3 INVESTIGATIONAL PLAN

3.1 Overall Study Design

This is a multi-centre, Phase 2a study, which incorporates an enrichment open-label phase followed by randomised, double-blind, placebo-controlled phase.

Approximately 200 subjects will be enrolled across three countries following screening against the eligibility criteria. Subjects successfully enrolled will participate in up to 4 phases:

- Phase 1: A screening phase of up to 4 weeks (Day -28 to -1);
- Phase 2: An enrichment phase: open-label treatment phase for 1 week (Days 1-7) to identify responders for the enrichment population to continue in the subsequent double-blind treatment phase;
- Phase 3: A double-blind treatment phase for 12 weeks (Days 8-92);
- Phase 4: A 4-week post-treatment (follow-up) phase.

The total study duration for each subject will be 20 weeks with the study end being the completion of the last subject's follow-up visit.

The treatment section of the study will comprise of two parts:

Part 1: Enrichment open-label phase

- Approximately 200 subjects will be screened and selected into the enrichment phase consisting of an open-label treatment phase of 5 doses of R-107 at 120 mg (Days 1-5).

Part 2: Double-blind randomisation treatment

- 150 subjects who have a positive clinical response (≥50% reduction in their MADRS total score which is ≤12) in Part 1 will be randomised into 5 arms within the Part 2 double-blind treatment phase (Days 8-92).

Subjects will be screened from Days -28 to -1 prior to dose administration for enrolment. Evaluations will be performed throughout the study duration as per the Study Schedule. All participating subjects who have had at least one dose of R-107/placebo will be followed up for safety for 4 weeks after their last dose.

Part 1: On Day 1, approximately 200 subjects will receive a single dose of R-107 120 mg (2 x 60 mg) and will complete assessments outlined in the Study Schedule of Part 1 (Table 1). Subjects will return to the study site on Days 2-5 for 4 additional single doses of R-107 120 mg. Subjects will remain in the study centre for 4 hours post-dose on Day 1 and will complete procedures. Subjects will be given the telephone number of the study centre which they will be encouraged to call if they experience any post-dose AEs on the remaining study days.

Subjects will return to the study centre on Day 8 for evaluation of their response to treatment with the open-label R-107 120 mg for 5 days and randomisation if the response criteria is met.

Enrolment to Part 1 of the study will continue until 150 responders enter Part 2 of the study.

Part 2: Following the enrichment open-label phase, 150 subjects with a clinical response as demonstrated by ≥50% reduction of their MADRS total score which is ≤12 will enter into Part

Confidential © 2017 Clintec All Rights Reserved

2 of the study. The subjects will be randomised on Day 8 into double-blind treatment with either placebo, R-107 30 mg, R-107 60 mg, R-107 120 mg or R-107 180 mg (n=30/treatment). A pre-dose blood sample will be obtained on Day 8 prior to study drug administration. Subjects will be dispensed tablets for their corresponding randomised R-107 dose or placebo followed by the completion of the safety and efficacy assessments including blood sample collection to assess PK at 4 and 24-hours post-dose.

Subjects will also be contacted via telephone calls weekly throughout the treatment phase as detailed in Figure 3. The additional contact between visits allows the Investigator to encourage compliance and document any AEs the subject may experience outside of site visits. Subjects will also attend Pharmacy Visits on Day 50 and Day 78 for study drug dosing and dispensing (Figure 3). There is a window of +24 hours for a missed dose in the Part 2 of the study.

All laboratory testing will be performed at local laboratories, with the exception of the PK assessments, which will be tested at a Central Laboratory. Subjects will be followed up for safety at each visit and full complement of study safety parameters, laboratory assessments, procedures, and evaluations will be performed at the primary endpoint conclusion on Day 92 or at a subject's early study termination.

All AEs and special events, whether serious or non-serious, will be reported from the time a signed and dated informed consent form (ICF) is obtained until completion of the subject's last study-related procedure (which may include contact for follow-up of safety).

The duration of the subject's participation will be approximately 20 weeks. The end of study will occur when the last subject in the study completes his/her last study assessment.

Subjects who withdraw or are withdrawn from the study prior to Day 92 may be eligible to enter a separate OLE study, if the reason for withdrawal was a relapse of depressive symptoms during the double-blind treatment phase of the study. Subjects withdrawn because of safety or tolerability issues may also be eligible to enter a separate OLE study, if the issues were unrelated to R-107. The blind will not be broken for subjects withdrawing prematurely.

Confidential © 2017 Clintec All Rights Reserved

3.2 Study Flow Chart and Timelines

Figure 1: Study Timeline

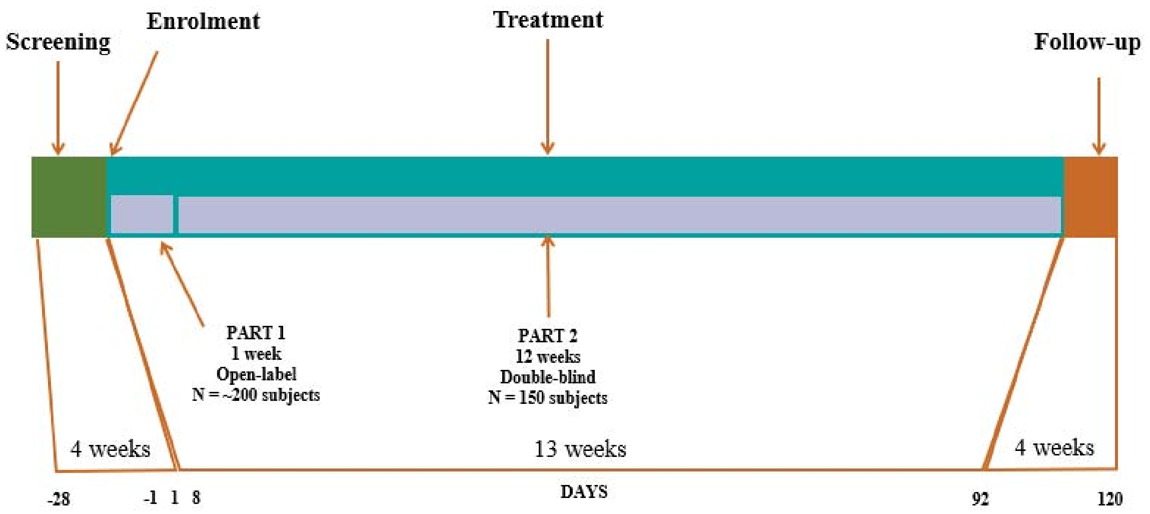

Figure 2: Subject Visit and Dosing Schedule

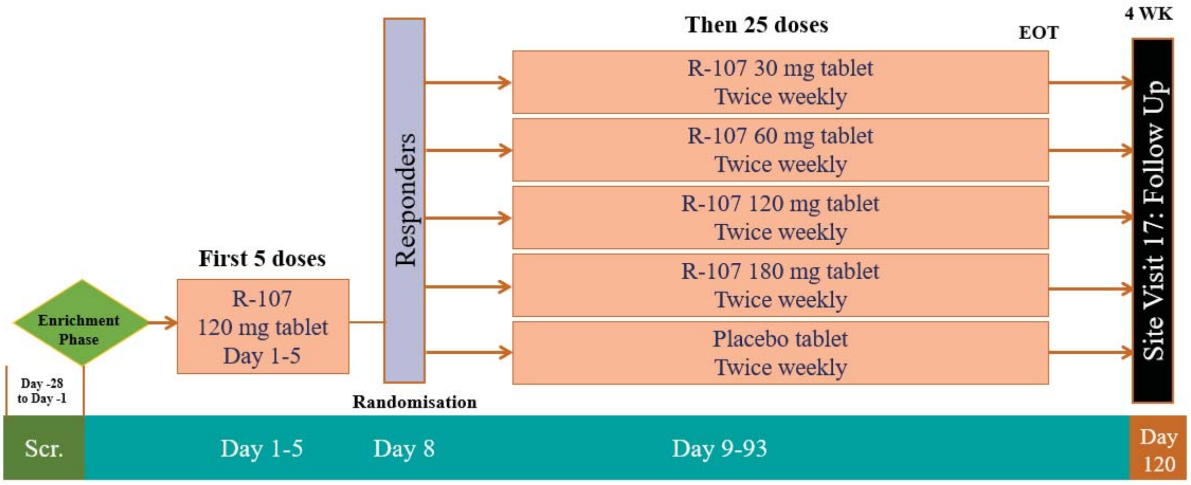

17 Site Visits + 2 Pharmacy Visits

Confidential © 2017 Clintec All Rights Reserved

Figure 3: Subject Visit and Telephone Follow-up Schedule (representative).

Phase	Week No.	Day						
1: Screening	-4 to -1	-28	-27	-26	-25	-24	-23	-22
		-21	-20	-19	-18	-17	-16	-15
		-14	-13	-12	-11	-10	-9	-8
		-1	-2	-3	-4	-5	-6	-7
2: Open Label	1	1	2	3	4	5	6	7
3: Double-blind randomisation phase	2	8	9	10	11	12	13	14
	3	15	16	17	18	19	20	21
	4	22	23	24	25	26	27	28
	5	29	30	31	32	33	34	35
	6	36	37	38	39	40	41	42
	7	43	44	45	46	47	48	49
	8	50	51	52	53	54	55	56
	9	57	58	59	60	61	62	63
	10	64	65	66	67	68	69	70
	11	71	72	73	74	75	76	77
	12	78	79	80	81	82	83	84
	13	85	86	87	88	89	90	91
	14	92	93	94	95	96	97	98
4: Safety Follow-up	15	99	100	101	102	103	104	105
	16	106	107	108	109	110	111	112
	17	113	114	115	116	117	118	119
	18	120						

Key

Onsite visit
Telephone calls
Pharmacy Visit

Confidential © 2017 Clintec All Rights Reserved

Table 1: Study Schedule – Part 1 (Enrichment Open-Label R-107 120 mg QD x 5D)

	Screening	Treatment		Early Termination (a)
		Baseline	Dosing	
	Day -28 to -1	Day 1	Day 2-5	
Informed consent	X			
Inclusion/exclusion criteria	X	X		
Demographics and medical history	X			
Medication history	X			
Physical examination	X			
Vital signs (b)	X	X	X	X
Weight, height (c)	X	X		X
Concomitant medications (d)	X	X	X	X
Concurrent medical conditions	X	X		
Clinical laboratory tests (e)	X	X		X
HIV/Hepatitis panel	X			
Serum Pregnancy test (f)	X			
Urine Pregnancy test (f)		X		
FSH (g)	X			
Urine drug screen/Alcohol breath test	X	X		
Safety ECG (h)	X	X		X
MINI	X			
MSM	X			
MoCA (i)	X			X
MADRS	X	X[m]	X[o]	X
CADSS	X	X[n]	X	X
eC-SSRS (j)	X	X[m]		X
BPIC-SS	X	X[m]		X
PGI-I	X	X[m]		X
PGI-S	X	X[m]		X
CGI-S	X	X[m]		X
WSAS	X	X[m]		X
EQ-5D-5L	X	X[m]		X
BPRS+	X	X[n]		X
Verbal Fluency		X[m]		X
SDMT		X[m]		X
Study Drug Dosing		X	X	
PTE assessment (k)	X	X		
AE assessment (l)		X	X	X

(a) Conduct procedures for subjects discontinued early.
(b) Vital signs (oral temperature, pulse rate [radial], respiratory rate, blood oxygen saturation and BP) will be obtained at screening, during each treatment period prior to and 30 minutes after dosing, and Early Termination, as appropriate.
(c) Height collected only at screening.
(d) Record all ongoing medications and non-pharmacologic therapies.
(e) Clinical laboratory tests (haematology, serum chemistry, urinalysis) will be collected at screening, Day 1 and 8, and Early Termination, as appropriate.
(f) For subjects of childbearing potential only.
(g) An FSH level will be obtained for post-menopausal subjects (defined as continuous amenorrhea >12 months and not surgically sterile).
(h) Safety ECG will be measured by standard stationary 12-lead ECG machines. ECG will be recorded at screening, Day 1 (pre-dose [within 30 minutes prior to dosing], and at Day 8, and Early Termination, as appropriate.
(i) The administration sequence of the MoCA assessment will be from Version 8.1 to Version 8.2 to Version 8.3, back to Version 8.1 and so on. The version of the MoCA assessment used at the specified study visit should differ from and will depend on the last version of MoCA administered to the subject.
(j) eC-SSRS screening version to be used at screening and "since last visit" version for all others.
(k) Pre-treatment events (PTEs) will be collected from signing of informed consent until dosing on Day 1 of the first treatment period.
(l) Any event after dosing on Day 1 of the first treatment period will be captured as an AE.

Confidential © 2017 Clintec All Rights Reserved

(m) Questionnaires will be completed pre-dose on Day 1 visit to ensure a valid baseline measurement prior to any drug dosing.
(n) CADSS and BPRS+ assessments will be performed pre-dose and 2 hours after dosing
(o) MADRS to be completed on Day 5 only

Confidential © 2017 Clintec All Rights Reserved

Table 2: Study Schedule – Part 2 (Randomisation, double-blind treatments)

	Randomisation	Treatment							Early Termination (a)	Safety Follow-up (g) (±5)
		Dosing					Check-out			
	Day 8	Day 9	Day 15, 22, 29	Day 36	Day 64 (±5)	Day 65	Day 92 (±5)	Day 93		
Physical examination	X		X	X	X		X		X	X
Vital signs (b)	X		X	X	X		X		X	X
Weight			X	X	X		X		X	X
Concomitant medications (c)	X		X	X	X		X		X	X
Clinical laboratory tests (d)	X		X	X	X		X		X	X
Urine Pregnancy test (e)					X		X		X	X
Safety ECG (f)	X			X	X		X		X	X
MoCA (k)	X			X	X		X		X	X
MADRS	X		X	X	X		X		X	X
CADSS	X		X	X	X		X		X	X
eC-SSRS	X		X	X	X		X		X	X
BPIC-SS	X		X	X	X		X		X	X
PGI-I	X		X	X	X		X		X	X
PGI-S	X		X	X	X		X		X	X
CGI-S	X		X	X	X		X		X	X
WSAS	X		X	X	X		X		X	X
EQ-5D-5L	X		X	X	X		X		X	X
BPRS+	X		X	X	X		X		X	X
Verbal Fluency	X		X	X	X		X		X	X
SDMT	X		X	X	X		X		X	X
Study Drug Dosing and Dispensing (j)	X		X	X	X		X			
AE assessment	X		X	X	X		X		X	X
PK sample collection	X[i]	X[h]			X[i]	X[h]	X[i]	X[h]		

(a) Conduct procedures for subjects discontinued early.
(b) Vital signs (oral temperature, pulse rate [radial], respiratory rate, blood oxygen saturation, and BP) will be obtained at Enrolment, during each treatment period prior to and 30 minutes after dosing, and Early Termination, as appropriate.
(c) Record all ongoing medications including non-pharmacologic therapies.
(d) Clinical laboratory tests (haematology, serum chemistry, urinalysis) will be collected at Enrolment, during each treatment period, and Early Termination, as appropriate.
(e) For subjects of childbearing potential only.
(f) Safety ECG will be measured by standard stationary 12-lead ECG machines pre-dose on Days 8, 36, 64, 92, at follow-up safety visit and Early Termination. ECG will also be measured 4 hours post-dose on Days 8, 64 and 92. This will be completed in conjunction with PK sample collections.
(g) A safety follow-up visit to be conducted 28 (±5) days after the subject's last dose of study drug.
(h) PK 24hr sample collection.
(i) PK plasma samples to be collected pre-dose and 4hrs post-dose.
(j) Reconciliation of drugs at each visit. There is a window of +24 hours for a missed dose in the Part 2. Subjects will be asked to return used and unused bottles of drugs given at prior visit to ensure subject compliance. Subjects will have scheduled Pharmacy Visits on Day 50 and Day 78 for study drug dosing and dispensing.
(k) The administration sequence of the MoCA assessment will be from Version 8.1 to Version 8.2 to Version 8.3, back to Version 8.1 and so on. The version of the MoCA assessment used at the specified study visit should differ from and will depend on the last version of MoCA administered to the subject.

Confidential © 2017 Clintec All Rights Reserved

4 STUDY POPULATION

4.1 Subject Selection

Approximately 200 subjects who meet the eligibility criteria will be enrolled through the screening phase of up to 28 days. Following the enrichment, open-label phase, 150 subjects who respond to treatment will be taken forward to be randomised on Day 8 into one of 5 treatment arms for the randomised double-blind treatment phase.

Approval to re-screen will be granted on a case by case basis by the Medical Monitor and the global study team. The request and rationale to re-screen should be documented. There will be no waivers granted for eligibility criteria at Day 1 of the Enrichment phase. Subjects not meeting the MADRS criteria at Day 8 (≥50% reduction of their MADRS total score which is ≤12) will not be re-screened.

The initial recruitment approach will be carried out by a registered nurse who will assist the Investigator in obtaining written informed consent and checking inclusion and exclusion criteria.

4.2 Inclusion Criteria

Subjects must meet all the following criteria:

1. Provision of written informed consent prior to any study specific procedures;
2. Subjects aged 18-80 years inclusive at the time of enrolment (screening visit);
3. Diagnosed with MDD as per DSM-5 and MINI 7.0.2 without psychotic features for at least three months prior to screening (comorbid anxiety disorders are also acceptable);
4. Montgomery Asberg Depression Rating Scale (MADRS) total score of ≥20 at screening and baseline;
5. Treatment resistance in major depression (TRD) defined as lack of clinically meaningful improvement despite the use of adequate doses of at least two antidepressant agents, derived from the group(s) of commonly used first line treatment, prescribed for adequate duration with adequate affirmation of treatment adherence[3];
6. Montreal Cognitive Assessment (MoCA) score ≥26 assessing cognitive function;
7. Psychotropic medication and/or psychotherapy is stable (i.e. no change of drugs or drug dose or visit schedule within 28 days prior to Day 1);
8. Subjects must weigh at least 50kg and have a Body Mass Index (BMI) between 18 and 40 kg/m^2 inclusive;

[3] Note: 'Lack of clinically meaningful improvement' is defined as failure of treatment to produce response or remission for patients and is determined after detailed clinical interview with the patient. In addition, patients are required to have depression of at least moderate severity, based on a MADRS score of 20 or greater.
'Adequate doses' is defined as the minimum therapeutic dose as per the product label OR maximum tolerated dose.
'Adequate duration' is defined as a minimum duration of 6 weeks.
'Adequate affirmation of treatment adherence' is affirmation sought during routine clinical interview (Fekadu, 2009).

Confidential © 2017 Clintec All Rights Reserved

9. Subjects of childbearing potential (SOCBP) must use a highly effective form of birth control (confirmed by the Investigator). Highly effective forms of birth control include:
   - True sexual abstinence (defined as refraining from heterosexual intercourse for the duration of the study and a minimum of 30 days following the last dose of study drug);
   - Oral, intravaginal, or transdermal combined (oestrogen and progestogen containing) hormonal contraception associated with inhibition of ovulation;
   - Oral, injectable, or implantable progestogen-only hormonal contraception associated with inhibition of ovulation;
   - Intrauterine device (IUD);
   - Intrauterine hormone-releasing system (IUS);
   - Bilateral tubal occlusion;
   - Vasectomised partner (provided that the partner is the sole sexual partner of the participant with childbearing potential and that the vasectomised partner has received medical assessment of the surgical success).

   SOCBP must agree to use highly effective method of birth control, as defined above, from enrolment, throughout the study duration and within 30 days after last dose of Investigational Medicinal Product (IMP) and must have negative serum pregnancy test result at Visit 1.

   SOCBP are defined as subjects with a uterus who are NOT either permanently sterilized (hysterectomy, bilateral oophorectomy, or bilateral salpingectomy), or who are NOT postmenopausal. Subjects will be considered post-menopausal if they have been amenorrhoeic for 12 months prior to the planned date of randomisation without an alternative medical cause.

   The following age-specific requirements apply:
   - <50 years old would be considered postmenopausal if they have been amenorrhoeic for 12 months or more following cessation of exogenous hormonal treatment and follicle stimulating hormone (FSH) levels in the postmenopausal range (FSH level >40 mIU/mL);
   - ≥50 years old would be considered postmenopausal if they have been amenorrhoeic for 12 months or more following cessation of all exogenous hormonal treatment.

   Rhythm methods will not be considered as highly effective methods of birth control.

10. Subjects with the ability of producing sperm must use an adequate method of contraception (condom or condom with spermicide depending on local regulations) from the first dose of IMP until 30 days after their last dose. Subjects with a partner or partners who is (are) not of childbearing potential are exempt of these requirements;
11. Subjects with the ability of producing sperm must not donate sperm for at least 30 days post-dose of the last study treatment;

Confidential © 2017 Clintec All Rights Reserved

12. A subject with the ability of producing eggs (ova, oocytes) must agree not to donate eggs for the purposes of assisted reproduction during the study and for at least 3 months after receiving the last dose of study drug;
13. Ability and willingness to attend the necessary visits to the study centre;
14. Ability to read and write;
15. Subject must be willing and able to adhere to the prohibitions and restrictions specified in the protocol;
16. Able to swallow tablets.

Entry to Double-blind phase of the study at Day 8

1. Subjects with a clinical response as demonstrated by ≥50% reduction of their MADRS total score which is ≤12 will enter into Part 2 of the study.

4.3 Exclusion Criteria

Subjects must NOT meet any of the following exclusion criteria to be enrolled:

1. Any significant disease or disorder (e.g., cardiovascular, pulmonary, gastrointestinal, hepatic, renal, neurological, musculoskeletal, endocrine, metabolic, malignant, psychiatric, major physical impairment) which, in the opinion of the Investigator, may either put the subject at risk because of participation in the study, or may influence the results of the study, or the subject's ability to participate in the study;
2. Contraindication to the use of R-107, e.g., any condition in which a significant elevation of BP would be hazardous, such as decompensated heart failure, severe or poorly controlled hypertension (BP systolic ≥160 or diastolic ≥90, tested on 2 or more occasions); within last 3 months, recent myocardial infarction, stroke, cerebral haemorrhage; myasthenia gravis; known allergy, hypersensitivity, or intolerance to ketamine or its excipients;
3. History of neurodegenerative disorder e.g. Alzheimer's disease, vascular dementia, Parkinson's disease or evidence of mild cognitive impairment;
4. Any clinically significant abnormal findings in physical examination, vital signs, haematology, clinical chemistry, or urinalysis during screening and at baseline, which in the opinion of the Investigator, may put the subject at risk because of his/her participation in the study, or may influence the results of the study, or the subject's ability to complete entire duration of the study;
5. Any ECG abnormality obtained during the screening period that in the Investigator's judgement may put the subject at risk or negatively affect the outcome of the study;
6. Subjects are excluded if they have any of the following:
    - A history of known immunodeficiency disorder including a positive test for human immunodeficiency virus, HIV-1 or HIV-2;

Confidential © 2017 Clintec All Rights Reserved

- Positive hepatitis B surface antigen, or positive hepatitis C virus antibody serology, or a positive medical history for hepatitis B or C. Subjects with a history of hepatitis B vaccination without history of hepatitis B are allowed to enrol.

7. Hepatic Insufficiency: Alanine aminotransferase (ALT) or aspartate aminotransferase (AST) level ≥2 times the upper limit of normal (ULN) confirmed by repeated testing during screening period;
8. Pregnant, breastfeeding, or lactating subjects (subjects of childbearing potential must have a negative serum pregnancy test at screening and a negative urine pregnancy test on Day 1);
9. Significant current risk of suicide:
   - As assessed by eC-SSRS (baseline), or
   - Serious risk for suicide, as assessed by the evaluating study clinician; a serious suicide risk will be considered:
     (a) an inability to control suicide impulses or imminent or unacceptably high risk of suicide in the Investigator's judgment; or
     (b) a recent history of suicidal behaviour, which is defined as either one or more suicide attempts (or interrupted suicide attempts) in the 12 months before study entry; or
     (c) history of serious suicidal behaviour, defined as one or more suicide attempts (or interrupted attempts) in the last 3 years with a potential lethality judged by the evaluating study clinician to have possibly resulted in serious injury or death.
10. History of alcohol or drug abuse within the past year, which may compromise the study data interpretation as judged by Investigator or Study Physician;
11. Subject has a positive test result(s) for drugs of abuse (including barbiturates, methadone, opiates, cocaine, phencyclidine, and amphetamine/methamphetamine) at screening or pre-dose on Day 1. In addition to the drugs of abuse previously mentioned, cannabinoids will also be tested on Day 1.
    - Subjects that have a positive test result at screening due to prescribed opiates may be permitted to continue the screening phase if the prohibited medication is discontinued at least 1 week or 5 half-lives, whichever is longer, before the first dose of study medication. Provided the Day 1 pre-dose test for drugs of abuse result is negative, the subject may be enrolled. Retesting is not permitted for positive test result(s) from non-prescription use of drugs of abuse.
    - Subjects who have a positive test result at screening due to prescribed psychostimulants (e.g., amphetamine, methylphenidate, etc.) taken for an indication other than MDD are permitted to continue to take this medication during the study.
    - A positive test result for cannabinoids pre-dose on Day 1 is exclusionary.

Confidential © 2017 Clintec All Rights Reserved

12. Any clinically significant infection or febrile illness in the five days prior to dosing Day 1;
13. Past or current history of schizophrenia, bipolar disorder, ongoing severe personality disorder, ongoing post-traumatic stress disorder, intellectual disability or severe obsessive-compulsive disorder;
14. History of abuse of ketamine or phencyclidine;
15. Electroconvulsive therapy, transcranial magnetic stimulation, vagal nerve stimulation, deep brain stimulation or other brain stimulation treatment within the past 4 weeks or currently used as either an acute or maintenance treatment of depression;
16. Receipt of any investigational product within 30 days or 5 half-lives prior to dosing; whichever is longer;
17. Subjects should not consume grapefruit, grapefruit juice or Seville oranges for 72 hours before R-107 administration and during the study
18. Subject has a history of malignancy within 5 years before screening (exceptions are squamous and basal cell carcinomas of the skin and carcinoma in situ of the cervix, or malignancy that in the opinion of the Investigator, with concurrence with the Sponsor's medical monitor, is considered cured with minimal risk of recurrence);
19. Subject has received any disallowed therapies as noted in the protocol (Table 7), Pre-study and Concomitant Therapy before the specific time relative to the planned first dose of study drug;
20. Subject has had major surgery, (e.g., requiring general anaesthesia) within 2 weeks before screening, or will not have fully recovered from surgery, or has surgery planned during the time the subject is expected to participate in the study. Note: subjects with planned surgical procedures to be conducted under local anaesthesia may participate;
21. Employees of the clinical study centre or family members (first-degree relatives) of such individuals or anyone involved in the planning and/or conduct of the study;
22. Subjects who do not consent to their General Practitioner being contacted prior to the commencement of the study, if necessary, about their medical history or after the study about any adverse results or reactions;
23. Subjects who, in the opinion of the Investigator, do not understand the information and procedures of the study, or would not be compliant with them (in particular the study restrictions and risks involved).

Investigators should ensure that all study enrolment criteria have been met at screening. If a subject's status changes (including laboratory results or receipt of additional medical records) after screening but before the first dose of study drug is given such that they no longer meet all eligibility criteria, then the subject should be excluded from participation in the study.

Confidential © 2017 Clintec All Rights Reserved

4.4 Discontinuation of Study Medication

In the event of discontinuation of a subject on the study medication, the Investigator should assess the cause for discontinuation. As far as possible, subjects who have discontinued study medication should still be assessed for safety and primary efficacy as per the Study Schedule.

Reasons that a subject may discontinue study medication include but are not limited to:

- The Investigator or Sponsor believes (e.g., that for safety or tolerability reasons such as an AE) it is in the best interest of the subject to discontinue the study medication;
- A subject takes a break in study medication and returns to the normal schedule after the break;
- If a subject misses more than two consecutive doses of study medication during the double-blind treatment phase or has <80% compliance based on counts of returned medication, they will not be included in the per-protocol efficacy analysis but will be included in safety outcomes and so will continue to be assessed.

Subjects who discontinue study medication should be encouraged to continue to attend site visits to be assessed so their data can be used in safety reporting.

4.5 Withdrawal of Subjects

In the event of withdrawal of a subject, the Investigator should assess the primary cause for the subject's withdrawal and document this in the Case Report Form (CRF). As far as possible all subjects should undergo all assessments.

Subjects will be informed that they have the right to withdraw from the study at any time, without prejudice to their medical care, and that they are not obliged to state their reasons. Any withdrawal must be fully documented in the CRF and source documents.

A subject will be withdrawn from the study for any of the following reasons:

- Lost to follow-up;
- Withdrawal of consent;
- Violation of protocol procedures (determined on a case by case basis);
- The subject becomes pregnant.

If a subject is lost to follow-up, every reasonable effort must be made by the study site personnel to contact the subject and determine the reason for discontinuation/withdrawal. The measures taken to follow-up must be documented.

Subjects showing clinically significant worsening of symptoms (as measured through change in MADRS, CADSS, eC-SSRS, BPIC-SS score) will be immediately withdrawn from the study if:

- MADRS score of ≥22

  OR

- The Principal Investigator sees fit that a subject is withdrawn because of significant tolerability and/or safety issues.

Confidential © 2017 Clintec All Rights Reserved

Subjects who are withdrawn from the study should consult their own treating physician about receiving further clinical care. If appropriate, the study investigator may provide an opinion to the treating physician on further treatment options.

Subjects who withdraw or are withdrawn from the study prior to Day 92 may be eligible to enter a separate OLE study, if the reason for withdrawal was a relapse of depressive symptoms during the double-blind treatment phase of the study. Subjects withdrawn because of safety or tolerability issues may also be eligible to enter a separate OLE study, if the issues were unrelated to R-107. The blind will not be broken for subjects withdrawing prematurely.

4.6 Ethical and Regulatory Considerations

4.6.1 Study-Specific Design Considerations

Justification for enrichment phase: The study includes an enrichment design element to screen for subjects who respond to the drug R-107, and these subjects go forward to the randomised double-blind treatment phase. The enrichment phase is an open-label treatment of 5 doses of R-107 120 mg over 5 consecutive days. The use of an open-label phase without a control group raises ethical concerns which have been considered in the design of this study. Any AEs with a start date during the open-label treatment phase could be difficult to interpret without a randomised placebo-control group to compare to. Enrichment has been incorporated into the design of this study to select a population who are more likely to benefit from treatment. The investigational drug is well studied and so there is a wealth of literature surrounding its clinical use. For this reason, the absence of a placebo arm in the open-label enrichment phase is justified to reduce the subject population required to enter the screening phase.

Justification for using placebo: Assessment of the potential efficacy of a new compound for the treatment of major depression requires adequate and well-controlled clinical studies. For a new compound, this can be achieved either through a placebo-controlled study or through a study comparing it to current standard treatments through a non-inferiority design. To compare efficacy to standard of care treatment, previous placebo-controlled studies must show the standard of care drug to be superior to placebo. Therefore, the use of a placebo-controlled study is the gold standard for assessment of efficacy of investigational products to allow for scientifically meaningful results.

It can be considered unethical to perform placebo-controlled studies in major depression due to the potential risk of irreversible harm resulting from lack of treatment. Subjects to be recruited for this study must have treatment resistance in major depression (TRD), defined as lack of clinically meaningful improvement despite the use of adequate doses of at least two antidepressant agents, derived from the group(s) of commonly used first line treatment, prescribed for adequate duration with adequate affirmation of treatment adherence[4]. Subjects enrolled will be resistant to standard available treatment and so the use of placebo-control in

[4] Note: 'Lack of clinically meaningful improvement' is defined as failure of treatment to produce response or remission for patients and is determined after detailed clinical interview with the patient. In addition, patients are required to have depression of at least moderate severity, based on a MADRS score of 20 or greater.
'Adequate doses' is defined as the minimum therapeutic dose as per the product label OR maximum tolerated dose.
'Adequate duration' is defined as a minimum duration of 6 weeks.
'Adequate affirmation of treatment adherence' is affirmation sought during routine clinical interview (Fekadu, 2009).

Confidential © 2017 Clintec All Rights Reserved

this study could be thought of as ethical as the subject is not being denied access to a standard treatment that can effectively treat their depression. This is a key-point when considering the ethical implications of this study design. The ketamine/placebo is an adjunct treatment, therefore even on placebo arm, the patients will still be receiving the same drug regime they were on prior to starting the study, including their regular treatment for MDD, prescribed by their own treating doctor. Subjects enrolled will be under close, proactive monitoring from the study team as well as their own clinical care team responsible for their usual care. Each subject's standard care will remain the same, i.e. what is judged to be clinically optimised by their own doctor (except any treatments specified as prohibited in the protocol [see Section 6.2]), prior to study entry.

This study is likely to provide information on the dose-response for R-107, which will guide dosing in subsequent trials. The use of a placebo control is essential to be able to evaluate dose-response. Finally, if patients' depressive symptoms return while being randomized to placebo, they will be eligible to enrol in a subsequent open-label extension protocol with continued access to R-107.

4.6.2 Declaration of Helsinki and ICH-GCP

The study will be conducted according to the protocol and to SOPs that meet the guidelines laid down by the International Conference on Harmonisation (ICH) for Good Clinical Practice (GCP) in clinical trials and in compliance with all relevant guidelines and Ethics Committee/Regulatory approval requirements in each participating country.

The Investigator must inform and obtain approval from the Ethics Committee (EC) for the conduct of the study at sites, the protocol, written subject information and ICF, and for any other written information that will be provided to the subjects and any advertisements that will be used. Written approval must be obtained from the EC prior to recruitment of subjects into the study. If regulatory approval is also required from higher authorities (local, state, or national level) this approval must also be obtained for all relevant material prior to recruitment into the study.

4.6.3 Protocol Amendments

Amendments to the protocol and aforementioned documents must be submitted to the Sponsor for review and approval, and then to the EC and any other regulatory authority as necessary. Amendments may be implemented only after a copy of the EC approval letter has been transmitted to the Sponsor. Amendments that are intended to eliminate an apparent immediate hazard to subjects may be implemented prior to receiving Sponsor or EC approval. However, in this case, approval must be obtained as soon as possible after implementation. Also, in that event, the Investigator must notify the EC and regulatory agency in line with the regulatory requirements in each participating country.

4.7 Protocol Deviations

A protocol deviation is any change, divergence, or departure from the study design or procedures defined in the protocol and can be classed as below:

- Major: may impact the safety and health, well-being of subjects; or the validity of the clinical study data;

Confidential © 2017 Clintec All Rights Reserved

- Minor: all other deviations from the approved protocol version (valid at that time for that particular subject) deriving from non-compliance with the study protocol which are not categorized as major.

All deviations from the approved protocol should be documented. The following details will be captured:

- Site and subject number
- Description of deviation
- Date of deviation
- Date reported and by whom
- Category – Major or minor
- Action
- Status
- Notification to EC

In case of a deviation, the Investigator will inform Clintec and Douglas Pharmaceuticals Ltd. and seek approval for continuation/discontinuation of the concerned subject. The Investigator should not deviate from the protocol. Clintec will not assume any resulting responsibility or liability from unapproved deviations. The EC will be informed of protocol deviations by the Investigator, according to applicable regulations and the EC's established procedures. The EC must provide written agreement for any deviation prior to continuing with the study. In medical emergencies, prior approval for protocol deviations will not be required. Unless Clintec has consented to any such deviations in writing, Clintec will not assume any resulting responsibility or liability. All protocol deviations will be assessed prior to unblinding the study.

4.8 Informed Consent

The principles of informed consent in the Declaration of Helsinki, should be implemented in each clinical study before protocol-specified procedures or interventions are carried out. Information should be given in written form. Subjects, their relatives, guardians, if necessary legal representatives must be given ample opportunity to discuss any details of the study with trained local personnel.

ICFs will be, in a language fully comprehensible to the prospective subjects and be completed by the subject with assistance if requested from trained local personnel. Informed consent shall be documented by the use of a written consent form approved by the EC and signed by the subject.

None of the oral and written information concerning the study, including the written ICF should contain any language that causes the subject or the subject's legally acceptable representative to waive or to appear to waive any legal rights, or that releases or appears to release the Investigator, the institution, the Sponsor, or their agents from liability for negligence.

The written consent document will embody the elements of informed consent as described in the Declaration of Helsinki and will also comply with local regulations. This form may be read to the subject or the subject's legally authorised representative, but, in any event, the

Confidential © 2017 Clintec All Rights Reserved

Investigator shall give either the subject or the representative adequate opportunity to thoroughly comprehend the consent document before it is signed.

The dated signature of the subject must document consent. The signature confirms the consent is based on information that has been understood. The Investigator, for possible inspection by regulatory authorities and/or Clintec and regulatory compliance, must keep each subject's signed ICF on file. A copy of the ICF along with the subject information sheet (SIS) will be given to the subject (and the original retained in the records of the Investigator).

This task is to be performed prior to any other study procedure and is the duty of the Investigator. The Investigator will ensure that the subject has been given enough information, both written and oral, about the nature, possible risks, benefits and procedures that the study will entail, in a language that the subject can understand. They will be informed that participation is purely voluntary and withdrawal at any time is possible and that non-participation in the study will in no way affect their medical treatment. Ample time will be given for questions, discussion, and consideration.

4.9 Subject Information

All subjects will be provided a copy of the SIS that details the study procedures, the risk and benefits of the study, emergency contact numbers, procedures to be followed, voluntary participation and withdrawal, etc. The SIS will be provided in a language fully comprehensible to the prospective subject. It is the responsibility of the Investigator (or a person designated by the Investigator when accepted by local regulations) to give each individual full and adequate verbal and written information regarding the objective, aims, methods and procedures of the study and the anticipated benefits, possible risks and potential hazards involved. Subjects must be informed about the right to withdraw from the study at any time.

Confidential © 2017 Clintec All Rights Reserved

5 DESCRIPTION OF STUDY MEDICATION

5.1 Characterisation of Study Medication

Ketamine (as hydrochloride) (CAS number 1867-66-9) is a non-barbiturate anaesthetic chemically designated dl-2-(o-chloro-phenyl)-2-(methylamino) cyclohexanone hydrochloride.

Figure 4: Structural Formula of Ketamine.

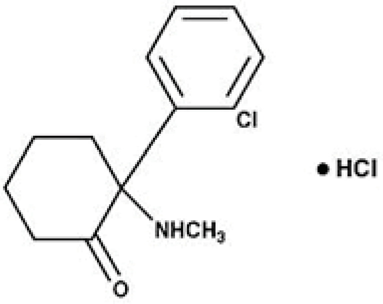

Ketamine contains a chiral centre at the C-2 carbon of the cyclohexanone ring, so that two enantiomers exist S- (+)-ketamine and R-(-)-ketamine. The S- enantiomer is more active pharmacologically in terms of analgesic effects; however, it is unclear if this is applicable to antidepressant effects (Yang, 2015).

Ketamine is a racemic mixture. Ketamine is freely soluble in water and methyl alcohol and is soluble in alcohol (Product Datasheet, 2010).

The study drug will be produced in 30 mg and 60 mg tablets and multiples of these tablets will be taken by subjects depending on which treatment arm they are randomised to.

Formulations:

Table 3: Study Placebo for Ketamine 60 mg Extended Release Tablet Composition:

**Raw Material**	**Content per Tablet**
Polyethylene Oxide	~96%
Magnesium Stearate	~1%
Opadry white Y-1-7000*	~3%
Total Theoretical Tablet Mass	412 mg / 100%

*Contains Hypromellose; Titanium Dioxide (E171); Macrogol 400

Table 4: Study Drug (Ketamine 30 mg Extended Release Tablet) Composition:

**Raw Material**	**Content per Tablet**
Ketamine Hydrochloride	34.6 mg / ~8.5%
Polyethylene Oxide	~87.5%
Magnesium Stearate	~1%
Opadry white Y-1-7000*	~3%
Total Theoretical Tablet Mass	412 mg / 100%

*Contains Hypromellose; Titanium Dioxide (E171); Macrogol 400

Confidential © 2017 Clintec All Rights Reserved

Table 5: Study Drug (Ketamine 60 mg Extended Release Tablet) Composition:

**Raw Material**	**Content per Tablet**
Ketamine Hydrochloride	69.2 mg / ~17%
Polyethylene Oxide	~79%
Magnesium Stearate	~1%
Opadry white Y-1-7000*	~3%
Total Theoretical Tablet Mass	412 mg / 100%

*Contains Hypromellose; Titanium Dioxide (E171); Macrogol 400

The treatment arms of this study are 30 mg, 60 mg, 120 mg, and 180 mg Ketamine tablets and placebo. The study drug will be formulated as a 30 mg tablet and a 60 mg tablet which are identical and identical to the placebo tablet. All subjects will take the same number of tablets regardless to what treatment arm they have been allocated. By treating all subjects with the same number of tablets, the blinding of the study remains valid.

5.2 Packaging, Labelling and Storage

Study drug and placebo will be packaged in white medication bottles. Each bottle will be labelled with a code that is generated by the interactive web response system (IWRS) system to ensure blinding.

5.3 Dispensing

During the Enrichment open-label phase of the study each subject will be assigned with one medication bottle consisting of 10 x 60 mg R-107 tablets. During the visits in the open-label phase each subject will be administered two tablets - 2 x 60 mg R-107.

In the randomised double-blind treatment phase each subject will be provided with bottles containing 3 tablets, identical in appearance, to be taken per dose. The placebo, 30 mg and 60 mg R-107 tablets will all look identical to ensure the study remains blinded.

At each visit, subjects will be provided with the correct number of bottles required to last the correct dose of twice weekly until their next visit, where they will again be provided the study drug. There is a window of +24 hours for a missed dose in the Part 2 of the study, however the subject should not consume the two weekly doses on consecutive days.

<u>Visit Days 1-5:</u>

All subjects enrolled will receive the same dose of 120 mg each day during the enrichment open-label phase.

- Medication will be packed in a white bottle (10 x 60 mg R-107), 2 tablets to be taken per dose, consisting of 2 x 60 mg R-107 tablets.

<u>Visit Days 8, 15, 22, 29, 36 and 64:</u>

The enriched population of 150 subjects, who demonstrated ≥50% reduction in MADRS which is ≤12, from Day 1 to Day 8, will be randomised into one of 5 treatment arms. At visits on Day 8, 15, 22 and 29 each subject will be given two bottles of tablets. The first of the two doses will be taken at the site during the visit. The second dose will be taken by the subject at home.

Confidential © 2017 Clintec All Rights Reserved

At visits on Day 36 and 64 each subject will be dispensed 4 bottles with 3 tablets in each bottle. The first dose will be taken at the site and 3 doses will be taken home to account for 2 weeks' medication.

Pharmacy Visit Days 50 and 78:

On Day 50 and 78, subjects will attend Pharmacy Visits and be dispensed 4 bottles with 3 tablets in each bottle to account for 2 weeks' medication.

Medication will be packed in a bottle, with 3 identical looking tablets to be taken per dose, twice weekly on Days 1 and 4 of the 7-day week. There is a window of +24 hours for a missed dose in the Part 2 of the study, however the subject should not consume the two weekly doses on consecutive days.:

- Placebo: 3 x placebo tablets
- 30 mg: 1 x 30 mg and 2 x placebo tablets
- 60 mg: 1 x 60 mg and 2 x placebo tablets
- 120 mg: 2 x 60 mg and 1 x placebo tablets
- 180 mg: 3 x 60 mg tablets

Visit Day 92:

Each subject will be given one bottle with 3 tablets at this visit. This dose will be taken on site followed by assessments.

Table 6: Study Medication Supplies

Day	Dispensed
Day 1	Dispensed 1 bottle containing 10 tablets (2/day over 5 days)
Days 8, 15, 22, 29	Dispensed 2 bottles of 3 tablets at each visit – 1 dose to be taken in clinic and 1 dose to be taken home for the midweek dose.
Day 36, 64	Dispensed 4 bottles of 3 tablets at each visit – 1 dose to be taken in clinic and 3 doses to be taken home for dosing between clinic/Pharmacy visits.
Day 50, 78	Dispensed 4 bottles of 3 tablets at each Pharmacy Visit – 4 doses to be taken home for dosing between clinic/Pharmacy visits.
Day 92	Dispensed 1 bottle of 3 tablets

5.4 Drug Accountability

Relevant forms will be provided to the Investigator and the pharmacist to maintain accurate written records of all treatment stock from the Sponsor, dispensed to subjects and returned. At the end of the study the drug will be returned to Douglas Pharmaceuticals Ltd. Reconciliation performed of delivery records.

Confidential © 2017 Clintec All Rights Reserved

6 STUDY DRUG TREATMENT

6.1 Drug Administration

Study drug will be administered to each subject at the visits outlined in the Study Schedule (Figure 3, Table 1 and Table 2). Each subject will take the required dose at the site/ Pharmacy visit and will be followed up with assessments at that visit pre- and post-dose administration. In the first 4 weeks of treatment, each subject will visit the site weekly for drug administration and assessments. At these visits the subject will be provided with the study drug for the second dose of that week. At the site visit on Day 36 and Day 64, each subject will return to the site every 4 weeks to receive drug administration and assessments. At these visits (Days 36 and 64) each subject will be provided with one dose of the study drug to take at the site and three doses for that week and the following week. On Days 50 and 78, subjects will also attend Pharmacy Visits for study drug dispensing. There is a window of +24 hours for a missed dose in the Part 2 of the study. The study drug will not be taken on consecutive days and the study drug dose will not be doubled at any point during the study (i.e. patients should not take two doses on the same day). Subjects will be provided with four doses for that week and the following week. The number of tablets required at each visit is outlined in Section 5.3.

Each subject will be allocated to one of the 5 treatment arms for the duration of the double-blind randomised treatment phase through an IWRS automated system. The system will provide a treatment code to the Investigator when appropriate information is dialled in regarding a subject. Using the code provided, the Investigator will select the treatment labelled with the corresponding code. The subject will then be provided with the correct dosage as detailed in this protocol at each visit.

6.2 Concomitant Indications and Therapy

Any pre-study anti-depressant therapies administered up to 6 months prior to screening should be recorded. Pre-study non-antidepressant therapies that have been administered up to 30 days prior to screening should be recorded along with any ongoing therapies throughout the study.

Concomitant Therapies (including the current antidepressant treatment(s), if applicable) must be recorded throughout the study beginning with signing of the informed consent (i.e., screening) until the last follow-up visit. Concomitant Therapies will continue to be recorded throughout the study. All therapies (prescription or over-the-counter medications, including vaccines, vitamins, herbal supplements; non-pharmacologic therapies such as electrical stimulation, acupuncture, special diets, exercise regimens) different from the study drug must be recorded in the CRF.

The table below represents a guide to therapies which are allowed or prohibited during the duration of this study. The therapies listed are excluded as they could interfere with efficacy evaluation of the study drug and/or affect subject safety.

Allowed (Y) and Not Allowed (N).

Confidential © 2017 Clintec All Rights Reserved

Table 7: Concomitant Therapies

Drug Class/Therapy	Episodic Use (PRN)	Continuous Use	Comments
Allopurinol	N	Y	
Amantadine	N	N	
Analgesics (e.g., NSAIDS, acetaminophen), except opioids	Y	Y	See "Opioids" row below.
Anorexiants (e.g., phenteramine)	N	N	
Antacids	Y	Y	
Anti-anginal agents	N	N	Subjects with angina are excluded
Anti-arrhythmics	N	N	Subjects with any history of cardiovascular arrhythmias excluded
Anticholinesterase inhibitors	N	N	
Anticoagulants	N	N	
Anticonvulsants	N	N	Subjects with seizures are excluded.<br>Anticonvulsants used for other indications may be allowed (e.g., valproate for migraine, lamotrigine for mood disorder)
Antidepressants (except monoamine oxidase inhibitors)	N	Y	
Antidepressants: Monoamine oxidase inhibitors	N	N	
Antidiarrheal preparations	Y	N	
Anti-emetics	Y	N	
Anti-inflammatory drugs, except steroids	Y	Y	See "Steroid" rows below.
Antipsychotics	N	Y	Use of antipsychotics for treatment of depression is not exclusionary. It would be excluded if being used for psychotic symptoms.
Aspirin	Y	Y	
Benzodiazepines	Y	Y	
Benztropine	Y	Y	
Calcium Channel Blockers	Y	Y	
Chloral hydrate	N	N	
Chloramphenicol	N	N	
Clonidine	Y	Y	
Cough/Cold preparations (except those containing diphenhydramine or dextromethorphan)	Y	N	
CYP3A4 inhibitors - Potent	N	N	Examples (not all-inclusive): indinavir, nelfinavir, ritonavir, clarithromycin, itraconazole, ketoconazole, nefazodone, saquinavir, telithromycin
CYP3A4 inducers - Potent[5]	N	N	Examples (not all-inclusive): efavirenz, nevirapine, barbiturates, carbamazepine, glucocorticoids, modafinil, oxcarbazepine, phenobarbital, phenytoin, rifabutin, rifampin, St. John's wort

[5] NB. subjects should not consume grapefruit, grapefruit juice or Seville oranges for 72 hours before R-107 administration and during the study.

Confidential © 2017 Clintec All Rights Reserved

DHEA	Y	Y	
Electroconvulsive therapy (ECT)	N	N	
Fish oils	Y	Y	
Ginko	Y	Y	
Ginseng	Y	Y	
Guanabenz	N	N	
Guanadrel	N	N	
Guanethidine	N	N	
Guanfacine	N	N	
HIV antiviral drugs	N	N	Subjects testing positive for HIV excluded
Hormones (e.g., contraceptives, thyroid hormones etc.)	N	Y	
Ketanserin	N	N	
Lithium	Y	Y	Subjects with TRD should continue to take Lithium medication if this is part of their current treatment.
Methyldopa	N	N	
Metyrosine	N	N	
Naltrexone	N	N	
Opioids	Y	N	Opioids should be used only when required episodically, for example, to treat severe pain.
Omega-3-fatty acids	N	Y	
Other formulations of Ketamine	N	N	
Psychostimulants (e.g., amphetamines)	Y	Y	Prescribed psychostimulants (e.g., amphetamine, methylphenidate, etc.) taken for an indication other than MDD are permitted to continue to take this medication during the study
Reserpine	N	N	
Scopolamine	N	N	
Sleep-aids (non-benzodiazepine)	Y	Y	
St. John's Wort	N	N	
Steroids (inhaled, topical, ophthalmic only)	Y	Y	
Steroids (oral)	N	N	
Thyroid hormone supplement	N	Y	Subjects needing supplements must be on a stable thyroid supplement dose for at least 3 months prior to Day 1 of the double-blind treatment phase.
Tramadol	Y	N	Tramadol can be used only when required episodically, for example, to treat pain.

6.3 Rescue Medication

Subjects showing clinically significant worsening of symptoms (as measured through change in MADRS, CADSS, eC-SSRS, BPIC-SS score) will be immediately withdrawn from the study if:

- MADRS score of ≥22

  OR

Confidential © 2017 Clintec All Rights Reserved

- The Principal Investigator sees fit that a subject is withdrawn because of significant tolerability and/or safety issues.

Subjects who are withdrawn from the study should consult their own treating physician about receiving further clinical care. If appropriate, the study investigator may provide an opinion to the treating physician on further treatment options. However, the blind will not be broken in these cases.

Confidential © 2017 Clintec All Rights Reserved

7 STUDY DATA COLLECTION

7.1 Study Visits

A total of 17 site visits and 2 pharmacy visits will be required by subjects with a total of 25 doses over the total study duration for the enriched population.

7.1.1 Screening

Screening of subjects will take place from Days -28 to -1 prior to drug administration. The following assessments will take place at the screening visit:

- Informed consent
- Inclusion/exclusion criteria
- Demographics and medical history
- Medication history
- Physical examination
- Vital signs
- Weight and height
- Concomitant Medications
- Concurrent medical conditions
- Clinical laboratory tests
- HIV/Hepatitis panel
- Serum pregnancy test
- FSH
- Urine drug screen/alcohol breath test
- Safety ECG
- MINI assessment for MDD diagnosis
- MSM scale for TRD assessment
- MoCA scale for cognition
- MADRS
- CADSS
- eC-SSRS
- BPIC-SS
- PGI-I
- PGI-S
- CGI-S
- WSAS
- EQ-5D-5L
- BPRS+

Confidential © 2017 Clintec All Rights Reserved

- PTE assessment

Screen Failures:

Subjects who sign an ICF, and are not assigned to a treatment, and do not receive either R-107 or placebo, are defined as screen failures. For all screen failures, the following information will be captured in the electronic data capture (EDC) system: screening number, subject demographics, and reason(s) for screen failure into the EDC system. Serious Adverse Event (SAE) information will also be captured in the EDC.

Re-screening:

Approval to re-screen will be granted on a case by case basis by the Medical Monitor and the global study team. The request and rationale to re-screen should be documented. There will be no waivers granted for eligibility criteria at Day 1 of the Enrichment phase. Subjects not meeting the MADRS criteria at Day 8 (≥50% reduction of their MADRS total score which is ≤12) will not be re-screened.

7.1.2 Day 1 Visit

On Day 1 visit, all subjects who have been successfully screened will receive a single dose of R-107 120 mg (2 x 60 mg). This visit will provide the baseline measures for the study and the following assessments will be taken on this visit:

- Inclusion/exclusion criteria
- Vital signs - prior to and 30 minutes after dosing
- Weight
- Concomitant medications
- Concurrent medical conditions
- Clinical laboratory tests
- Urine pregnancy test
- Urine drug screen/alcohol breath test
- Safety ECG
- MADRS
- CADSS – performed pre-dose and 2 hours after dosing
- eC-SSRS
- BPIC-SS
- PGI-I
- PGI-S
- CGI-S
- WSAS
- EQ-5D-5L
- BPRS+ – performed pre-dose and 2 hours after dosing
- Verbal fluency

Confidential © 2017 Clintec All Rights Reserved

- SDMT
- Study drug dosing
- PTE assessment
- AE assessment

7.1.3 Open-Label Treatment Phase - Days 2-5

Following the baseline Day 1 visit, four consecutive visits will be made in the days following for repeated doses to select the enrichment population. The assessments made on these visits are as follows:

- Vital signs - prior to and 30 minutes after dosing
- Concomitant medications
- MADRS – performed at Day 5
- CADSS
- Study drug dosing
- AE assessment

7.1.4 Early Termination

Non-responders may be removed from the study on Day 8 or earlier. The procedures for subjects discontinued early should be consulted. In such cases the following assessments would be carried out:

- Physical examination
- Vital signs
- Weight
- Concomitant medications
- Clinical laboratory tests
- Safety ECG
- MoCA scale for cognition (the version of the MoCA assessment used should differ from the version used at the previous visit)
- MADRS
- CADSS
- eC-SSRS
- BPIC-SS
- PGI-I
- PGI-S
- CGI-S
- WSAS
- EQ-5D-5L
- BPRS+
- Verbal fluency

Confidential © 2017 Clintec All Rights Reserved

- SDMT
- AE assessment

7.1.5 Randomisation

The subjects will visit the site on Day 8 when randomisation will occur, and the following assessments will be performed at this visit:

- Physical examination
- Vital signs - prior to and 30 minutes after dosing
- Concomitant medications
- Clinical laboratory tests
- Safety ECG
- MoCA scale for cognition (the version of the MoCA assessment used should differ from the version used at the previous visit)
- MADRS
- CADSS
- eC-SSRS
- BPIC-SS
- PGI-I
- PGI-S
- CGI-S
- WSAS
- EQ-5D-5L
- BPRS+
- Verbal fluency
- SDMT
- Study drug dosing and dispensing
- AE assessment
- PK sample collection (pre-dose and 4hrs post-dose)

Randomisation will be controlled through IWRS. During randomisation, when subject information is entered into the system (subject identification number etc.) the system will generate a treatment number corresponding to one of the 5 treatment arms. This process maintains double-blinding as the Investigator and the subject are blind to treatment arm.

7.1.6 Randomised, Double-blind Treatment Phase

The treatment period will occur from Days 8-92. A total of 12 weeks where the drug is administered twice weekly in a dose that is randomised to either 30 mg, 60 mg, 120 mg, 180 mg or placebo. The following outlines the assessments which will take place throughout the treatment period.

Days 15, 22, 29, 36 and 64:

Confidential © 2017 Clintec All Rights Reserved

- Physical examination
- Vital signs - prior to and 30 minutes after dosing
- Weight
- Concomitant medications
- Clinical laboratory tests
- Urine pregnancy test (Day 64 only)
- Safety ECG (Day 36 and Day 64 only)
- MoCA scale for cognition (the version of the MoCA assessment used should differ from the version used at the previous visit)
- MADRS
- CADSS
- eC-SSRS (since last visit)
- BPIC-SS
- PGI-I
- PGI-S
- CGI-S
- WSAS
- EQ-5D-5L
- BPRS+
- Verbal fluency
- SDMT
- Study drug dosing and dispensing
- AE assessment
- PK sample Collection (pre-dose and 4hrs post-dose; Day 64 only)

In addition to the visits described above, on Day 9 and Day 65 the subject should visit for PK sample collection (24 hours from dose administration).

At each visit the study drug will be administered prior to completion of the assessments. In addition to the site visits, subjects will be contacted by telephone to discuss their compliance with treatment and follow-up and AEs experienced between visits. Subjects will be contacted on Days 11, 18, 25 and 32 which are days subjects are taking a dose at home whilst visiting the study site weekly. After day 36, subjects will be contacted weekly on Days 43, 57, 71 and 85 whilst they are attending the study site once every 4 weeks. This additional contact will aid compliance with dose schedule and assess subject well-being.

Days 50 and 78:

- Pharmacy visit for study drug dispensing

Confidential © 2017 Clintec All Rights Reserved

7.1.7 *Treatment Phase - Check-out Visits*

Check-out visits will occur on Days 92 and 93. The following assessments will take place on visit Day 92:

- Physical examination
- Vital signs - prior to and 30 minutes after dosing
- Weight
- Concomitant medications
- Clinical Laboratory tests
- Urine Pregnancy test
- Safety ECG
- MoCA scale for cognition (the version of the MoCA assessment used should differ from the version used at the previous visit)
- MADRS
- CADSS
- eC-SSRS
- BPIC-SS
- PGI-I
- PGI-S
- CGI-S
- WSAS
- EQ-5D-5L
- BPRS+
- Verbal fluency
- SDMT
- Study drug dosing
- AE assessment
- PK sample collection (pre-dose and 4hrs post-dose)

On visit Day 93 PK sample would be collected for 24 hours post-dose.

7.1.8 *Safety Follow-up Visit*

A safety follow-up visit will take place 28 days after Day 92 or the Early Termination Visit which will involve the following assessments:

- Physical examination
- Vital signs
- Weight
- Concomitant medications
- Clinical laboratory tests

Confidential © 2017 Clintec All Rights Reserved

- Urine pregnancy test
- Safety ECG
- MoCA scale for cognition (the version of the MoCA assessment used should differ from the version used at the previous visit)
- MADRS
- CADSS
- eC-SSRS (since last visit)
- BPIC-SS
- PGI-I
- PGI-S
- CGI-S
- WSAS
- EQ-5D-5L
- BPRS+
- Verbal fluency
- SDMT
- AE assessment

The same assessments which are carried out at the follow-up visit will be carried out for any early termination throughout the treatment period.

Subjects who withdraw or are withdrawn from the study prior to Day 92 may be eligible to enter a separate OLE study, if the reason for withdrawal was a relapse of depressive symptoms during the double-blind treatment phase of the study. Subjects withdrawn because of safety or tolerability issues may also be eligible to enter a separate OLE study, if the issues were unrelated to R-107. The blind will not be broken for Subjects withdrawing prematurely.

Subjects who enter the OLE BEDROC-1 study prior to the Safety Follow-up Visit of BEDROC will not be required to complete the Safety Follow-up Visit of BEDROC.

7.2 Study Assessments

7.2.1 Written Informed Consent

The informed consent will be given by means of ICF, written in non-technical language. The study subject considered for the study should read and consider the study information before signing and dating it and should be given a copy of the signed document. The informed consent should be signed and dated before starting any study related procedures. After the signature of the informed consent, the subject will be centrally assigned a subject's screening number by the Sponsor or Delegated Party and, upon completion of the screening evaluation, the subject, if eligible, will be enrolled.

7.2.2 Demography

The following demographical details will be taken and recorded in the CRF:

Confidential © 2017 Clintec All Rights Reserved

- Age
- Gender
- Date of birth
- Race
- Ethnicity
- Height
- Weight
- BMI

7.2.3 *Physical Examination*

Care should be taken to examine and assess any abnormalities that may be present as indicated by the subject's medical history. Any abnormality at screening should be reported in the Baseline Signs and Symptoms CRF page, and during treatment should be reported in the AE CRF page. Subjects should be examined during rest. Physical Examinations will take place at screening, Day 8 (randomisation), once weekly throughout the treatment period until Day 36 and then once every 4 weeks up to Day 92 (check-out) during the double-blinded treatment phase, at the safety follow-up visit and at early termination as required.

7.2.4 *Vital Signs*

Vital signs will be recorded at every visit, with the exception of Day 9, Day 65 and Day 93 which are visits to assess PK at 24 hours post-dose only. Vital signs will be measured prior to and 30 minutes after dosing at all visits.

The following vital signs will be assessed:

- BP (systolic and diastolic measured with an electronic monitor)
- Pulse rate (radial)
- Body temperature (Celsius scale with an electronic thermometer)
- Respiratory rate
- Blood oxygen saturation (pulse oximeter)

An electronic blood pressure monitor will be used to measure BP. Measurements will be made on the subject's non-dominant arm supported at heart level, using the same cuff size at each assessment.

7.3 Laboratory Assessments

Laboratory tests should be performed at the local site laboratory. Clinical laboratory test will include haematology, serum chemistry, urinalysis, serum pregnancy test, urine pregnancy test, FSH, Hepatitis panel and HIV testing. A total of 37 blood samples will be required from each subject throughout the entire study. Samples required for laboratory testing will total approximately 87 mL from each subject over the study duration (Table 8). The following tests will be done at the study sites.

7.3.1 *Haematology Tests*

Haemoglobin, haematocrit, RBCs, WBCs with differential count (neutrophils, lymphocytes, monocytes, eosinophils, basophils, and differential other cells), and platelet count. These tests will be performed at screening, Day 1, Day 8, once weekly throughout the treatment period

Confidential © 2017 Clintec All Rights Reserved

until Day 36 and then once every 4 weeks up to Day 92 (check-out), follow-up visit and at early termination as appropriate.

7.3.2 Serum Chemistry

Sodium, potassium, chloride, blood glucose, uric acid, total protein, BUN or urea, creatinine, albumin, AST/SGOT, ALT/SGPT, ALP, total bilirubin, direct bilirubin, LDH, ammonia, calcium, phosphorus, magnesium, amylase and lipase will be tested. These tests will be performed at screening, Day 1, Day 8, once weekly throughout the treatment period until Day 36 and then once every 4 weeks up to Day 92 (check-out), follow-up visit and at early termination as appropriate.

7.3.3 Urinalysis Tests

Complete urinalysis will include specific gravity, pH, protein, glucose, ketones, blood, and microscopic analysis. These tests will be performed at screening, Day 1, Day 8, once weekly throughout the treatment period until Day 36 and then once every 4 weeks up to Day 92 (check-out), follow-up visit and at early termination as appropriate.

7.3.4 Serum Pregnancy Test

A blood sample will be used to perform a serum pregnancy test at screening only.

7.3.5 Pregnancy Urine Test

A urine dip-stick pregnancy test will be performed at Day 1 visit and again at Days 64 and 92 (check-out) visits. This will also be performed in the safety follow-up visit and in the case of early termination.

7.3.6 FSH Test

An FSH level will be obtained for post-menopausal subjects (defined as continuous amenorrhea >12 months and not surgically sterile) at screening only.

7.3.7 Hepatitis and HIV

HBsAg, HbeAg, anti-Hbe [HbeAb], anti-Hbs [HbsAb], anti-Hbc [HbcAb], hepatitis C antibody, hepatitis C RNA, and anti-HIV testing is to be completed at screening only.

7.4 Efficacy Assessments

7.4.1 MADRS

The Montgomery-Asberg Depression Rating Scale (MADRS) (Appendix 1) is a clinician assessed scale comprised of 10 items, specifically sensitive to anti-depressive treatment effects in major depressive subjects. This scale is short and reliable and, therefore, useful for fast use by clinicians (Montgomery and Asberg, 1979). The following symptoms are addressed in the scale:

- Apparent sadness
- Reported sadness
- Inner tension
- Reduced sleep

Confidential © 2017 Clintec All Rights Reserved

- Reduced appetite
- Concentration difficulties
- Lassitude
- Inability to feel
- Pessimistic thoughts
- Suicidal thoughts

Each item is rated from 0 to 6 with six being the worst. The overall score ranges from 0 to 60. A higher MADRS score indicates more severe depression.

In depression, 'response' is commonly defined as a >50% reduction in the initial symptom score and remission is typically defined as a total score of <10. The primary efficacy evaluation will be the change from baseline in the MADRS total score in each period in the double-blind treatment phase.

7.4.2 PGI-I

The patient global impression-improvement score (PGI-I) (Appendix 2) is a 7-point scale which allows the subject to assess their own improvement in disease compared to a baseline measure prior to treatment. They rate their improvement as:

1. Very much improved
2. Much Improved
3. Minimally improved
4. No change
5. Minimally worse
6. Much worse
7. Very much worse

7.4.3 PGI-S

Patient Global Impression-Severity (PGI-S) (Appendix 3) provides an overall summary of the subject's perception of the severity of their depression.

7.4.4 CGI-S

The Clinical Global Impression-Severity (CGI-S) score (Appendix 4) is a well-established rating tool for many psychiatric disorders that can be utilised quickly in a clinical environment. The CGI-S scale provides an overall clinical summary measure, considering all available information including symptoms, subject history and psychological circumstances. The scale asks a single question to the clinician: "Considering your total clinical experience with this particular population, how mentally ill is the patient at this time?" (Busner and Targum, 2007). The response is in the form of a seven-point scale:

1. Normal, not at all ill
2. Borderline mentally ill
3. Mildly ill
4. Moderately ill

Confidential © 2017 Clintec All Rights Reserved

5. Markedly ill
6. Severely ill
7. Among the most extremely ill patients

7.4.5 EQ-5D-5L

The EQ-5D is a standardised 2-part instrument for use as a measure of health outcome, primarily designed for self-completion by respondents. The EQ-5D-5L (Appendix 5) descriptive system comprises the following 5 dimensions: mobility, self-care, usual activities, pain/discomfort and anxiety/depression. Each dimension has 5 levels: no problems, slight problems, moderate problems, severe problems, and extreme problems. The descriptive system can be represented as a health state. The second part of the instrument comprises a visual analogue scale (EQ-VAS), on which patients are asked to rate their current state of health on a scale of 0 to 100.

7.4.6 WSAS

The Work and Social Adjustment Scale (WSAS) (Appendix 6) is a reliable and valid measure of impaired functioning. It is a sensitive and useful outcome measure offering the potential for readily interpretable comparisons across studies and disorders. This is a simple, 5-item scale used to assess the treatment of depression (Mundt et al, 2002).

7.5 Safety Assessments

Evaluations of AEs, concomitant medications, physical examination findings, vital signs (oral body temperature, pulse rate [radial], respiratory rate, BP and blood oxygen saturation), body weight and 12-lead ECGs will be performed throughout the study to monitor subject safety.

Safety laboratory tests includes haematology, serum chemistry and urinalysis assessments.

7.5.1 BPRS

The Brief Psychiatric Rating Scale (Appendix 7) is an assessment specifically designed to measure subject's change with regard to positive psychiatric symptoms including grandiosity, suspiciousness, hallucinations, unusual thought content and conceptual disorganisation. The scale contains 5 items, each of which are rated on a 7-point scale (Overall and Gorham, 1962).

1. Not present
2. Very mild
3. Mild
4. Moderate
5. Moderately Severe
6. Severe
7. Extremely Severe

7.5.2 CADSS

The Clinician Administered Dissociative States Scale (CADSS) (Appendix 8) is used as a measurement of dissociative states. Dissociative states encapsulate a number of symptoms

Confidential © 2017 Clintec All Rights Reserved

including amnesia, depersonalisation, derealisation and identity disturbances. The CADSS is a 19-item scale (Bremner et al., 1998). Each item is scored from 0 to 4 as follows:

1. 0 = not at all
2. 1 = slightly
3. 2 = moderately
4. 3 = considerably
5. 4 = extremely

7.5.3 eC-SSRS

The electronic Columbia-Suicide Severity Rating Scale (eC-SSRS) (Appendix 9) is a suicidal ideation rating scale created to evaluate suicidality (Baseline and 'since last visit'). It is a computer-automated, patient-reported version of the C-SSRS, in which the user's response to a question prompts and shows the appropriate follow-up questions (if any). It rates an individual's degree of suicidal ideation with "yes" or "no" questions revolving around thoughts about wanting to be dead and actions the individual may have actually done, or preparations made in an attempt of suicide. Each question addresses a different component of the respondent's suicide ideation severity. (The Columbia Lighthouse Project 2018).

7.5.4 BPIC-SS

The Bladder Pain/Interstitial Cystitis Symptom Score (BPIC-SS) (Appendix 10) is a systematic scoring questionnaire to assess a subject's bladder pain or cystitis symptoms. It records 5 questions scored 0-5 for never to always, 2 questions scored 0-4 for not at all to a great deal, and 1 global question recording the worst bladder pain in the last 7 days from 0-10. It provides a total scoring range from 0-38.

7.5.5 Verbal Fluency (category and letter)

Verbal fluency tests (Appendix 11) are a psychological test in which participants have to produce as many words as possible from a category or a letter in a given time (usually 60 seconds). This category can be semantic, including objects such as animals or fruits. Letter fluency will be assessed by Controlled Oral Word Association Test. Performance measure is the total number of correct non-repetitive words produced in 60 seconds (Shao, 2014).

7.5.6 SDMT

The Symbol Digit Modalities Test (SDMT) (Appendix 12) detects cognitive impairment and is used by clinicians to screen for organic cerebral dysfunction in both children (eight years and older) and adults. The SDMT is sensitive to detecting the presence of brain damage and also changes in cognitive functioning over time and in response to treatment. The SDMT involves a simple substitution task that normal children and adults can easily perform. Using a reference key, the test taker has 90 seconds to pair specific numbers with given geometric figures (Pereira, 2015).

7.5.7 Electrocardiogram (ECG)

Safety ECG will be measured by standard stationary 12-lead ECG machines. ECG will be recorded at screening, Day 1), Days 8, 36, 64, 92, at follow-up safety visit and Early

Confidential © 2017 Clintec All Rights Reserved

Termination, as appropriate (pre-dose [within 30 minutes prior to dosing]. ECG testing will be completed on site and then sent to be centrally read, maintaining consistency in results across sites. ECG will be assessed at the previously mentioned visits before dosing and also at 4 hours post dosing on Days 8, 64 and 92. This will be performed in conjunction with the PK assessments which are performed on these dosing site visits.

7.5.8 MoCA

The Montreal Cognitive Assessment (MoCA) (Appendix 15) is a brief cognitive screening tool for Mild Cognitive Impairment. It was validated in the setting of mild cognitive impairment and has subsequently been adopted in numerous other settings clinically. The MoCA test is a one-page 30-point test administered in approximately 10 minutes. MoCA scores range between 0 and 30. A score of 26 or over is considered to be normal (Blair, 2016). Thirty items assessing multiple cognitive domains are contained in the MoCA:

- short-term memory (5 points);
- visuospatial abilities via clock drawing (3 points), and a cube copy task (1 point);
- executive functioning via an adaptation of Trail Making Test Part B (1 point), phonemic fluency (1 point), and verbal abstraction (2 points);
- attention, concentration, and working memory via target detection (1 point), serial subtraction (3 points), digits forward (1 point), and digits backward (1 point);
- language via confrontation naming with low-familiarity animals (3 points), and repetition of complex sentences (2 points);
- orientation to time and place (6 points).

Different versions of the tool are available and should be used at different visits in order to avoid learning effects. The administration sequence of the MoCA assessment will be from Version 8.1 to Version 8.2 to Version 8.3, back to Version 8.1 and so on. The version of the MoCA assessment used at the specified study visit should differ from and will depend on the last version of MoCA administered to the subject, in order to identify any emerging cerebral dysfunction, including short-term memory and visuospatial processing.

7.6 Screening Assessments

At screening the subjects will be evaluated for diagnosis of MDD and TRD by MINI 7.0.2. Additionally, TRD assessment will be performed using Maudsley Staging Method (MSM).

7.6.1 MINI version 7.0.2

Mini-International Neuropsychiatric Interview (MINI) (Appendix 13) is used to assess the 17 most common psychiatric disorders in DSM-III-R, DSM-IV and DSM-5 and ICD-10. The MINI *is* designed as a brief structured diagnostic interview to meet the need for a short but accurate structured psychiatric interview for multicentre clinical trials and epidemiology studies and to be used as a first step in outcome tracking in non-research clinical settings. *T*he MINI is a structured interview in which patients are asked to answer questions "Yes" or "No" (e.g., "Were you ever depressed or down, or felt sad, empty or hopeless most of the day, nearly every

Confidential © 2017 Clintec All Rights Reserved

day, for two weeks?"). The MINI is designed to map onto diagnoses defined by the DSM-5 (Sheehan, 1998). For the current study the following modules will be completed:

- Major Depressive Episode (Module A)
- Suicidality (Module B)
- Manic Episode (Module C)
- Any Psychotic disorders (Module K)
- Alcohol use disorder (Module I)
- Substance use disorder (Module J)

7.6.2 MSM

Maudsley Staging Method (MSM) (Appendix 14) was developed to support the effort to better understand and stage TRD. The first and the core dimension is treatment failure. The MSM incorporates severity (dimension 2) and duration of the depressive episode (dimension 3) as important dimensions to quantify treatment-resistance. The MSM defines treatment-resistance as: *failure to attain significant level of improvement (equated with clinical remission) from an accurately defined depressive episode following treatment with an antidepressant medication given at an adequate (minimum effective) dose for a minimum of six weeks* (Fekadu, 2018).

7.7 Pharmacokinetic (PK) Assessments

Venous blood samples will be collected for a population PK Assessment throughout the duration of treatment to measure the concentration of study drug and any other metabolites of interest at timepoints indicated in the Study Schedule. Dates and times of when blood is taken for PK assessment must be recorded in the CRF.

Blood samples will be collected On Day 8 (pre-dose and 4hrs post-dose), Day 9 (24hrs post-dose), Day 64 (pre-dose and 4hrs post-dose), Day 65 (24hrs post-dose), Day 92 (pre-dose and 4hrs post-dose) and Day 93 (24hrs post-dose). A total of 9 samples will be required for PK assessment throughout study duration, at approximately 4 mL per sample, a total volume of 36 mL per subject will be taken throughout study duration for PK assessment.

Plasma samples will be analysed to determine concentrations of R-107 using a validated, achiral LC-MS/MS method by a central laboratory. If required, plasma samples may be analysed to document the presence of circulating metabolites using a qualified research method. Additionally, plasma PK samples may be stored for future analysis of the metabolite profile. The bioanalytical report, including a description and a summary of the assay performance data, will be included as part of the final study report.

Table 8: Approximate Volume of Blood to be Collected from each subject.

Type of Sample	Volume (mL) per sample	Number of Samples per Subject	Total Volume of Blood (mL)
**Screening**			
Serum Chemistry	2.5	1	2.5
Haematology	2.0	1	2.0

Confidential © 2017 Clintec All Rights Reserved

Type of Sample	Volume (mL) per sample	Number of Samples per Subject	Total Volume of Blood (mL)
Serology – HIV	3.5	1	3.5
Serology - Hepatitis	2.5	1	2.5
Approximate total blood volume for screening phase			10.5
**Open-Label**			
Serum Chemistry	2.5	1	2.5
Haematology	2.0	1	2.0
Approximate total blood volume for open-label phase			4.5
**Randomisation and Double-blinded Treatment**			
Serum Chemistry	2.5	7	17.5
Haematology	2.0	7	14.0
Pharmacokinetics	4.0	9	36.0
Approximate total blood volume for treatment phase			67.5
**Follow-up**			
Serum Chemistry	2.5	1	2.5
Haematology	2.0	1	2.0
Approximate total blood volume for follow-up phase			4.5
**Approximate total blood volume for study**			**87.0 mL**

All PK samples will be sent to a central laboratory for assessment.

Confidential © 2017 Clintec All Rights Reserved

8 ADVERSE EVENTS

Definition (ICH-GCP): An adverse event (AE) is any untoward medical occurrence in a subject or clinical investigation subject administered a pharmaceutical product and which does not necessarily have a causal relationship with this treatment. An AE can therefore be any unfavourable and unintended sign (including an abnormal laboratory finding), symptom, or disease temporarily associated with the use of a medicinal (investigational) product, whether or not related to the medical (investigational) product.

The responsibility of the Investigator to record any AEs starts when the ICF is signed.

8.1 Documentation and Reporting of AEs

The Investigator must record all AEs on standard AE reporting form in the electronic Case Report Form (eCRF). The Investigator will be required to describe the AE, onset and stop date, severity, the course of action taken, if any, as well as any pertinent data necessary to allow a complete evaluation of the AE. Relevant AE data will be obtained at the study visits, based on information spontaneously provided by the subject and/or thorough questioning of the subject and through diary card data recorded by the subject.

If a physician not involved with the study sees a subject in relation to an AE, the Investigator should make every effort to contact the treating physician in a timely manner to obtain all information necessary to appropriate reporting of the event. As the quality and precision of acquired AE data are critical, Investigators should use the AE definitions provided below and should observe the following guidelines when completing the AE pages of the CRFs.

- Whenever possible, recognized medical terms should be used to describe AEs rather than colloquialisms (for example, ‘influenza’ rather than ‘flu’), and abbreviations should be avoided.
- AEs should be described using specific clinical diagnosis, if this is available, rather than a list of component signs or symptoms (for example, ‘congestive heart failure’ rather than ‘dyspnoea, rales and cyanosis’).
- Signs and symptoms that are not linked (as co-manifestations) to an identified disease or syndrome, or for which an overall diagnosis is not available, should be reported as individual AEs.
- Any medical condition that is present at the time that the subject is screened will be considered as baseline and not reported as an AE. However, if the study subject’s condition worsens at any time during the study, or if the Investigator believes there is a causal relationship between the study intervention protocol-required therapies and disease worsening it will be recorded as an AE or a serious adverse event (SAE). Worsening indicates that the pre-existing medical condition or underlying disease (e.g., diabetes, migraine headaches, gout) has increased in severity, frequency, and/or duration more than would be expected, and/or has an association with a significantly worse outcome than expected. A pre-existing condition that has not worsened more than anticipated (i.e., more than usual fluctuation of disease) during the study or involves an

Confidential © 2017 Clintec All Rights Reserved

intervention such as elective cosmetic surgery or a medical procedure while on study, is not considered an adverse event.

- Provisional diagnosis (e.g. 'suspected myocardial infarction') are acceptable but should be followed up to a definite diagnosis when available.
- AEs describing an infection must be as specific as possible i.e. type and location of infection must be provided.

Investigators need to evaluate the severity of AEs using the following definitions:

- ***Mild:*** The subject is aware of the event or symptom, but the event or symptom is easily tolerated.
- ***Moderate:*** The subject experiences sufficient discomfort to interfere with or reduce their usual level of activity.
- ***Severe:*** Significant impairment of functioning: the subject is unable to carry out usual activities and therapeutic intervention is required.

AEs characterised as intermittent require documentation of onset and duration of each episode. If the severity of an AE changes from the date of onset to the date of resolution, record as a single event with the worst severity on the event CRF.

The relationship of the AE/SAE to the study drugs will be assessed according to the classification given below.

Certain:

- Event or laboratory test abnormality, with plausible time relationship to drug intake.
- Cannot be explained by disease or other drugs.
- Response to withdrawal plausible (pharmacologically, pathologically).
- Event definitive pharmacologically or phenomenologically (i.e., an objective and specific medical disorder or a recognised pharmacological phenomenon).
- Re-challenge satisfactory, if necessary.

Probable:

- Event or laboratory test abnormality, with reasonable time relationship to drug intake.
- Unlikely to be attributed to disease or other drugs.
- Response to withdrawal clinically reasonable.
- Re-challenge not required.

Possible:

- Event or laboratory test abnormality, with reasonable time relationship to drug intake.
- Could also be explained by disease or other drugs.
- Information on drug withdrawal may be lacking or unclear.

Unlikely:

Confidential © 2017 Clintec All Rights Reserved

- Event or laboratory test abnormality, with a time to drug intake that makes a relationship improbable (but not impossible).
- Disease or other drugs provide plausible explanations.

Not related:

- Event or laboratory test abnormality definitely not associated with the study medication.

Not assessable:

- Report suggesting an adverse reaction.
- Cannot be judged because information is insufficient or contradictory.

8.2 Follow-up of Adverse Events

All AEs must be recorded until a subject completes the entire duration of the study (28 days after the last dose of the study drug). Any ongoing AEs secondary to the study treatment will be followed up for 12 months or until resolution, whichever occurs first. After which the study team will confirm with the subject's primary physician concerning ongoing care for any medical issues. For SAEs that are ongoing at study completion (28 days after the last dose of the study drug), they should be followed until resolution or are diagnosed as chronic.

8.3 Serious Adverse Events

Definition (ICH-GCP): A Serious Adverse Event (SAE) is any untoward medical occurrence, that at any dose:

- results in death
- is life threatening
- requires in-patient hospitalization (at least an overnight stay required) or prolongation of existing hospitalization
- results in persistent or significant disability/incapacity
- is a congenital anomaly/birth defect
- Or other medically important events.

8.3.1.1 <u>SAE Reporting</u>

A Safety Management Plan outlining the execution of clinical trial drug safety activities will be provided. All stakeholders of the study will be trained to ensure a systematic approach to safety monitoring and to identify, evaluate, minimize and appropriately manage the risks.

The responsibility of the Investigator to record any SAE starts when the ICF is signed and ends with the end of study visit (safety follow-up visit – 28 days after the last dose of the study drug) of each subject. SAEs will be reported through RAVE EDC. All SAEs, regardless of relationship to the study agents or study procedures, must be reported to Clintec and Douglas Pharmaceuticals Ltd. by the Investigator within 24 hours of observation or notification of the event. Douglas Pharmaceuticals Ltd. assumes responsibility for appropriate reporting of SAEs

Confidential © 2017 Clintec All Rights Reserved

to regulatory authorities. The Investigator must also report to the appropriate EC that approved the protocol.

Upon learning of a new SAE or of new information on a previously reported SAE (follow-up), the Investigator must notify the clinical research associate (CRA) and Douglas Pharmaceuticals Ltd. Drug Safety representatives (see below) by email within 24 hours of learning of the event or the new information.

Roger Smart and Manar Al-Murrani<br>Douglas Pharmaceuticals Limited<br>Central Park Drive<br>Lincoln 0651, Auckland<br>New Zealand<br>T: +64 (0)9 835 0660<br>E: DrugSafety@douglas.co.nz	Medical Monitor<br>Douglas Pharmaceuticals Limited

Even if an SAE form report cannot be fully completed at this stage, the following minimum information is required as initial notification:

1) The subject's identification details
2) The current medication details
3) The diagnosis of the event with the description (or event description if the diagnosis is not available) and the date of its occurrence
4) Causality assessment and the reason for considering the events serious

The Investigator or delegate must be available within the 2 calendar days from initial notification to allow all required information to be obtained on the case. The Investigator must comply with any applicable requirements specifically related to the reporting of SAEs involving his/her subjects to the EC that approved the study. Specifically, all deaths must be promptly reported to this EC.

In accordance with ICH-GCP guidelines, Douglas Pharmaceuticals Ltd. or its designee will inform the Investigators of findings that could adversely affect the safety of subjects and/or impact the conduct of the study or alter the EC's favourable opinion to continue the study.

Any ongoing SAEs at the time of the subject's participation in the study will be followed up thereafter until an outcome is known, e.g. resolution is reached.

8.4 Pregnancy Reporting

Treatment should generally be discontinued for pregnant subjects if this can be done safely and excluded from the trial. The Investigator will be asked to submit a Pregnancy Report Form if a

Confidential © 2017 Clintec All Rights Reserved

trial subject becomes or is found to be pregnant while receiving the IMP or within 30 days after last study product intake. This must be done irrespective of whether an AE has occurred and within 24 hours of awareness of the pregnancy. The information submitted should include the anticipated date of delivery (see below for information related to induced termination of pregnancy).

The Investigator will counsel the subject as there is no information about foetal exposure and limited information that can be derived from animal studies. If the subject chooses to continue the pregnancy, then consent should be obtained to allow the Investigator to follow the pregnancy to term or longer if possible for developmental sequelae.

The Investigator will follow-up with the subject until completion of the pregnancy or until pregnancy termination (i.e., induced abortion) and then notify Clintec/Douglas Pharmaceuticals Ltd. of the outcome within 5 days or as specified below. The Investigator will provide this information as a follow-up to the initial Pregnancy Report Form. The reason(s) for an induced abortion must be specified.

If the outcome of the pregnancy meets the criteria for immediate classification as an SAE (i.e., spontaneous abortion, stillbirth, neonatal death, or congenital anomaly [including that in an aborted foetus]), the Investigator should follow the procedures for reporting SAEs, i.e., report the event to the Sponsor. In the case of a live birth, the “normality” of the new-born can be assessed at the time of birth.

Additional information about pregnancy outcomes that are classified as SAEs follows:

- “Spontaneous abortion” includes miscarriage and missed abortion.
- All neonatal deaths that occur within 1 month of birth should be reported, without regard to causality, as SAEs.

If a partner of a subject taking the IMP becomes pregnant while the subject is still on study treatment or within 90 days after last dose of IMP, the subject taking the IMP should notify the Investigator, and the pregnant partner should be advised to call their healthcare provider immediately. If a pregnancy related event is reported in a partner of a subject, the Investigator should obtain consent from the partner to share information with Clintec/Douglas Pharmaceuticals Ltd. and allow the pregnancy related event to be followed up to completion. The Investigator must submit the information of pregnancy or suspect pregnancy of a partner of a subject, to Clintec/Douglas Pharmaceuticals Ltd. by e-mail, using the Pregnancy Report Form, within 24 hours of awareness of the event by the Investigator.

8.5 Significant Safety Issues and Urgent Safety Measures.

A significant safety issue (SSI) is a new safety issue or validated signal considered in relation to the IMP that requires urgent attention. This may be because of the seriousness and potential major impact on the benefit-risk balance of the IMP and/or on patient or public health, which could warrant prompt regulatory action and/or communication to patients and healthcare professionals.

Confidential © 2017 Clintec All Rights Reserved

Significant safety issues can be identified as a result of ongoing review and analysis of information that is pertinent to the safety or benefit-risk balance of the medicine.

Examples of significant safety issues include:

- Changes in the nature, severity or frequency of known serious adverse reactions which are medically significant
- Series of reports of similar or linked adverse reactions reported at the same time (that is, a cluster) assessed to suggest a quality defect issue that may have implications for public health
- An unusual and significant lack of efficacy occurring that may have implications for public health
- Major safety findings from a newly completed non-clinical study, post-registration study or clinical trial

Significant safety issues requiring implementation of urgent safety measures should be notified to the Clintec/Douglas Pharmaceuticals Ltd. by e-mail within 24 hours of awareness of the issue/measure by the Investigator.

8.6 Disease-Related Events

Disease-related events (DRE) are events (serious or non-serious) anticipated to occur in the study population due to the underlying disease. DREs and/or Disease Related Outcomes that do not qualify as SAE are:

- Events which are part of the normal course of disease under study (e.g., disease progression in oncology or hospitalisation due to disease progression) is to be reported as a DRE.
- Death due to the disease under study is to be recorded on the Event CRF.

A DRE is considered as SAE if:

- The subject's pre-existing condition becomes worse than what the investigator would consider typical for a patient with the same underlying condition, or
- If the investigator believes a causal relationship exists between the IMP/protocol-required therapies and the event,
- The event meets at least 1 of the serious criteria.

8.7 Special Reporting

Safety events that require special monitoring and/or safety evaluation include, but are not limited to:

- Overdose of study drug;
- Psychiatric AEs (proportion of subjects deteriorating during treatment will be measured using the BPRS+ and MADRS score);
- AEs on cognitive functioning (measured using the verbal fluency and SDMT);
- Increase in suicidal thoughts and behaviour (measured using the eC-SSRS);

Confidential © 2017 Clintec All Rights Reserved

- Metabolic risk factors will be assessed throughout the duration of the study through physical evaluations (outcomes compared to placebo group);
- Haematological AEs (incidences of neutropenia, agranulocytosis and aplastic anaemia require special attention);
- Suspected abuse/misuse of study drug;
- Inadvertent or accidental exposure to study drug;
- Medication error involving study drug (with or without subject exposure to the study drug, e.g., name confusion);
- Rebound/Withdrawal/Dependence (safety follow-up will assess these areas closely).

Special reporting situations should be recorded in the eCRF. Any special reporting situation that meets the criteria of a SAE should be recorded on the SAE page of the eCRF.

8.8 Data Safety Monitoring Committee

An independent Data Safety Monitoring Committee (DSMC) will be established. The DSMC will be independent of the sponsor, regulatory agencies, ethics committees and investigators.

The DSMC has a primary responsibility for safeguarding the interests of study participants. To fulfil this responsibility, the DSMC will review efficacy and safety data on a regular basis during the study. The DSMC also has a responsibility to review and evaluate study conduct to determine whether the study integrity may affect the ability of the DSMC to fulfil its primary responsibility. Therefore, the DSMC will review and evaluate the safety data collected during the study and will provide recommendations to the sponsor about stopping or continuing the study based on their review of this safety data.

The DSMC will include at least four members, including two members with relevant clinical experience (Psychiatrist), one member with experience in early phase clinical research and one statistician with experience related to clinical trials. It is not required that all DSMC members have prior DSMC experience, however the DSMC Chair is required to have prior DSMC experience. The DSMC is an independent, multidisciplinary group consisting of clinicians and biostatisticians that collectively has experience in the management of subjects with mental illness and/or in the conduct and monitoring of randomised studies. The members will be selected by the sponsor and should be deemed acceptable by the participating investigators.

The DSMC will formally meet within 28 days of the following recruitment milestones:

- When 20 patients have completed the enrichment phase of the BEDROC study
- When 50 patients have completed the enrichment phase of the BEDROC study
- When 100 patients have completed the enrichment phase of the BEDROC study
- When 150 patients have completed the enrichment phase of the BEDROC study
- The DSMC will then meet quarterly until Last Patient Last Visit of the BEDROC-1 study (~8 formal meetings)

Confidential © 2017 Clintec All Rights Reserved

All available safety data for the BEDROC and BEDROC-1 studies will be reviewed by the DSMC at each scheduled meeting.

The independent DSMC Chair will review SAEs monthly and has the ability to call a formal meeting if required.

8.9 Medical monitoring

In addition to the safety measures for actively reporting AE and SAEs, a medical monitor assigned to the project will proactively monitor all patient safety aspects. The medical monitor at Douglas Pharmaceuticals Ltd. and Clintec, will provide medical oversight to ensure that the trial is conducted and documented as per ICH-GCP, applicable Standard Operating Procedures (SOPs) and regulatory requirements. The major activities for a medical monitor will include:

- Eligibility criteria review;
- Medical query handling throughout the study;
- Patient data review via patient profiles or data listings for medical consistency (frequency - monthly);
- Medical review of serious adverse events (SAEs);
- SAE reconciliation;
- Safety narrative review;
- Handling emergency unblinding;
- Provide medical oversight to ensure protocol and regulatory compliance;
- Ensure training of team regarding the study protocol and procedures.

Confidential © 2017 Clintec All Rights Reserved

9 DATA HANDLING, RECORDING and QUALITY ASSURANCE

9.1 Electronic Case Record Forms

Electronic Data Capture (EDC) will be used for this study. The study data will be transcribed by study site personnel from the source documents onto an electronic CRF (eCRF). An eCRF must be completed and dated for each enrolled study subject by the Investigator or authorised delegate.

Completed or partially completed eCRF will be made available to the Clintec or Delegated Party Representatives, Regulatory Authorities or, in case the eCRF serves as source document, to the study subject's physicians.

It is the Investigator's or authorised delegate's responsibility to ensure completion and to review and authorise release of the eCRF for each enrolled study subject. The signature on the eCRF serves to attest that the information contained in the eCRF is true, accurate and reliable.

At all times, the Investigator has full responsibility for the accuracy, legibility, completeness, and timeliness of all data (e.g., clinical data, laboratory results) reported in the eCRF and in the related reports (e.g., Data Clarification, Discrepancy Notes, Queries).

9.2 Source Documentation

Source data are all the information in original records and certified copies of original records of clinical findings, observations, or other activities in the study, which are necessary for the reconstruction and evaluation of the study. The Investigator will permit study-related monitoring, audits, IEC reviews and regulatory inspections, with a direct access to all the required source records.

At a minimum, source documentation must be available for the following to confirm data collected in the CRF: subject identification, eligibility, and study identification; study discussion and date of signed informed consent; dates of visits; results of safety and efficacy parameters as required by the protocol; record of all AEs and follow-up of AEs; concomitant medication; drug receipt/dispensing/return records; study drug administration information; and date of study completion and reason for early discontinuation of study drug or withdrawal from the study, if applicable.

Worksheets may be used for the capture of some data to facilitate completion of the CRF. Any such worksheets will become part of the subject's source documentation.

The source documentation requirements for inclusion and exclusion criteria that specify a need for documented medical history are as follows:

- Referral letter from treating physician, or;
- Complete history of medical notes at the site;
- Discharge summaries;
- Antidepressant treatment in the current episode of depression.

Inclusion and exclusion criteria not requiring documented medical history must be verified at a minimum by subject interview or other protocol required assessment (e.g., physical examination, laboratory assessment) and documented in the source documents.

Confidential © 2017 Clintec All Rights Reserved

Information collected on the eCRF must match the source documents.

9.3 Data Handling and Record Keeping

To enable evaluations/auditing and inspections by the Clinical Trial Monitor, Sponsor or the Delegated Party and Regulatory Authorities, the Investigator agrees to keep the Investigator File and the study subject's source documents. It is the Investigator's responsibility to retain study essential documents for at least 15 years following the formal discontinuation of the study. These documents should be retained for a longer period if required by an agreement with the Sponsor. In such an instance, it is the responsibility of the Sponsor to inform the Investigator/institution as to when these documents no longer need to be retained.

The Investigator's Clinical Trial File contains, but is not limited to, number of the enrolled and screening failures' study subjects (sufficient information to link records, CRF and study subject's records), the original signed Informed Consent/Genetic consent Forms, copies of all CRFs and related Reports (e.g., Data Clarifications, Discrepancy Notes, Queries). Clinical Trial subject's source documents also include detailed records of treatment disposition.

The Investigator must ensure that institutional regulations and the ICF clearly permit study-related monitoring, audits, EC review, and regulatory inspections providing direct access to source data and documents. It is responsibility of the Sponsor or the Delegated Party to inform the Investigator of when these documents can be destroyed. The Investigator must obtain Sponsor, or the Delegated Party written permission before disposing of any records.

If the responsible Investigator retires, relocates, or for other reasons withdraws from the responsibility of keeping the study records, custody must be transferred to a person who will accept the responsibility. The Sponsor must be notified in writing of the name and address of the new custodian. Under no circumstance shall the Investigator relocate or dispose of any study documents before having obtained written approval from the Sponsor.

9.4 Monitoring

The study will be monitored by the CRO. Monitoring and auditing procedures developed by the CRO will be followed, in order to comply with GCP guidelines. Monitoring may be done by personal visits by the site monitor who will review the CRFs and source documents, and/or by frequent communications (letter, email, telephone, and fax). The site monitor will ensure that the investigation is conducted according to protocol design and regulatory requirements. The monitor is responsible for inspecting the CRFs at regular intervals (appropriate for the nature and size of the study) throughout the study to verify adherence to the protocol, completeness, accuracy and consistency of the data and adherence to local regulations on the conduct of clinical research. The monitor should have access to subject records/notes and other study-related source documents (e.g. ECG and laboratory print outs), required to verify the entries on the CRFs.

Regular source data verification (SDV) will be carried out by a Clinical Research Associate (CRA) or Site Monitor as agreed between the Sponsor and Clintec. In addition, regulatory authorities, the EC, and/or the Sponsor's quality assurance personnel or designate may request access to all source documents, CRFs, and other study documentation for on-site audit inspections. The Investigator must provide support for these activities and must guarantee direct access to these documents.

Confidential © 2017 Clintec All Rights Reserved

The Investigator agrees to cooperate with the monitor to ensure that any problems detected in the course of these monitoring visits, including delays in completing CRFs, are resolved.

9.5 Data Management

9.5.1 Responsibilities

The Data Managers will provide all tools, instructions, and training necessary to complete the eCRF, and each user will be issued a unique username and password. The Data Management team will be responsible for data processing, in accordance with the Clintec data management procedures.

The Investigators will have to verify that all data entries in the eCRF are accurate and correct. If some assessments are not done, or if certain information is not available, not applicable, or unknown, the Investigators will have to indicate this in the eCRF. The Investigators will be required to electronically sign off the clinical data.

The Monitors will review the eCRFs and evaluate them for completeness and consistency. The eCRF will be compared with the source documents to ensure that there are no discrepancies. All entries, corrections, and alterations will be made by the Investigator or his/her delegate. The Monitors cannot enter data in the eCRFs.

9.5.2 Data collection and validation

All clinical data will be entered in the eCRF at the investigational sites. For each subject of a given site (screen failure and enrolled), an eCRF must be completed in English, by the Investigator or designee, and signed by the Investigator. A unique subject code will identify the subjects on the eCRF.

Once clinical data of the eCRF have been submitted to the central server, corrections to the data fields will be audit trailed, meaning that the reason for change and the name of the person who performed the change, together with time and date, will be logged. Roles and rights of the site personnel responsible for entering the clinical data into the eCRF will be determined in advance and documented on the “delegation form”.

Automatic checks and listings will be designed and performed according to the data validation plan, developed by Clintec Data Managers and approved by Douglas Pharmaceuticals Ltd. In case of missing values, out of range values, data inconsistencies or values that fail logical checks, queries will be edited in the EDC application. In addition, the Monitors and Data Manager(s) can raise manual queries in the EDC application.

The appropriate investigational staff will answer automatic and manual queries. This will be audit-trailed by the EDC application, meaning that the name of the person who answered and the time and date stamp are captured.

9.5.3 Data coding

AEs and Medical History (MH) will be coded using the last version of MedDRA terminology. Concomitant medications will be coded using WHO Drug 2016 Q1 (or a more recent version) terminology. The medical coding will be performed by Clintec coding specialist and reviewed by a Clintec medical monitor before being submitted to for approval.

Confidential © 2017 Clintec All Rights Reserved

9.6 Quality Assurance

Before enrolling any subjects in this study, Sponsor personnel and the Investigator review the protocol, the Investigator's Brochure, the eCRFs and instructions for their completion, the procedure for obtaining informed consent, and the procedure for reporting AEs and SAEs.

Monitoring visits to the study site will be made periodically during the study to ensure that GCPs and all aspects of the protocol are followed. During monitoring visits, the monitors will verify the adherence to the protocol, the maintenance of all study-related records and the accuracy and completeness of all eCRF entries compared with source data to ensure that:

- Data are authentic, accurate, and complete.
- Safety and rights of subjects are being protected.
- Study is conducted in accordance with the currently approved protocol, ICH-GCP and all applicable regulatory requirements

The Investigator/institution guarantees direct access to source documents the Sponsor, Delegated Party (monitoring, auditing) Regulatory Authorities (inspections). It is important that Investigator(s) and relevant personnel are available during monitoring visits, audits and/or inspections and that sufficient time is devoted to the process.

Following data entry or electronic receipt of data, data validation will take place and Forms/Reports for data clarifications will be addressed to the Investigator. Data management activities will address the coding and review of terms by scientific and clinically qualified staff.

Confidential © 2017 Clintec All Rights Reserved

10 DATA ANALYSIS AND STATISTICAL CONSIDERATIONS

The following information provides an overview of the issues related to the analysis of data collected during the study.

10.1 Determination of Sample Size

The sample size estimate is based on the primary endpoint, which is the change in MADRS total score from baseline to Day 92. The following assumptions have been made in the sample size calculations:

- R-107 compared to placebo, testing each dose sequentially in descending dose in a closed test procedure
- 2-sided alpha (Type 1) error = 0.05
- Beta error (Type 2) = 0.20 (Power=80%)
- Expected clinically relevant difference of 6 MADRS units
- Expected standard deviation of change in MADRS of 7.5 units
- No interim analysis

With these assumptions, the required sample size is n=26 subjects in each group. Allowing for up to 13% drop-out, a total of 150 subjects will be randomised equally between the five treatment groups. Assuming an attrition rate of 25% during the enrichment phase, will require an approximate total of 200 subjects to be recruited into the enrichment phase.

10.2 Statistical Methods

10.2.1 Analysis Populations

Open-Label Safety Set and Double-Blind Safety Set

All safety and tolerability data will be analysed using the Safety Set. The Safety Set will include all subjects who received at least one administration of the study medication. Due to the design of this study incorporating an open-label enrichment phase and a double-blind randomised treatment phase there will be two Safety Sets defined. The Open-Label Safety Set will comprise all subjects who received at least one administration of the study medication during the open-label phase. A Double-Blind Safety Set will comprise all subjects who received at least one administration of the study medication during the double-blind randomised phase.

Full Analysis Set

All primary and secondary efficacy endpoints will be analysed using the Full Analysis Set (FAS). The FAS will be defined as the intention-to-treat (ITT) set and will include all randomised subjects.

Per Protocol Analysis Set

A Per Protocol (PP) Set will be defined and used only for the analysis of the primary endpoint to examine the robustness of the primary analysis. The PP Set is a subset of the FAS and will

Confidential © 2017 Clintec All Rights Reserved

include all randomised subjects who have been treated according to the protocol and fulfil the following criteria:

- All inclusion/exclusion criteria satisfied;
- Absence of protocol violations that are likely to affect the efficacy of treatment;
- Adequate study medication compliance;
- Adequate measurement of the primary variable.

Full details of the PP Set definition will be described in the standalone statistical analysis plan.

PK Population

The PK Population will be defined as all randomised subjects who take at least 1 dose of randomised study medication and have at least 1 post-dose plasma concentration data point.

10.2.2 Randomization Scheme

Randomisation to treatment group will be stratified by study site. A computer-generated randomisation schedule will be used to assign subjects to treatments in a 1:1:1:1:1 ratio amongst the five treatments. The list will be specific to the subject's site. Random permuted blocks of varying sizes will be used to ensure approximate balance of treatment allocation within each site.

10.2.3 Number of Subjects Per Site

The subjects in the trial will be equally distributed across 15 sites in Australia, New Zealand and Singapore. Each site is expected to enrol 13-14 patients in the open-label phase and randomise 10 patients in the double-blind phase.

10.2.4 Primary Endpoint

Change in MADRS from baseline to Day 92

The primary analysis of efficacy will be performed with the FAS population. Additionally, for exploring the robustness of the results, a supportive analysis using the PP population will be carried out.

Change in MADRS from baseline to Day 92 will be assessed with an analysis of covariance (ANCOVA). The analysis will be based on differences in MADRS at Day 92 from baseline MADRS, with dose as a factor and baseline MADRS as a covariate.

Missing values for the Day 92 MADRS score will be imputed using the last available MADRS score (LOCF). If it is assumed that more relapses will occur in the placebo group, and that relapsed patients would have deteriorated further had they remained in the study, then this imputation method is conservative in terms of the estimation of a treatment effect.

The least square mean differences between each active treatment with placebo will be presented along with a 95% confidence interval. The four treatment comparisons from the primary efficacy analysis at Day 92 are:

- R-107 180 mg vs. placebo
- R-107 120 mg vs. placebo
- R-107 60 mg vs. placebo
- R-107 30 mg vs. placebo

Confidential © 2017 Clintec All Rights Reserved

To control family-wise type I error, a fixed sequence step-down closed test procedure will be used for hypothesis testing. Starting with the highest dose, R-107 180 mg will be compared with placebo as the first step. Only if this comparison is statistically significant at the 2-sided 5% level will R-107 120 mg be compared with placebo. Only if the R-107 120 mg dose group is statistically significant to placebo will R-107 60 mg be compared with placebo. Only if the R-107 60 mg dose group is statistically significant to placebo will R-107 30 mg be compared with placebo. A 2-sided significance level of 5% will be used for each comparison. The primary analysis set for this analysis is the FAS, but the analysis will be repeated for the PP set, as a sensitivity analysis.

Two sensitivity analyses will be performed on the primary endpoint. The first is a repeat of the primary analysis using the PP population instead of the FAS. The second is to use the Mixed Model Repeated Measures (MMRM) method, with treatment, time and the interaction of treatment with time, and country as fixed effects, subject as random effect and baseline MADRS as a covariate. This will be run without imputing missing data. If feasible, the MMRM analysis will also be run using a multiple imputation (MI) approach to handling missing data.

10.2.5 Secondary Endpoints

Change in MADRS from baseline to Day 36 and Day 64

Mean treatment difference between each of the R-107 doses and placebo will be estimated at the Day 36 and Day 64 assessments from both the MMRM model and MI analysis described above for the primary endpoint for the FAS. Means will be presented along with the 95% confidence intervals for each comparison.

Response at Day 36, Day 64 and Day 92

Response is defined as ≥ 50% reduction from baseline (Day 1) in MADRS total score. A binary response variable will be derived for each subject at the Day 36, Day 64, and Day 92 time points. Subjects who have withdrawn for lack of efficacy or are lost to follow-up will be regarded as failing to achieve a response. The number and proportion of responses for each treatment group will be tabulated for each timepoint (Day 36, Day 64 and Day 92). At each time point separately, the response rate for each dose of R-107 will be compared with that of placebo for all subjects in the FAS using a Fisher's Exact test of proportions.

Remission at Day 36, Day 64 and Day 92

Remission is defined as a MADRS total score ≤10. A binary remission variable will be derived for each subject at the Day 36, Day 64 and Day 92 time points. Subjects who have withdrawn for lack of efficacy or are lost to follow-up will be regarded as failing to achieve remission. The number and proportion of subjects achieving remission for each treatment group will be tabulated for each timepoint (Day 36, Day 64 and Day 92). At each time point separately, the remission rate for each dose of R-107 will be compared with that of placebo using a Fisher's Exact test of proportions.

Clinician Global Impression – Severity (CGI-S) and Patient Global Impression – Improvement (PGI-I)

The categorical scores from the CGI-S and PGI-I will be tabulated, showing frequency counts and percentages for each assessment during the double-blind treatment phase for the FAS. The

Confidential © 2017 Clintec All Rights Reserved

final CGI-S and PGI-I scores obtained for each subject during the double-blind phase will be assessed for treatment effects using a proportional odds model for the FAS.

Pharmacokinetic (PK) levels of R-107

Blood levels of R-107 will be summarised and analysed using population PK methods. Details will be provided in a standalone PK SAP.

Dose-response investigation

The change from baseline in MADRS will be used to assess the relationship between dose of R-107 and response, where placebo would be treated as 0 mg. Graphical methods and statistical modelling will be used. If possible, preclinical results will be used to identify likely dose-response models which will be considered in the modelling. Full details will be given in the SAP. Similar analyses will be conducted for safety outcomes. The results from the safety and efficacy analyses may be combined informally (e.g. in a graph) to allow a preliminary assessment of benefit-risk.

10.2.6 Exploratory Endpoints

Exploratory endpoints based on binary endpoints will be summarised and analysed for the FAS in the same way as the secondary binary endpoints.

Likewise, exploratory endpoints based on categorical endpoints (PGI-I, and WSAS) will be summarised and analysed for the FAS in the same way as the secondary categorical endpoints.

Continuous variables (EQ-VAS) will be summarised in the same way as the other continuous outcome variables.

Time to relapse will be analysed using Kaplan-Meier analysis. Restricted mean survival time (RMST) from randomization until day 92 will be calculated for each treatment group, and differences compared to the placebo group results will be presented. Patients that do not relapse or who withdraw for other reasons are censored.

10.2.7 Safety Endpoints

The principal assessments of safety will be performed during the double-blind randomised phase of the study and will use the Double-Blind Safety Set. Assessment of safety will be based on the frequency of AEs, clinical laboratory assessments, vital signs, and 12 lead ECGs.

AEs will be coded using the Medical Dictionary for Regulatory Activities (MedDRA). Reports will be provided to the Medical Monitor for approval of the coded terms after the database is clean, prior to unblinding. Treatment emergent (TE) AEs will be defined in this study as an AE that occurred during the double-blind randomised treatment phase. All TE AEs will be summarised by system organ class and preferred term for each treatment group. A list of subjects with TE SAEs and those who discontinue from the double-blind phase of the study due to a TE AE will be provided.

Summary statistics by treatment group at each visit during the double-blind phase will be provided for each laboratory parameter. Summary statistics of changes from baseline to each visit will also be provided for each laboratory parameter. Vital signs, body weight, and 12 lead ECG measurements will also be summarised in the same way. Occurrences of significant

Confidential © 2017 Clintec All Rights Reserved

abnormalities in the changes of laboratory values from baseline will be summarised by treatment group.

Any AEs that occur during the open-label enrichment phase will be listed for the Open-Label Safety Set. As all subjects will be receiving R-107 120 mg during this phase of the study, no comparative tables of AEs will be performed for this phase.

10.2.8 General Considerations

Statistical evaluation will be performed using SAS®, Version 9.4 or later. All data will be presented in data listings and summarised by treatment group using descriptive statistics. For continuous data, descriptive statistics will comprise: N, mean, SD, median minimum and maximum. Categorical data will be summarised using frequency counts and percentages.

10.3 Presentation of Results

Neither the complete nor any part of the results of the study carried out under this protocol, nor any of the information provided by the Sponsor for the purposes of performing the study, will be published or passed on to any third party without the consent of the study Sponsor. Any Investigator involved with this study is obligated to provide the Sponsor with complete test results and all data derived from the study.

The Sponsor will notify study sites immediately upon identifying any aspect of the protocol, including information discovered during site monitoring visits or the study results that may adversely affect the safety, well-being, or medical care of the subjects, or that may affect the willingness of subjects to continue participation in the study, influence the conduct of the study, or that may alter the IRB's approval to continue the study. Investigators agree to notify the IRB of record of any such events.

10.4 Handling of Missing Data

All subjects recruited into the study will be accounted for, including those who did not complete the study along with the reasons for withdrawal. The statistical analysis methods proposed in section 10.2 above are an attempt to include and account for missing values.

Confidential © 2017 Clintec All Rights Reserved

11 STUDY ADMINISTRATIVE PROCEDURES

11.1 Regulatory Authority Approval

The Sponsor will obtain approval to conduct the study at each site from the relevant regulatory authorities within each country, in accordance with applicable regulatory requirements prior to initiating a site. The study will be performed in compliance with ICH-GCP guidelines and relevant guidelines in each country.

11.2 Confidentiality of Subjects

All data obtained throughout the study duration from the subjects involved in the study will be kept confidential. The subjects enrolled in the study will be identified using a subject identification number which will be used throughout the study to identify respective subject's data. All information along with the subject ID will be available to Douglas Pharmaceuticals Ltd. and its representatives and regulatory authorities only for the study purposes outlined in this protocol. No information concerning the study, or the data will be released to any unauthorised third party, without prior written approval from the Sponsor. All information recorded, collected and stored will be done in compliance with the consent of the subject, GPP guidelines, ICH-GCP guidelines, ECs, IRBs, regulatory authorities and local health authorities.

11.3 Delegation of Investigator Responsibilities

The Investigator should ensure that all persons assisting with the study are adequately informed about the protocol, any amendments to the protocol, the study treatments, and their study-related duties and functions. The Investigator should maintain a list of Co-Investigators and other appropriately qualified persons to whom he or she has delegated significant study-related duties on a delegation log, which will be provided by the Sponsor's assigned representative (Clintec International Ltd).

11.4 Central Laboratory

All Pharmacokinetic samples will be sent to a central laboratory for analysis.

All other lab testing (haematology, serum chemistry, urinalysis) will be performed on site following collection at a local accredited laboratory.

11.5 Coding and Decoding of subject data

11.5.1 Randomisation

Each subject will be given an identification number to maintain confidentiality of subject personal information.

On Day 8, subjects will be randomly assigned to a treatment arm based on IWRS randomisation. Subjects will be randomly allocated treatment on a 1:1:1:1:1 ratio (n=30 subjects per treatment arm). The randomisation will be maintained within the IWRS system and will not be disclosed until after the study has completed and the database has been finalised.

Based on the randomisation code, the study drug for the double-blind treatment phase will be packaged and labelled. Unique medication identification numbers will be pre-printed on the study drug labels and assigned as subjects qualify for the study and are assigned to treatment.

Confidential © 2017 Clintec All Rights Reserved

Central randomisation will be implemented in this study. The IWRS will assign a unique treatment code, which will dictate the treatment assignment and matching study drug kit for the subject. The requestor must use their own user identification and personal identification number when contacting the IWRS and will then give the relevant subject details to uniquely identify the subject.

11.5.2 Blinding

In the open-label enrichment phase, blinding procedures are not applicable.

For the randomised double-blind phase, the Investigator will not be provided with randomisation codes. The codes will be maintained within the IWRS, which has the functionality to allow the Investigator to break the blind for an individual subject.

Under normal circumstances, the blind should not be broken until all subjects have completed the study and the database is finalized. Otherwise, the blind should be broken only if specific emergency treatment/course of action would be dictated by knowing the treatment status of the subject. In such cases, the Investigator may in an emergency determine the identity of the treatment by contacting the IWRS. It is recommended that the Investigator contact the Sponsor or its designee if possible to discuss the situation, before breaking the blind. Telephone contact with the medical monitor will be available 24 hours per day, 7 days per week. In the event the blind is broken, the Sponsor must be informed as soon as possible. The date, time, and reason for the unblinding must be documented by the IWRS, and in the source document. The documentation received from the IWRS indicating the code break must be retained with the subject's source documents in a secure manner.

11.5.3 Unblinding

ICH GCP section 5.13.4 states "In blinded trials, the coding system for the investigational product(s) should include a mechanism that permits rapid identification of the product(s) in case of a medical emergency but does not permit undetectable breaks of the blinding." Unblinding is the process by which the allocation code is broken so that the Investigator, clinical staff and/or the study statistician becomes aware of the intervention for a subject in a study. Moreover, unblinding must be undertaken by a pre-determined process to ensure that participating people are not unblinded unnecessarily and the study results are not compromised.

The blind will be broken by the pharmacovigilance team before reporting a Suspected Unexpected Serious Adverse Reaction (SUSAR) to the competent authority. However, the study team, Investigators and ECs are not informed of those cases, thus preserving the blind and keeping the subject valid if he/she continues with the study. If the subject drops out of the study due to the SAE, then that case is sent to the Investigators and ECs.

Equally, emergency unblinding should occur in a responsive manner when it is clinically indicated. Emergency unblinding in a study is required for the following but not limited to the below reasons:

- To take treatment decisions where knowledge of the blinded treatment is necessary for the treatment of an AE/SAE;
- A medical emergency that will influence the subject's treatment;
- When someone, other than subject, accidentally takes the IMP;
- When the subject consumes an incorrect dose.

Confidential © 2017 Clintec All Rights Reserved

The site monitor will provide training to site staff on the process of emergency unblinding and the protocol to be followed if the primary unblinding method fails. A 24-hour Sponsor/Clintec contact number will be provided to site by Site monitor/Sponsor at the start of the study for requesting unblinding. The delegated site staff performing the unblinding will be trained by the site monitor on the minimum information to keep ready for unblinding before connecting to the IWRS. The minimum information includes but is not limited to:

- Protocol/Study Number;
- Site Number;
- Subject Number;
- Randomisation number;
- Subject's Age;
- Any other relevant details specified in the unblinding instructions.

The personnel performing the unblinding should log on to the IWRS system and follow the instructions providing minimum information as requested by the system. The information collected via unblinding should immediately be passed on to the appropriate personnel (for example - The treating doctor should be informed in case of medical emergency).

If the primary method of unblinding fails (IWRS system), envelopes/scratch cards will be provided to each site and stored in a secure location. If the internet connection is down or the system could not be accessed in an emergency, the envelopes/scratch cards can be opened to unblind subject treatment. This back-up unblinding method is required to ensure the safety of subjects at all times.

Site Monitor will ensure that once the code is broken by the designated personnel, the whole event is documented on the Unblinding Report Form. The Site Monitor will train the site staff to record the unblinding event on source notes and subject specific CRF.

11.6 Insurance and Financial Arrangements

Insurance coverage will be provided for all subjects enrolled in the study from the time of the subject's inclusion in the study (i.e. date of signing the ICF). The insurance coverage will be provided by the Sponsor and will be in line with GCP guidance and legal requirements, but also in accordance with local regulations. Depending on the local policies and the services/availabilities of the insurance providers, different providers may be used in individual countries. A confirmation of insurance and corresponding insurance conditions should be archived in the Investigator File.

11.7 Study Termination and Site Closure

Upon completion of the study, the monitor will conduct the following activities in conjunction with the Investigator or site staff, as appropriate, including but not limited to the following:

Confidential © 2017 Clintec All Rights Reserved

- Return of all study data to the Sponsor;
- Data queries;
- Review of site study records for completeness.

The Sponsor reserves the right to temporarily suspend or prematurely discontinue this study at any time for reasons including, but are not limited to, ethical issues or severe non-compliance e.g.:

- New information or other evaluation regarding the safety or efficacy of the study medication that indicates a change in the known risk/benefit profile of the compound, such that the risk/benefit is no longer acceptable for subjects participating in the study;
- Significant violation of GCP that compromises the ability to achieve the primary study objectives or compromises subject safety.

In addition, a study site may be terminated prematurely or suspended at any time, provided there is reasonable cause and sufficient notice is given in advance of the intended termination. Reasons for the early termination of a study site by the Sponsor may include but are not limited to:

- Failure of the Investigator to comply with the protocol, the requirements of the IEC/IRB or local health authorities, the Sponsor's procedures or GCP guidelines;
- Inadequate recruitment of subjects by the Investigator;
- Discontinuation of further study drug development.

If the Sponsor determines such action is needed, the Sponsor will discuss this with the Investigator (including the reasons for taking such action) at that time. When feasible, the Sponsor will provide advance notification to the Investigator of the impending action prior to it taking effect.

The Sponsor will also inform the regulatory authorities of the suspension or termination of the study and the reason(s) for the action. The Investigator must inform the IRB/EC promptly and provide the reason for the suspension or termination.

If the study is prematurely discontinued, all study data must be returned to Sponsor.

The study will not be terminated for commercial reasons.

11.8 Archiving and Record Retention

The following records must be retained by the Investigator for a minimum of 15 years after the Sponsor has notified regulatory authorities that investigations have been discontinued:

- Signed informed consent documents for all subjects;
- Subject identification code list, screening log (if applicable), and enrolment log;
- Record of all communications between the Investigator and the EC;
- Composition of the EC;
- Record of all communication between the Investigator and the CRO;
- List of Co-Investigators and other appropriately qualified persons to whom the Investigator has delegated significant study-related duties, together with their roles in the study and their signatures;
- Copies of CRFs, questionnaires, diaries and of documentation of correction for all subjects;

Confidential © 2017 Clintec All Rights Reserved

- All other source documents (subject records, hospital records, laboratory records, etc.);
- All other documents deemed relevant for this study from those listed in Section 8 of the ICH consolidated guideline on GCP (Essential Documents for the conduct of a clinical trial).

Normally, these records will be held in the Investigator's archives. If the Investigator is unable to meet this obligation, he/she must ask the Sponsor for permission to make alternative arrangements. Details of these arrangements must be documented.

11.9 Study Report and Publication Policy

All publications associated with this study will be coordinated through the Trial Steering Committee. The Trial Steering Committee will consist of the National Lead Investigator from each country and representatives of the Sponsor. No publications will go forward without permission from the Sponsor. The Sponsor will provide the major findings of the study to all the Investigators.

At the Sponsor's request, the submission or other disclosure of a proposed publication will be delayed a sufficient time to allow the Sponsor to seek patent or similar protection of any inventions, know-how or other intellectual or industrial property rights disclosed in the proposed Publication.

As a multi-centre study, the first publication, or disclosure of study results shall be a complete report or disclosure coordinated by the Sponsor. Thereafter, any secondary publications will reference the original publication(s).

If a written contract for the conduct of the study, which includes publication provisions inconsistent with this statement is executed, that contract's publication provisions shall apply rather than this statement.

Any intellectual property developed as a result of this study is solely the property of the Sponsor – Douglas Pharmaceuticals Ltd.

Confidential © 2017 Clintec All Rights Reserved

12 REFERENCES

1. Al-Harbi, Khalid Saad (2012): Treatment-resistant depression: therapeutic trends, challenges, and future directions. In *Patient preference and adherence* 6, pp. 369–388. DOI: 10.2147/PPA.S29716.
2. American Psychiatric Association (2010): Treating Major Depressive Disorder: A Quick Reference Guide.
3. Blair, M., Coleman, K., Jesso, S., Jodoin, VD., Smolewska, K., Erin Warriner, Finger, E., Pasternak, SH. (2016). Depressive Symptoms Negatively Impact Montreal Cognitive Assessment Performance: A Memory Clinic Experience. THE CANADIAN JOURNAL OF NEUROLOGICAL SCIENCES INC. 43 (4), p513-517.
4. Bremner, J., Krystal, J., Putnam, F., Southwick, S., Marmar, C., Charney, D. and Mazure, C. (1998). Measurement of dissociative states with the Clinician-Administered Dissociative States Scale (CADSS). *Journal of Traumatic Stress*, 11(1), pp.125-136.
5. Busner J, Targum S. The Clinical Global Impressions Scale: Applying a Research Tool in Clinical Practice. Psychiatry. 2007;4(7):28-37.
6. Conway, Charles R.; George, Mark S.; Sackeim, Harold A. (2017). Toward an Evidence-Based, Operational Definition of Treatment-Resistant Depression: When Enough Is Enough. In JAMA psychiatry 74 (1), pp. 9–10. DOI: 10.1001/jamapsychiatry.2016.2586.
7. Fekadu, A., Donocik, JG., and Cleare, AJ. (2018). Standardisation framework for the Maudsley staging method for treatment resistance in depression. BMC Psychiatry. 18 (100), p1-13.
8. Fekadu A, Wooderson S, Donaldson C et al, A Multidimensional Tool to Quantify Treatment Resistance in Depression: The Maudsley Staging Method. J Clin Psychiatry 2009;70(2):177-184
9. Kavalali, E. and Monteggia, L. (2012). Synaptic Mechanisms Underlying Rapid Antidepressant Action of Ketamine. *American Journal of Psychiatry*, 169(11), pp.1150-1156.
10. Malhi, G. and Byrow, Y. (2016). Is treatment-resistant depression a useful concept?. *Evidence Based Mental Health*, 19(1), pp.1-3.
11. Manji, H., Drevets, W. and Charney, D. (2001). The cellular neurobiology of depression. *Nature Medicine*, 7(5), pp.541-547.
12. Marcus M, Yasamy MT, van, et al. Depression: a global public health concern. WHO Department of Mental Health and Substance Abuse.
13. Montgomery, S. and Asberg, M. (1979). A new depression scale designed to be sensitive to change. *The British Journal of Psychiatry*, 134(4), pp.382-389.
14. Muller, John; Pentyala, Sahana; Dilger, James; Pentyala, Srinivas (2016): Ketamine enantiomers in the rapid and sustained antidepressant effects. In Therapeutic advances in psychopharmacology 6 (3), pp. 185–192. DOI: 10.1177/2045125316631267.

Confidential © 2017 Clintec All Rights Reserved

15. Mundt, J., Marks, I., Shear, M. and Greist, J. (2002). The Work and Social Adjustment Scale: a simple measure of impairment in functioning. *British Journal of Psychiatry*, 180(05), pp.461-464.
16. Ng, SH., Tse, ML., Ng, HW., Lau, FL. (2010). Emergency department presentation of ketamine abusers in Hong Kong: a review of 233 cases. *Hong Kong Medical Journal*. 16 (1), 6-11.
17. Overall, J. and Gorham, D. (1962). The Brief Psychiatric Rating Scale. *Psychological Reports*, 10(3), pp.799-812.
18. Papakostas, G. and Ionescu, D. (2015). Towards new mechanisms: an update on therapeutics for treatment-resistant major depressive disorder. *Molecular Psychiatry*, 20(10), pp.1142-1150.
19. Paul, Rajib K.; Singh, Nagendra S.; Khadeer, Mohammed; Moaddel, Ruin; Sanghvi, Mitesh; Green, Carol E. et al. (2014): (R,S)-Ketamine metabolites (R,S)-norketamine and (2S,6S)-hydroxynorketamine increase the mammalian target of rapamycin function. In Anesthesiology 121 (1), pp. 149–159. DOI: 10.1097/ALN.0000000000000285.
20. Pereira, DR., Costa1, p., and Cerqueira, J.. (2015). Repeated Assessment and Practice Effects of the Written Symbol Digit Modalities Test Using a Short Inter-Test Interval. *Archives of Clinical Neuropsychology*. 30 (5), p424-434.
21. Sheehan, D.V.; Lecrubier, Y.; Sheehan, K.H.; Amorim, P.; Janavs, J.; Weiller, E.; Hergueta, T.; Baker, R.; Dunbar, G.C. The Mini-International Neuropsychiatric Interview (M.I.N.I.): The development and validation of a structured diagnostic psychiatric interview for DSM-IV and ICD-10. J. Clin. Psychiat. 1998, 59 (Suppl 20), 22–33.
22. Preston, T. and Shelton, R. (2013). Treatment Resistant Depression: Strategies for Primary Care. *Current Psychiatry Reports*, 15(7).
23. Price, J. and Drevets, W. (2012). Neural circuits underlying the pathophysiology of mood disorders. *Trends in Cognitive Sciences*, 16(1), pp.61-71.
24. Rush, A. John; Trivedi, Madhukar H.; Wisniewski, Stephen R.; Nierenberg, Andrew A.; Stewart, Jonathan W.; Warden, Diane et al. (2006): Acute and longer-term outcomes in depressed outpatients requiring one or several treatment steps: a STAR*D report. In The American journal of psychiatry 163 (11), pp. 1905–1917. DOI: 10.1176/ajp.2006.163.11.1905.
25. Sanacora, Gerard; Schatzberg, Alan F. (2015): Ketamine: promising path or false prophecy in the development of novel therapeutics for mood disorders? In Neuropsychopharmacology: official publication of the American College of Neuropsychopharmacology 40 (2), pp. 259–267. DOI: 10.1038/npp.2014.261.
26. Sanacora, G., Zarate, C., Krystal, J. and Manji, H. (2008). Targeting the glutamatergic system to develop novel, improved therapeutics for mood disorders. *Nature Reviews Drug Discovery*, 7(5), pp.426-437.
27. Schwartz, J., Murrough, J. and Iosifescu, D. (2016). Ketamine for treatment-resistant depression: recent developments and clinical applications: Table 1. *Evidence Based Mental Health*, 19(2), pp.35-38.

Confidential © 2017 Clintec All Rights Reserved

28. Shao, Z., Janse1, E., Visser., K and Meyer., AS. (2014). What do verbal fluency tasks measure? Predictors of verbal fluency performance in older adults. *Frontiers in Psychology*. 5 (772), p1-10.
29. The Columbia Lighthouse Project. (2018). *About the Scale The Columbia Lighthouse Project*. [online] Available at: http://cssrs.columbia.edu/the-columbia-scale-c-ssrs/about-the-scale/ [Accessed 15 Feb. 2018].
30. Trivedi, M., Fava, M., Wisniewski, S., Thase, M., Quitkin, F., Warden, D., Ritz, L., Nierenberg, A., Lebowitz, B., Biggs, M., Luther, J., Shores-Wilson, K. and Rush, A. (2006). Medication Augmentation after the Failure of SSRIs for Depression. *New England Journal of Medicine*, 354(12), pp.1243-1252.
31. Undurraga, J. and Baldessarini, R. (2011). Randomized, Placebo-Controlled Trials of Antidepressants for Acute Major Depression: Thirty-Year Meta-Analytic Review. *Neuropsychopharmacology*, 37(4), pp.851-864.
32. Yang, C., Shirayama, Y., Zhang, J., Ren, Q., Yao, W., Ma, M., Dong, C. and Hashimoto, K. (2015). R-ketamine: a rapid-onset and sustained antidepressant without psychotomimetic side effects. *Translational Psychiatry*, 5(9), pp.e632-e632.
33. Zarate, C. A.; Machado-Vieira, R. (2017): Ketamine: translating mechanistic discoveries into the next generation of glutamate modulators for mood disorders. In Molecular psychiatry 22 (3), pp. 324–327. DOI: 10.1038/mp.2016.249.

Confidential © 2017 Clintec All Rights Reserved

13 APPENDICES

13.1 Appendix 1 MADRS

Montgomery-Asberg Depression Scale (MADRS)
Instructions: The ratings should be based on a clinical interview moving from broadly phrased questions about symptoms to more detailed ones which allow a precise rating of severity. The rater must decide whether the rating lies on the defined scale steps (0, 2, 4, 6) or between them (1, 3, 5). It is important to remember that it is only rare occasions that a depressed patient is encountered who cannot be rated on the items in the scale. If definite answers cannot be elicited from the patients, all relevant clues as well as information from other sources should be used as a basis for the rating in line with customary clinical practice. This scale may be used for any time interval between ratings, be it weekly or otherwise, but this must be recorded.
Item List
1. Apparent sadness
2. Reported sadness
3. Inner tension
4. Reduced sleep
5. Reduced appetite
6. Concentration difficulties
7. Lassitude
8. Inability to feel
9. Pessimistic thoughts
10. Suicidal thoughts

1. Apparent Sadness
Representing despondency, gloom and despair, (more than just ordinary transient low spirits) reflected in speech, facial expression, and posture. Rate on depth and inability to brighten up.
0 No sadness
1
2 Looks dispirited but does brighten up without difficulty.
3
4 Appears sad and unhappy most of the time.
5
6 Looks miserable all the time. Extremely despondent.

2. Reported Sadness
Representing reports of depressed mood, regardless of whether it is reflected in appearance or not. Includes low spirits, despondency or feeling of being beyond help without hope. Rate according to intensity, duration and the extent to which the mood is reported to be influenced by events.
0 Occasional sadness in keeping with the circumstances.

Confidential © 2017 Clintec All Rights Reserved

1
2 Sad or low but brightens up without difficulty.
3
4 Pervasive feelings of sadness or gloominess. The mood is still influenced by external circumstances.
5
6 Continuous or unvarying sadness, misery or despondency.

3. Inner Tension
Representing feelings of ill-defined discomfort, edginess, inner turmoil mounting to either panic, dread or anguish. Rate according to intensity, frequency, duration and the extent of reassurance called for.
0 Placid. Only reflecting inner tension.
1
2 Occasional feelings of edginess and ill-defined discomfort.
3
4 Continuous feelings of inner tension or intermittent panic which the patient can only master with some difficulty.
5
6 Unrelenting dread or anguish. Overwhelming panic.

4. Reduced Sleep
Representing the experience of reduced duration or depth of sleep compared to the subject's own normal pattern when well.
0 Sleeps as usual.
1
2 Slight difficulty dropping off to sleep or slightly reduced light or fitful sleep.
3
4 Sleep reduced or broken by at least two hours.
5
6 Less than two or three hours sleep

5. Reduced Appetite
Representing the feeling of loss of appetite compared with when well. Rate by loss of desire for food or the need to force oneself to eat.
0 Normal or increased appetite.
1
2 Slightly reduced appetite.
3
4 No appetite. Food is tasteless.
5
6 Needs persuasion to eat.

6. Concentration Difficulties
Representing difficulties in collecting one's thoughts mounting to incapacitating lack of concentration. Rate according to intensity, frequency, and degree of incapacity produced.

Confidential © 2017 Clintec All Rights Reserved

0 No difficulties in concentrating.
1
2 Occasional difficulties in collecting one's thoughts.
3
4 Difficulties in concentrating and sustaining thought which reduces ability to read or hold a conversation.
5
6 Unable to read or converse without great initiative.

7. Lassitude
Representing a difficulty getting started or slowness initiating and performing everyday activities.
0 Hardly no difficulty in getting started. No sluggishness.
1
2 Difficulties in starting activities.
3
4 Difficulties in starting simple routine activities which are carried out with effort.
5
6 Complete lassitude. Unable to do anything without help.

8. Inability to Feel
Representing the subjective experience of reduced interest in the surroundings, or activities that normally give pleasure. The ability to react with adequate emotion to circumstances or people is reduced.
0 Normal interest in the surroundings and in other people.
1
2 Reduced ability to enjoy usual interest.
3
4 Loss of interest in surroundings. Loss of feelings for friends and acquaintances.
5
6 The experience of being emotionally paralyzed, inability to feel anger, grief or pleasure and a complete or even painful failure to feel for close relatives and friends.

9. Pessimistic Thoughts
Representing thoughts of guilt. Inferiority, self-reproach, sinfulness, remorse and ruin.
0 No pessimistic thoughts.
1
2 Fluctuating ideas of failure, self-reproach or self-depreciation.
3
4 Persistent self-accusations, or definite but still rational ideas of guilt or sin. Increasingly pessimistic about the future.
5
6 Delusions of ruin, remorse or unredeemable sin. Self-accusations which are absurd and unshakable.

Confidential © 2017 Clintec All Rights Reserved

10. Suicidal Thoughts
Representing the feeling that life is not worth living, that a natural death would be welcome, suicidal thoughts, and the preparations for suicide. Suicidal attempts should not in themselves influence the rating.

0 Enjoys life or takes it as it comes.
1
2 Weary of life. Only fleeting suicidal thoughts.
3
4 Probably better off dead. Suicidal thoughts are common, and suicide is considered as a possible solution, but without specific plans or intention.
5
6 Explicit plans for suicide when there is an opportunity. Active preparations for suicide.

Total Score: _________________

Confidential © 2017 Clintec All Rights Reserved

13.2 Appendix 2 PGI-I

Patient Global Impression of Improvement (PGI-I)

Check the number that best describes how your depressive condition is now, compared with how it was before you started treatment:

Very much better	1
Much better	2
A little better	3
No change	4
A little worse	5
Much worse	6
Very much worse	7

Confidential © 2017 Clintec All Rights Reserved

13.3 Appendix 3 PGI-S

Patient Global Impression of Severity (PGI-S)

Considering all aspects of your depression right now would you say your depression is?

None	1
Mild	2
Moderate	3
Severe	4

Confidential © 2017 Clintec All Rights Reserved

13.4 Appendix 4 CGI-S

Clinician Global Impression

1. Severity of illness

Considering your total clinical experience with this particular population, how mentally ill is the patient at this time?

0 = Not assessed	4 = Moderately ill
1 = Normal, not at all ill	5 = Markedly ill
2 = Borderline mentally ill	6 = Severely ill
3 = Mildly ill	7 = Among the most extremely ill patients

Confidential © 2017 Clintec All Rights Reserved

13.5 Appendix 5 EQ-5D-5L

Under each heading, please tick the ONE box that best describes your health TODAY.

MOBILITY

I have no problems in walking about ☐

I have slight problems in walking about ☐

I have moderate problems in walking about ☐

I have severe problems in walking about ☐

I am unable to walk about ☐

SELF-CARE

I have no problems washing or dressing myself ☐

I have slight problems washing or dressing myself ☐

I have moderate problems washing or dressing myself ☐

I have severe problems washing or dressing myself ☐

I am unable to wash or dress myself ☐

USUAL ACTIVITIES (e.g. work, study, housework, family or leisure activities)

I have no problems doing my usual activities ☐

I have slight problems doing my usual activities ☐

I have moderate problems doing my usual activities ☐

I have severe problems doing my usual activities ☐

I am unable to do my usual activities ☐

PAIN / DISCOMFORT

I have no pain or discomfort ☐

Confidential © 2017 Clintec All Rights Reserved

I have slight pain or discomfort ☐

I have moderate pain or discomfort ☐

I have severe pain or discomfort ☐

I have extreme pain or discomfort ☐

ANXIETY / DEPRESSION

I am not anxious or depressed ☐

I am slightly anxious or depressed ☐

I am moderately anxious or depressed ☐

I am severely anxious or depressed ☐

I am extremely anxious or depressed ☐

Confidential © 2017 Clintec All Rights Reserved

- We would like to know how good or bad your health is TODAY.
- This scale is numbered from 0 to 100.
- 100 means the best health you can imagine.
  0 means the worst health you can imagine.
- Mark an X on the scale to indicate how your health is TODAY.
- Now, please write the number you marked on the scale in the box below.

YOUR HEALTH TODAY = ☐

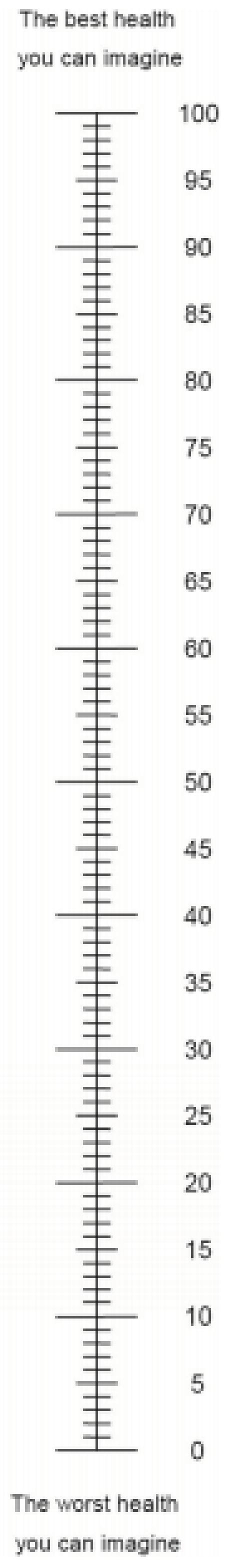

Confidential © 2017 Clintec All Rights Reserved

13.6 Appendix 6 WSAS

Work and Social Adjustment Scale

Rate each of the following questions on a 0 to 8 scale: 0 indicates no impairment at all and 8 indicates very severe impairment.

1. Because of my depression, my ability to work is impaired. 0 means not at all impaired and 8 means very severely impaired to the point I can't work.
2. Because of my depression, my home management (cleaning, tidying, shopping, cooking, looking after home or children, paying bills) is impaired. 0 means not at all impaired and 8 means very severely impaired.
3. Because of my depression, my social leisure activities (with other people, such as parties, bars, clubs, outings, visits, dating, home entertainment) are impaired. 0 means not at all impaired and 8 means very severely impaired.
4. Because of my depression, my private leisure activities (done alone, such as reading, gardening, collecting, sewing, walking alone) are impaired. 0 means not at all impaired and 8 means very severely impaired.
5. Because of my depression, my ability to form and maintain close relationships with others, including those I live with, is impaired. 0 means not at all impaired and 8 means very severely impaired.

Confidential © 2017 Clintec All Rights Reserved

13.7 Appendix 7 BPRS

The Brief Psychiatric Rating Scale

<u>*Scale Items and Anchor Points*</u>

Rate items 1-4 on the basis of individual's self-report. Item 5 is rated on the basis of observed behaviour and speech.

1. <u>*Grandiosity*</u>

Exaggerated self-opinion, self-enhancing conviction of special abilities or powers or identity as someone rich or famous. Rate only individual’s statements about themselves, not their demeanour. Note: ratings of 6 or 7 due to grandiose delusions, should be rated under Unusual Thought at least 4 or above.

1 **Not Present**
2 **Very mild** Feels great and denies obvious problems, but not unrealistic.
3 **Mild** Exaggerated self-opinion beyond abilities and training.
4 **Moderate** Inappropriate boastfulness, e.g., claims to be brilliant, insightful or gifted beyond realistic proportions, but rarely self-discloses or acts on these inflated self-concepts. Does not claim that grandiose accomplishments have actually occurred.
5 **Moderately Severe** Same as 4 but often self discloses and acts on these grandiose ideas. May have doubts about the reality of the grandiose ideas. Not delusional.
6 **Severe** Delusional – claims to have special powers like ESP, to have millions of dollars, invented new machines, worked at jobs when it is known that he was never employed in these capacities, be Jesus Christ, or the President. Patient may not be very preoccupied.
7 **Extremely Severe** Delusional – same as 6, but subject seems very preoccupied and tends to disclose or act on grandiose delusions.

2. <u>*Suspiciousness*</u>

Expressed or apparent belief that other persons have acted maliciously or with discriminatory intent. Include persecution by supernatural or other non-human agencies (e.g., the devil). Note: ratings of 3 or above should also be rated under Unusual Thought Content.

1 **Not Present**
2 **Very mild** Seems on guard. Reluctant to respond to some `personal' questions. Reports being overly self-conscious in public.
3 **Mild** Describes incidents in which others have harmed or wanted to harm him/her that sound plausible. Individual feels as if others are watching, laughing or criticising him/her in public, but this occurs only occasionally or rarely. Little or no preoccupation.
4 **Moderate** Says other persons are talking about him/her maliciously, have negative intentions or may harm him/her. Beyond the likelihood of plausibility, but not delusional. Incidents of suspected persecution occur occasionally (less than once per week) with some preoccupation.
5 **Moderately Severe** Same as 4, but incidents occur frequently, such as more than once per week. Individual is moderately preoccupied with ideas of persecution OR individual reports persecutory delusions expressed with much doubt (e.g., partial delusion).
6 **Severe** Delusional - speaks of Mafia plots, the FBI or others poisoning his/her food, persecution by supernatural forces.
7 **Extremely Severe** Same as 6, but the beliefs are bizarre or more preoccupying. Individual tends to disclose or act on persecutory delusions.

Confidential © 2017 Clintec All Rights Reserved

"Do you ever feel uncomfortable in public? Does it seem as though others are watching you? Are you concerned about anyone's intentions toward you? Is anyone going out of their way to give you a hard time, or trying to hurt you? Do you feel in any danger?"

[If individual reports any persecutory ideas/delusions, ask the following]:
"How often have you been concerned that [use individual's description]? Have you told anyone about these experiences?"

3. *Hallucinations*

Reports of perceptual experiences in the absence of relevant external stimuli. When rating degree to which functioning is disrupted by hallucinations, include preoccupation with the content and experience of the hallucinations, as well as functioning disrupted by acting out on the hallucinatory content (e.g., engaging in deviant behaviour due to command hallucinations). Include thoughts aloud (`gedenkenlautwerden') or pseudohallucinations (e.g., hears a voice inside head) if a voice quality is present.

1 **Not Present**
2 **Very mild** While resting or going to sleep, sees visions, smells odours or hears voices, sounds, or whispers in the absence of external stimulation, but no impairment in functioning.
3 **Mild** While in a clear state of consciousness, hears a voice calling the individual's name, experiences non-verbal auditory hallucinations (e.g., sounds or whispers), formless visual hallucinations or has sensory experiences in the presence of a modality relevant stimulus (e.g., visual illusions) infrequently (e.g., 1-2 times per week) and with no functional impairment.
4 **Moderate** Occasional verbal, visual, gustatory, olfactory or tactile hallucinations with no functional impairment OR non-verbal auditory hallucinations/visual illusions more than infrequently or with impairment.
5 **Moderately Severe** Experiences daily hallucinations OR some areas of functioning are disrupted by hallucinations.
6 **Severe** Experiences verbal or visual hallucinations several times a day OR many areas of functioning are disrupted by these hallucinations.
7 **Extremely Severe** Persistent verbal or visual hallucinations throughout the day OR most areas of functioning are disrupted by these hallucinations.

"Do you ever seem to hear your name being called?"
"Have you heard any sounds or people talking to you or about you when there has been nobody around?

[If hears voices]:
"What does the voice/voices say? Did it have a voice quality?"
"Do you ever have visions or see things that others do not see? What about smell odours that others do not smell?"

[If the individual reports hallucinations, ask the following]:
"Have these experiences interfered with your ability to perform your usual activities/work? How do you explain them? How often do they occur?"

4. *Unusual thought content*

Confidential © 2017 Clintec All Rights Reserved

Unusual, odd, strange, or bizarre thought content. Rate the degree of unusualness, not the degree of disorganisation of speech. Delusions are patently absurd, clearly false or bizarre ideas that are expressed with full conviction. Consider the individual to have full conviction if he/she has acted as though the delusional belief was true. Ideas of reference/persecution can be differentiated from delusions in that ideas are expressed with much doubt and contain more elements of reality. Include thought insertion, withdrawal and broadcast. Include grandiose, somatic and persecutory delusions even if rated elsewhere. Note: if Somatic Concern, Guilt, Suspiciousness or Grandiosity are rated 6 or 7, due to delusions, then Unusual Thought Content must be rated 4 or above.

1 **Not Present**
2 **Very mild** Ideas of reference (people may stare or may laugh at him), ideas of persecution (people may mistreat him). Unusual beliefs in psychic powers, spirits, UFOs, or unrealistic beliefs in one's own abilities. Not strongly held. Some doubt.
3 **Mild** Same as 2, but degree of reality distortion is more severe as indicated by highly unusual ideas or greater conviction. Content may be typical of delusions (even bizarre), but without full conviction. The delusion does not seem to have fully formed but is considered as one possible explanation for an unusual experience.
4 **Moderate** Delusion present but no preoccupation or functional impairment. May be an encapsulated delusion or a firmly endorsed absurd belief about past delusional circumstances.
5 **Moderately Severe** Full delusion(s) present with some preoccupation OR some areas of functioning disrupted by delusional thinking.
6 **Severe** Full delusion(s) present with much preoccupation OR many areas of functioning are disrupted by delusional thinking.
7 **Extremely Severe** Full delusion(s) present with almost total preoccupation OR most areas of functioning disrupted by delusional thinking.

"Have you been receiving any special messages from people or from the way things are arranged around you? Have you seen any references to yourself on TV or in the newspapers?"
"Can anyone read your mind?"
"Do you have a special relationship with God?"
"Is anything like electricity, X-rays, or radio waves affecting you?"
"Are thoughts put into your head that are not your own?"
"Have you felt that you were under the control of another person or force?"

[If individual reports any odd ideas/delusions, ask the following]:
"How often do you think about [use individual's description]?"
"Have you told anyone about these experiences? How do you explain the things that have been happening [specify]?"
Rate items 12-13 on the basis of individual's self-report and observed behaviour.

5. *Conceptual disorganisation*

Degree to which speech is confused, disconnected, vague or disorganised. Rate tangentiality, circumstantiality, sudden topic shifts, incoherence, derailment, blocking, neologisms, and other speech disorders. Do not rate content of speech.

1 **Not Present**
2 **Very mild** Peculiar use of words or rambling but speech is comprehensible.
3 **Mild** Speech a bit hard to understand or make sense of due to tangentiality, circumstantiality, or sudden topic shifts.

Confidential © 2017 Clintec All Rights Reserved

4 **Moderate** Speech difficult to understand due to tangentiality, circumstantiality, idiosyncratic speech, or topic shifts on many occasions OR 1-2 instances of incoherent phrases.

5 **Moderately Severe** Speech difficult to understand due to circumstantiality, tangentiality, neologisms, blocking or topic shifts most of the time, OR 3-5 instances of incoherent phrases.

6 **Severe** Speech is incomprehensible due to severe impairment most of the time. Many BPRS items cannot be rated by self-report alone.

7 **Extremely Severe** Speech is incomprehensible throughout interview.

Confidential © 2017 Clintec All Rights Reserved

13.8 Appendix 8 CADSS

The Clinician Administered Dissociative States Scale (CADSS)

Subjective Items:

1. Do things seem to be moving in slow motion?
   0= Not at all.
   1= Mild, things seem slightly slowed down, but not very noticeable.
   2= Moderate, things are moving about twice as slow as normally.
   3= Severe, things are moving so slowly that they are barely moving.
   4= Extreme, things are moving so slowly, I have the perception that everything has come to a stop, as if time is standing still.

2. Do things seem to be unreal to you, as if you are in a dream?
   0= Not at all.
   1= Mild, things seem a little unreal, but I'm well aware of where I'm at.
   2= Moderate, things seem dreamlike, although I know I am awake.
   3= Severe, things seem very dreamlike, although I know that I am here, I have the feeling like I might be asleep.
   4= Extreme, I feel like nothing is real, like I should pinch myself to wake up, or ask someone if this is a dream.

3. Do you have some experience that separates you from what is happening; for instance, do you feel as if you are in a movie or a play, or as if you are a robot?
   0= Not at all.
   1= Mild, I feel a little bit separated from what is happening, but I am basically here.
   2= Moderate, I feel somewhat separated from what is going on, or I feel as if I am in a movie or a play.
   3= Severe, I feel extremely separated from what is happening, but I can understand what people are saying.
   4= Extreme, I feel as if everyone around me is talking a foreign language, so that I cannot understand what they are saying, or I feel as if I am on the outside looking in, or like I am a robot or a machine.

4. Do you feel as if you are looking at things from outside of your body?
   0= Not at all.
   1= Mild, I feel somewhat disconnected from myself, but I am basically all together.
   2= Moderate, I feel like I am just outside of my body, but not looking down upon myself from far above.
   3= Severe, I feel like I am twenty feet or more away from my body, looking down from above.

Confidential © 2017 Clintec All Rights Reserved

4= Extreme, I feel as if I am hundreds of feet above myself, looking down at myself and everyone else here.

5. Do you feel as if you are watching the situation as an observer or a spectator?
   0= Not at all.
   1= Mild, I feel slightly detached from what is going on, but I am basically here.
   2= Moderate, I feel somewhat removed as an observer or a spectator, but I am definitely in this room.
   3= Severe, I feel very much as if I am an observer or a spectator, but I am still here in this room.
   4= Extreme, I feel completely removed from what is happening, as if I am not a part of this experience in any way, but totally removed from what is happening, as an observer or a spectator.

6. Do you feel disconnected from your own body?
   0= Not at all.
   1= Mild, I feel a little bit disconnected from myself, but I am basically all here.
   2= Moderate, I feel somewhat detached from my own body, but I am basically all together.
   3= Severe, I feel detached from my own body, but not far removed from my body, and I feel as if it is me there.
   4= Extreme, I feel like I am completely out of my body, as if I am looking at my own body from a long way off, as if there is another person there.

7. Does your sense of your own body feel changed: for instance, does your own body feel unusually large or unusually small?
   0= Not at all.
   1= Mild, I have a vague feeling that something about my body has changed, but I can’t say exactly what it is.
   2= Moderate, I feel like my body has increased or decreased in size slightly, or that it feels somewhat as if it is not my body.
   3= Severe, I feel as if my body has increased to twice its normal size, or decreased to twice its normal size, or I very much feel as if this is not my body.
   4= Extreme, I feel as if my body has swelled up to at least ten times its normal size, or as if it is ten times as small, or as if my arms have become like toothpicks.

8. Do people seem motionless, dead, or mechanical?
   0= Not at all.
   1= Mild, people seem a little bit more motionless, dead, or mechanical than would be normal.
   2= Moderate, people seem to be at least twice as motionless or mechanical than would be normal.
   3= Severe, people seem to be barely moving, or barely alive, or very mechanical.

Confidential © 2017 Clintec All Rights Reserved

4= Extreme, it's as if everyone were frozen or completely like machines.

9. Do objects look different than you would expect?
   0= Not at all.
   1= Mild, things seem slightly different than normal, although it is barely perceptible.
   2= Moderate, things are somewhat distorted, but I have no problems recognizing things around me.
   3= Severe, things are much more distorted or unreal than normal, but I am able to recognize things in the room.
   4= Extreme, like everything is distorted, not real, I feel like I cannot recognize anything, everything is alien or strange.

10. Do colours seem to be diminished in intensity?
   0= Not at all.
   1= Mild, things seem slightly paler than usual if I think about it.
   2= Moderate, colours are somewhat diminished, but still recognizable.
   3= Severe, colours are extremely pale, in no way as vivid as they usually are.
   4= Extreme, as if everything is in black and white, or all the colours have been washed out.

11. Do you see things as if you were in a tunnel, or looking through a wide angle photographic lens?
   0= Not at all.
   1= Mild, I feel a little bit like I am looking through a tunnel, or a wide angle lens.
   2= Moderate, the periphery of my vision is blacked out, but I still have most of my visual field, or things are somewhat like a wide angle lens.
   3= Severe, it seems as if I'm looking through a tunnel, or through a wide angle lens, but I can see everything clearly.
   4= Extreme, as if I'm looking through a pair of binoculars backwards, where everything around the periphery is blacked out, and I can see a little point of light at the end of a tunnel, with little tiny people and objects, or I am seeing things as if through a wide lens and things are incredibly expanded.

12. Does this interview [assessment, questionnaire] seem to be taking much longer than you would have expected?
   0= Not at all.
   1= Mild, it seems as if this interview has gone on for at least twice as long as the true elapsed time.
   2= Moderate, it seems as if this interview has gone on for at least two hours.
   3= Severe, it seems as if at least ten hours have gone on since the start of the interview.

Confidential © 2017 Clintec All Rights Reserved

4= Extreme, it seems as if time is standing still, so that we have been here at this point in time forever.

13. Do things seem to be happening very quickly, as if there is a lifetime in a moment?
0= Not at all.
1= Mild, things are happening slightly faster than normal.
2= Moderate, things seem to be happening at least twice as fast as normal.
3= Severe, things seem to be happening at least 10 times faster than normal.
4= Extreme, as if this whole experience has happened at once, or as if there is a lifetime in a moment.

14. Have there been things which have happened during this interview [assessment] that now you can't account for?
0= Not at all.
1= Mild, there may have been things which happened which now I can't account for, but nothing pronounced.
2= Moderate, at least once there were things which happened which now I can't account for.
3= Severe, at least twice I have lost several minutes of time, so that now there are things I cannot account for.
4= Extreme, large pieces of time are missing, of ten minutes or more, so that I am confused about what has happened.

15. Have you spaced out, or in some other way lost track of what was going on during this experience?
0= Not at all.
1= Mild, I have had some episodes of losing track of what is going on, but I have followed everything for the most part.
2= Moderate, I have lost at least a minute of time, or have completely lost track of what is going on now.
3= Severe, I have lost several segments of time of one minute or more.
4= Extreme, I have lost large segments of time of at least 15 minutes or more.

16. Have sounds almost disappeared or become much stronger than you would have expected?
0= Not at all.
1= Mild, things are either a little quieter than normal, or a little louder than normal, but it is not very noticeable.
2= Moderate, things have become about twice as soft as normal, or twice as loud as normal.
3= Severe, things have become very quiet, as if everyone is whispering, or things have become very loud (although not deafening).

Confidential © 2017 Clintec All Rights Reserved

4= Extreme, things have become completely silent, or sounds are so loud that it is deafening, and I feel as if I am going to break my eardrums.

17. Do things seem very real, as if there is a special sense of clarity?
    0= Not at all.
    1= Mild, things seem to be a little bit more real than normal.
    2= Moderate, things seem to be more real than normal.
    3= Severe, things seem to be very real or have a special sense of clarity.
    4= Extreme, things seem to have an incredible sense of realness or clarity.

18. Does it seem as if you are looking at the world through a fog, so that people and objects appear far away or unclear?
    0= Not at all.
    1= Mild, things seem somewhat foggy and unclear, or I do have the feeling that things are far away, but there is not a major effect on how I perceive things around me.
    2= Moderate, things seem very foggy and unclear, or things seem like they are far away, but I can identify the interviewer and objects in the room easily.
    3= Severe, I can barely see things around me, such as the interviewer and the objects in the room.
    4= Extreme, I cannot make anything out around me.

19. Do colours seem much brighter than you would have expected?
    0= Not at all.
    1= Mild, colours seem a little bit brighter than normal, but not more than twice as bright.
    2= Moderate, colours seem brighter, about twice as bright as normal.
    3= Severe, colours seem very bright, at least five times as bright as normal.
    4= Extreme, colours seem extremely bright, almost fluorescent, at least 10 times as bright as normal.

Confidential © 2017 Clintec All Rights Reserved

13.9 Appendix 9 eC-SSRS

The electronic Columbia Suicide Severity Rating Scale - Baseline

eC-SSRS 2.0 Web Script – Lifetime

Sheet Name:
en-US-Web

Key:

**NRT** – No response text<br>**Q** - Question<br>I – Months for recent ideation<br>B – Months for recent behavior	* **NOTES ABOUT THE WEB TEXT:**<br>1.) Text between the **and** notations will appear as **bolded** when displayed on the web page.<br>2.) The <br>tags indicates a new paragraph when text is displayed on the web page.<br>3.) The "\\" preceding a comma or a parenthesis is used to properly keep long response phrases together on the web page as a continuous sentence.

Core Language: US-English Web	Translated Language: US-English Web
**Introduction**	**Introduction**
**NRT01**	**NRT01**
In this interview, we are going to ask questions about thoughts you may have had and actions you may have done related to wanting to be dead or killing yourself. <br>**First** we will ask about **thoughts** regarding **wanting to be dead** or killing yourself that you have **not actually** acted on. <br>**Later** we will ask about any actions you may have **actually done or preparations you have made.** We will let you know when we switch from thoughts to actions.	In this interview, we are going to ask questions about thoughts you may have had and actions you may have done related to wanting to be dead or killing yourself. <br>**First** we will ask about **thoughts** regarding **wanting to be dead** or killing yourself that you have **not actually** acted on. <br>**Later** we will ask about any actions you may have **actually done or preparations you have made.** We will let you know when we switch from thoughts to actions.
**Passive Suicide Ideation**	**Passive Suicide Ideation**
**Q01**	**Q01**
At any time in your life, have you wished you were dead or wished you could go to sleep and not wake up?<br><br>Yes<br>No<br><br>**Ideation Level = 1**	At any time in your life, have you wished you were dead or wished you could go to sleep and not wake up?<br><br>Yes<br>No<br><br>**Ideation Level = 1**
**Active Suicide Ideation**	**Active Suicide Ideation**
**Q02**	**Q02**
Have you actually had any thoughts of killing yourself, at any time?<br><br>Yes<br>No<br><br>**Ideation Level = 2**	Have you actually had any thoughts of killing yourself, at any time?<br><br>Yes<br>No<br><br>**Ideation Level = 2**
**Q03**	**Q03**
Have you thought about how you might do this?	Have you thought about how you might do this?

Confidential © 2017 Clintec All Rights Reserved

Core Language: US-English Web	Translated Language: US-English Web
Yes<br>No<br>Ideation Level = 3	Yes<br>No<br>Ideation Level = 3
Q03.1	Q03.1
What way of killing yourself did you think of most often?<br><br>with medication<br>by hanging<br>by jumping<br>with a gun<br>by some other method	What way of killing yourself did you think of most often?<br><br>with medication<br>by hanging<br>by jumping<br>with a gun<br>by some other method
Q04	Q04
At any time, have you ever had any intention of acting on these thoughts of killing yourself? <br>As opposed to, you have the thoughts, but you definitely would not act on them?<br><br>Yes<br>No<br>Ideation Level = 4	At any time, have you ever had any intention of acting on these thoughts of killing yourself? <br>As opposed to, you have the thoughts, but you definitely would not act on them?<br><br>Yes<br>No<br>Ideation Level = 4
Q05	Q05
At any time, have you ever started to work out, or actually worked out, the specific details of how to kill yourself?<br><br>Yes<br>No	At any time, have you ever started to work out, or actually worked out, the specific details of how to kill yourself?<br><br>Yes<br>No
Q05q	Q05q
Did you actually intend to carry out the details of your plan?<br><br>Yes<br>No<br>Ideation Level = 5	Did you actually intend to carry out the details of your plan?<br><br>Yes<br>No<br>Ideation Level = 5
Q05r	Q05r
How did you intend to kill yourself?<br><br>with medication<br>by hanging<br>by jumping<br>with a gun	How did you intend to kill yourself?<br><br>with medication<br>by hanging<br>by jumping<br>with a gun

Core Language: US-English Web	Translated Language: US-English Web
by some other method	by some other method
Ideation Probing	Ideation Probing
Q01aNRT	Q01aNRT
You just indicated that you, at some point, had wished you were dead or wished that you could go to sleep and not wake up. We want to ask you a few more questions about that. <br>When responding to these questions, we want you to think about the time when these thoughts were most severe.	You just indicated that you, at some point, had wished you were dead or wished that you could go to sleep and not wake up. We want to ask you a few more questions about that. <br>When responding to these questions, we want you to think about the time when these thoughts were most severe.
Q01a	Q01a
When your wishes to be dead or to go to sleep and not wake up were most severe, how often did the thoughts occur?<br><br>less than once a week<br>about once a week<br>2 to 5 times a week<br>daily or almost daily<br>many times a day	When your wishes to be dead or to go to sleep and not wake up were most severe, how often did the thoughts occur?<br><br>less than once a week<br>about once a week<br>2 to 5 times a week<br>daily or almost daily<br>many times a day
Q01b	Q01b
How long did the thoughts last?<br><br>fleeting\\, lasting seconds to minutes<br>less than an hour<br>between 1 and 4 hours<br>between 4 and 8 hours<br>more than 8 hours	How long did the thoughts last?<br><br>fleeting\\, lasting seconds to minutes<br>less than an hour<br>between 1 and 4 hours<br>between 4 and 8 hours<br>more than 8 hours
Q01c	Q01c
Did you make any attempt to **try** to control these thoughts about wanting to die or going to sleep and not waking up, whether you were successful in controlling them or not?<br><br>Yes<br>No	Did you make any attempt to **try** to control these thoughts about wanting to die or going to sleep and not waking up, whether you were successful in controlling them or not?<br><br>Yes<br>No
Q01d	Q01d
How easily could you control or stop these thoughts?<br><br>easily controlled these thoughts<br>with a little difficulty<br>with some difficulty<br>with a lot of difficulty	How easily could you control or stop these thoughts?<br><br>easily controlled these thoughts<br>with a little difficulty<br>with some difficulty<br>with a lot of difficulty

Confidential © 2017 Clintec All Rights Reserved

Core Language: US-English Web	Translated Language: US-English Web
unable to control these thoughts	unable to control these thoughts
**Q01e.1**	**Q01e.1**
Did you think about things like family, religion, or fear of pain or death that might affect your wish to be dead or going to sleep and not waking up?<br><br>Yes<br>No	Did you think about things like family, religion, or fear of pain or death that might affect your wish to be dead or going to sleep and not waking up?<br><br>Yes<br>No
**Q01e**	**Q01e**
Choose one of the following statements that best describes whether anyone or anything did or did not stop you from wishing you were dead or that you could fall asleep and not wake up.<br><br>something definitely stopped you<br>something probably stopped you<br>you are uncertain whether something stopped you<br>something most likely did not stop you<br>something definitely did not stop you	Choose one of the following statements that best describes whether anyone or anything did or did not stop you from wishing you were dead or that you could fall asleep and not wake up.<br><br>something definitely stopped you<br>something probably stopped you<br>you are uncertain whether something stopped you<br>something most likely did not stop you<br>something definitely did not stop you
**Q01f.1**	**Q01f.1**
When you thought about wishing to be dead or going to sleep and not waking up, did you have a reason in mind like ending your pain or getting attention or revenge?<br><br>Yes<br>No	When you thought about wishing to be dead or going to sleep and not waking up, did you have a reason in mind like ending your pain or getting attention or revenge?<br><br>Yes<br>No
**Q01f**	**Q01f**
What sort of reasons did you have for thinking about wanting to die? Was it to end the pain or stop the way you were feeling? <br>In other words, you could not go on living with this pain or how you were feeling? Was it to get attention, revenge or a reaction from others? Or both?<br><br>it was completely to get attention\\, revenge or a reaction from others<br>it was mostly to get attention\\, revenge or a reaction from others<br>equally to get attention\\, revenge or a reaction from others and to end or stop the pain<br>mostly to end or stop the pain \\(that is\\, you could not go on living with the pain or how you were feeling\\)<br>completely to end or stop the pain	What sort of reasons did you have for thinking about wanting to die? Was it to end the pain or stop the way you were feeling? <br>In other words, you could not go on living with this pain or how you were feeling? Was it to get attention, revenge or a reaction from others? Or both?<br><br>it was completely to get attention\\, revenge or a reaction from others<br>it was mostly to get attention\\, revenge or a reaction from others<br>equally to get attention\\, revenge or a reaction from others and to end or stop the pain<br>mostly to end or stop the pain \\(that is\\, you could not go on living with the pain or how you were feeling\\)<br>completely to end or stop the pain
**Q02aNRT**	**Q02aNRT**

Core Language: US-English Web	Translated Language: US-English Web
You indicated before that you had thought of killing yourself. We want to ask you a few more questions about that. <br>When responding to these questions, we want you to think about the time you were feeling most suicidal.	You indicated before that you had thought of killing yourself. We want to ask you a few more questions about that. <br>When responding to these questions, we want you to think about the time you were feeling most suicidal.
**Q02a**	**Q02a**
When you were feeling most suicidal, how often did you think of killing yourself?<br><br>less than once a week<br>about once a week<br>2 to 5 times a week<br>daily or almost daily<br>many times a day	When you were feeling most suicidal, how often did you think of killing yourself?<br><br>less than once a week<br>about once a week<br>2 to 5 times a week<br>daily or almost daily<br>many times a day
**Q02b**	**Q02b**
How long did these thoughts of killing yourself last?<br><br>fleeting\\, lasting seconds to minutes<br>less than an hour<br>between 1 and 4 hours<br>between 4 and 8 hours<br>more than 8 hours	How long did these thoughts of killing yourself last?<br><br>fleeting\\, lasting seconds to minutes<br>less than an hour<br>between 1 and 4 hours<br>between 4 and 8 hours<br>more than 8 hours
**Q02c**	**Q02c**
Did you make any attempt to **try** to control or stop these thoughts, whether you were successful in controlling them or not?<br><br>Yes<br>No	Did you make any attempt to **try** to control or stop these thoughts, whether you were successful in controlling them or not?<br><br>Yes<br>No
**Q02d**	**Q02d**
How easily could you control or stop thinking about killing yourself?<br><br>easily controlled these thoughts<br>with a little difficulty<br>with some difficulty<br>with a lot of difficulty<br>unable to control these thoughts	How easily could you control or stop thinking about killing yourself?<br><br>easily controlled these thoughts<br>with a little difficulty<br>with some difficulty<br>with a lot of difficulty<br>unable to control these thoughts
**Q02e.1**	**Q02e.1**
Did you think about things like family, religion, or fear of pain or death that might affect your decision about killing yourself?<br><br>Yes	Did you think about things like family, religion, or fear of pain or death that might affect your decision about killing yourself?<br><br>Yes

Confidential © 2017 Clintec All Rights Reserved

Core Language: US-English Web	Translated Language: US-English Web
No	No
Q02e	Q02e
Choose one of the following statements that best describes whether anyone or anything did or did not stop you from acting on your thoughts of committing suicide.<br><br>something definitely stopped you<br>something probably stopped you<br>you are uncertain whether something stopped you<br>something most likely did not stop you<br>something definitely did not stop you	Choose one of the following statements that best describes whether anyone or anything did or did not stop you from acting on your thoughts of committing suicide.<br><br>something definitely stopped you<br>something probably stopped you<br>you are uncertain whether something stopped you<br>something most likely did not stop you<br>something definitely did not stop you
Q02f.1	Q02f.1
When you thought about killing yourself, did you have a reason in mind like ending your pain or getting attention or revenge?<br><br>Yes<br>No	When you thought about killing yourself, did you have a reason in mind like ending your pain or getting attention or revenge?<br><br>Yes<br>No
Q02f	Q02f
What sort of reasons did you have for thinking about wanting to kill yourself? Was it to end the pain or stop the way you were feeling? <br>In other words, you could not go on living with this pain or how you were feeling? Or was it to get attention, revenge or a reaction from others? Or both?<br><br>it was completely to get attention\\, revenge or a reaction from others<br>it was mostly to get attention\\, revenge or a reaction from others<br>equally to get attention\\, revenge or a reaction from others and to end or stop the pain<br>mostly to end or stop the pain \\(that is\\, you could not go on living with the pain or how you were feeling\\)<br>completely to end or stop the pain	What sort of reasons did you have for thinking about wanting to kill yourself? Was it to end the pain or stop the way you were feeling? <br>In other words, you could not go on living with this pain or how you were feeling? Or was it to get attention, revenge or a reaction from others? Or both?<br><br>it was completely to get attention\\, revenge or a reaction from others<br>it was mostly to get attention\\, revenge or a reaction from others<br>equally to get attention\\, revenge or a reaction from others and to end or stop the pain<br>mostly to end or stop the pain \\(that is\\, you could not go on living with the pain or how you were feeling\\)<br>completely to end or stop the pain
Q03aNRT	Q03aNRT
You indicated before that you had thought about how you might kill yourself. We want to ask you a few more questions about that. <br>When responding to these questions, we want you to think about the time you were feeling the most suicidal.	You indicated before that you had thought about how you might kill yourself. We want to ask you a few more questions about that. <br>When responding to these questions, we want you to think about the time you were feeling the most suicidal.
Q03a	Q03a
When you were feeling most suicidal, how often did you think about how you might kill yourself?<br><br>less than once a week<br>about once a week	When you were feeling most suicidal, how often did you think about how you might kill yourself?<br><br>less than once a week<br>about once a week

Core Language: US-English Web	Translated Language: US-English Web
2 to 5 times a week<br>daily or almost daily<br>many times a day	2 to 5 times a week<br>daily or almost daily<br>many times a day
Q03b	Q03b
When you had these thoughts, how long did they last?<br><br>fleeting\\, lasting seconds to minutes<br>less than an hour<br>between 1 and 4 hours<br>between 4 and 8 hours<br>more than 8 hours	When you had these thoughts, how long did they last?<br><br>fleeting\\, lasting seconds to minutes<br>less than an hour<br>between 1 and 4 hours<br>between 4 and 8 hours<br>more than 8 hours
Q03c	Q03c
Did you make any attempt to **try** to control or stop these thoughts, whether you were successful in controlling them or not?<br><br>Yes<br>No	Did you make any attempt to **try** to control or stop these thoughts, whether you were successful in controlling them or not?<br><br>Yes<br>No
Q03d	Q03d
How easily could you control or stop thinking about how you might kill yourself?<br><br>easily controlled these thoughts<br>with a little difficulty<br>with some difficulty<br>with a lot of difficulty<br>unable to control these thoughts	How easily could you control or stop thinking about how you might kill yourself?<br><br>easily controlled these thoughts<br>with a little difficulty<br>with some difficulty<br>with a lot of difficulty<br>unable to control these thoughts
Q03e.1	Q03e.1
Did you think about things like family, religion, or fear of pain or death that might affect your decision about killing yourself?<br><br>Yes<br>No	Did you think about things like family, religion, or fear of pain or death that might affect your decision about killing yourself?<br><br>Yes<br>No
Q03e	Q03e
Choose one of the following statements that best describes whether anyone or anything did or did not stop you from acting on your thoughts of committing suicide.<br><br>something definitely stopped you<br>something probably stopped you<br>you are uncertain whether something stopped you	Choose one of the following statements that best describes whether anyone or anything did or did not stop you from acting on your thoughts of committing suicide.<br><br>something definitely stopped you<br>something probably stopped you<br>you are uncertain whether something stopped you

Confidential © 2017 Clintec All Rights Reserved

Core Language: US-English Web	Translated Language: US-English Web
something most likely did not stop you<br>something definitely did not stop you	something most likely did not stop you<br>something definitely did not stop you
Q03f.1	Q03f.1
When you thought about killing yourself, did you have a reason in mind like ending your pain or getting attention or revenge?<br><br>Yes<br>No	When you thought about killing yourself, did you have a reason in mind like ending your pain or getting attention or revenge?<br><br>Yes<br>No
Q03f	Q03f
What sort of reasons did you have for thinking about wanting to kill yourself? Was it to end the pain or stop the way you were feeling? <br>In other words, you could not go on living with this pain or how you were feeling? Or was it to get attention, revenge or a reaction from others? Or both?<br><br>it was completely to get attention\\, revenge or a reaction from others<br>it was mostly to get attention\\, revenge or a reaction from others<br>equally to get attention\\, revenge or a reaction from others and to end or stop the pain<br>mostly to end or stop the pain \\(that is\\, you could not go on living with the pain or how you were feeling\\)<br>completely to end or stop the pain	What sort of reasons did you have for thinking about wanting to kill yourself? Was it to end the pain or stop the way you were feeling? <br>In other words, you could not go on living with this pain or how you were feeling? Or was it to get attention, revenge or a reaction from others? Or both?<br><br>it was completely to get attention\\, revenge or a reaction from others<br>it was mostly to get attention\\, revenge or a reaction from others<br>equally to get attention\\, revenge or a reaction from others and to end or stop the pain<br>mostly to end or stop the pain \\(that is\\, you could not go on living with the pain or how you were feeling\\)<br>completely to end or stop the pain
Q04aNRT	Q04aNRT
You indicated before that you had thought about killing yourself and that you had some intention of acting on these thoughts. We want to ask you a few more questions about that. <br>When responding to these questions, we want you to think about the time you were feeling the most suicidal.	You indicated before that you had thought about killing yourself and that you had some intention of acting on these thoughts. We want to ask you a few more questions about that. <br>When responding to these questions, we want you to think about the time you were feeling the most suicidal.
Q04a	Q04a
When you were feeling most suicidal and had some intention of acting on those thoughts of killing yourself, how often did those thoughts occur?<br><br>less than once a week<br>about once a week<br>2 to 5 times a week<br>daily or almost daily<br>many times a day	When you were feeling most suicidal and had some intention of acting on those thoughts of killing yourself, how often did those thoughts occur?<br><br>less than once a week<br>about once a week<br>2 to 5 times a week<br>daily or almost daily<br>many times a day
Q04b	Q04b
How long did the thoughts last?	How long did the thoughts last?

Core Language: US-English Web	Translated Language: US-English Web
fleeting\\, lasting seconds to minutes<br>less than an hour<br>between 1 and 4 hours<br>between 4 and 8 hours<br>more than 8 hours	fleeting\\, lasting seconds to minutes<br>less than an hour<br>between 1 and 4 hours<br>between 4 and 8 hours<br>more than 8 hours
Q04c	Q04c
Did you make any attempt to **try** to control or stop these thoughts about actually killing yourself, whether you were successful in controlling them or not?<br><br>Yes<br>No	Did you make any attempt to **try** to control or stop these thoughts about actually killing yourself, whether you were successful in controlling them or not?<br><br>Yes<br>No
Q04d	Q04d
How easily could you control or stop thinking about killing yourself?<br><br>easily controlled these thoughts<br>with a little difficulty<br>with some difficulty<br>with a lot of difficulty<br>unable to control these thoughts	How easily could you control or stop thinking about killing yourself?<br><br>easily controlled these thoughts<br>with a little difficulty<br>with some difficulty<br>with a lot of difficulty<br>unable to control these thoughts
Q04e.1	Q04e.1
Did you think about things like family, religion, or fear of pain or death that might affect your decision about killing yourself?<br><br>Yes<br>No	Did you think about things like family, religion, or fear of pain or death that might affect your decision about killing yourself?<br><br>Yes<br>No
Q04e	Q04e
Choose one of the following statements that best describes whether anyone or anything did or did not stop you from acting on your thoughts of committing suicide.<br><br>something definitely stopped you<br>something probably stopped you<br>you are uncertain whether something stopped you<br>something most likely did not stop you<br>something definitely did not stop you	Choose one of the following statements that best describes whether anyone or anything did or did not stop you from acting on your thoughts of committing suicide.<br><br>something definitely stopped you<br>something probably stopped you<br>you are uncertain whether something stopped you<br>something most likely did not stop you<br>something definitely did not stop you
Q04f.1	Q04f.1
When you thought about killing yourself, did you have a reason in mind like ending your pain or getting attention or revenge?	When you thought about killing yourself, did you have a reason in mind like ending your pain or getting attention or revenge?

Confidential © 2017 Clintec All Rights Reserved

Core Language: US-English Web	Translated Language: US-English Web
Yes<br>No	Yes<br>No
Q04f	Q04f
What sort of reasons did you have for thinking about wanting to kill yourself? Was it to end the pain or stop the way you were feeling? <br>In other words, you could not go on living with this pain or how you were feeling? Or was it to get attention, revenge or a reaction from others? Or both?<br><br>it was completely to get attention\\, revenge or a reaction from others<br>it was mostly to get attention\\, revenge or a reaction from others<br>equally to get attention\\, revenge or a reaction from others and to end or stop the pain<br>mostly to end or stop the pain \\(that is\\, you could not go on living with the pain or how you were feeling\\)<br>completely to end or stop the pain	What sort of reasons did you have for thinking about wanting to kill yourself? Was it to end the pain or stop the way you were feeling? <br>In other words, you could not go on living with this pain or how you were feeling? Or was it to get attention, revenge or a reaction from others? Or both?<br><br>it was completely to get attention\\, revenge or a reaction from others<br>it was mostly to get attention\\, revenge or a reaction from others<br>equally to get attention\\, revenge or a reaction from others and to end or stop the pain<br>mostly to end or stop the pain \\(that is\\, you could not go on living with the pain or how you were feeling\\)<br>completely to end or stop the pain
Q05aNRT	Q05aNRT
You indicated before that you had started working on plans or had actually worked out the details of how to kill yourself and had some intention to act on them. We want to ask you a few more questions about that. <br>When responding to these questions, we want you to think about the time you were feeling the most suicidal.	You indicated before that you had started working on plans or had actually worked out the details of how to kill yourself and had some intention to act on them. We want to ask you a few more questions about that. <br>When responding to these questions, we want you to think about the time you were feeling the most suicidal.
Q05a	Q05a
When you were feeling the most suicidal and started planning or worked out details of how to kill yourself, how often did you think about killing yourself?<br><br>less than once a week<br>about once a week<br>2 to 5 times a week<br>daily or almost daily<br>many times a day	When you were feeling the most suicidal and started planning or worked out details of how to kill yourself, how often did you think about killing yourself?<br><br>less than once a week<br>about once a week<br>2 to 5 times a week<br>daily or almost daily<br>many times a day
Q05b	Q05b
How long did the thoughts last?<br><br>fleeting\\, lasting seconds to minutes<br>less than an hour<br>between 1 and 4 hours<br>between 4 and 8 hours<br>more than 8 hours	How long did the thoughts last?<br><br>fleeting\\, lasting seconds to minutes<br>less than an hour<br>between 1 and 4 hours<br>between 4 and 8 hours<br>more than 8 hours
Q05c	Q05c

Core Language: US-English Web	Translated Language: US-English Web
Did you make any attempt to **try** to control or stop these thoughts about actually killing yourself, whether you were successful in controlling them or not?<br><br>Yes<br>No	Did you make any attempt to **try** to control or stop these thoughts about actually killing yourself, whether you were successful in controlling them or not?<br><br>Yes<br>No
Q05d	Q05d
How easily could you control or stop thinking about killing yourself?<br><br>easily controlled these thoughts<br>with a little difficulty<br>with some difficulty<br>with a lot of difficulty<br>unable to control these thoughts	How easily could you control or stop thinking about killing yourself?<br><br>easily controlled these thoughts<br>with a little difficulty<br>with some difficulty<br>with a lot of difficulty<br>unable to control these thoughts
Q05e.1	Q05e.1
Did you think about things like family, religion, or fear of pain or death that might affect your decision about killing yourself?<br><br>Yes<br>No	Did you think about things like family, religion, or fear of pain or death that might affect your decision about killing yourself?<br><br>Yes<br>No
Q05e	Q05e
Choose one of the following statements that best describes whether anyone or anything did or did not stop you from acting on your thoughts of committing suicide.<br><br>something definitely stopped you<br>something probably stopped you<br>you are uncertain whether something stopped you<br>something most likely did not stop you<br>something definitely did not stop you	Choose one of the following statements that best describes whether anyone or anything did or did not stop you from acting on your thoughts of committing suicide.<br><br>something definitely stopped you<br>something probably stopped you<br>you are uncertain whether something stopped you<br>something most likely did not stop you<br>something definitely did not stop you
Q05f.1	Q05f.1
When you thought about killing yourself, did you have a reason in mind like ending your pain or getting attention or revenge?<br><br>Yes<br>No	When you thought about killing yourself, did you have a reason in mind like ending your pain or getting attention or revenge?<br><br>Yes<br>No
Q05f	Q05f

Confidential © 2017 Clintec All Rights Reserved

Core Language: US-English Web	Translated Language: US-English Web
What sort of reasons did you have for thinking about wanting to kill yourself? Was it to end the pain or stop the way you were feeling? <br>In other words, you could not go on living with this pain or how you were feeling? Or was it to get attention, revenge or a reaction from others? Or both?	What sort of reasons did you have for thinking about wanting to kill yourself? Was it to end the pain or stop the way you were feeling? <br>In other words, you could not go on living with this pain or how you were feeling? Or was it to get attention, revenge or a reaction from others? Or both?
it was completely to get attention\\, revenge or a reaction from others<br>it was mostly to get attention\\, revenge or a reaction from others<br>equally to get attention\\, revenge or a reaction from others and to end or stop the pain<br>mostly to end or stop the pain \\(that is\\, you could not go on living with the pain or how you were feeling\\)<br>completely to end or stop the pain	it was completely to get attention\\, revenge or a reaction from others<br>it was mostly to get attention\\, revenge or a reaction from others<br>equally to get attention\\, revenge or a reaction from others and to end or stop the pain<br>mostly to end or stop the pain \\(that is\\, you could not go on living with the pain or how you were feeling\\)<br>completely to end or stop the pain
If Recent Ideation is required, go to Recent Ideation Section	If Recent Ideation is required, go to Recent Ideation Section
**Recent Ideation**	**Recent Ideation**
**Level 1 Ideation**	**Level 1 Ideation**
You indicated that at some time in your life you have wished you were dead or that you could go to sleep and not wake up. <br>Have you had any thoughts like that in the past I months?	You indicated that at some time in your life you have wished you were dead or that you could go to sleep and not wake up. <br>Have you had any thoughts like that in the past I months?
Have you wished you were dead or that you could go to sleep and not wake up in the past I months?	Have you wished you were dead or that you could go to sleep and not wake up in the past I months?
Yes<br>No	Yes<br>No
**Level 2 Ideation**	**Level 2 Ideation**
You indicated that there has been a time in your life when you had thought of killing yourself. <br>Have you had any thoughts like that in the past I months?	You indicated that there has been a time in your life when you had thought of killing yourself. <br>Have you had any thoughts like that in the past I months?
Have you had any thoughts of killing yourself in the past I months?	Have you had any thoughts of killing yourself in the past I months?
Yes<br>No	Yes<br>No
**Level 3 Ideation**	**Level 3 Ideation**
You indicated that there has been a time in your life when you thought about how you might kill yourself. <br>Have you thought about how you might kill yourself in the past I months?	You indicated that there has been a time in your life when you thought about how you might kill yourself. <br>Have you thought about how you might kill yourself in the past I months?
Have you thought about how you might kill yourself in the past I months, even though you did not intend to act on the thoughts?	Have you thought about how you might kill yourself in the past I months, even though you did not intend to act on the thoughts?
Yes<br>No	Yes<br>No
**Level 4 Ideation**	**Level 4 Ideation**
You indicated that there has been a time in your life when you thought about how you might kill yourself and that you had some intention of acting on those thoughts. <br>Have you had any intentions of acting on thoughts about killing yourself in the past I months?	You indicated that there has been a time in your life when you thought about how you might kill yourself and that you had some intention of acting on those thoughts. <br>Have you had any intentions of acting on thoughts about killing yourself in the past I months?
Have you had any intentions of acting on a method to kill yourself in the past I months?	Have you had any intentions of acting on a method to kill yourself in the past I months?
Yes<br>No	Yes<br>No
**Level 5 Ideation**	**Level 5 Ideation**
You indicated that there was a time in your life when you worked on a plan or had worked out details for killing yourself and that you had some intention to carry out the plan. <br>Have you made specific plans or worked out the details for killing yourself with the intention of carrying them out in the past I months?	You indicated that there was a time in your life when you worked on a plan or had worked out details for killing yourself and that you had some intention to carry out the plan. <br>Have you made specific plans or worked out the details for killing yourself with the intention of carrying them out in the past I months?
Yes<br>No	Yes<br>No
**Midpoint Transition (NRT02)**	**Midpoint Transition (NRT02)**
We are almost finished. <br>So far we have been asking about thoughts and feelings you may have had. <br>Now we would like to know about things you may have done to try to hurt yourself.	We are almost finished. <br>So far we have been asking about thoughts and feelings you may have had. <br>Now we would like to know about things you may have done to try to hurt yourself.
**Suicidal Behavior**	**Suicidal Behavior**
**Q06a**	**Q06a**
At any time in your life, have you made a suicide attempt?	At any time in your life, have you made a suicide attempt?
Yes<br>No	Yes<br>No
**Q06b**	**Q06b**
Use the number keys to enter the number of suicide attempts you have made.	Use the number keys to enter the number of suicide attempts you have made.
**Q06cNRT01**	**Q06cNRT01**
If attempts >= 3	If attempts >= 3
Consider your most recent attempt, your first attempt, and your most serious attempt separately.	Consider your most recent attempt, your first attempt, and your most serious attempt separately.

Confidential © 2017 Clintec All Rights Reserved

Core Language: US-English Web	Translated Language: US-English Web
**Q06cNRT02**	**Q06cNRT02**
If attempts = 2<br>Consider your most recent attempt and your first attempt separately.	If attempts = 2<br>Consider your most recent attempt and your first attempt separately.
**Q06c**	**Q06c**
If loop = 1<br>When you made your most recent attempt, were you trying to end your life?<br><br>Yes<br>No<br><br>If loop = 2<br>When you made your first attempt, were you trying to end your life?<br><br>Yes<br>No<br><br>If loop = 3<br>When you made your most serious attempt, were you trying to end your life?<br><br>Yes<br>No	If loop = 1<br>When you made your most recent attempt, were you trying to end your life?<br><br>Yes<br>No<br><br>If loop = 2<br>When you made your first attempt, were you trying to end your life?<br><br>Yes<br>No<br><br>If loop = 3<br>When you made your most serious attempt, were you trying to end your life?<br><br>Yes<br>No
**Q06e**	**Q06e**
Did you think it was possible that you could have died from what you did?<br><br>Yes<br>No	Did you think it was possible that you could have died from what you did?<br><br>Yes<br>No
**Q06d**	**Q06d**
So then<br>you wanted to die\\, even a little\\, when you did this<br>you did it purely for other reasons\\, like to relieve stress\\, feel better\\, get sympathy\\, or get something else to happen to you\\, without any intention of killing yourself	So then<br>you wanted to die\\, even a little\\, when you did this<br>you did it purely for other reasons\\, like to relieve stress\\, feel better\\, get sympathy\\, or get something else to happen to you\\, without any intention of killing yourself
**Q07a**	**Q07a**
Have you ever done anything to intentionally hurt or harm yourself?<br><br>Yes	Have you ever done anything to intentionally hurt or harm yourself?<br><br>Yes

Core Language: US-English Web	Translated Language: US-English Web
No	No
**Q07b**	**Q07b**
Use the number keys to enter the number of times you have intentionally hurt or harmed yourself. If you cannot remember the exact number, enter your best estimate.	Use the number keys to enter the number of times you have intentionally hurt or harmed yourself. If you cannot remember the exact number, enter your best estimate.
**Q07cNRT01**	**Q07cNRT01**
Just consider the three most recent times you have intentionally harmed or hurt yourself.	Just consider the three most recent times you have intentionally harmed or hurt yourself.
**Q07c_Attempt**	**Q07c_Attempt**
If loop = 1<br>Think about the time you intentionally hurt or harmed yourself most recently<br><br>If loop > 1<br>Consider the time you hurt or harmed yourself before that	If loop = 1<br>Think about the time you intentionally hurt or harmed yourself most recently<br><br>If loop > 1<br>Consider the time you hurt or harmed yourself before that
**Q07c**	**Q07c**
Were you trying to end your life?<br><br>Yes<br>No	Were you trying to end your life?<br><br>Yes<br>No
**Q07e**	**Q07e**
Did you think it was possible that you could have died from what you did?<br><br>Yes<br>No	Did you think it was possible that you could have died from what you did?<br><br>Yes<br>No
**Q07d**	**Q07d**
So then<br>you wanted to die\\, even a little\\, when you did this<br>you did it purely for other reasons\\, like to relieve stress\\, feel better\\, get sympathy\\, or get something else to happen to you\\, without any intention of killing yourself	So then<br>you wanted to die\\, even a little\\, when you did this<br>you did it purely for other reasons\\, like to relieve stress\\, feel better\\, get sympathy\\, or get something else to happen to you\\, without any intention of killing yourself
**Q08a**	**Q08a**
Have you done anything dangerous where you could have died?<br><br>Yes<br>No	Have you done anything dangerous where you could have died?<br><br>Yes<br>No

Confidential © 2017 Clintec All Rights Reserved

Core Language: US-English Web	Translated Language: US-English Web
**Q08b**<br>Use the number keys to enter the number of times you have done dangerous activities where you could have died.	**Q08b**<br>Use the number keys to enter the number of times you have done dangerous activities where you could have died.
**Q08c_NRT01**<br>Just consider the three most recent times you have done something dangerous where you could have died.	**Q08c_NRT01**<br>Just consider the three most recent times you have done something dangerous where you could have died.
**Q08c_Attempt**<br>If loop = 1<br>Think about the most recent time you did a dangerous activity where you could have died<br><br>If loop > 1<br>Consider the time you did something dangerous before that	**Q08c_Attempt**<br>If loop = 1<br>Think about the most recent time you did a dangerous activity where you could have died<br><br>If loop > 1<br>Consider the time you did something dangerous before that
**Q08c_1**<br>Were you trying to harm yourself when you did this?<br><br>Yes<br>No	**Q08c_1**<br>Were you trying to harm yourself when you did this?<br><br>Yes<br>No
**Q08c**<br>Were you trying to end your life?<br><br>Yes<br>No	**Q08c**<br>Were you trying to end your life?<br><br>Yes<br>No
**Q08d**<br>So then<br>you wanted to die\\, even a little\\, when you did this<br>you did it purely for other reasons\\, like to relieve stress\\, feel better\\, get sympathy\\, or get something else to happen to you\\, without any intention of killing yourself	**Q08d**<br>So then<br>you wanted to die\\, even a little\\, when you did this<br>you did it purely for other reasons\\, like to relieve stress\\, feel better\\, get sympathy\\, or get something else to happen to you\\, without any intention of killing yourself
**Lethality**	**Lethality**
**Q09**<br>If Q06a = YES<br>As a result of your **most serious** attempt, were you injured more seriously than surface scratches or mild nausea?<br><br>Yes<br>No<br><br>If Q06a = NO<br>As a result of the **most serious** time you tried to hurt yourself, were you injured more seriously than surface scratches or mild nausea?<br><br>Yes<br>No	**Q09**<br>If Q06a = YES<br>As a result of your **most serious** attempt, were you injured more seriously than surface scratches or mild nausea?<br><br>Yes<br>No<br><br>If Q06a = NO<br>As a result of the **most serious** time you tried to hurt yourself, were you injured more seriously than surface scratches or mild nausea?<br><br>Yes<br>No
**Q09RB**<br>Did this occur within the past B months?<br><br>Yes<br>No	**Q09RB**<br>Did this occur within the past B months?<br><br>Yes<br>No
**Q09a**<br>Were you hospitalized for **medical** treatment of the physical injury you suffered? <br>For example, were you comatose from an overdose, or did you suffer extensive blood loss that required a transfusion, or severe damage to your head or a vital organ? <br>If you were hospitalized for psychiatric evaluation, but not for medical treatment of a severe physical injury, answer No.<br><br>Yes<br>No	**Q09a**<br>Were you hospitalized for **medical** treatment of the physical injury you suffered? <br>For example, were you comatose from an overdose, or did you suffer extensive blood loss that required a transfusion, or severe damage to your head or a vital organ? <br>If you were hospitalized for psychiatric evaluation, but not for medical treatment of a severe physical injury, answer No.<br><br>Yes<br>No
**Q09b**<br>Were you injured so severely that you would have died without treatment in an intensive care unit, or did you suffer permanent physical damage from which you will never completely recover, such as paralysis or disfigurement?<br><br>Yes<br>No	**Q09b**<br>Were you injured so severely that you would have died without treatment in an intensive care unit, or did you suffer permanent physical damage from which you will never completely recover, such as paralysis or disfigurement?<br><br>Yes<br>No
**Q09c**<br>Did your injury cause you to be extremely drowsy, or result in broken bones, or severe bleeding?<br><br>Yes<br>No	**Q09c**<br>Did your injury cause you to be extremely drowsy, or result in broken bones, or severe bleeding?<br><br>Yes<br>No
**Q09.1NRT**	**Q09.1NRT**

Confidential © 2017 Clintec All Rights Reserved

Core Language: US-English Web	Translated Language: US-English Web
Earlier, you indicated that there were two times when you intended to kill yourself or thought you could have died from what you did. <br>We want to know if you suffered any physical injuries.	Earlier, you indicated that there were two times when you intended to kill yourself or thought you could have died from what you did. <br>We want to know if you suffered any physical injuries.
**Q09.1**	**Q09.1**
If Q06a = YES<br>As a result of your **most recent** attempt, were you injured more seriously than surface scratches or mild nausea?<br><br>Yes<br>No<br><br>If Q06a = NO<br>As a result of the **most recent** time you tried to hurt yourself, were you injured more seriously than surface scratches or mild nausea?<br><br>Yes<br>No	If Q06a = YES<br>As a result of your **most recent** attempt, were you injured more seriously than surface scratches or mild nausea?<br><br>Yes<br>No<br><br>If Q06a = NO<br>As a result of the **most recent** time you tried to hurt yourself, were you injured more seriously than surface scratches or mild nausea?<br><br>Yes<br>No
**Q09.1RB**	**Q09.1RB**
Did this occur within the past B months?<br><br>Yes<br>No	Did this occur within the past B months?<br><br>Yes<br>No
**Q09.1a**	**Q09.1a**
Were you hospitalized for **medical** treatment of the physical injury you suffered? <br>For example, were you comatose from an overdose, or did you suffer extensive blood loss that required a transfusion, or severe damage to your head or a vital organ? <br>If you were hospitalized for psychiatric evaluation, but not for medical treatment of a severe physical injury, answer No.<br><br>Yes<br>No	Were you hospitalized for **medical** treatment of the physical injury you suffered? <br>For example, were you comatose from an overdose, or did you suffer extensive blood loss that required a transfusion, or severe damage to your head or a vital organ? <br>If you were hospitalized for psychiatric evaluation, but not for medical treatment of a severe physical injury, answer No.<br><br>Yes<br>No
**Q09.1b**	**Q09.1b**
Were you injured so severely that you would have died without treatment in an intensive care unit, or did you suffer permanent physical damage from which you will never completely recover, such as paralysis or disfigurement?<br><br>Yes<br>No	Were you injured so severely that you would have died without treatment in an intensive care unit, or did you suffer permanent physical damage from which you will never completely recover, such as paralysis or disfigurement?<br><br>Yes<br>No

Core Language: US-English Web	Translated Language: US-English Web
**Q09.1c**	**Q09.1c**
Did your injury cause you to be extremely drowsy, or result in broken bones, or severe bleeding?<br><br>Yes<br>No	Did your injury cause you to be extremely drowsy, or result in broken bones, or severe bleeding?<br><br>Yes<br>No
**Q09.2**	**Q09.2**
If Q06a = YES<br>As a result of your **first** attempt, were you injured more seriously than surface scratches or mild nausea?<br><br>Yes<br>No<br><br>If Q06a = NO<br>As a result of the **first** time you tried to hurt yourself, were you injured more seriously than surface scratches or mild nausea?<br><br>Yes<br>No	If Q06a = YES<br>As a result of your **first** attempt, were you injured more seriously than surface scratches or mild nausea?<br><br>Yes<br>No<br><br>If Q06a = NO<br>As a result of the **first** time you tried to hurt yourself, were you injured more seriously than surface scratches or mild nausea?<br><br>Yes<br>No
**Q09.2RB**	**Q09.2RB**
Did this occur within the past B months?<br><br>Yes<br>No	Did this occur within the past B months?<br><br>Yes<br>No
**Q09.2a**	**Q09.2a**
Were you hospitalized for **medical** treatment of the physical injury you suffered? <br>For example, were you comatose from an overdose, or did you suffer extensive blood loss that required a transfusion, or severe damage to your head or a vital organ? <br>If you were hospitalized for psychiatric evaluation, but not for medical treatment of a severe physical injury, answer No.<br><br>Yes<br>No	Were you hospitalized for **medical** treatment of the physical injury you suffered? <br>For example, were you comatose from an overdose, or did you suffer extensive blood loss that required a transfusion, or severe damage to your head or a vital organ? <br>If you were hospitalized for psychiatric evaluation, but not for medical treatment of a severe physical injury, answer No.<br><br>Yes<br>No
**Q09.2b**	**Q09.2b**

Confidential © 2017 Clintec All Rights Reserved

Core Language: US-English Web	Translated Language: US-English Web
Were you injured so severely that you would have died without treatment in an intensive care unit, or did you suffer permanent physical damage from which you will never completely recover, such as paralysis or disfigurement?<br><br>Yes<br>No	Were you injured so severely that you would have died without treatment in an intensive care unit, or did you suffer permanent physical damage from which you will never completely recover, such as paralysis or disfigurement?<br><br>Yes<br>No
Q09.2c	Q09.2c
Did your injury cause you to be extremely drowsy, or result in broken bones, or severe bleeding?<br><br>Yes<br>No	Did your injury cause you to be extremely drowsy, or result in broken bones, or severe bleeding?<br><br>Yes<br>No
Q09.4NRT	Q09.4NRT
Earlier, you indicated that there were three or more times you intended to kill yourself or thought you could have died from what you did. <br>Now we want to know if you suffered any physical injuries each time.	Earlier, you indicated that there were three or more times you intended to kill yourself or thought you could have died from what you did. <br>Now we want to know if you suffered any physical injuries each time.
Q09.4	Q09.4
If Q06a = YES<br>As a result of your **most recent** attempt, were you injured more seriously than surface scratches or mild nausea?<br><br>Yes<br>No<br><br>If Q06a = NO<br>As a result of the **most recent** time you tried to hurt yourself, were you injured more seriously than surface scratches or mild nausea?<br><br>Yes<br>No	If Q06a = YES<br>As a result of your **most recent** attempt, were you injured more seriously than surface scratches or mild nausea?<br><br>Yes<br>No<br><br>If Q06a = NO<br>As a result of the **most recent** time you tried to hurt yourself, were you injured more seriously than surface scratches or mild nausea?<br><br>Yes<br>No
Q09.4RB	Q09.4RB
Did this occur within the past B months?<br><br>Yes<br>No	Did this occur within the past B months?<br><br>Yes<br>No
Q09.4a	Q09.4a

Core Language: US-English Web	Translated Language: US-English Web
Were you hospitalized for **medical** treatment of the physical injury you suffered? <br>For example, were you comatose from an overdose, or did you suffer extensive blood loss that required a transfusion, or severe damage to your head or a vital organ? <br>If you were hospitalized for psychiatric evaluation, but not for medical treatment of a severe physical injury, answer No.<br><br>Yes<br>No	Were you hospitalized for **medical** treatment of the physical injury you suffered? <br>For example, were you comatose from an overdose, or did you suffer extensive blood loss that required a transfusion, or severe damage to your head or a vital organ? <br>If you were hospitalized for psychiatric evaluation, but not for medical treatment of a severe physical injury, answer No.<br><br>Yes<br>No
Q09.4b	Q09.4b
Were you injured so severely that you would have died without treatment in an intensive care unit, or did you suffer permanent physical damage from which you will never completely recover, such as paralysis or disfigurement?<br><br>Yes<br>No	Were you injured so severely that you would have died without treatment in an intensive care unit, or did you suffer permanent physical damage from which you will never completely recover, such as paralysis or disfigurement?<br><br>Yes<br>No
Q09.4c	Q09.4c
Did your injury cause you to be extremely drowsy, or result in broken bones, or severe bleeding?<br><br>Yes<br>No	Did your injury cause you to be extremely drowsy, or result in broken bones, or severe bleeding?<br><br>Yes<br>No
Q09.5	Q09.5
If Q06a = YES<br>As a result of your **first** attempt, were you injured more seriously than surface scratches or mild nausea?<br><br>Yes<br>No<br><br>If Q06a = NO<br>As a result of the **first** time you tried to hurt yourself, were you injured more seriously than surface scratches or mild nausea?<br><br>Yes<br>No	If Q06a = YES<br>As a result of your **first** attempt, were you injured more seriously than surface scratches or mild nausea?<br><br>Yes<br>No<br><br>If Q06a = NO<br>As a result of the **first** time you tried to hurt yourself, were you injured more seriously than surface scratches or mild nausea?<br><br>Yes<br>No
Q09.5a	Q09.5a

Confidential © 2017 Clintec All Rights Reserved

Core Language: US-English Web	Translated Language: US-English Web
Were you hospitalized for **medical** treatment of the physical injury you suffered? <br>For example, were you comatose from an overdose, or did you suffer extensive blood loss that required a transfusion, or severe damage to your head or a vital organ? <br>If you were hospitalized for psychiatric evaluation, but not for medical treatment of a severe physical injury, answer No.<br><br>Yes<br>No	Were you hospitalized for **medical** treatment of the physical injury you suffered? <br>For example, were you comatose from an overdose, or did you suffer extensive blood loss that required a transfusion, or severe damage to your head or a vital organ? <br>If you were hospitalized for psychiatric evaluation, but not for medical treatment of a severe physical injury, answer No.<br><br>Yes<br>No
**Q09.5b**	**Q09.5b**
Were you injured so severely that you would have died without treatment in an intensive care unit, or did you suffer permanent physical damage from which you will never completely recover, such as paralysis or disfigurement?<br><br>Yes<br>No	Were you injured so severely that you would have died without treatment in an intensive care unit, or did you suffer permanent physical damage from which you will never completely recover, such as paralysis or disfigurement?<br><br>Yes<br>No
**Q09.5c**	**Q09.5c**
Did your injury cause you to be extremely drowsy, or result in broken bones, or severe bleeding?<br><br>Yes<br>No	Did your injury cause you to be extremely drowsy, or result in broken bones, or severe bleeding?<br><br>Yes<br>No
**Q09.6**	**Q09.6**
If Q06a = YES<br>As a result of your **most serious** attempt, were you injured more seriously than surface scratches or mild nausea?<br><br>Yes<br>No<br><br>If Q06a = NO<br>As a result of the **most serious** time you tried to hurt yourself, were you injured more seriously than surface scratches or mild nausea?<br><br>Yes<br>No	If Q06a = YES<br>As a result of your **most serious** attempt, were you injured more seriously than surface scratches or mild nausea?<br><br>Yes<br>No<br><br>If Q06a = NO<br>As a result of the **most serious** time you tried to hurt yourself, were you injured more seriously than surface scratches or mild nausea?<br><br>Yes<br>No
**Q09.6a**	**Q09.6a**

Core Language: US-English Web	Translated Language: US-English Web
Were you hospitalized for **medical** treatment of the physical injury you suffered? <br>For example, were you comatose from an overdose, or did you suffer extensive blood loss that required a transfusion, or severe damage to your head or a vital organ? <br>If you were hospitalized for psychiatric evaluation, but not for medical treatment of a severe physical injury, answer No.<br><br>Yes<br>No	Were you hospitalized for **medical** treatment of the physical injury you suffered? <br>For example, were you comatose from an overdose, or did you suffer extensive blood loss that required a transfusion, or severe damage to your head or a vital organ? <br>If you were hospitalized for psychiatric evaluation, but not for medical treatment of a severe physical injury, answer No.<br><br>Yes<br>No
**Q09.6b**	**Q09.6b**
Were you injured so severely that you would have died without treatment in an intensive care unit, or did you suffer permanent physical damage from which you will never completely recover, such as paralysis or disfigurement?<br><br>Yes<br>No	Were you injured so severely that you would have died without treatment in an intensive care unit, or did you suffer permanent physical damage from which you will never completely recover, such as paralysis or disfigurement?<br><br>Yes<br>No
**Q09.6c**	**Q09.6c**
Did your injury cause you to be extremely drowsy, or result in broken bones, or severe bleeding?<br><br>Yes<br>No	Did your injury cause you to be extremely drowsy, or result in broken bones, or severe bleeding?<br><br>Yes<br>No
**Q09.8RB**	**Q09.8RB**
Did this occur within the past B months?<br><br>Yes<br>No	Did this occur within the past B months?<br><br>Yes<br>No
**Q09.8**	**Q09.8**
Although you were not injured **most recently**, how serious could your injuries have been?<br><br>what you did was not likely to cause injury<br>what you did was likely to cause physical injury\\, but probably not death<br>what you did was likely to cause death with or without medical help\\, for example trying to shoot yourself in the head but the gun failed to fire	Although you were not injured **most recently**, how serious could your injuries have been?<br><br>what you did was not likely to cause injury<br>what you did was likely to cause physical injury\\, but probably not death<br>what you did was likely to cause death with or without medical help\\, for example trying to shoot yourself in the head but the gun failed to fire
**Q09.9**	**Q09.9**
Although you were not injured the **first** time, how serious could your injuries have been?	Although you were not injured the **first** time, how serious could your injuries have been?

Confidential © 2017 Clintec All Rights Reserved

Core Language: US-English Web	Translated Language: US-English Web
what you did was not likely to cause injury<br>what you did was likely to cause physical injury, but probably not death<br>what you did was likely to cause death with or without medical help, for example trying to shoot yourself in the head but the gun failed to fire	what you did was not likely to cause injury<br>what you did was likely to cause physical injury\\, but probably not death<br>what you did was likely to cause death with or without medical help\\, for example trying to shoot yourself in the head but the gun failed to fire
Q09.10RB<br>Did this occur within the past B months?<br><br>Yes<br>No	Q09.10RB<br>Did this occur within the past B months?<br><br>Yes<br>No
Q09.10<br>Although you were not injured during the **most serious** time, how serious could your injuries have been?<br><br>what you did was not likely to cause injury<br>what you did was likely to cause physical injury\\, but probably not death<br>what you did was likely to cause death with or without medical help\\, for example trying to shoot yourself in the head but the gun failed to fire	Q09.10<br>Although you were not injured during the **most serious** time, how serious could your injuries have been?<br><br>what you did was not likely to cause injury<br>what you did was likely to cause physical injury\\, but probably not death<br>what you did was likely to cause death with or without medical help\\, for example trying to shoot yourself in the head but the gun failed to fire
Interrupted Attempts	Interrupted Attempts
Q10<br>Has there ever been a time when you started to do something to end your life, but someone or something stopped you before you actually did anything?<br><br>Yes<br>No	Q10<br>Has there ever been a time when you started to do something to end your life, but someone or something stopped you before you actually did anything?<br><br>Yes<br>No
Q10a<br>About how many times have you been stopped from ending your life by someone or something?<br><br>Please enter the number.	Q10a<br>About how many times have you been stopped from ending your life by someone or something?<br><br>Please enter the number.
Q10RB<br>Was the last time you were stopped from trying to end your life by someone or something in the past B months?<br><br>Yes<br>No	Q10RB<br>Was the last time you were stopped from trying to end your life by someone or something in the past B months?<br><br>Yes<br>No
Aborted Attempts	Aborted Attempts

Core Language: US-English Web	Translated Language: US-English Web
Q11<br>Has there been a time when you started to do something to try to end your life, but you stopped yourself before you actually did anything?<br><br>Yes<br>No	Q11<br>Has there been a time when you started to do something to try to end your life, but you stopped yourself before you actually did anything?<br><br>Yes<br>No
Q11a<br>About how many times have you stopped yourself from ending your life?<br><br>Please enter the number.	Q11a<br>About how many times have you stopped yourself from ending your life?<br><br>Please enter the number.
Q11RB<br>Was the last time you stopped yourself from trying to end your life in the past B months?<br><br>Yes<br>No	Q11RB<br>Was the last time you stopped yourself from trying to end your life in the past B months?<br><br>Yes<br>No
Preparatory Acts or Behaviors	Preparatory Acts or Behaviors
Q12NRT<br>Asses prior responses and present introduction:<br>**Other** than the times you have already told us about when you did things intending to kill yourself or thought you might have died, when you started to do something to end your life but someone or something stopped you, and when you started to do something to end your life but stopped yourself,<br><br>**Other** than the times you have already told us about when you did things intending to kill yourself or thought you might have died, and when you started to do something to end your life but stopped yourself,<br><br>**Other** than the times you have already told us about when you did things intending to kill yourself or thought you might have died and when you started to do something to end your life but someone or something stopped you,<br><br>**Other** than the times you have already told us about when you started to do something to end your life but someone or something stopped you and when you started to do something to end your life but stopped yourself,<br><br>**Other** than the times you have already told us about when you did things intending to kill yourself or thought you might have died,	Q12NRT<br>Asses prior responses and present introduction:<br>**Other** than the times you have already told us about when you did things intending to kill yourself or thought you might have died, when you started to do something to end your life but someone or something stopped you, and when you started to do something to end your life but stopped yourself,<br><br>**Other** than the times you have already told us about when you did things intending to kill yourself or thought you might have died, and when you started to do something to end your life but stopped yourself,<br><br>**Other** than the times you have already told us about when you did things intending to kill yourself or thought you might have died and when you started to do something to end your life but someone or something stopped you,<br><br>**Other** than the times you have already told us about when you started to do something to end your life but someone or something stopped you and when you started to do something to end your life but stopped yourself,<br><br>**Other** than the times you have already told us about when you did things intending to kill yourself or thought you might have died,

Confidential © 2017 Clintec All Rights Reserved

Core Language: US-English Web	Translated Language: US-English Web
**Other** than the times you have already told us about when you started to do something to end your life but someone or something stopped you,<br><br>**Other** than the times you have already told us about when you started to do something to end your life but stopped yourself,	**Other** than the times you have already told us about when you started to do something to end your life but someone or something stopped you,<br><br>**Other** than the times you have already told us about when you started to do something to end your life but stopped yourself,
**Q12**	**Q12**
Have you ever taken any steps toward making a suicide attempt or preparing to kill yourself, such as collecting pills, getting a gun, giving valuables away or writing a suicide note?<br><br>Yes<br>No	Have you ever taken any steps toward making a suicide attempt or preparing to kill yourself, such as collecting pills, getting a gun, giving valuables away or writing a suicide note?<br><br>Yes<br>No
**Q12a**	**Q12a**
About how many times?<br><br>Please enter the number.	About how many times?<br><br>Please enter the number.
**Q12RB**	**Q12RB**
Was the last time you took steps toward making a suicide attempt or preparing to kill yourself in the past B months?<br><br>Yes<br>No	Was the last time you took steps toward making a suicide attempt or preparing to kill yourself in the past B months?<br><br>Yes<br>No
**Exit**	**Exit**
You have completed your interview. Thank you and Good bye.	You have completed your interview. Thank you and good-bye.

The electronic Columbia Suicide Severity Rating Scale – Since Last Visit

<u>**eC-SSRS 2.0 Web Script – Since Last Call**</u>

Sheet Name:
en-US-Web

<u>Key:</u>

**NRT** – No response text<br>**Q** - Question	<u>*** NOTES ABOUT THE WEB TEXT:**</u><br>1.) Text between the **and** notations will appear as **bolded** when displayed on the web page.<br>2.) The <br>tags indicates a new paragraph when text is displayed on the web page.<br>3.) The "\\" preceding a comma or a parenthesis is used to properly keep long response phrases together on the web page as a continuous sentence.

<u>Usage Notes:</u>
When the interval between calls exceeds 120 days the number of days since the last call is not repeated to the subject.

Core Language: US-English Web	Translated Language: US-English Web
**Introduction**	**Introduction**
**NRT01**	**NRT01**
The last time you were interviewed about thoughts or actions related to wanting to be dead or killing yourself was [day] [date]. That was [num] days ago. <br>During **this** interview we want you to **only** consider thoughts or actions that have occurred since that date. <br>In answering the following questions, only report your thoughts and actions over the past [num] days or since [day] [date].<br><br>**If days SLC > 120**<br><br>The last time you were interviewed about thoughts or actions related to wanting to be dead or killing yourself was [day] [date]. <br>During **this** interview we want you to **only** consider thoughts or actions that have occurred since that date. <br>In answering the following questions, only report your thoughts and actions since [day] [date].	The last time you were interviewed about thoughts or actions related to wanting to be dead or killing yourself was [day] [date]. That was [num] days ago. <br>During **this** interview we want you to **only** consider thoughts or actions that have occurred since that date. <br>In answering the following questions, only report your thoughts and actions over the past [num] days or since [day] [date].<br><br>**If days SLC > 120**<br><br>The last time you were interviewed about thoughts or actions related to wanting to be dead or killing yourself was [day] [date]. <br>During **this** interview we want you to **only** consider thoughts or actions that have occurred since that date. <br>In answering the following questions, only report your thoughts and actions since [day] [date].
**Passive Suicide Ideation**	**Passive Suicide Ideation**
**Q01**	**Q01**
Since your last interview, have you wished you were dead or wished you could go to sleep and not wake up?<br><br>Yes<br>No<br><br>**Ideation Level = 1**	Since your last interview, have you wished you were dead or wished you could go to sleep and not wake up?<br><br>Yes<br>No<br><br>**Ideation Level = 1**
**Active Suicide Ideation**	**Active Suicide Ideation**
**Q02**	**Q02**

Confidential © 2017 Clintec All Rights Reserved

Core Language: US-English Web	Translated Language: US-English Web
Since your last interview on [day] [date], [num] days ago have you actually had any thoughts of killing yourself?<br><br>If days SLC > 120<br>Since your last interview on [day] [date], have you actually had any thoughts of killing yourself?<br><br>Yes<br>No<br><br>Ideation Level = 2	Since your last interview on [day] [date], [num] days ago have you actually had any thoughts of killing yourself?<br><br>If days SLC > 120<br>Since your last interview on [day] [date], have you actually had any thoughts of killing yourself?<br><br>Yes<br>No<br><br>Ideation Level = 2
Q03<br>Have you thought about how you might do this?<br><br>Yes<br>No<br><br>Ideation Level = 3	Q03<br>Have you thought about how you might do this?<br><br>Yes<br>No<br><br>Ideation Level = 3
Q03.1<br>What way of killing yourself did you think of most often?<br><br>with medication<br>by hanging<br>by jumping<br>with a gun<br>by some other method	Q03.1<br>What way of killing yourself did you think of most often?<br><br>with medication<br>by hanging<br>by jumping<br>with a gun<br>by some other method
Q04<br>Since your last interview, have you had any intention of acting on these thoughts of killing yourself? <br>As opposed to, you have the thoughts, but you definitely would not act on them?<br><br>Yes<br>No<br><br>Ideation Level = 4	Q04<br>Since your last interview, have you had any intention of acting on these thoughts of killing yourself? <br>As opposed to, you have the thoughts, but you definitely would not act on them?<br><br>Yes<br>No<br><br>Ideation Level = 4
Q05<br>Have you started to work out, or actually worked out, the specific details of how to kill yourself since your last interview?	Q05<br>Have you started to work out, or actually worked out, the specific details of how to kill yourself since your last interview?

Core Language: US-English Web	Translated Language: US-English Web
Yes<br>No	Yes<br>No
Q05q<br>Did you actually intend to carry out the details of your plan?<br><br>Yes<br>No<br><br>Ideation Level = 5	Q05q<br>Did you actually intend to carry out the details of your plan?<br><br>Yes<br>No<br><br>Ideation Level = 5
Q05r<br>How did you intend to kill yourself?<br><br>with medication<br>by hanging<br>by jumping<br>with a gun<br>by some other method	Q05r<br>How did you intend to kill yourself?<br><br>with medication<br>by hanging<br>by jumping<br>with a gun<br>by some other method
Ideation Probing	Ideation Probing
Q01aNRT<br>You just indicated that since your last interview, you had wished you were dead or wished that you could go to sleep and not wake up. We want to ask you a few more questions about that. <br>When responding to these questions, we want you to think about the time when these thoughts were most severe in the past [num] days.<br><br>If days SLC > 120<br>You just indicated that since your last interview, you had wished you were dead or wished that you could go to sleep and not wake up. We want to ask you a few more questions about that. <br>When responding to these questions, we want you to think about the time when these thoughts were most severe since your last interview.	Q01aNRT<br>You just indicated that since your last interview, you had wished you were dead or wished that you could go to sleep and not wake up. We want to ask you a few more questions about that. <br>When responding to these questions, we want you to think about the time when these thoughts were most severe in the past [num] days.<br><br>If days SLC > 120<br>You just indicated that since your last interview, you had wished you were dead or wished that you could go to sleep and not wake up. We want to ask you a few more questions about that. <br>When responding to these questions, we want you to think about the time when these thoughts were most severe since your last interview.
Q01a<br>When your wishes to be dead or to go to sleep and not wake up were most severe, how often did the thoughts occur?<br><br>less than once a week<br>about once a week<br>2 to 5 times a week<br>daily or almost daily<br>many times a day	Q01a<br>When your wishes to be dead or to go to sleep and not wake up were most severe, how often did the thoughts occur?<br><br>less than once a week<br>about once a week<br>2 to 5 times a week<br>daily or almost daily<br>many times a day

Confidential © 2017 Clintec All Rights Reserved

Core Language: US-English Web	Translated Language: US-English Web
**Q01b**	**Q01b**
How long did the thoughts last?<br><br>fleeting\\, lasting seconds to minutes<br>less than an hour<br>between 1 and 4 hours<br>between 4 and 8 hours<br>more than 8 hours	How long did the thoughts last?<br><br>fleeting\\, lasting seconds to minutes<br>less than an hour<br>between 1 and 4 hours<br>between 4 and 8 hours<br>more than 8 hours
**Q01c**	**Q01c**
Did you make any attempt to **try** to control these thoughts about wanting to die or going to sleep and not waking up, whether you were successful in controlling them or not?<br><br>Yes<br>No	Did you make any attempt to **try** to control these thoughts about wanting to die or going to sleep and not waking up, whether you were successful in controlling them or not?<br><br>Yes<br>No
**Q01d**	**Q01d**
How easily could you control or stop these thoughts?<br><br>easily controlled these thoughts<br>with a little difficulty<br>with some difficulty<br>with a lot of difficulty<br>unable to control these thoughts	How easily could you control or stop these thoughts?<br><br>easily controlled these thoughts<br>with a little difficulty<br>with some difficulty<br>with a lot of difficulty<br>unable to control these thoughts
**Q01e.1**	**Q01e.1**
Did you think about things like family, religion, or fear of pain or death that might affect your wish to be dead or going to sleep and not waking up?<br><br>Yes<br>No	Did you think about things like family, religion, or fear of pain or death that might affect your wish to be dead or going to sleep and not waking up?<br><br>Yes<br>No
**Q01e**	**Q01e**
Choose one of the following statements that best describes whether anyone or anything did or did not stop you from wishing you were dead or that you could fall asleep and not wake up.<br><br>something definitely stopped you<br>something probably stopped you<br>you are uncertain whether something stopped you<br>something most likely did not stop you<br>something definitely did not stop you	Choose one of the following statements that best describes whether anyone or anything did or did not stop you from wishing you were dead or that you could fall asleep and not wake up.<br><br>something definitely stopped you<br>something probably stopped you<br>you are uncertain whether something stopped you<br>something most likely did not stop you<br>something definitely did not stop you

Core Language: US-English Web	Translated Language: US-English Web
**Q01f.1**	**Q01f.1**
When you thought about wishing to be dead or going to sleep and not waking up, did you have a reason in mind like ending your pain or getting attention or revenge?<br><br>Yes<br>No	When you thought about wishing to be dead or going to sleep and not waking up, did you have a reason in mind like ending your pain or getting attention or revenge?<br><br>Yes<br>No
**Q01f**	**Q01f**
What sort of reasons did you have for thinking about wanting to die? Was it to end the pain or stop the way you were feeling? <br>In other words, you could not go on living with this pain or how you were feeling? Was it to get attention, revenge or a reaction from others? Or both?<br><br>it was completely to get attention\\, revenge or a reaction from others<br>it was mostly to get attention\\, revenge or a reaction from others<br>equally to get attention\\, revenge or a reaction from others and to end or stop the pain<br>mostly to end or stop the pain \\(that is\\, you could not go on living with the pain or how you were feeling\\)<br>completely to end or stop the pain	What sort of reasons did you have for thinking about wanting to die? Was it to end the pain or stop the way you were feeling? <br>In other words, you could not go on living with this pain or how you were feeling? Was it to get attention, revenge or a reaction from others? Or both?<br><br>it was completely to get attention\\, revenge or a reaction from others<br>it was mostly to get attention\\, revenge or a reaction from others<br>equally to get attention\\, revenge or a reaction from others and to end or stop the pain<br>mostly to end or stop the pain \\(that is\\, you could not go on living with the pain or how you were feeling\\)<br>completely to end or stop the pain
**Q02aNRT**	**Q02aNRT**
You indicated before that since your last interview you had thought of killing yourself. We want to ask you a few more questions about that. <br>When responding to these questions, we want you to think about the time you were feeling most suicidal in the past [num] days.<br><br>If days SLC > 120<br><br>You indicated before that since your last interview you had thought of killing yourself. We want to ask you a few more questions about that. <br>When responding to these questions, we want you to think about the time you were feeling most suicidal since your last interview.	You indicated before that since your last interview you had thought of killing yourself. We want to ask you a few more questions about that. <br>When responding to these questions, we want you to think about the time you were feeling most suicidal in the past [num] days.<br><br>If days SLC > 120<br><br>You indicated before that since your last interview you had thought of killing yourself. We want to ask you a few more questions about that. <br>When responding to these questions, we want you to think about the time you were feeling most suicidal since your last interview.
**Q02a**	**Q02a**
When you were feeling most suicidal, how often did you think of killing yourself?<br><br>less than once a week<br>about once a week<br>2 to 5 times a week<br>daily or almost daily<br>many times a day	When you were feeling most suicidal, how often did you think of killing yourself?<br><br>less than once a week<br>about once a week<br>2 to 5 times a week<br>daily or almost daily<br>many times a day
**Q02b**	**Q02b**

Confidential © 2017 Clintec All Rights Reserved

Core Language: US-English Web	Translated Language: US-English Web
How long did these thoughts of killing yourself last?<br><br>fleeting\\, lasting seconds to minutes<br>less than an hour<br>between 1 and 4 hours<br>between 4 and 8 hours<br>more than 8 hours	How long did these thoughts of killing yourself last?<br><br>fleeting\\, lasting seconds to minutes<br>less than an hour<br>between 1 and 4 hours<br>between 4 and 8 hours<br>more than 8 hours
Q02c	Q02c
Did you make any attempt to **try** to control or stop these thoughts, whether you were successful in controlling them or not?<br><br>Yes<br>No	Did you make any attempt to **try** to control or stop these thoughts, whether you were successful in controlling them or not?<br><br>Yes<br>No
Q02d	Q02d
How easily could you control or stop thinking about killing yourself?<br><br>easily controlled these thoughts<br>with a little difficulty<br>with some difficulty<br>with a lot of difficulty<br>unable to control these thoughts	How easily could you control or stop thinking about killing yourself?<br><br>easily controlled these thoughts<br>with a little difficulty<br>with some difficulty<br>with a lot of difficulty<br>unable to control these thoughts
Q02e.1	Q02e.1
Did you think about things like family, religion, or fear of pain or death that might affect your decision about killing yourself?<br><br>Yes<br>No	Did you think about things like family, religion, or fear of pain or death that might affect your decision about killing yourself?<br><br>Yes<br>No
Q02e	Q02e
Choose one of the following statements that best describes whether anyone or anything did or did not stop you from acting on your thoughts of committing suicide.<br><br>something definitely stopped you<br>something probably stopped you<br>you are uncertain whether something stopped you<br>something most likely did not stop you<br>something definitely did not stop you	Choose one of the following statements that best describes whether anyone or anything did or did not stop you from acting on your thoughts of committing suicide.<br><br>something definitely stopped you<br>something probably stopped you<br>you are uncertain whether something stopped you<br>something most likely did not stop you<br>something definitely did not stop you
Q02f.1	Q02f.1

Core Language: US-English Web	Translated Language: US-English Web
When you thought about killing yourself, did you have a reason in mind like ending your pain or getting attention or revenge?<br><br>Yes<br>No	When you thought about killing yourself, did you have a reason in mind like ending your pain or getting attention or revenge?<br><br>Yes<br>No
Q02f	Q02f
What sort of reasons did you have for thinking about wanting to kill yourself? Was it to end the pain or stop the way you were feeling? <br>In other words, you could not go on living with this pain or how you were feeling? Or was it to get attention, revenge or a reaction from others? Or both?<br><br>it was completely to get attention\\, revenge or a reaction from others<br>it was mostly to get attention\\, revenge or a reaction from others<br>equally to get attention\\, revenge or a reaction from others and to end or stop the pain<br>mostly to end or stop the pain \\(that is\\, you could not go on living with the pain or how you were feeling\\)<br>completely to end or stop the pain	What sort of reasons did you have for thinking about wanting to kill yourself? Was it to end the pain or stop the way you were feeling? <br>In other words, you could not go on living with this pain or how you were feeling? Or was it to get attention, revenge or a reaction from others? Or both?<br><br>it was completely to get attention\\, revenge or a reaction from others<br>it was mostly to get attention\\, revenge or a reaction from others<br>equally to get attention\\, revenge or a reaction from others and to end or stop the pain<br>mostly to end or stop the pain \\(that is\\, you could not go on living with the pain or how you were feeling\\)<br>completely to end or stop the pain
Q03aNRT	Q03aNRT
You indicated before that since your last interview you had thought about how you might kill yourself. We want to ask you a few more questions about that. <br>When responding to these questions, we want you to think about the time you were feeling the most suicidal in the past [num] days.<br><br>If days SLC > 120<br>You indicated before that since your last interview you had thought about how you might kill yourself. We want to ask you a few more questions about that. <br>When responding to these questions, we want you to think about the time you were feeling the most suicidal since your last interview.	You indicated before that since your last interview you had thought about how you might kill yourself. We want to ask you a few more questions about that. <br>When responding to these questions, we want you to think about the time you were feeling the most suicidal in the past [num] days.<br><br>If days SLC > 120<br>You indicated before that since your last interview you had thought about how you might kill yourself. We want to ask you a few more questions about that. <br>When responding to these questions, we want you to think about the time you were feeling the most suicidal since your last interview.
Q03a	Q03a
When you were feeling most suicidal, how often did you think about how you might kill yourself?<br><br>less than once a week<br>about once a week<br>2 to 5 times a week<br>daily or almost daily<br>many times a day	When you were feeling most suicidal, how often did you think about how you might kill yourself?<br><br>less than once a week<br>about once a week<br>2 to 5 times a week<br>daily or almost daily<br>many times a day
Q03b	Q03b
When you had these thoughts, how long did they last?	When you had these thoughts, how long did they last?

Confidential © 2017 Clintec All Rights Reserved

Core Language: US-English Web	Translated Language: US-English Web
fleeting\\, lasting seconds to minutes<br>less than an hour<br>between 1 and 4 hours<br>between 4 and 8 hours<br>more than 8 hours	fleeting\\, lasting seconds to minutes<br>less than an hour<br>between 1 and 4 hours<br>between 4 and 8 hours<br>more than 8 hours
Q03c<br>Did you make any attempt to **try** to control or stop these thoughts, whether you were successful in controlling them or not?<br><br>Yes<br>No	Q03c<br>Did you make any attempt to **try** to control or stop these thoughts, whether you were successful in controlling them or not?<br><br>Yes<br>No
Q03d<br>How easily could you control or stop thinking about how you might kill yourself?<br><br>easily controlled these thoughts<br>with a little difficulty<br>with some difficulty<br>with a lot of difficulty<br>unable to control these thoughts	Q03d<br>How easily could you control or stop thinking about how you might kill yourself?<br><br>easily controlled these thoughts<br>with a little difficulty<br>with some difficulty<br>with a lot of difficulty<br>unable to control these thoughts
Q03e.1<br>Did you think about things like family, religion, or fear of pain or death that might affect your decision about killing yourself?<br><br>Yes<br>No	Q03e.1<br>Did you think about things like family, religion, or fear of pain or death that might affect your decision about killing yourself?<br><br>Yes<br>No
Q03e<br>Choose one of the following statements that best describes whether anyone or anything did or did not stop you from acting on your thoughts of committing suicide.<br><br>something definitely stopped you<br>something probably stopped you<br>you are uncertain whether something stopped you<br>something most likely did not stop you<br>something definitely did not stop you	Q03e<br>Choose one of the following statements that best describes whether anyone or anything did or did not stop you from acting on your thoughts of committing suicide.<br><br>something definitely stopped you<br>something probably stopped you<br>you are uncertain whether something stopped you<br>something most likely did not stop you<br>something definitely did not stop you
Q03f.1<br>When you thought about killing yourself, did you have a reason in mind like ending your pain or getting attention or revenge?<br><br>Yes<br>No	Q03f.1<br>When you thought about killing yourself, did you have a reason in mind like ending your pain or getting attention or revenge?<br><br>Yes<br>No
Q03f<br>What sort of reasons did you have for thinking about wanting to kill yourself? Was it to end the pain or stop the way you were feeling? <br>In other words, you could not go on living with this pain or how you were feeling? Or was it to get attention, revenge or a reaction from others? Or both?<br><br>it was completely to get attention\\, revenge or a reaction from others<br>it was mostly to get attention\\, revenge or a reaction from others<br>equally to get attention\\, revenge or a reaction from others and to end or stop the pain<br>mostly to end or stop the pain \\(that is\\, you could not go on living with the pain or how you were feeling\\)<br>completely to end or stop the pain	Q03f<br>What sort of reasons did you have for thinking about wanting to kill yourself? Was it to end the pain or stop the way you were feeling? <br>In other words, you could not go on living with this pain or how you were feeling? Or was it to get attention, revenge or a reaction from others? Or both?<br><br>it was completely to get attention\\, revenge or a reaction from others<br>it was mostly to get attention\\, revenge or a reaction from others<br>equally to get attention\\, revenge or a reaction from others and to end or stop the pain<br>mostly to end or stop the pain \\(that is\\, you could not go on living with the pain or how you were feeling\\)<br>completely to end or stop the pain
Q04aNRT<br>You indicated before that since your last interview you thought about killing yourself and that you had some intention of acting on these thoughts. We want to ask you a few more questions about that. <br>When responding to these questions, we want you to think about the time you were feeling the most suicidal in the past [num] days.<br><br>If days SLC > 120<br>You indicated before that since your last interview you thought about killing yourself and that you had some intention of acting on these thoughts. We want to ask you a few more questions about that. <br>When responding to these questions, we want you to think about the time you were feeling the most suicidal since your last interview.	Q04aNRT<br>You indicated before that since your last interview you thought about killing yourself and that you had some intention of acting on these thoughts. We want to ask you a few more questions about that. <br>When responding to these questions, we want you to think about the time you were feeling the most suicidal in the past [num] days.<br><br>If days SLC > 120<br>You indicated before that since your last interview you thought about killing yourself and that you had some intention of acting on these thoughts. We want to ask you a few more questions about that. <br>When responding to these questions, we want you to think about the time you were feeling the most suicidal since your last interview.
Q04a<br>When you were feeling most suicidal and had some intention of acting on those thoughts of killing yourself, how often did those thoughts occur?<br><br>less than once a week<br>about once a week<br>2 to 5 times a week<br>daily or almost daily<br>many times a day	Q04a<br>When you were feeling most suicidal and had some intention of acting on those thoughts of killing yourself, how often did those thoughts occur?<br><br>less than once a week<br>about once a week<br>2 to 5 times a week<br>daily or almost daily<br>many times a day
Q04b<br>How long did the thoughts last?<br><br>fleeting\\, lasting seconds to minutes	Q04b<br>How long did the thoughts last?<br><br>fleeting\\, lasting seconds to minutes

Confidential © 2017 Clintec All Rights Reserved

Core Language: US-English Web	Translated Language: US-English Web
less than an hour<br>between 1 and 4 hours<br>between 4 and 8 hours<br>more than 8 hours	less than an hour<br>between 1 and 4 hours<br>between 4 and 8 hours<br>more than 8 hours
**Q04c**	**Q04c**
Did you make any attempt to **try** to control or stop these thoughts about actually killing yourself, whether you were successful in controlling them or not?<br><br>Yes<br>No	Did you make any attempt to **try** to control or stop these thoughts about actually killing yourself, whether you were successful in controlling them or not?<br><br>Yes<br>No
**Q04d**	**Q04d**
How easily could you control or stop thinking about killing yourself?<br><br>easily controlled these thoughts<br>with a little difficulty<br>with some difficulty<br>with a lot of difficulty<br>unable to control these thoughts	How easily could you control or stop thinking about killing yourself?<br><br>easily controlled these thoughts<br>with a little difficulty<br>with some difficulty<br>with a lot of difficulty<br>unable to control these thoughts
**Q04e.1**	**Q04e.1**
Did you think about things like family, religion, or fear of pain or death that might affect your decision about killing yourself?<br><br>Yes<br>No	Did you think about things like family, religion, or fear of pain or death that might affect your decision about killing yourself?<br><br>Yes<br>No
**Q04e**	**Q04e**
Choose one of the following statements that best describes whether anyone or anything did or did not stop you from acting on your thoughts of committing suicide.<br><br>something definitely stopped you<br>something probably stopped you<br>you are uncertain whether something stopped you<br>something most likely did not stop you<br>something definitely did not stop you	Choose one of the following statements that best describes whether anyone or anything did or did not stop you from acting on your thoughts of committing suicide.<br><br>something definitely stopped you<br>something probably stopped you<br>you are uncertain whether something stopped you<br>something most likely did not stop you<br>something definitely did not stop you
**Q04f.1**	**Q04f.1**
When you thought about killing yourself, did you have a reason in mind like ending your pain or getting attention or revenge?	When you thought about killing yourself, did you have a reason in mind like ending your pain or getting attention or revenge?

Core Language: US-English Web	Translated Language: US-English Web
Yes<br>No	Yes<br>No
**Q04f**	**Q04f**
What sort of reasons did you have for thinking about wanting to kill yourself? Was it to end the pain or stop the way you were feeling? <br>In other words, you could not go on living with this pain or how you were feeling? Or was it to get attention, revenge or a reaction from others? Or both?<br><br>it was completely to get attention\\, revenge or a reaction from others<br>it was mostly to get attention\\, revenge or a reaction from others<br>equally to get attention\\, revenge or a reaction from others and to end or stop the pain<br>mostly to end or stop the pain \\(that is\\, you could not go on living with the pain or how you were feeling\\)<br>completely to end or stop the pain	What sort of reasons did you have for thinking about wanting to kill yourself? Was it to end the pain or stop the way you were feeling? <br>In other words, you could not go on living with this pain or how you were feeling? Or was it to get attention, revenge or a reaction from others? Or both?<br><br>it was completely to get attention\\, revenge or a reaction from others<br>it was mostly to get attention\\, revenge or a reaction from others<br>equally to get attention\\, revenge or a reaction from others and to end or stop the pain<br>mostly to end or stop the pain \\(that is\\, you could not go on living with the pain or how you were feeling\\)<br>completely to end or stop the pain
**Q05aNRT**	**Q05aNRT**
You indicated before that since your last interview you had started working on plans or had actually worked out the details of how to kill yourself and had some intention to act on them. We want to ask you a few more questions about that. <br>When responding to these questions, we want you to think about the time you were feeling the most suicidal in the past [num] days.<br><br>If days SLC > 120<br><br>You indicated before that since your last interview you had started working on plans or had actually worked out the details of how to kill yourself and had some intention to act on them. We want to ask you a few more questions about that. <br>When responding to these questions, we want you to think about the time you were feeling the most suicidal since your last interview.	You indicated before that since your last interview you had started working on plans or had actually worked out the details of how to kill yourself and had some intention to act on them. We want to ask you a few more questions about that. <br>When responding to these questions, we want you to think about the time you were feeling the most suicidal in the past [num] days.<br><br>If days SLC > 120<br><br>You indicated before that since your last interview you had started working on plans or had actually worked out the details of how to kill yourself and had some intention to act on them. We want to ask you a few more questions about that. <br>When responding to these questions, we want you to think about the time you were feeling the most suicidal since your last interview.
**Q05a**	**Q05a**
When you were feeling the most suicidal and started planning or worked out details of how to kill yourself, how often did you think about killing yourself?<br><br>less than once a week<br>about once a week<br>2 to 5 times a week<br>daily or almost daily<br>many times a day	When you were feeling the most suicidal and started planning or worked out details of how to kill yourself, how often did you think about killing yourself?<br><br>less than once a week<br>about once a week<br>2 to 5 times a week<br>daily or almost daily<br>many times a day
**Q05b**	**Q05b**
How long did the thoughts last?	How long did the thoughts last?

Confidential © 2017 Clintec All Rights Reserved

Core Language: US-English Web	Translated Language: US-English Web
fleeting\\, lasting seconds to minutes<br>less than an hour<br>between 1 and 4 hours<br>between 4 and 8 hours<br>more than 8 hours	fleeting\\, lasting seconds to minutes<br>less than an hour<br>between 1 and 4 hours<br>between 4 and 8 hours<br>more than 8 hours
Q05c	Q05c
Did you make any attempt to **try** to control or stop these thoughts about actually killing yourself, whether you were successful in controlling them or not?<br><br>Yes<br>No	Did you make any attempt to **try** to control or stop these thoughts about actually killing yourself, whether you were successful in controlling them or not?<br><br>Yes<br>No
Q05d	Q05d
How easily could you control or stop thinking about killing yourself?<br><br>easily controlled these thoughts<br>with a little difficulty<br>with some difficulty<br>with a lot of difficulty<br>unable to control these thoughts	How easily could you control or stop thinking about killing yourself?<br><br>easily controlled these thoughts<br>with a little difficulty<br>with some difficulty<br>with a lot of difficulty<br>unable to control these thoughts
Q05e.1	Q05e.1
Did you think about things like family, religion, or fear of pain or death that might affect your decision about killing yourself?<br><br>Yes<br>No	Did you think about things like family, religion, or fear of pain or death that might affect your decision about killing yourself?<br><br>Yes<br>No
Q05e	Q05e
Choose one of the following statements that best describes whether anyone or anything did or did not stop you from acting on your thoughts of committing suicide.<br><br>something definitely stopped you<br>something probably stopped you<br>you are uncertain whether something stopped you<br>something most likely did not stop you<br>something definitely did not stop you	Choose one of the following statements that best describes whether anyone or anything did or did not stop you from acting on your thoughts of committing suicide.<br><br>something definitely stopped you<br>something probably stopped you<br>you are uncertain whether something stopped you<br>something most likely did not stop you<br>something definitely did not stop you
Q05f.1	Q05f.1
When you thought about killing yourself, did you have a reason in mind like ending your pain or getting attention or revenge?	When you thought about killing yourself, did you have a reason in mind like ending your pain or getting attention or revenge?

Core Language: US-English Web	Translated Language: US-English Web
Yes<br>No	Yes<br>No
Q05f	Q05f
What sort of reasons did you have for thinking about wanting to kill yourself? Was it to end the pain or stop the way you were feeling? <br>In other words, you could not go on living with this pain or how you were feeling? Or was it to get attention, revenge or a reaction from others? Or both?<br><br>it was completely to get attention\\, revenge or a reaction from others<br>it was mostly to get attention\\, revenge or a reaction from others<br>equally to get attention\\, revenge or a reaction from others and to end or stop the pain<br>mostly to end or stop the pain \\(that is\\, you could not go on living with the pain or how you were feeling\\)<br>completely to end or stop the pain	What sort of reasons did you have for thinking about wanting to kill yourself? Was it to end the pain or stop the way you were feeling? <br>In other words, you could not go on living with this pain or how you were feeling? Or was it to get attention, revenge or a reaction from others? Or both?<br><br>it was completely to get attention\\, revenge or a reaction from others<br>it was mostly to get attention\\, revenge or a reaction from others<br>equally to get attention\\, revenge or a reaction from others and to end or stop the pain<br>mostly to end or stop the pain \\(that is\\, you could not go on living with the pain or how you were feeling\\)<br>completely to end or stop the pain
Midpoint Transition (NRT02)	Midpoint Transition (NRT02)
We are almost finished. <br>So far we have been asking about thoughts and feelings you may have had. <br>Now we would like to know about things you may have done to try to hurt yourself since your last interview.	We are almost finished. <br>So far we have been asking about thoughts and feelings you may have had. <br>Now we would like to know about things you may have done to try to hurt yourself since your last interview.
Suicidal Behavior	Suicidal Behavior
Q06a	Q06a
Since your last interview on [day] [date] have you made a suicide attempt?<br><br>Yes<br>No	Since your last interview on [day] [date] have you made a suicide attempt?<br><br>Yes<br>No
Q06b	Q06b
Use the number keys to enter the number of suicide attempts you have made since your last interview.	Use the number keys to enter the number of suicide attempts you have made since your last interview.
Q06cNRT01	Q06cNRT01
If attempts >= 3<br><br>Consider your most recent attempt, your first attempt, and your most serious attempt separately.	If attempts >= 3<br><br>Consider your most recent attempt, your first attempt, and your most serious attempt separately.
Q06cNRT02	Q06cNRT02
If attempts = 2<br>Consider your most recent attempt and your first attempt separately	If attempts = 2<br>Consider your most recent attempt and your first attempt separately
Q06c	Q06c
If loop = 1	If loop = 1

Confidential © 2017 Clintec All Rights Reserved

Core Language: US-English Web	Translated Language: US-English Web
When you made your most recent attempt, were you trying to end your life?<br><br>Yes<br>No<br><br>If loop = 2<br>When you made your first attempt, were you trying to end your life?<br><br>Yes<br>No<br><br>If loop = 3<br>When you made your most serious attempt, were you trying to end your life?<br><br>Yes<br>No	When you made your most recent attempt, were you trying to end your life?<br><br>Yes<br>No<br><br>If loop = 2<br>When you made your first attempt, were you trying to end your life?<br><br>Yes<br>No<br><br>If loop = 3<br>When you made your most serious attempt, were you trying to end your life?<br><br>Yes<br>No
Q06e	Q06e
Did you think it was possible that you could have died from what you did?<br><br>Yes<br>No	Did you think it was possible that you could have died from what you did?<br><br>Yes<br>No
Q06d	Q06d
So then<br>you wanted to die\\, even a little\\, when you did this<br>you did it purely for other reasons\\, like to relieve stress\\, feel better\\, get sympathy\\, or get something else to happen to you\\, without any intention of killing yourself	So then<br>you wanted to die\\, even a little\\, when you did this<br>you did it purely for other reasons\\, like to relieve stress\\, feel better\\, get sympathy\\, or get something else to happen to you\\, without any intention of killing yourself
Q07a	Q07a
Since your last interview, have you done anything to intentionally hurt or harm yourself?<br><br>Yes<br>No	Since your last interview, have you done anything to intentionally hurt or harm yourself?<br><br>Yes<br>No
Q07b	Q07b
Use the number keys to enter the number of times you have intentionally hurt or harmed yourself since your last interview. If you cannot remember the exact number, enter your best estimate.	Use the number keys to enter the number of times you have intentionally hurt or harmed yourself since your last interview. If you cannot remember the exact number, enter your best estimate.
Q07cNRT01	Q07cNRT01
Just consider the three most recent times you have intentionally harmed or hurt yourself.	Just consider the three most recent times you have intentionally harmed or hurt yourself.

Core Language: US-English Web	Translated Language: US-English Web
Q07c_Attempt	Q07c_Attempt
If loop = 1<br><br>Think about the time you intentionally hurt or harmed yourself most recently.<br><br>If loop > 1<br><br>Consider the time you hurt or harmed yourself before that	If loop = 1<br><br>Think about the time you intentionally hurt or harmed yourself most recently.<br><br>If loop > 1<br><br>Consider the time you hurt or harmed yourself before that
Q07c	Q07c
Were you trying to end your life?<br><br>Yes<br>No	Were you trying to end your life?<br><br>Yes<br>No
Q07e	Q07e
Did you think it was possible that you could have died from what you did?<br><br>Yes<br>No	Did you think it was possible that you could have died from what you did?<br><br>Yes<br>No
Q07d	Q07d
So then<br>you wanted to die\\, even a little\\, when you did this<br>you did it purely for other reasons\\, like to relieve stress\\, feel better\\, get sympathy\\, or get something else to happen to you\\, without any intention of killing yourself	So then<br>you wanted to die\\, even a little\\, when you did this<br>you did it purely for other reasons\\, like to relieve stress\\, feel better\\, get sympathy\\, or get something else to happen to you\\, without any intention of killing yourself
Q08a	Q08a
Since your last interview, have you done anything dangerous where you could have died?<br><br>Yes<br>No	Since your last interview, have you done anything dangerous where you could have died?<br><br>Yes<br>No
Q08b	Q08b
Use the number keys to enter the number of times you have done dangerous activities where you could have died in the past [num] days.<br><br>If days SLC > 120<br>Use the number keys to enter the number of times you have done dangerous activities where you could have died since your last interview.	Use the number keys to enter the number of times you have done dangerous activities where you could have died in the past [num] days.<br><br>If days SLC > 120<br>Use the number keys to enter the number of times you have done dangerous activities where you could have died since your last interview.

Confidential © 2017 Clintec All Rights Reserved

Core Language: US-English Web	Translated Language: US-English Web
Q08c_NRT01<br>Just consider the three most recent times you have done something dangerous where you could have died.	Q08c_NRT01<br>Just consider the three most recent times you have done something dangerous where you could have died.
Q08c_Attempt<br>If loop = 1<br>Think about the most recent time you did a dangerous activity where you could have died<br><br>If loop > 1<br>Consider the time you did something dangerous before that	Q08c_Attempt<br>If loop = 1<br>Think about the most recent time you did a dangerous activity where you could have died<br><br>If loop > 1<br>Consider the time you did something dangerous before that
Q08c_1<br>Were you trying to harm yourself when you did this?<br><br>Yes<br>No	Q08c_1<br>Were you trying to harm yourself when you did this?<br><br>Yes<br>No
Q08c<br>Were you trying to end your life?<br><br>Yes<br>No	Q08c<br>Were you trying to end your life?<br><br>Yes<br>No
Q08d<br>So then<br>you wanted to die\\, even a little\\, when you did this<br>you did it purely for other reasons\\, like to relieve stress\\, feel better\\, get sympathy\\, or get something else to happen to you\\, without any intention of killing yourself	Q08d<br>So then<br>you wanted to die\\, even a little\\, when you did this<br>you did it purely for other reasons\\, like to relieve stress\\, feel better\\, get sympathy\\, or get something else to happen to you\\, without any intention of killing yourself
**Lethality**	**Lethality**
Q09<br>If Q06a = YES<br>As a result of your **most serious** attempt since your last interview, were you injured more seriously than surface scratches or mild nausea?<br><br>Yes<br>No<br><br>If Q06a = NO<br>As a result of the **most serious** time you tried to hurt yourself since your last interview, were you injured more seriously than surface scratches or mild nausea?<br><br>Yes<br>No	Q09<br>If Q06a = YES<br>As a result of your **most serious** attempt since your last interview, were you injured more seriously than surface scratches or mild nausea?<br><br>Yes<br>No<br><br>If Q06a = NO<br>As a result of the **most serious** time you tried to hurt yourself since your last interview, were you injured more seriously than surface scratches or mild nausea?<br><br>Yes<br>No
Q09a<br>Were you hospitalized for **medical** treatment of the physical injury you suffered? <br>For example, were you comatose from an overdose, or did you suffer extensive blood loss that required a transfusion, or severe damage to your head or a vital organ? <br>If you were hospitalized for psychiatric evaluation, but not for medical treatment of a severe physical injury, answer No.<br><br>Yes<br>No	Q09a<br>Were you hospitalized for **medical** treatment of the physical injury you suffered? <br>For example, were you comatose from an overdose, or did you suffer extensive blood loss that required a transfusion, or severe damage to your head or a vital organ? <br>If you were hospitalized for psychiatric evaluation, but not for medical treatment of a severe physical injury, answer No.<br><br>Yes<br>No
Q09b<br>Were you injured so severely that you would have died without treatment in an intensive care unit, or did you suffer permanent physical damage from which you will never completely recover, such as paralysis or disfigurement?<br><br>Yes<br>No	Q09b<br>Were you injured so severely that you would have died without treatment in an intensive care unit, or did you suffer permanent physical damage from which you will never completely recover, such as paralysis or disfigurement?<br><br>Yes<br>No
Q09c<br>Did your injury cause you to be extremely drowsy, or result in broken bones, or severe bleeding?<br><br>Yes<br>No	Q09c<br>Did your injury cause you to be extremely drowsy, or result in broken bones, or severe bleeding?<br><br>Yes<br>No
Q09.10<br>Although you were not injured during the **most serious** time, how serious could your injuries have been?<br><br>what you did was not likely to cause injury<br>what you did was likely to cause physical injury\\, but probably not death<br>what you did was likely to cause death with or without medical help\\, for example trying to shoot yourself in the head but the gun failed to fire	Q09.10<br>Although you were not injured during the **most serious** time, how serious could your injuries have been?<br><br>what you did was not likely to cause injury<br>what you did was likely to cause physical injury\\, but probably not death<br>what you did was likely to cause death with or without medical help\\, for example trying to shoot yourself in the head but the gun failed to fire
**Interrupted Attempts**	**Interrupted Attempts**
Q10	Q10

Confidential © 2017 Clintec All Rights Reserved

Core Language: US-English Web	Translated Language: US-English Web
Since your last interview, was there a time when you started to do something to end your life, but someone or something stopped you before you actually did anything?<br><br>Yes<br>No	Since your last interview, was there a time when you started to do something to end your life, but someone or something stopped you before you actually did anything?<br><br>Yes<br>No
Q10a	Q10a
About how many times have you been stopped from ending your life by someone or something since your last interview?<br><br>Please enter the number.	About how many times have you been stopped from ending your life by someone or something since your last interview?<br><br>Please enter the number.
Aborted Attempts	Aborted Attempts
Q11	Q11
Since your last interview, has there been a time when you started to do something to try to end your life, but you stopped yourself before you actually did anything?<br><br>Yes<br>No	Since your last interview, has there been a time when you started to do something to try to end your life, but you stopped yourself before you actually did anything?<br><br>Yes<br>No
Q11a	Q11a
About how many times have you stopped yourself from ending your life in the last [num] days, since your last interview?<br><br>If days SLC > 120<br><br>About how many times have you stopped yourself from ending your life since your last interview?<br><br>Please enter the number.	About how many times have you stopped yourself from ending your life in the last [num] days, since your last interview?<br><br>If days SLC > 120<br><br>About how many times have you stopped yourself from ending your life since your last interview?<br><br>Please enter the number.
Preparatory Acts or Behaviors	Preparatory Acts or Behaviors
Q12NRT	Q12NRT
Asses prior responses and present introduction:<br>**Other** than the times you have already told us about since your last interview when you did things intending to kill yourself or thought you might have died, when you started to do something to end your life but someone or something stopped you, and when you started to do something to end your life but stopped yourself,<br><br>**Other** than the times you have already told us about since your last interview when you did things intending to kill yourself or thought you might have died, and when you started to do something to end your life but stopped yourself,	Asses prior responses and present introduction:<br>**Other** than the times you have already told us about since your last interview when you did things intending to kill yourself or thought you might have died, when you started to do something to end your life but someone or something stopped you, and when you started to do something to end your life but stopped yourself,<br><br>**Other** than the times you have already told us about since your last interview when you did things intending to kill yourself or thought you might have died, and when you started to do something to end your life but stopped yourself,

Core Language: US-English Web	Translated Language: US-English Web
**Other** than the times you have already told us about since your last interview when you did things intending to kill yourself or thought you might have died and when you started to do something to end your life but someone or something stopped you<br><br>**Other** than the times you have already told us about since your last interview when you started to do something to end your life but someone or something stopped you and when you started to do something to end your life but stopped yourself,<br><br>**Other** than the times you have already told us about since your last interview when you did things intending to kill yourself or thought you might have died,<br><br>**Other** than the times you have already told us about since your last interview when you started to do something to end your life but someone or something stopped you,<br><br>**Other** than the times you have already told us about since your last interview when you started to do something to end your life but stopped yourself,	**Other** than the times you have already told us about since your last interview when you did things intending to kill yourself or thought you might have died and when you started to do something to end your life but someone or something stopped you<br><br>**Other** than the times you have already told us about since your last interview when you started to do something to end your life but someone or something stopped you and when you started to do something to end your life but stopped yourself,<br><br>**Other** than the times you have already told us about since your last interview when you did things intending to kill yourself or thought you might have died,<br><br>**Other** than the times you have already told us about since your last interview when you started to do something to end your life but someone or something stopped you,<br><br>**Other** than the times you have already told us about since your last interview when you started to do something to end your life but stopped yourself,
Q12	Q12
Have you taken any steps toward making a suicide attempt or preparing to kill yourself, such as collecting pills, getting a gun, giving valuables away or writing a suicide note?<br><br>Yes<br>No	Have you taken any steps toward making a suicide attempt or preparing to kill yourself, such as collecting pills, getting a gun, giving valuables away or writing a suicide note?<br><br>Yes<br>No
Q12a	Q12a
About how many times?<br><br>Please enter the number.	About how many times?<br><br>Please enter the number.
Exit	Exit
You have completed your interview. Thank you and Good bye.	You have completed your interview. Thank you and good-bye.

Confidential © 2017 Clintec All Rights Reserved

13.10 Appendix 10 BPIC-SS

Bladder Pain/ Interstitial Cystitis Symptom Score (BPIC-SS)

When answering the following questions, please think about the PAST 7 DAYS

Score column to be completed by staff.

	Never	Rarely	Sometimes	Most of the Time	Always	Score
1. In the past 7 days when you urinated, how often was it because of pain in your bladder?						
2. In the past 7 days, how often did you still feel the need to urinate just after you urinated?						
3. In the past 7 days, how often did you urinate to **avoid pain** in your **bladder** from **getting worse**?						
4. In the past 7 days, how often did you have a feeling of **pressure** in your **bladder**?						
5. In the past 7 days, how often did you have **pain** in your **bladder**?						

	Not at all	A little	Some-what	Moder-ately	A great deal	Score
6. In the past 7 days, how **bothered** were you by **frequent urination during the daytime**?						
7. In the past 7 days how **bothered** were you by **having to get up** during the **night** to urinate?						

8. Select the number that best describes your worst bladder pain in the past 7 days											Score
No bladder pain										Worst possible bladder pain	
0	1	2	3	4	5	6	7	8	9	10	

Add the scores for each question together to give a total BPIC-SS score.

**Total Score:**	

Total score ranges from 0 - 38. A total score can only be calculated if ALL questions are completed by the patient.

Confidential © 2017 Clintec All Rights Reserved

13.11 Appendix 11 Verbal Fluency (Category and letter)

Verbal fluency tests are psychological tests in which participants have to produce as many words as possible from a category in a given time (usually 60 seconds). This category can be semantic, including objects such as animals or fruits, or phonemic, including words beginning with a specified letter, such as p, for example. Although the most common performance measure is the total number of words, other analyses such as number of repetitions, number and length of clusters of words from the same semantic or phonetic subcategory, or number of switches to other categories can be carried out.

Instructions for the Category Fluency test:

Say: ''I'm going to give you a category and ask you to name all the different examples that you can think of from that category in one minute. For instance, if I said flowers, you might say rose, daisy, etc. Do you understand?''

''Now go ahead and tell me all the different **ANIMALS** you can think of.''

Introduce the following categories – **FRUIT**, **BIRDS**, **BREED OF DOG** in the same way.

Engage in casual conversation for approximately 1 minute.

Then introduce the inanimate categories of **HOUSEHOLD ITEMS**, **TOOLS**, **VEHICLES** and **TYPES OF BOAT** in the same way as above.

Notes:

- **TAPE THIS TEST** (optional) since subjects normally say more than you can write down. You can then go back and check if you have missed anything out, provided the individual gives permission to tape the test.
- Write down everything that the subject says including comments etc. and try to note down on the score sheet at what point during the minute they say it.
- Subjects often stop and say for instance "Birds, do they count as animals?" Try not to interrupt the flow, but answer very briefly e.g. "Yes, that's fine."
- If subjects stop within the minute encourage them to continue for the full 60 seconds. Encourage at the end of the minute if appropriate. Sometimes they refuse to do any more, so you can say "There are a few seconds left so we'll just let the time run on".

Scoring for the Category Fluency test:

<u>Animals</u>

- Mythical animals are not allowed, e.g. "Unicorn" is scored as an Intrusion.

Confidential © 2017 Clintec All Rights Reserved

- "Cuddly toy" is an Intrusion.

- Mark superordinates as "other", e.g. if a subject says "cat, dog, sheepdog, greyhound, horse...", mark 'dog' as Other, 'sheepdog' and 'greyhound' as Correct Responses.

- If subject says, "black dog", mark as Other.

- Insects are acceptable.

- "Calf, cow, bull" are all acceptable, and would be scored as 3 Correct Responses.

- If a subject says "I gave you dog" but they didn't, still give them a Correct Response.

- If subject had already said it, count it as an Other response (because the subject has remembered correctly that dog has already been given). If subject says, "Did I say dog?" and they are unsure, but they have in fact said it, then mark it as a Perseveration. If they haven't said it, then mark as a Correct Response.

<u>Birds</u>

- If patient says, "Does robin count?" mark it as Correct Response.
- If patient says, "Do dogs count?" mark it as an Intrusion.

<u>Breeds of dog</u>

- German Shepherd and Alsatian are counted as 2 separate Correct Responses.
- Mongrel is also a Correct Response.
- "Hunting/Racing dog" is not, mark as Other.

<u>Household Items</u>

- "Kitchen, bedroom, bath, toilet, window" etc. (i.e. household fittings/fixtures) are Intrusions.
- If patient says "Chair, table, coffee table, picture", mark 'table' as Other and 'coffee table' as Correct Response
- Don't allow cheese, shampoo, herbs etc.
- Carpets, cupboards, doors = Intrusions
- Furniture, chair, desk, table.... Furniture = Other, the rest are Correct Responses.

<u>Vehicles</u>

- Sidecar is not a Correct Response
- Wheelbarrow, caravan, skis, skates, skateboard, wheelchair are all Correct Responses.
- If subject says "plane, ferry, car ferry, train..." mark 'ferry' as Correct Responses and "car ferry' as Perseveration.

Confidential © 2017 Clintec All Rights Reserved

- If subject gives you makes of car e.g. Ford, BMW etc, these are correct, but they would then not get a point for the response 'car' (as it would be a superordinate and counted as an Other).

Boats

- Any names of boats e.g. QE2/Queen Mary are counted as Other responses

General remarks:

- Only score responses that are relevant to the task, i.e. patient questions and ramblings are entered as Other responses
- If the patient makes a phonemic error, e.g. guinea fig (for animals) and you can understand what they mean, mark as Correct Response. If not, mark as Other
- If patient corrects self or takes their comment back, it doesn't count as a Correct Response
- For household items, if patient says ''Chair, table, broom, toothbrush, hair brush… Oh, what are we on? Brushes?'', you can correct them and say, 'No, we are on household items'', i.e. if patient forgets and asks what category they are on, you can remind them.
- However, you cannot correct them unless they specifically ask, i.e. don't correct the patient unless they ask for guidance.

Instructions for Letter Fluency Test:

''This time I'm going to give you a letter of the alphabet and ask you to name as many different words as you can think that start with that letter. I don't want you to include the names of people or places. You'll have one minute to think of as many different words as you can. Try not to give the same words with different endings, e.g. run, runner and running.''

''Now go ahead and tell me all the different words that you can think of that start with the letter '**F**'.''

Repeat the above instructions for the letters **A** and **S**.

Scoring Letter Fluency:

- Proper Names and Places are scored as Intrusions because subject has been told explicitly NOT to give such words
- However, other proper names e.g. Friday, August, Sabbath are accepted because the instruction only mentions People and Places and not Proper nouns per se
- Groups of words such as 'run, runner, running' would be scored as 1 correct response and 2 preservations if testing for words beginning with the letter r
- Active, activate – would be scored as 2 correct responses

Confidential © 2017 Clintec All Rights Reserved

- Sink, sunk – again would score 2 correct responses because subject was not given an explicit instruction not to make new words by changing tenses
- pill, pillbox – scored as 2 correct responses (because they are two separate entries in the dictionary)
- frying pan, full moon – these are compound nouns and are therefore scored as correct responses
- If in doubt, CONSULT A DICTIONARY to see if the word given is a noun. As a rule of thumb, if the word has a separate entry to a similar word (as for pill and pillbox, above) it is scored as a correct response.

Confidential © 2017 Clintec All Rights Reserved

13.12 Appendix 12 Symbol Digit Modalities Test

The Symbol Digit Modalities Test (SDMT) detects cognitive impairment in less than five minutes. This simple, economical test is an ideal way for busy clinicians to screen for organic cerebral dysfunction in both children (eight years and older) and adults.

The SDMT is brief, easy to administer, and has demonstrated remarkable sensitivity in detecting not only the presence of brain damage, but also changes in cognitive functioning over time and in response to treatment.

The SDMT involves a simple substitution task that normal children and adults can easily perform. Using a reference key, the test taker has 90 seconds to pair specific numbers with given geometric figures. Responses can be written or given orally, and administration time is just five minutes for either response mode.

Individuals with cerebral dysfunction perform poorly on the SDMT, in spite of normal or above average intelligence. Studies documented in the SDMT Manual have shown the test effective in a wide range of clinical applications.

Instructions:
The SDMT is used to assess divided attention, visual scanning, tracking and motor speed. The SDMT can be administered orally and/or in written form. The SDMT requires the examinee to substitute a number, either orally or in writing, for randomised presentations of geometric figures.

The written test can be used alone but where an examinee obtains a score of 1.5 SD below the mean or lower on the written test, it is recommended that the oral test be administered for comparative purposes. Because the test involves only geometric figures and numbers, the SDMT is relatively culture free and can be administered to individuals who do not speak English. Scoring involves summing the number of correct substitutions within the 90 second interval (max = 110).

The SDMT manual reports that in a healthy sample, the test-retest reliability coefficient is 0.76 for the oral response and 0.80 for the written version. The SDMT manual suggests that comparisons between performance on the written and oral forms of the test can indicate possible areas of deficit or dysfunction such as manual motor skill or writing difficulties, speech disturbances, visual acuity, dyslexia, and/ or other learning difficulties. However, it does not elaborate upon how to interpret test results to indicate these possible and variable areas of deficit and to distinguish them from the many other possible forms of ‘cerebral dysfunction’ which, it is claimed, the test can assist in identifying or confirming.

Confidential © 2017 Clintec All Rights Reserved

≥	±	«	Π	ж	ψ	Δ	o	↑
1	2	3	4	5	6	7	8	9

ψ	±	Π	ψ	±	o	≥	Δ	↑	ж	±	«	±	≥	Δ
6	2	4												

ж	Δ	↑	o	Π	«	Δ	↑	ж	±	«	«	«	ж	ψ

o	±	«	Π	ж	ψ	≥	o	±	≥	±	«	«	ψ	o

≥	Π	«	ψ	ж	±	Δ	o	↑	o	±	«	Π	ж	«

±	±	«	Π	ж	ψ	o	±	o	≥	±	«	Π	o	ψ

«	Π	«	Δ	«	Π	Δ	o	↑	Δ	«	«	Δ	ж	ψ

≥	±	«	±	ж	«	±	o	«	≥	±	±	Π	Δ	ψ

Confidential © 2017 Clintec All Rights Reserved

13.13 Appendix 13 Mini-International Neuropsychiatric Interview (version 7.0.2)

Patient Name:	______________	***Patient Number:***	______________
Date of Birth:	______________	***Time Interview Began:***	______________
Interviewer's Name:	______________	***Time Interview Ended:***	______________
Date of Interview:		***Total Time:***	

	MODULES	TIME FRAME	MEETS CRITERIA	ICD-10-CM	PRIMARY DIAGNOSIS
A	MAJOR DEPRESSIVE EPISODE	Current (2 weeks)	☐		
		Past	☐		
		Recurrent	☐		
	MAJOR DEPRESSIVE DISORDER	Current (2 weeks)	☐	F32.x	☐
		Past	☐	F32.x	☐
		Recurrent	☐	F33.x	☐
B	SUICIDALITY	Current (Past Month)	☐		☐
		Lifetime attempt	☐	☐ Low ☐ Moderate ☐ High	☐
	SUICIDE BEHAVIOR DISORDER	Current	☐	(In Past Year)	☐
		In early remission	☐	(1 - 2 Years Ago)	☐
C	MANIC EPISODE	Current	☐		
		Past	☐		
	HYPOMANIC EPISODE	Current	☐		
		Past	☐	☐ Not Explored	
	BIPOLAR I DISORDER	Current	☐	F31.0 - F31.76	☐
		Past	☐	F31.0 - F31.76	☐
	BIPOLAR I DISORDER WITH PSYCHOTIC FEATURES	Current	☐	F31.2/31.5/F31.64	☐
		Past	☐	F31.2/31.5/F31.64	☐
	BIPOLAR II DISORDER	Current	☐	F31.81	☐
		Past	☐	F31.81	☐
	OTHER SPECIFIED BIPOLAR AND RELATED DISORDER	Current	☐	F31.89	☐
		Past	☐	F31.89	☐
D	PANIC DISORDER	Current (Past Month)	☐	F41.0	☐
		Lifetime	☐	F40.0	☐
E	AGORAPHOBIA	Current	☐	F40.00	☐
F	SOCIAL ANXIETY DISORDER (Social Phobia)	Current (Past Month)	☐	F40.10	☐
G	OBSESSIVE-COMPULSIVE DISORDER	Current (Past Month)	☐	F42.2	☐
H	POSTTRAUMATIC STRESS DISORDER	Current (Past Month)	☐	F43.10	☐
I	ALCOHOL USE DISORDER	Past 12 Months	☐	F10.10 - F10.21	☐
J	SUBSTANCE USE DISORDER (Non-alcohol)	Past 12 Months	☐	F11.10 - F19.21	☐

It is illegal to post this copyrighted PDF on any website.

Confidential © 2017 Clintec All Rights Reserved

K	ANY PSYCHOTIC DISORDER	Current<br>Lifetime	☐<br>☐	F20.81-F29<br>F20.81-F29	☐<br>☐
	MAJOR DEPRESSIVE DISORDER WITH PSYCHOTIC FEATURES	Current<br>Past	☐<br>☐	F32.3/F33.3<br>F32.3/F33.3	☐<br>☐
	BIPOLAR I DISORDER WITH PSYCHOTIC FEATURES	Current<br>Past	☐<br>☐	F31.2/F31.5/F31.64<br>F31.2/F31.5/F31.64	☐<br>☐
L	ANOREXIA NERVOSA	Current (Past 3 Months)	☐	F50.01/F50.02	☐
M	BULIMIA NERVOSA	Current (Past 3 Months)	☐	F50.2	☐
MB	BINGE-EATING DISORDER	Current (Past 3 Months)	☐	F50.81	☐
N	GENERALIZED ANXIETY DISORDER	Current (Past 6 Months)	☐	F41.1	☐
O	MEDICAL, ORGANIC, DRUG CAUSE RULED OUT		☐ No	☐ Yes	☐ Uncertain
P	ANTISOCIAL PERSONALITY DISORDER	Lifetime	☐	F60.2	☐

IDENTIFY THE PRIMARY DIAGNOSIS BY CHECKING THE APPROPRIATE CHECK BOX.
(Which problem troubles you the most or dominates the others or came first in the natural history?)

It is illegal to post this copyrighted PDF on any website.

Confidential © 2017 Clintec All Rights Reserved

GENERAL INSTRUCTIONS

The M.I.N.I. was designed as a brief structured interview for the major psychiatric disorders in DSM-5 and ICD-10. Validation and reliability studies have been done comparing the M.I.N.I. to the SCID-P for DSM-III-R and the CIDI (a structured interview developed by the World Health Organization). The results of these studies show that the M.I.N.I. has similar reliability and validity properties, but can be administered in a much shorter period of time (mean 18.7 ± 11.6 minutes, median 15 minutes) than the above referenced instruments. Clinicians can use it, after a brief training session. Lay interviewers require more extensive training.

INTERVIEW:

In order to keep the interview as brief as possible, inform the patient that you will conduct a clinical interview that is more structured than usual, with very precise questions about psychological problems which require a yes or no answer.

GENERAL FORMAT:

The M.I.N.I. is divided into **modules** identified by letters, each corresponding to a diagnostic category.

•At the beginning of each diagnostic module (except for psychotic disorders module), screening question(s) corresponding to the main criteria of the disorder are presented in a **gray box**.

•At the end of each module, diagnostic box(es) permit the clinician to indicate whether diagnostic criteria are met.

CONVENTIONS:

Sentences written in « normal font » should be read exactly as written to the patient in order to standardize the assessment of diagnostic criteria.

Sentences written in « CAPITALS » should not be read to the patient. They are instructions for the interviewer to assist in the scoring of the diagnostic algorithms.

Sentences written in « **bold** » indicate the time frame being investigated. The interviewer should read them as often as necessary. Only symptoms occurring during the time frame indicated should be considered in scoring the responses.

Answers with an arrow above them (➡) indicate that one of the criteria necessary for the diagnosis or diagnoses is not met. In this case, the interviewer should go to the end of the module, circle « **NO** » in all the diagnostic boxes and move to the next module.

When terms are separated by a *slash (/)* the interviewer should read only those symptoms known to be present in the patient (for example, questions J2b or K6b).

Phrases in (parentheses) are clinical examples of the symptom. These may be read to the patient to clarify the question.

RATING INSTRUCTIONS:

All questions must be rated. The rating is done at the right of each question by circling either YES or NO. Clinical judgment by the rater should be used in coding the responses. Interviewers need to be sensitive to the diversity of cultural beliefs in their administration of questions and rating of responses. The rater should ask for examples when necessary, to ensure accurate coding. The patient should be encouraged to ask for clarification on any question that is not absolutely clear.

The clinician should be sure that each dimension of the question is taken into account by the patient (for example, time frame, frequency, severity, and/or alternatives).

Symptoms better accounted for by an organic cause or by the use of alcohol or drugs should not be coded positive in the M.I.N.I. The M.I.N.I. has questions that investigate these issues.

For any questions, suggestions, need for a training session or information about updates of the M.I.N.I., please contact:
David V Sheehan, M.D., M.B.A.
University of South Florida College of Medicine
tel : +1 813-956-8437
e-mail : dsheehan@health.usf.edu
For licensing, permissions, or questions, contact: davidvsheehan@gmail.com

It is illegal to post this copyrighted PDF on any website.

Confidential © 2017 Clintec All Rights Reserved

A. MAJOR DEPRESSIVE EPISODE

(➡ MEANS: GO TO THE DIAGNOSTIC BOX, CIRCLE **NO** IN THE DIAGNOSTIC BOX, AND MOVE TO THE NEXT MODULE)

A1	a	Were you ever depressed or down, or felt sad, empty or hopeless most of the day, nearly every day, for two weeks?	NO	YES
		IF NO, CODE NO TO **A1b**: IF **YES** ASK:		
	b	For the past two weeks, were you depressed or down, or felt sad, empty or hopeless most of the day, nearly every day?	NO	YES
A2	a	Were you ever much less interested in most things or much less able to enjoy the things you used to enjoy most of the time, for two weeks?	NO	YES
		IF NO, CODE NO TO **A2b**: IF **YES** ASK:		
	b	In the past two weeks, were you much less interested in most things or much less able to enjoy the things you used to enjoy, most of the time?	NO	YES
		IS **A1a** OR **A2a** CODED **YES**?	➡ NO	YES

A3 IF **A1b** OR **A2b** = **YES**: EXPLORE THE **CURRENT** AND THE MOST SYMPTOMATIC **PAST** EPISODE, OTHERWISE
IF **A1b** AND **A2b** = **NO**: EXPLORE **ONLY** THE MOST SYMPTOMATIC **PAST** EPISODE.

			Past 2 Weeks		Past Episode	
		**Over that two week period, when you felt depressed or uninterested:**				
	a	Was your appetite decreased or increased nearly every day? Did your weight decrease or increase without trying intentionally (i.e., by ±5% of body weight or ±8 lb or ± 3.5 kg, for a 160 lb/70 kg person in a month)? IF **YES** TO EITHER, CODE **YES**.	NO	YES	NO	YES
	b	Did you have trouble sleeping nearly every night (difficulty falling asleep, waking up in the middle of the night, early morning wakening or sleeping excessively)?	NO	YES	NO	YES
	c	Did you talk or move more slowly than normal or were you fidgety, restless or having trouble sitting still almost every day? Did anyone notice this?	NO	YES	NO	YES
	d	Did you feel tired or without energy almost every day?	NO	YES	NO	YES
	e	Did you feel worthless or guilty almost every day?	NO	YES	NO	YES
		IF **YES**, ASK FOR EXAMPLES. LOOK FOR DELUSIONS OF FAILURE, OF INADEQUACY, OF RUIN OR OF GUILT, OR OF NEEDING PUNISHMENT OR DELUSIONS OF DISEASE OR DEATH OR NIHILISTIC OR SOMATIC DELUSIONS. THE EXAMPLES ARE CONSISTENT WITH A DELUSIONAL IDEA. Current Episode ☐ No ☐ Yes; Past Episode ☐ No ☐ Yes				
	f	Did you have difficulty concentrating, thinking or making decisions almost every day?	NO	YES	NO	YES
	g	Did you repeatedly think about death (**FEAR OF DYING DOES NOT COUNT HERE**), or have any thoughts of killing yourself, or have any intent or plan to kill yourself? Did you attempt suicide? **IF YES TO EITHER, CODE YES.**	NO	YES	NO	YES
A4		Did these symptoms cause significant distress or problems at home, at work, at school, socially, in your relationships, or in some other important way, and are they a change from your previous functioning?	NO	YES	NO	YES

It is illegal to post this copyrighted PDF on any website.

Confidential © 2017 Clintec All Rights Reserved

A5		In between 2 episodes of depression, did you ever have an interval of at least 2 months, without any significant depression or any significant loss of interest?	N/A NO YES

ARE **5** OR MORE ANSWERS **(A1-A3)** CODED **YES** AND IS **A4** CODED **YES** FOR THAT TIME FRAME?

AND

IS "RULE OUT ORGANIC CAUSE **(O2 SUMMARY)**" CODED **YES**?

SPECIFY IF THE EPISODE IS CURRENT AND / OR PAST.

IF **A5** IS CODED **YES**, CODE **YES** FOR RECURRENT.

**NO**	**YES**
MAJOR DEPRESSIVE EPISODE	
CURRENT	☐
PAST	☐
RECURRENT	☐

A6	a	How many episodes of depression did you have in your lifetime? _____

Between each episode there must be at least 2 months without any significant depression.

It is illegal to post this copyrighted PDF on any website

Confidential © 2017 Clintec All Rights Reserved

B. SUICIDALITY

				Points
	**In the past month did you:**			
B1	Have any accident? This includes taking too much of your medication accidentally.<br>IF NO TO **B1**, SKIP TO **B2**. IF YES, ASK **B1a**:	NO	YES	0
B1a	Plan or intend to hurt yourself in any accident, either by not avoiding a risk or by causing the accident on purpose?<br>IF NO TO **B1a**, SKIP TO **B2**. IF YES, ASK **B1b**:	NO	YES	0
B1b	Intend to die as a result of any accident?	NO	YES	0
B2	Think (even momentarily) that you would be better off dead or wish you were dead or needed to be dead?	NO	YES	1
B3	Think (even momentarily) about harming or of hurting or of injuring yourself<br>- with at least some intent or awareness that you might die as a result<br>- or think about suicide (i.e. about killing yourself)?	NO	YES	6

IF NO TO **B2 + B3**, SKIP TO **B4**. OTHERWISE ASK:

Frequency		Intensity	
Occasionally	☐	Mild	☐
Often	☐	Moderate	☐
Very often	☐	Severe	☐

B4	Hear a voice or voices telling you to kill yourself or have a dream with any suicidal content?<br>IF YES, mark either or both: ☐ was it a voice or voices? ☐ was it a dream?	NO	YES	4
B5	Have a suicide method in mind (i.e. how)?	NO	YES	8
B6	Have a suicide means in mind (i.e. with what)?	NO	YES	8
B7	Have any place in mind to attempt suicide (i.e. where)?	NO	YES	8
B8	Have any date/timeframe in mind to attempt suicide (i.e. when)?	NO	YES	8
B9	Think about any task you would like to complete before trying to kill yourself? (e.g. writing a suicide note)	NO	YES	8
B10	Intend to act on thoughts of killing yourself?<br>IF YES, mark either or both: ☐ did you intend to act at the time?<br>☐ did you intend to act at some time in the future?	NO	YES	8
B11	Intend to die as a result of a suicidal act?<br>IF YES, mark either or both: ☐ did you intend to die by suicide at the time?<br>☐ did you intend to die by suicide at some time in the future?	NO	YES	8
B12	Feel the need or impulse to kill yourself or to plan to kill yourself sooner rather than later?<br>IF YES, mark either or both: ☐ was this to kill yourself? ☐ was this to plan to kill yourself?<br>IF YES, mark either or both: ☐ was this largely unprovoked? ☐ was this provoked?	NO	YES	8

IN ASSESSING WHETHER THIS WAS LARGELY UNPROVOKED ASK: "5 minutes before this Impulse, could you have predicted it would occur at that time?"
IF NO TO **B12**, SKIP TO **B14**.

It is illegal to post this copyrighted PDF on any website.

Confidential © 2017 Clintec All Rights Reserved

B13	Have difficulty resisting these impulses?	NO	YES	8
B14	Take any active steps to prepare for a suicide attempt in which you expected or intended to die (include anything done or purposely not done that put you closer to making a suicide attempt)? This includes times when you were going to kill yourself, but were interrupted or stopped yourself, before harming yourself.<br>IF NO TO **B14**, SKIP TO **B15**.	NO	YES	
B14a	Take active steps to prepare to kill yourself, but you **did not start** the suicide attempt?	NO	YES	9
B14b	Take active steps to prepare to kill yourself, but then **you stopped yourself just before** harming yourself ("aborted")?	NO	YES	10
B14c	Take active steps to prepare to kill yourself, but then **someone or something stopped you just before** harming yourself ("interrupted")?	NO	YES	11
B15	Injure yourself on purpose without intending to kill yourself?<br>(B15 IS NOT COUNTED AS SUICIDAL BEHAVIOR)	NO	YES	0
B16	Attempt suicide (to kill yourself)?<br>IF NO TO **B16**, SKIP TO **B17**.	NO	YES	
B16a	Start a suicide attempt (to kill yourself), but then **you decided to stop** and did not finish the attempt?	NO	YES	12
B16b	Start a suicide attempt (to kill yourself), but then **you were interrupted** and did not finish the attempt?	NO	YES	13
B16c	Went through with a suicide attempt (to kill yourself), **completely** as you meant to? A suicide attempt means you did something where you could possibly be injured, with at least a slight intent to die.<br>IF NO TO **B16c**, SKIP TO **B17**:<br>Hope to be rescued / survive ☐<br>Expected / intended to die ☐	NO	YES	14

B17 **TIME SPENT PER DAY WITH ANY SUICIDAL IMPULSES, THOUGHTS OR ACTIONS:**

Usual time spent per day: ____ hours ____ minutes.
Least amount of time spent per day: ____ hours ____ minutes.
Most amount of time spent per day: ____ hours ____ minutes.

In your lifetime:

B18	Did you ever make a suicide attempt (try to kill yourself)?<br>If **YES**, how many times? ______________<br>If **YES**, when was the last suicide attempt?<br>Current: within the past 12 months ☐<br>In early remission: between 12 and 24 months ago ☐<br>In remission: more than 24 months ago ☐	NO	YES	4

"A suicide attempt is any self-injurious behavior, with at least some intent (> 0) to die as a result of the act. Evidence that the individual intended to kill him-or herself, at least to some degree, can be explicit or inferred from the behavior or circumstance. For example, it is defined as a suicide attempt if it is clearly not an accident or if the individual thinks the act could be lethal, even though denying intent." (FDA Guidance for Industry Suicidal Ideation and Behavior Document 2012 and C-CASA definition). Posner K et al. Am J Psychiatry 2007; 164 (7): 1035-1043 & http://www.fda.gov/Drugs/GuidanceComplianceRegulatoryInformation/Guidances/default.htm/

B19	How likely are you to try to kill yourself within the next 3 months on a scale of 0-100% ______%<br>ANY LIKELIHOOD > 0% ON **B19** SHOULD BE CODED YES	NO	YES	13

It is illegal to post this copyrighted PDF on any website.

Confidential © 2017 Clintec All Rights Reserved

IS AT LEAST **1** OF THE ABOVE (EXCEPT **B1**) CODED **YES**?

IF **YES**, ADD THE TOTAL POINTS FOR THE ANSWERS (**B1-B19**) CHECKED 'YES' AND SPECIFY THE SUICIDALITY SCORE CATEGORY AS INDICATED IN THE DIAGNOSTIC BOX:

INDICATE WHETHER THE SUICIDALITY IS CURRENT (PAST MONTH) OR A LIFETIME SUICIDE ATTEMPT OR BOTH BY MARKING THE APPROPRIATE BOXES OR BY LEAVING EITHER OR BOTH OF THEM UNMARKED.
CURRENT = ANY POSITIVE RESPONSE IN **B1a** THROUGH **B16c** (EXCEPT B15) OR ANY TIME SPENT IN **B17**.
LIFETIME ATTEMPT = **B18** CODED **YES**.
LIKELY IN THE NEAR FUTURE = **B19** CODED **YES**.

MAKE ANY ADDITIONAL COMMENTS ABOUT YOUR ASSESSMENT OF THIS PATIENT'S CURRENT AND NEAR FUTURE SUICIDALITY IN THE SPACE BELOW:

**NO**		**YES**
SUICIDALITY		
1-8 points	Low	☐
9-16 points	Moderate	☐
≥ 17 points	High	☐
CURRENT		☐
LIFETIME ATTEMPT		☐
LIKELY IN NEAR FUTURE		☐

IS **B18** CODED **YES**?

AND A YES RESPONSE TO

WAS THE SUICIDAL ACT STARTED WHEN THE SUBJECT WAS NOT IN A STATE OF CONFUSION OR DELIRIUM?

AND A YES RESPONSE TO

WAS THE SUICIDAL ACT DONE WITHOUT A POLITICAL OR RELIGIOUS PURPOSE?
IF **YES**, SPECIFY WHETHER THE DISORDER IS CURRENT, IN EARLY REMISSION OR IN REMISSION.

**NO**	**YES**
SUICIDAL BEHAVIOR DISORDER	
Current	☐
In early remission	☐
In remission	☐

It is illegal to post this copyrighted PDF on any website.

Confidential © 2017 Clintec All Rights Reserved

C. MANIC AND HYPOMANIC EPISODES

(➡ MEANS: GO TO THE DIAGNOSTIC BOXES, CIRCLE NO IN MANIC AND HYPOMANIC DIAGNOSTIC BOXES, AND MOVE TO NEXT MODULE)

Do you have any family history of manic-depressive illness or bipolar disorder, or any family member who had mood swings treated with a medication like lithium, sodium valproate (Depakote) or lamotrigine (Lamictal)? — NO YES

THIS QUESTION IS NOT A CRITERION FOR BIPOLAR DISORDER, BUT IS ASKED TO INCREASE THE CLINICIAN'S VIGILANCE ABOUT THE RISK FOR BIPOLAR DISORDER.

IF YES, PLEASE SPECIFY WHO:______________________________

C1	a	Have you **ever** had a period of time when you were feeling 'up' or 'high' or 'hyper' and so active or full of energy or full of yourself that you got into trouble, - or that other people thought you were not your usual self? (Do not consider times when you were intoxicated on drugs or alcohol.)<br><br>IF PATIENT IS PUZZLED OR UNCLEAR ABOUT WHAT YOU MEAN BY 'UP' OR 'HIGH' OR 'HYPER', CLARIFY AS FOLLOWS: By 'up' or 'high' or 'hyper' I mean: having elated mood; increased energy or increased activity; needing less sleep; having rapid thoughts; being full of ideas; having an increase in productivity, motivation, creativity, or impulsive behavior; phoning or working excessively or spending more money.<br><br>IF **NO**, CODE **NO** TO **C1b**: IF **YES** ASK:	NO	YES
	b	Are you currently feeling 'up' or 'high' or 'hyper' or full of energy?	NO	YES
C2	a	Have you **ever** been persistently irritable, for several days, so that you had arguments or verbal or physical fights, or shouted at people outside your family? Have you or others noticed that you have been more irritable or over reacted, compared to other people, even in situations that you felt were justified?<br><br>IF **NO**, CODE **NO** TO **C2b**: IF **YES** ASK:	NO	YES
	b	Are you currently feeling persistently irritable?	NO ➡	YES
		IS **C1a** OR **C2a** CODED **YES**?	NO	YES

C3 IF **C1b** OR **C2b** = **YES**: EXPLORE THE **CURRENT** EPISODE FIRST AND THEN THE MOST SYMPTOMATIC **PAST** EPISODE, OTHERWISE
IF **C1b** AND **C2b** = **NO**: EXPLORE **ONLY** THE MOST SYMPTOMATIC **PAST** EPISODE

WHEN EXPLORING THE CURRENT EPISODE, PREFACE EACH QUESTION AS FOLLOWS:
Over the past few days including today, when you felt high and full of energy or irritable, did you:

WHEN EXPLORING THE PAST EPISODE, PREFACE EACH QUESTION AS FOLLOWS:
Over a period of a few days in the past, when you felt most high and most full of energy or most irritable, did you:

		Current Episode		Past Episode	
a	Feel that you could do things others couldn't do, or that you were an especially important person? IF YES, ASK FOR EXAMPLES.<br>THE EXAMPLES ARE CONSISTENT WITH A DELUSIONAL IDEA. Current Episode ☐ No ☐ Yes<br>Past Episode ☐ No ☐ Yes	NO	YES	NO	YES
b	Need less sleep (for example, feel rested after only a few hours sleep)?	NO	YES	NO	YES

Confidential © 2017 Clintec All Rights Reserved

It is illegal to post this copyrighted PDF on any website.

		Current Episode		Past Episode	
c	Talk too much without stopping, or felt a pressure to keep talking?	NO	YES	NO	YES
d	Notice your thoughts going very fast or running together or racing or moving very quickly from one subject to another?	NO	YES	NO	YES
e	Become easily distracted so that any little interruption could distract you?	NO	YES	NO	YES
f	Have a significant increase in your activity or drive, at work, at school, socially or sexually or did you become physically or mentally restless? This increase in activity may be with or without a purpose.	NO	YES	NO	YES
g	Want so much to engage in pleasurable activities that you ignored the risks or consequences (for example, spending sprees, reckless driving, or sexual indiscretions)?	NO	YES	NO	YES
C3 **SUMMARY:**	WHEN RATING CURRENT EPISODE:<br>IF **C1b** IS **NO**, ARE **4** OR MORE **C3** ANSWERS **INCLUDING C3f** CODED **YES**?<br>IF **C1b** IS **YES**, ARE **3** OR MORE **C3** ANSWERS **INCLUDING C3f** CODED **YES**?<br><br>WHEN RATING PAST EPISODE:<br>IF **C1a** IS **NO**, ARE **4** OR MORE **C3** ANSWERS **INCLUDING C3f** CODED **YES**?<br>IF **C1a** IS **YES**, ARE **3** OR MORE **C3** ANSWERS **INCLUDING C3f** CODED **YES**?<br><br>CODE **YES** ONLY IF THE ABOVE 3 OR 4 SYMPTOMS OCCURRED DURING THE SAME TIME PERIOD.<br><br>RULE: ELATION/EXPANSIVENESS REQUIRES ONLY **3** OF THE **C3** SYMPTOMS, WHILE IRRITABLE MOOD ALONE REQUIRES **4** OF THE **C3** SYMPTOMS.	NO	YES	NO	YES
C4	What is the longest time these symptoms lasted (most of the day nearly every day)?<br>ASSESS THIS DURATION FROM THE VERY START TO THE VERY END OF SYMPTOMS, NOT JUST THE PEAK.				
	a) 3 consecutive days or less		☐		☐
	b) 4, 5 or 6 consecutive days or more		☐		☐
	c) 7 consecutive days or more		☐		☐
C5	Were you hospitalized for these problems?<br><br>IF **YES**, CIRCLE **YES** IN MANIC EPISODE FOR THAT TIME FRAME AND GO TO **C7**.	NO	YES	NO	YES
C6	Did these symptoms cause significant problems at home, at work, socially, in your relationships, at school or in some other important way?	NO	YES	NO	YES
C7	Were these symptoms associated with a clear change in the way that you previously functioned and that was different from the way that you usually are?	NO	YES	NO	YES

ARE **C3 SUMMARY** AND **C7** AND (**C4c** OR **C5** OR **C6** OR ANY PSYCHOTIC FEATURE IN **K1** THROUGH **K8**) CODED **YES**?

AND

IS "RULE OUT ORGANIC CAUSE (**O2 SUMMARY**)" CODED **YES**?

SPECIFY IF THE EPISODE IS CURRENT AND / OR PAST.

**NO**	**YES**
MANIC EPISODE	
CURRENT	☐
PAST	☐

It's illegal to post this copyrighted PDF on any website.

Confidential © 2017 Clintec All Rights Reserved

IS **C3 SUMMARY** CODED **YES** AND ARE **C5** AND **C6** CODED **NO** AND **C7** CODED **YES**,
AND IS EITHER **C4b** OR **C4c** CODED **YES**?
AND
IS "RULE OUT ORGANIC CAUSE (**O2 SUMMARY**)" CODED **YES**?
AND
ARE **ALL** PSYCHOTIC FEATURES IN **K1** THROUGH **K8** CODED **NO**?

SPECIFY IF THE EPISODE IS CURRENT AND / OR PAST.

IF **YES** TO CURRENT MANIC EPISODE, THEN CODE CURRENT HYPOMANIC EPISODE AS **NO**.

IF **YES** TO PAST MANIC EPISODE, THEN CODE PAST HYPOMANIC EPISODE AS **NOT EXPLORED**.

HYPOMANIC EPISODE

CURRENT ☐ **NO**
☐ **YES**

PAST ☐ **NO**
☐ **YES**
☐ **NOT EXPLORED**

ARE **C3 SUMMARY** AND **C4a** CODED **YES** AND IS **C5** CODED **NO**?

SPECIFY IF THE EPISODE IS CURRENT AND / OR PAST.

IF **YES** TO CURRENT MANIC EPISODE OR HYPOMANIC EPISODE,
THEN CODE CURRENT HYPOMANIC SYMPTOMS AS **NO**.

IF **YES** TO PAST MANIC EPISODE OR **YES** TO PAST HYPOMANIC EPISODE,
THEN CODE PAST HYPOMANIC SYMPTOMS AS **NOT EXPLORED**.

HYPOMANIC SYMPTOMS

CURRENT ☐ **NO**
☐ **YES**

PAST ☐ **NO**
☐ **YES**
☐ **NOT EXPLORED**

C8 a) IF MANIC EPISODE IS POSITIVE FOR EITHER CURRENT OR PAST ASK:
Did you have 2 or more of these (manic) episodes lasting 7 days or more (**C4c**) in your lifetime (including the current episode if present)? NO YES

b) IF MANIC OR HYPOMANIC EPISODE IS POSITIVE FOR EITHER CURRENT OR PAST ASK:
Did you have 2 or more of these (hypomanic) episodes lasting 4 days or more (**C4b**) in your lifetime (including the current episode)? NO YES

c) IF THE PAST "HYPOMANIC SYMPTOMS" CATEGORY IS CODED POSITIVE ASK:
Did you have these hypomanic symptoms lasting only 1 to 3 days (**C4a**) 2 or more times in your lifetime, (including the current episode if present)? NO YES

It is illegal to post this copyrighted PDF on any website.

Confidential © 2017 Clintec All Rights Reserved

I. ALCOHOL USE DISORDER

(➡ MEANS: GO TO DIAGNOSTIC BOX, CIRCLE NO AND MOVE TO THE NEXT MODULE)

			➡	
I1		**In the past 12 months**, have you had 3 or more alcoholic drinks, - within a 3-hour period, - on 3 or more occasions?	NO	YES
I2		**In the past 12 months:**		
	a	During the times when you drank alcohol, did you end up drinking more than you planned when you started?	NO	YES
	b	Did you repeatedly want to reduce or control your alcohol use?<br>Did you try to cut down or control your alcohol use, but failed?<br>IF YES TO EITHER, CODE YES.	NO	YES
	c	On the days that you drank, did you spend substantial time obtaining alcohol, drinking, or recovering from the effects of alcohol?	NO	YES
	d	Did you crave or have a strong desire or urge to use alcohol?	NO	YES
	e	Did you spend less time meeting your responsibilities at work, at school, or at home, because of your repeated drinking?	NO	YES
	f	If your drinking caused problems with your family or other people, did you still keep on drinking?	NO	YES
	g	Were you intoxicated more than once in any situation where you or others were physically at risk, for example, driving a car, riding a motorbike, using machinery, boating, etc.?	NO	YES
	h	Did you continue to use alcohol, even though it was clear that the alcohol had caused or worsened psychological or physical problems?	NO	YES
	i	Did you reduce or give up important work, social or recreational activities because of your drinking?	NO	YES
	j	Did you need to drink a lot more in order to get the same effect that you got when you first started drinking or did you get much less effect with continued use of the same amount?	NO	YES
	k1	When you cut down on heavy or prolonged drinking did you have any of the following:<br>1. increased sweating or increased heart rate, ☐<br>2. hand tremor or "the shakes" ☐<br>3. trouble sleeping ☐<br>4. nausea or vomiting ☐<br>5. hearing or seeing things other people could not see or hear or having sensations in your skin for no apparent reason ☐<br>6. agitation ☐<br>7. anxiety ☐<br>8. seizures ☐<br>IF YES TO 2 OR MORE OF THE ABOVE 8, CODE k1 AS YES.	NO	YES
	k2	Did you drink alcohol to reduce or avoid withdrawal symptoms or to avoid being hung-over?	NO	YES

It is illegal to post this copyrighted PDF on any website.

Confidential © 2017 Clintec All Rights Reserved

I2k SUMMARY: IF YES TO I2k1 OR I2k2, CODE YES — NO YES

ARE 2 OR MORE I2 ANSWERS FROM I2a THROUGH I2k SUMMARY CODED YES?
(I2k1 AND I2k2 TOGETHER COUNT AS ONE AMONG THESE CHOICES)

NO	YES
ALCOHOL USE DISORDER	
PAST 12 MONTHS	

SPECIFIERS FOR ALCOHOL USE DISORDER:

MILD = 2-3 OF THE I2 SYMPTOMS
MODERATE = 4-5 OF THE I2 SYMPTOMS
SEVERE = 6 OR MORE OF THE I2 SYMPTOMS

IN EARLY REMISSION = CRITERIA NOT MET FOR BETWEEN 3 & 12 MONTHS.
IN SUSTAINED REMISSION = CRITERIA NOT MET FOR 12 MONTHS OR MORE.
(BOTH WITH THE EXCEPTION OF CRITERION d [CRAVING] ABOVE.)

IN A CONTROLLED ENVIRONMENT = WHERE ALCOHOL ACCESS IS RESTRICTED.

SPECIFY IF:

MILD ☐
MODERATE ☐
SEVERE ☐

IN EARLY REMISSION ☐
IN SUSTAINED REMISSION ☐

IN A CONTROLLED ENVIRONMENT ☐

It is illegal to post this copyrighted PDF on any website.

Confidential © 2017 Clintec All Rights Reserved

J. SUBSTANCE USE DISORDER (NON-ALCOHOL)

(➡ MEANS: GO TO THE DIAGNOSTIC BOX, CIRCLE NO AND MOVE TO THE NEXT MODULE)

		**Now I am going to show you / read to you a list of street drugs or medicines.**		
J1	a	**In the past 12 months**, did you take any of these drugs more than once, to get high, to feel elated, to get "a buzz" or to change your mood?	➡ NO	YES

CIRCLE EACH DRUG TAKEN:

Stimulants: amphetamines, "speed", crystal meth, "crank", Dexedrine, Ritalin, diet pills.

Cocaine: snorting, IV, freebase, crack, "speedball".

Opiates: heroin, morphine, Dilaudid, opium, Demerol, methadone, Darvon, codeine, Percodan, Vicodin, OxyContin.

Hallucinogens: LSD ("acid"), mescaline, peyote, psilocybin, STP, "mushrooms", "ecstasy", MDA, MDMA.

Dissociative Drugs: PCP (Phencyclidine ,"Angel Dust", "Peace Pill", "Hog"), or ketamine ("Special K").

Inhalants: "glue", ethyl chloride, "rush", nitrous oxide ("laughing gas"), amyl or butyl nitrate ("poppers").

Cannabis: marijuana, hashish ("hash"), THC, "pot", "grass", "weed", "reefer".

Sedatives, Hypnotics or Anxiolytics: Quaalude, Seconal ("reds"), Valium, Xanax, Librium, Ativan, Dalmane, Halcion, barbiturates, Miltown, GHB, Roofinol, "Roofies".

Miscellaneous: steroids, nonprescription sleep or diet pills. Cough Medicine? Any others?

SPECIFY THE MOST USED DRUG(S): ____________________

WHICH DRUG(S) CAUSE THE BIGGEST PROBLEMS? ____________________

FIRST EXPLORE THE CRITERIA BELOW FOR THE DRUG CLASS CAUSING THE BIGGEST PROBLEMS AND THE ONE MOST LIKELY TO MEET CRITERIA FOR SUBSTANCE USE DISORDER. IF SEVERAL DRUG CLASSES HAVE BEEN MISUSED, EXPLORE AS MANY OR AS FEW AS REQUIRED BY THE PROTOCOL.

J2		**Considering your use of (NAME OF DRUG / DRUG CLASS SELECTED), in the past 12 months:**		
	a	During the times when you used the drug, did you end up using more (NAME OF DRUG / DRUG CLASS SELECTED) than you planned when you started?	NO	YES
	b	Did you repeatedly want to reduce or control your (NAME OF DRUG / DRUG CLASS SELECTED) use? Did you try to cut down or control your (NAME OF DRUG / DRUG CLASS SELECTED) use, but failed? IF **YES** TO EITHER, CODE **YES**.	NO	YES
	c	On the days that you used more (NAME OF DRUG / DRUG CLASS SELECTED), did you spend substantial time obtaining (NAME OF DRUG / DRUG CLASS SELECTED), using it, or recovering from the its effects?	NO	YES
	d	Did you crave or have a strong desire or urge to use (NAME OF DRUG / DRUG CLASS SELECTED)?	NO	YES
	e	Did you spend less time meeting your responsibilities at work, at school, or at home, because of your repeated (NAME OF DRUG / DRUG CLASS SELECTED) use?	NO	YES
	f	If your (NAME OF DRUG / DRUG CLASS SELECTED) use caused problems with your family or other people, did you still keep on using it?	NO	YES
	g	Did you use the drug more than once in any situation where you or others were physically at risk, for example, driving a car, riding a motorbike, using machinery, boating, etc.?	NO	YES
	h	Did you continue to use (NAME OF DRUG / DRUG CLASS SELECTED), even though it was clear that the (NAME OF DRUG / DRUG CLASS SELECTED) had caused or worsened psychological or physical problems?	NO	YES

It is illegal to post this copyrighted PDF on any website.

Confidential © 2017 Clintec All Rights Reserved

i	Did you reduce or give up important work, social or recreational activities because of your (NAME OF DRUG / DRUG CLASS SELECTED) use?	NO	YES
j	Did you need to use (NAME OF DRUG / DRUG CLASS SELECTED) a lot more in order to get the same effect that you got when you first started using it or did you get much less effect with continued use of the same amount?<br>THIS CRITERION IS CODED **NO** IF THE MEDICATION IS PRESCRIBED AND USED UNDER APPROPRIATE MEDICAL SUPERVISION.	NO	YES
k1	When you cut down on heavy or prolonged use of the drug did you have any of the following withdrawal symptoms:<br>IF **YES** TO THE REQUIRED NUMBER OF WITHDRAWAL SYMPTOMS FOR EACH CLASS, CODE **J2k1** AS **YES**.<br>THIS CRITERION IS CODED **NO** IF THE MEDICATION IS PRESCRIBED AND USED UNDER APPROPRIATE MEDICAL SUPERVISION.	NO	YES

Sedatives, Hypnotics or Anxiolytics (2 or more withdrawal symptoms)
1. increased sweating or increased heart rate ☐
2. hand tremor or "the shakes" ☐
3. trouble sleeping ☐
4. nausea or vomiting ☐
5. hearing or seeing things other people could not see or hear or having sensations in your skin for no apparent reason ☐
6. agitation ☐
7. anxiety ☐
8. seizures ☐

Opiates (3 or more withdrawal symptoms)
1. feeling depressed ☐
2. nausea or vomiting ☐
3. muscle aches ☐
4. runny nose or teary eyes ☐
5. dilated pupils, goose bumps or hair standing on end or sweating ☐
6. diarrhea ☐
7. yawning ☐
8. hot flashes ☐
9. trouble sleeping ☐

Stimulants and Cocaine (2 or more withdrawal symptoms)
1. fatigue ☐
2. vivid or unpleasant dreams ☐
3. difficulty sleeping or sleeping too much ☐
4. increased appetite ☐
5. feeling or looking physically or mentally slowed down ☐

Cannabis (3 or more withdrawal symptoms)
1. irritability, anger or aggression ☐
2. nervousness or anxiety ☐
3. trouble sleeping ☐
4. appetite or weight loss ☐
5. restlessness ☐
6. feeling depressed ☐
7. significant discomfort from one of the following: "stomach pain", tremors or "shakes", sweating, hot flashes, chills, headaches. ☐

k2	Did you use (NAME OF DRUG / DRUG CLASS SELECTED) to reduce or avoid withdrawal symptoms?	NO	YES

It is illegal to post this copyrighted PDF on any website.

Confidential © 2017 Clintec All Rights Reserved

J2k SUMMARY: IF **YES** TO **J2k1** <u>OR</u> **J2k2**, CODE **YES**. NO YES

ARE **2** OR MORE **J2** ANSWERS FROM **J2a** THROUGH **J2k SUMMARY** CODED **YES**?
(**J2k1** AND **J2k2** TOGETHER COUNT AS ONE AMONG THESE CHOICES.)

NO	YES
SUBSTANCE (Drug or Drug Class Name) USE DISORDER	
PAST 12 MONTHS	

SPECIFIERS FOR SUBSTANCE USE DISORDER:

MILD = **2-3** OF THE **J2** SYMPTOMS
MODERATE = **4-5** OF THE **J2** SYMPTOMS
SEVERE = **6** OR MORE OF THE **J2** SYMPTOMS

IN EARLY REMISSION = CRITERIA NOT MET FOR BETWEEN 3 & 12 MONTHS.
IN SUSTAINED REMISSION = CRITERIA NOT MET FOR 12 MONTHS OR MORE.
(BOTH WITH THE EXCEPTION OF **CRITERION d** [CRAVING] ABOVE.)

IN A CONTROLLED ENVIRONMENT = WHERE SUBSTANCE / DRUG ACCESS IS RESTRICTED.

SPECIFY IF:

MILD ☐
MODERATE ☐
SEVERE ☐

IN EARLY REMISSION ☐
IN SUSTAINED REMISSION ☐

IN A CONTROLLED ENVIRONMENT ☐

It is illegal to post this copyrighted PDF on any website.

Confidential © 2017 Clintec All Rights Reserved

K. PSYCHOTIC DISORDERS AND MOOD DISORDER WITH PSYCHOTIC FEATURES

ASK FOR AN EXAMPLE OF EACH QUESTION ANSWERED POSITIVELY. CODE **YES** ONLY IF THE EXAMPLES CLEARLY SHOW A DISTORTION OF THOUGHT OR OF PERCEPTION OR IF THEY ARE NOT CULTURALLY APPROPRIATE. THE PURPOSE OF THIS MODULE IS TO EXCLUDE PATIENTS WITH PSYCHOTIC DISORDERS. THIS MODULE NEEDS EXPERIENCE.

Now I am going to ask you about unusual experiences that some people have.

K1	a	Have you ever believed that people were spying on you, or that someone was plotting against you, or trying to hurt you?<br>**NOTE:** ASK FOR EXAMPLES TO RULE OUT ACTUAL STALKING.	NO	YES
	b	IF **YES:** do you currently believe these things?	NO	YES
K2	a	Have you ever believed that someone was reading your mind or could hear your thoughts, or that you could actually read someone's mind or hear what another person was thinking?	NO	YES
	b	IF **YES:** do you currently believe these things?	NO	YES
K3	a	Have you ever believed that someone or some force outside of yourself put thoughts in your mind that were not your own, or made you act in a way that was not your usual self? Have you ever felt that you were possessed?<br>CLINICIAN: ASK FOR EXAMPLES AND DISCOUNT ANY THAT ARE NOT PSYCHOTIC.	NO	YES
	b	IF **YES:** do you currently believe these things?	NO	YES
K4	a	Have you ever believed that you were being sent special messages through the TV, radio, internet, newspapers, books, or magazines or that a person you did not personally know was particularly interested in you?	NO	YES
	b	IF **YES:** do you currently believe these things?	NO	YES
K5	a	Have your relatives or friends ever considered any of your beliefs odd or unusual?<br>CLINICIAN: ASK FOR EXAMPLES. ONLY CODE YES IF THE EXAMPLES ARE CLEARLY DELUSIONAL IDEAS NOT EXPLORED IN QUESTIONS **K1** TO **K4**. FOR EXAMPLE, RELIGIOUS, DEATH, DISEASE OR SOMATIC DELUSIONS, DELUSIONS OF GRANDIOSITY, JEALOUSY OR GUILT, OR OF FAILURE, INADEQUACY, RUIN, OR DESTITUTION, OR NIHILISTIC DELUSIONS.	NO	YES
	b	IF **YES:** do they currently consider your beliefs strange or unusual?	NO	YES
K6	a	Have you ever heard things other people couldn't hear, such as voices?	NO	YES
		**IF YES TO VOICE HALLUCINATION:** Was the voice commenting on your thoughts or behavior or did you hear two or more voices talking to each other?	NO	YES
	b	IF YES TO **K6a:** have you heard sounds / voices in the past month?	NO	YES
		**IF YES TO VOICE HALLUCINATION:** Was the voice commenting on your thoughts or behavior or did you hear two or more voices talking to each other?	NO	YES

It is illegal to post this copyrighted PDF on any website.

Confidential © 2017 Clintec All Rights Reserved

K7	a	Have you ever had visions when you were awake or have you ever seen things other people couldn't see?<br>CLINICIAN: CHECK TO SEE IF THESE ARE CULTURALLY INAPPROPRIATE.	NO	YES
	b	IF YES: have you seen these things in the past month?	NO	YES
		**CLINICIAN'S JUDGMENT**		
K8	a	DID THE PATIENT EVER IN THE PAST EXHIBIT DISORGANIZED, INCOHERENT OR DERAILED SPEECH, OR MARKED LOOSENING OF ASSOCIATIONS?	NO	YES
K8	b	IS THE PATIENT CURRENTLY EXHIBITING INCOHERENCE, DISORGANIZED OR DERAILED SPEECH, OR MARKED LOOSENING OF ASSOCIATIONS?	NO	YES
K9	a	DID THE PATIENT EVER IN THE PAST EXHIBIT DISORGANIZED OR CATATONIC BEHAVIOR?	NO	YES
K9	b	IS THE PATIENT CURRENTLY EXHIBITING DISORGANIZED OR CATATONIC BEHAVIOR?	NO	YES
K10	a	DID THE PATIENT EVER IN THE PAST HAVE NEGATIVE SYMPTOMS, E.G. SIGNIFICANT REDUCTION OF EMOTIONAL EXPRESSION OR AFFECTIVE FLATTENING, POVERTY OF SPEECH (ALOGIA) OR AN INABILITY TO INITIATE OR PERSIST IN GOAL-DIRECTED ACTIVITIES (AVOLITION)?	NO	YES
K10	b	ARE NEGATIVE SYMPTOMS OF SCHIZOPHRENIA, E.G. SIGNIFICANT REDUCTION OF EMOTIONAL EXPRESSION OR AFFECTIVE FLATTENING, POVERTY OF SPEECH (ALOGIA) OR AN INABILITY TO INITIATE OR PERSIST IN GOAL-DIRECTED ACTIVITIES (AVOLITION), PROMINENT DURING THE INTERVIEW?	NO	YES
K11	a	ARE 1 OR MORE « a » QUESTIONS FROM K1a TO K7a, CODED YES?<br><br>**AND IS EITHER:**<br><br>MAJOR DEPRESSIVE EPISODE, (CURRENT, RECURRENT OR PAST)<br>**OR**<br>MANIC OR HYPOMANIC EPISODE, (CURRENT OR PAST) CODED YES?<br><br>**AND**<br><br>HOW LONG HAS THE MOOD EPISODE LASTED? ___________<br>HOW LONG HAS THE PSYCHOTIC EPISODE LASTED? ___________<br>IF SUCH A MOOD EPISODE IS PRESENT, CODE **YES** TO **K11a** ONLY IF THE MOOD DISTURBANCE IS PRESENT FOR THE MAJORITY OF THE TOTAL DURATION OF THE ACTIVE AND RESIDUAL PERIODS OF THE PSYCHOTIC SYMPTOMS. OTHERWISE CODE **NO**.	NO<br>↳ **K13**	YES

IF **NO** TO **K11a** AND THE TOTAL DURATION OF THE MOOD EPISODE IS **LESS THAN** THE TOTAL DURATION OF THE PSYCHOTIC EPISODE, THEN CIRCLE **NO** IN BOTH 'MOOD DISORDER WITH PSYCHOTIC FEATURES' DIAGNOSTIC BOXES AND MOVE TO **K13**.

It is illegal to post this copyrighted PDF on any website.

Confidential © 2017 Clintec All Rights Reserved

b You told me earlier that you had period(s) when you felt (depressed/high/persistently irritable).

Were the beliefs and experiences you just described (SYMPTOMS CODED **YES** FROM **K1a** TO **K7a**) restricted exclusively to times when you were feeling depressed/high/irritable?

IF THE PATIENT EVER HAD A PERIOD OF AT LEAST **2 WEEKS** OF HAVING THESE BELIEFS OR EXPERIENCE (PSYCHOTIC SYMPTOMS) WHEN THEY WERE NOT DEPRESSED/HIGH/IRRITABLE, CODE **NO** TO THIS DISORDER.

IF THE ANSWER IS **NO** TO THIS DISORDER GROUPING, ALSO CIRCLE **NO** TO **K12** AND MOVE TO **K13**

NO **YES**

MOOD DISORDER WITH *PSYCHOTIC FEATURES*

LIFETIME

K12 a ARE **1** OR MORE « **b** » QUESTIONS FROM **K1b** TO **K7b** CODED **YES**?

AND IS EITHER:

MAJOR DEPRESSIVE EPISODE (CURRENT)
OR
MANIC OR HYPOMANIC EPISODE (CURRENT) CODED **YES**?

IF THE ANSWER IS **YES** TO THIS DISORDER (LIFETIME OR CURRENT), CIRCLE **NO** TO **K13** AND **K14** AND MOVE TO THE NEXT MODULE.

NO **YES**

MOOD DISORDER WITH *PSYCHOTIC FEATURES*

CURRENT

K13 ARE **1** OR MORE « **b** » QUESTIONS FROM **K1b** TO **K8b**, CODED **YES**?

AND

ARE **2** OR MORE « **b** » QUESTIONS FROM **K1b** TO **K10b**, CODED **YES**?

AND DID AT LEAST TWO OF THE PSYCHOTIC SYMPTOMS OCCUR DURING THE SAME 1-MONTH PERIOD?

AND

IS "RULE OUT ORGANIC CAUSE (**O2 SUMMARY**)" CODED **YES**?

NO **YES**

PSYCHOTIC DISORDER
CURRENT

K14 IS **K13** CODED **YES**?

OR

(ARE **1** OR MORE « **a** » QUESTIONS FROM **K1a** TO **K8a**, CODED **YES**?

AND

ARE **2** OR MORE « **a** » QUESTIONS FROM **K1a** TO **K10a**, CODED **YES**

AND

DID AT LEAST **2** OF THE PSYCHOTIC SYMPTOMS OCCUR DURING THE SAME 1-MONTH PERIOD?)

AND

IS "RULE OUT ORGANIC CAUSE (**O2 SUMMARY**)" CODED **YES**?

NO **YES**

PSYCHOTIC DISORDER
LIFETIME

It is illegal to post this copyrighted PDF on any website.

Confidential © 2017 Clintec All Rights Reserved

13.14 Appendix 14 Maudsley Staging Method

To support the effort to better understand and stage TRD, a multidimensional staging model, the Maudsley Staging Method (MSM) was developed. The initial development of the model was based on extensive literature review, and systematic assessment of the dimensions making the MSM as well as testing of the construct using original data. The MSM has shown promising predictive validity for both short-term and longer-term outcomes of TRD. In addition to indications of construct validity based on more elaborate evaluation, the MSM has also been used for screening purposes in clinical trials and in studies of determinants of treatment outcomes.

The tool was developed as a loosely structured instrument such that a clinician with mental health training would be able to complete it.

TABLE 6

MAUDSLEY STAGING METHOD FOR TREATMENT RESISTANT DEPRESSION – RECOMMENDED SCORING CONVENTIONS

Parameter/Dimension	Parameter Specification	Score
Duration	Acute (≤ 12 months)	1
	Sub-acute (13-24 months)	2
	Chronic (> 24 months)	3
Symptom Severity (at baseline)	Subsyndromal	1
	Syndromal	
	▪ Mild	2
	▪ Moderate	3
	▪ Severe without psychosis	4
	▪ Severe with psychosis	5
Treatment Failures		
▪ Antidepressants	Level 1: 1 – 2 Medications	1
	Level 2: 3 – 4 Medications	2
	Level 3: 5 – 6 Medications	3
	Level 4: 7 – 10 Medications	4
	Level 5: > 10 Medications	5
▪ Augmentation[1]	Not Used	0
	Used	1
▪ Electroconvulsive Therapy	Not Used	0
	Used	1
Total		(15)

[1] Augmentation refers exclusively to the use of medication. Non-pharmacological treatments e.g. psychotherapy are not rated.

(Fekadu, Wooderson, Donaldson, et al., 2009)

Confidential © 2017 Clintec All Rights Reserved

13.15 Appendix 15 Montreal Cognitive Assessment

The Montreal Cognitive Assessment (MoCA) is a brief cognitive screening tool for Mild Cognitive Impairment. It was validated in the setting of mild cognitive impairment and has subsequently been adopted in numerous other settings clinically. The MoCA test is a one-page 30-point test administered in approximately 10 minutes. The test and administration instructions are available for clinicians online. The test is available in 46 languages and dialects.

MoCA Version 8.1 June 28, 2017 © Z. Nasreddine MD www.mocatest.org

Montreal Cognitive Assessment (MoCA)
Version 8.1

Administration and Scoring Instructions
The Montreal Cognitive Assessment (MoCA) was designed as a rapid screening instrument for mild cognitive dysfunction. It assesses different cognitive domains: attention and concentration, executive functions, memory, language, visuoconstructional skills, conceptual thinking, calculations, and orientation. The MoCA may be administered by anyone who understands and follows the instructions, however, only a health professional with expertise in the cognitive field may interpret the results. Time to administer the MoCA is approximately 10 minutes. The total possible score is 30 points; a score of 26 or above is considered normal.

All instructions may be repeated once.

1. Alternating Trail Making:
Administration: The examiner instructs the subject: *"Please draw a line going from a number to a letter in ascending order. Begin here* [point to (1)] *and draw a line from 1 then to A then to 2 and so on. End here* [point to (E)]."

Scoring: One point is allocated if the subject successfully draws the following pattern:
1- A- 2- B- 3- C- 4- D- 5- E, without drawing any lines that cross. Any error that is not immediately self-corrected (meaning corrected before moving on to the Cube task) earns a score of 0. A point is not allocated if the subject draws a line to connect the end (E) to the beginning (1).

2. Visuoconstructional Skills (Cube):
Administration: The examiner gives the following instructions, pointing to the cube: "*Copy this drawing as accurately as you can.*"
Scoring: One point is allocated for a correctly executed drawing.
• Drawing must be three-dimensional.
• All lines are drawn.
• All lines meet with little or no space.
• No line is added.

Confidential © 2017 Clintec All Rights Reserved

• Lines are relatively parallel, and their length is similar (rectangular prisms are accepted).
• The cube's orientation in space must be preserved.

A point is not assigned if any of the above criteria is not met.

3. Visuoconstructional Skills (Clock):
Administration: The examiner must ensure that the subject does not look at his/her watch while performing the task and that no clocks are in sight. The examiner indicates the appropriate space and gives the following instructions: *"Draw a clock. Put in all the numbers and set the time to 10 past 11."*
Scoring: One point is allocated for each of the following three criteria:

• Contour (1 pt.): the clock contour must be drawn (either a circle or a square). Only minor distortions are acceptable (e.g., slight imperfection on closing the circle). If the numbers are arranged in a circular manner but the contour is not drawn the contour is scored as incorrect.
• Numbers (1 pt.): all clock numbers must be present with no additional numbers. Numbers must be in the correct order, upright and placed in the approximate quadrants on the clock face. Roman numerals are acceptable. The numbers must be arranged in a circular manner (even if the contour is a square). All numbers must either be placed inside or outside the clock contour. If the subject places some numbers inside the clock contour and some outside the clock contour, (s)he does not receive a point for Numbers.
• Hands (1 pt.): there must be two hands jointly indicating the correct time. The hour hand must be clearly shorter than the minute hand. Hands must be centered within the clock face with their junction close to the clock center.

4. Naming:
Administration: Beginning on the left, the examiner points to each figure and says: *"Tell me the name of this animal."*

Scoring: One point is given for each of the following responses: (1) lion (2) rhinoceros or rhino (3) camel or dromedary.

5. Memory:
Administration: The examiner reads a list of five words at a rate of one per second, giving the following instructions: *"This is a memory test. I am going to read a list of words that you will have to remember now and later on. Listen carefully. When I am through, tell me as many words as you can remember. It doesn't matter in what order you say them."* The examiner marks a check in the allocated space for each word the subject produces on this first trial. The examiner may not correct the subject if (s)he recalls a deformed word or a word that sounds like the target word. When the subject indicates that (s)he has finished (has recalled all words), or can recall no more words, the examiner reads the list a second time with the following instructions: *"I am going to read the same list for a second time. Try to remember and tell me as many words as you can, including words you said the first time."* The examiner puts a check in the allocated space for each word the subject recalls on the second trial. At the end of the second trial, the examiner

Confidential © 2017 Clintec All Rights Reserved

informs the subject that (s)he will be asked to recall these words again by saying: *"I will ask you to recall those words again at the end of the test."*

Scoring: No points are given for Trials One and Two.

6. Attention:
Forward Digit Span: Administration: The examiner gives the following instructions: "*I am going to say some numbers and when I am through, repeat them to me exactly as I said them.*" The examiner reads the five number sequence at a rate of one digit per second.
Backward Digit Span: Administration: The examiner gives the following instructions: "*Now I am going to say some more numbers, but when I am through you must repeat them to me in the backward order.*" The examiner reads the three number sequence at a rate of one digit per second. If the subject repeats the sequence in the forward order, the examiner may not ask the subject to repeat the sequence in backward order at this point.

Scoring: One point is allocated for each sequence correctly repeated (N.B.: the correct response for the backward trial is 2-4-7).

Vigilance: Administration: The examiner reads the list of letters at a rate of one per second, after giving the following instructions: "*I am going to read a sequence of letters. Every time I say the letter A, tap your hand once. If I say a different letter, do not tap your hand.*"
Scoring: One point is allocated if there is zero to one error (an error is a tap on a wrong letter or a failure to tap on letter A).

Serial 7s: Administration: The examiner gives the following instructions: *"Now, I will ask you to count by subtracting 7 from 100, and then, keep subtracting 7 from your answer until I tell you to stop."* The subject must perform a mental calculation, therefore, (s)he may not use his/her fingers nor a pencil and paper to execute the task. The examiner may not repeat the subject's answers. If the subject asks what her/his last given answer was or what number (s)he must subtract from his/her answer, the examiner responds by repeating the instructions if not already done so.

Scoring: This item is scored out of 3 points. Give no (0) points for no correct subtractions, 1 point for one correct subtraction, 2 points for two or three correct subtractions, and 3 points if the subject successfully makes four or five correct subtractions. Each subtraction is evaluated independently; that is, if the subject responds with an incorrect number but continues to correctly subtract 7 from it, each correct subtraction is counted. For example, a subject may respond "92 – 85 – 78 – 71 – 64" where the "92" is incorrect, but all subsequent numbers are subtracted correctly. This is one error and the task would be given a score of 3.

7. Sentence repetition:
Administration: The examiner gives the following instructions: *"I am going to read you a sentence. Repeat it after me, exactly as I say it* [pause]: ***I only know that John is the one to help today."*** Following the response, say: *"Now I am going to read you another sentence. Repeat it*

Confidential © 2017 Clintec All Rights Reserved

after me, exactly as I say it [pause]: ***The cat always hid under the couch when dogs were in the room.”***
Scoring: One point is allocated for each sentence correctly repeated. Repetitions must be exact. Be alert for omissions (e.g., omitting "only"), substitutions/additions (e.g., substituting "only" for "always"), grammar errors/altering plurals (e.g. "hides" for "hid"), etc.

8. Verbal fluency:
Administration: The examiner gives the following instructions: *“Now, I want you to tell me as many words as you can think of that begin with the letter F. I will tell you to stop after one minute. Proper nouns, numbers, and different forms of a verb are not permitted. Are you ready?* [Pause] [Time for 60 sec.] *Stop.”* If the subject names two consecutive words that begin with another letter of the alphabet, the examiner repeats the target letter if the instructions have not yet been repeated.

Scoring: One point is allocated if the subject generates 11 words or more in 60 seconds. The examiner records the subject’s responses in the margins or on the back of the test sheet.

9. Abstraction:
Administration: The examiner asks the subject to explain what each pair of words has in common, starting with the example: *“I will give you two words and I would like you to tell me to what category they belong to* [pause]: *an orange and a banana.*” If the subject responds correctly the examiner replies: *‘‘Yes, both items are part of the category Fruits.’’* If the subject answers in a concrete manner, the examiner gives one additional prompt: *“Tell me another category* to *which these items belong to.”* If the subject does not give the appropriate response *(fruits)*, the examiner says: *“Yes, and they also both belong to the category Fruits.”* No additional instructions or clarifications are given. After the practice trial, the examiner says: *“Now, a train and a bicycle.”* Following the response, the examiner administers the second trial by saying: *“Now, a ruler and a watch.”* A prompt (one for the entire abstraction section) may be given if none was used during the example.

Scoring: Only the last two pairs are scored. One point is given for each pair correctly answered. The following responses are acceptable:
- train-bicycle = means of transportation, means of travelling, you take trips in both
- ruler-watch = measuring instruments, used to measure

The following responses are **not** acceptable:
- train-bicycle = they have wheels
- ruler-watch = they have numbers

10. Delayed recall:
Administration: The examiner gives the following instructions: *“I read some words to you earlier, which I asked you to remember. Tell me as many of those words as you can remember.”* The examiner makes a check mark (√) for each of the words correctly recalled spontaneously without any cues, in the allocated space.

Confidential © 2017 Clintec All Rights Reserved

Scoring: **One point is allocated for each word recalled freely without any cues.**

Memory index score (MIS):
Administration: Following the delayed free recall trial, the examiner provides a category (semantic) cue for each word the subject was unable to recall. Example: ''*I will give you some hints to see if it helps you remember the words, the first word was a body part*.'' If the subject is unable to recall the word with the category cue, the examiner provides him/her with a multiple-choice cue. Example: *"Which of the following words do you think it was, NOSE, FACE, or HAND?"* All non-recalled words are prompted in this manner. The examiner identifies the words the subject was able to recall with the help of a cue (category or multiple-choice) by placing a check mark (√) in the appropriate space. The cues for each word are presented below:

**Target Word**	**Category Cue**	**Multiple Choice**
FACE	body part	nose, face, hand (shoulder, leg)
VELVET	type of fabric	denim, velvet, cotton (nylon, silk)
CHURCH	type of building	church, school, hospital (library, store)
DAISY	type of flower	rose, daisy, tulip (lily, daffodil)
RED	color	red, blue, green (yellow, purple)

* The words in parentheses are to be used if the subject mentions one or two of the multiple-choice responses during the category cuing.

Scoring: To determine the MIS (which is a sub-score), the examiner attributes points according to the type of recall (see table below). The use of cues provides clinical information on the nature of the memory deficits. For memory deficits due to retrieval failures, performance can be improved with a cue. For memory deficits due to encoding failures, performance does not improve with a cue.

**MIS scoring**				**Total**
Number of words recalled spontaneously	…	multiplied by	3	…
Number of words recalled with a category	…	multiplied by	2	…

Confidential © 2017 Clintec All Rights Reserved

cue				
Number of words recalled with a multiple choice cue	…	multiplied by	1	…

Total MIS (add all points) **---/15**

11. Orientation:
Administration: The examiner gives the following instructions: "*Tell me today's date.*" If the subject does not give a complete answer, the examiner prompts accordingly by saying: *"Tell me the [year, month, exact date, and day of the week]."* Then the examiner says: *"Now, tell me the name of this place, and which city it is in."*

Scoring: One point is allocated for each item correctly answered. The date and place (name of hospital, clinic, office) must be exact. No points are allocated if the subject makes an error of one day for the day and date.

TOTAL SCORE: Sum all sub-scores listed on the right-hand side. Add one point for subject who has 12 years or fewer of formal education, for a possible maximum of 30 points. A final total score of 26 and above is considered normal.

Montreal Cognitive Assessment (MoCA)
Version 8.2

Administration and Scoring Instructions
The Montreal Cognitive Assessment (MoCA) was designed as a rapid screening instrument for mild cognitive dysfunction. It assesses different cognitive domains: attention and concentration, executive functions, memory, language, visuoconstructional skills, conceptual thinking, calculations, and orientation. The MoCA may be administered by anyone who understands and follows the instructions, however, only a health professional with expertise in the cognitive field may interpret the results. Time to administer the MoCA is approximately 10 minutes. The total possible score is 30 points; a score of 26 or above is considered normal.
All instructions may be repeated once.

1. Alternating Trail Making:
Administration: The examiner instructs the subject: *"Please draw a line going from a number to a letter in ascending order. Begin here* [point to (1)] *and draw a line from 1 then to A then to 2 and so on. End here* [point to (E)]."
Scoring: One point is allocated if the subject successfully draws the following pattern:
1- A- 2- B- 3- C- 4- D- 5- E, without drawing any lines that cross. Any error that is not immediately self-corrected (meaning corrected before moving on to the Chair task) earns a score of 0. A point is not allocated if the subject draws a line to connect the end (E) to the beginning (1).

Confidential © 2017 Clintec All Rights Reserved

2. Visuoconstructional Skills (Chair):
Administration: The examiner gives the following instructions, pointing to the chair: "*Copy this drawing as accurately as you can.*"
Scoring: One point is allocated for a correctly executed drawing.
• Drawing must be three-dimensional.
• All lines are drawn.
• All lines meet with little or no space.
• No line is added.
• Lines are relatively parallel and their length is similar.
• The chair's orientation in space must be preserved.

A point is not assigned if any of the above criteria is not met.

3. Visuoconstructional Skills (Clock):
Administration: The examiner must ensure that the subject does not look at his/her watch while performing the task and that no clocks are in sight. The examiner indicates the appropriate space and gives the following instructions: *"Draw a clock. Put in all the numbers and set the time to 10 past 9."*
Scoring: One point is allocated for each of the following three criteria: MoCA Version 8.1

• Contour (1 pt.): the clock contour must be drawn (either a circle or a square). Only minor distortions are acceptable (e.g., slight imperfection on closing the circle). If the numbers are arranged in a circular manner but the contour is not drawn the contour is scored as incorrect.
• Numbers (1 pt.): all clock numbers must be present with no additional numbers. Numbers must be in the correct order, upright and placed in the approximate quadrants on the clock face. Roman numerals are acceptable. The numbers must be arranged in a circular manner (even if the contour is a square). All numbers must either be placed inside or outside the clock contour. If the subject places some numbers inside the clock contour and some outside the clock contour, (s)he does not receive a point for Numbers.
• Hands (1 pt.): there must be two hands jointly indicating the correct time. The hour hand must be clearly shorter than the minute hand. Hands must be centered within the clock face with their junction close to the clock center.

4. Naming:
Administration: Beginning on the left, the examiner points to each figure and says: *"Tell me the name of this animal."*
Scoring: One point is given for each of the following responses: (1) snake (or a type of snake like Boa or Cobra) (2) elephant (3) crocodile or alligator.

5. Memory:
Administration: The examiner reads a list of five words at a rate of one per second, giving the following instructions*: "This is a memory test. I am going to read a list of words that you will have to remember now and later on. Listen carefully. When I am through, tell me as many words as you can remember. It doesn't matter in what order you say them."* The examiner marks a check in the allocated space for each word the subject produces on this first trial. The examiner may not correct the subject if (s)he recalls a deformed word or a word that sounds like the target word. When the subject indicates that (s)he has finished (has recalled all words), or can recall no more words, the examiner reads the list a second time with the following instructions: *"I am going to read the same list for a second time. Try to remember and tell me as many words as you can, including words you said the first time."* The examiner puts a check in the allocated space

Confidential © 2017 Clintec All Rights Reserved

for each word the subject recalls on the second trial. At the end of the second trial, the examiner informs the subject that (s)he will be asked to recall these words again by saying: *"I will ask you to recall those words again at the end of the test."*
Scoring: No points are given for Trials One and Two.

6. Attention:
Forward Digit Span: Administration: The examiner gives the following instructions: "*I am going to say some numbers and when I am through, repeat them to me exactly as I said them.*" The examiner reads the five number sequence at a rate of one digit per second.
Backward Digit Span: Administration: The examiner gives the following instructions: "*Now I am going to say some more numbers, but when I am through you must repeat them to me in the backward order.*" The examiner reads the three number sequence at a rate of one digit per second. If the subject repeats the sequence in the forward order, the examiner may not ask the subject to repeat the sequence in backward order at this point.
Scoring: One point is allocated for each sequence correctly repeated (N.B.: the correct response for the backward trial is 7-4-2).
Vigilance: Administration: The examiner reads the list of letters at a rate of one per second, after giving the following instructions: "*I am going to read a sequence of letters. Every time I say the letter A, tap your hand once. If I say a different letter, do not tap your hand.*"
Scoring: One point is allocated if there is zero to one error (an error is a tap on a wrong letter or a failure to tap on letter A).
Serial 7s: Administration: The examiner gives the following instructions: *"Now, I will ask you to count by subtracting 7 from 70, and then, keep subtracting 7 from your answer until I tell you to stop."* The subject must perform a mental calculation, therefore, (s)he may not use his/her fingers nor a pencil and paper to execute the task. The examiner may not repeat the subject's answers. If the subject asks what her/his last given answer was or what number (s)he must subtract from his/her answer, the examiner responds by repeating the instructions if not already done so.
Scoring: This item is scored out of 3 points. Give no (0) points for no correct subtractions, 1 point for one correct subtraction, 2 points for two or three correct subtractions, and 3 points if the subject successfully makes four or five correct subtractions. Each subtraction is evaluated independently; that is, if the subject responds with an incorrect number but continues to correctly subtract 7 from it, each correct subtraction is counted. For example, a subject may respond "62 - 55 - 48 - 41 – 34" where the "62" is incorrect, but all subsequent numbers are subtracted correctly. This is one error and the task would be given a score of 3.

7. Sentence repetition:
Administration: The examiner gives the following instructions: *"I am going to read you a sentence. Repeat it after me, exactly as I say it* [pause]: *The robber of the gray car was stopped by the police."* Following the response, say: *"Now I am going to read you another sentence. Repeat it after me, exactly as I say it* [pause]: *The student went back to school without his books and pencils."*
Scoring: One point is allocated for each sentence correctly repeated. Repetitions must be exact. Be alert for omissions (e.g., omitting "his"), substitutions/additions (e.g., substituting "and pencils" for "and his pencils"), grammar errors/altering plurals (e.g. "went" for "goes"), etc.

8. Verbal fluency:
Administration: The examiner gives the following instructions: *"Now, I want you to tell me as many words as you can think of that begin with the letter S. I will tell you to stop after one minute. Proper nouns, numbers, and different forms of a verb are not permitted. Are you ready?* [Pause] [Time for 60 sec.] *Stop."* If the subject names two consecutive words that begin with

Confidential © 2017 Clintec All Rights Reserved

another letter of the alphabet, the examiner repeats the target letter if the instructions have not yet been repeated.
Scoring: One point is allocated if the subject generates 11 words or more in 60 seconds. The examiner records the subject's responses in the margins or on the back of the test sheet.

9. Abstraction:
Administration: The examiner asks the subject to explain what each pair of words has in common, starting with the example: *"I will give you two words and I would like you to tell me to what category they belong to* [pause]: *an orange and a banana.*" If the subject responds correctly the examiner replies: *''Yes, both items are part of the category Fruits.''* If the subject answers in a concrete manner, the examiner gives one additional prompt: *"Tell me another category* to *which these items belong to."* If the subject does not give the appropriate response *(fruits)*, the examiner says: *"Yes, and they also both belong to the category Fruits."* No additional instructions or clarifications are given. After the practice trial, the examiner says: *"Now, a bed and a table."* Following the response, the examiner administers the second trial by saying: *"Now, a letter and a telephone."* A prompt (one for the entire abstraction section) may be given if none was used during the example.
Scoring: Only the last two pairs are scored. One point is given for each pair correctly answered. The following responses are acceptable:
- bed- table = furniture, pieces of furniture, furnishings
- letter- telephone = communication, means of communication, means of corresponding

The following responses are not acceptable:
- bed-table = flat, four legs, bedroom, made out of wood
- letter- telephone = alphabet, they have letters, they have numbers, news, messages

10. Delayed recall:
Administration: The examiner gives the following instructions: *"I read some words to you earlier, which I asked you to remember. Tell me as many of those words as you can remember."* The examiner makes a check mark (√) for each of the words correctly recalled spontaneously without any cues, in the allocated space.

Scoring: **One point is allocated for each word recalled freely without any cues.**

Memory index score (MIS):
Administration: Following the delayed free recall trial, the examiner provides a category (semantic) cue for each word the subject was unable to recall. Example: ''*I will give you some hints to see if it helps you remember the words, the first word was a body part*.'' If the subject is unable to recall the word with the category cue, the examiner provides him/her with a multiple choice cue. Example: *"Which of the following words do you think it was, HAND, SHOULDER or FACE?"* All non-recalled words are prompted in this manner. The examiner identifies the words the subject was able to recall with the help of a cue (category or multiple-choice) by placing a check mark (√) in the appropriate space. The cues for each word are presented below:

Target Word	Category Cue	Multiple Choice
HAND	body part	hand, shoulder, face (nose, leg)
NYLON	type of fabric	cotton, nylon, silk (velvet, denim)
PARK	public place	park, library, school (church,

Confidential © 2017 Clintec All Rights Reserved

		hospital)
CARROT	type of food	tomato, carrot, lettuce (cucumber, celery)
YELLOW	color	yellow, purple, green (red, blue)

* The words in parentheses are to be used if the subject mentions one or two of the multiple choice responses during the category cuing.

Scoring: To determine the MIS (which is a sub-score), the examiner attributes points according to the type of recall (see table below). The use of cues provides clinical information on the nature of the memory deficits. For memory deficits due to retrieval failures, performance can be improved with a cue. For memory deficits due to encoding failures, performance does not improve with a cue.

**MIS scoring**				**Total**
Number of words recalled spontaneously	…	multiplied by	3	…
Number of words recalled with a category cue	…	multiplied by	2	…
Number of words recalled with a multiple choice cue	…	multiplied by	1	…
**Total MIS** (add all points)				**---/15**

11. Orientation:
Administration: The examiner gives the following instructions: "*Tell me today's date.*" If the subject does not give a complete answer, the examiner prompts accordingly by saying: *"Tell me the [year, month, exact date, and day of the week]."* Then the examiner says: *"Now, tell me the name of this place, and which city it is in."*
Scoring: One point is allocated for each item correctly answered. The date and place (name of hospital, clinic, office) must be exact. No points are allocated if the subject makes an error of one day for the day and date.

TOTAL SCORE: Sum all subscores listed on the right-hand side. Add one point for subject who has 12 years or fewer of formal education, for a possible maximum of 30 points. A final total score of 26 and above is considered normal.

Montreal Cognitive Assessment (MoCA)

Confidential © 2017 Clintec All Rights Reserved

Version 8.3
Administration and Scoring Instructions

The Montreal Cognitive Assessment (MoCA) was designed as a rapid screening instrument for mild cognitive dysfunction. It assesses different cognitive domains: attention and concentration, executive functions, memory, language, visuoconstructional skills, conceptual thinking, calculations, and orientation. The MoCA may be administered by anyone who understands and follows the instructions, however, only a health professional with expertise in the cognitive field may interpret the results. Time to administer the MoCA is approximately 10 minutes. The total possible score is 30 points; a score of 26 or above is considered normal.

All instructions may be repeated once.

1. Alternating Trail Making:

Administration: The examiner instructs the subject: "Please draw a line going from a number to a letter in ascending order. Begin here [point to (1)] and draw a line from 1 then to A then to 2 and so on. End here [point to (E)]."

Scoring: One point is allocated if the subject successfully draws the following pattern: 1- A- 2- B- 3- C- 4- D- 5- E, without drawing any lines that cross. Any error that is not immediately self-corrected (meaning corrected before moving on to the Bed task) earns a score of 0. A point is not allocated if the subject draws a line to connect the end (E) to the beginning (1).

2. Visuoconstructional Skills (Bed):

Administration: The examiner gives the following instructions, pointing to the bed: "*Copy this drawing as accurately as you can*."

Scoring: One point is allocated for a correctly executed drawing. • Drawing must be three-dimensional.

• All lines are drawn.

• All lines meet with little or no space.

• No line is added.

• Lines are relatively parallel and their length is similar.

• The bed's orientation in space must be preserved.

A point is not assigned if any of the above criteria is not met.

3. Visuoconstructional Skills (Clock):

Administration: The examiner must ensure that the subject does not look at his/her watch while performing the task and that no clocks are in sight. The examiner indicates the appropriate space and gives the following instructions: "*Draw a clock. Put in all the numbers and set the time to 5 past 10.*"

Confidential © 2017 Clintec All Rights Reserved

Scoring: One point is allocated for each of the following three criteria:

• Contour (1 pt.): the clock contour must be drawn (either a circle or a square). Only minor distortions are acceptable (e.g., slight imperfection on closing the circle). If the numbers are arranged in a circular manner but the contour is not drawn the contour is scored as incorrect.

• Numbers (1 pt.): all clock numbers must be present with no additional numbers. Numbers must be in the correct order, upright and placed in the approximate quadrants on the clock face. Roman numerals are acceptable. The numbers must be arranged in a circular manner (even if the contour is a square). All numbers must either be placed inside or outside the clock contour. If the subject places some numbers inside the clock contour and some outside the clock contour, (s)he does not receive a point for Numbers.

• Hands (1 pt.): there must be two hands jointly indicating the correct time. The hour hand must be clearly shorter than the minute hand. Hands must be centered within the clock face with their junction close to the clock center.

4. Naming:

Administration: Beginning on the left, the examiner points to each figure and says: "*Tell me the name of this animal*."

Scoring: One point is given for each of the following responses: (1) horse, pony, mare or foal (2) tiger (3) duck.

5. Memory:

Administration: The examiner reads a list of five words at a rate of one per second, giving the following instructions: "*This is a memory test. I am going to read a list of words that you will have to remember now and later on. Listen carefully. When I am through, tell me as many words as you can remember. It doesn't matter in what order you say them*." The examiner marks a check in the allocated space for each word the subject produces on this first trial. The examiner may not correct the subject if (s)he recalls a deformed word or a word that sounds like the target word. When the subject indicates that (s)he has finished (has recalled all words), or can recall no more words, the examiner reads the list a second time with the following instructions: "*I am going to read the same list for a second time. Try to remember and tell me as many words as you can, including words you said the first time*." The examiner puts a check in the allocated space for each word the subject recalls on the second trial. At the end of the second trial, the examiner informs the subject that (s)he will be asked to recall these words again by saying: "*I will ask you to recall those words again at the end of the test*."

Scoring: No points are given for Trials One and Two.

6. Attention:

Forward Digit Span: Administration: The examiner gives the following instructions: "I am going to say some numbers and when I am through, repeat them to me exactly as I said them." The examiner reads the five number sequence at a rate of one digit per second.

Confidential © 2017 Clintec All Rights Reserved

Backward Digit Span: Administration: The examiner gives the following instructions: "Now I am going to say some more numbers, but when I am through you must repeat them to me in the backward order." The examiner reads the three number sequence at a rate of one digit per second. If the subject repeats the sequence in the forward order, the examiner may not ask the subject to repeat the sequence in backward order at this point.

Scoring: One point is allocated for each sequence correctly repeated (N.B.: the correct response for the backward trial is 7-2-4).

Vigilance: Administration: The examiner reads the list of letters at a rate of one per second, after giving the following instructions: "*I am going to read a sequence of letters. Every time I say the letter A, tap your hand once. If I say a different letter, do not tap your hand.*"

Scoring: One point is allocated if there is zero to one error (an error is a tap on a wrong letter or a failure to tap on letter A).

Serial 7s: Administration: The examiner gives the following instructions: "*Now, I will ask you to count by subtracting 7 from 60, and then, keep subtracting 7 from your answer until I tell you to stop*." The subject must perform a mental calculation, therefore, (s)he may not use his/her fingers nor a pencil and paper to execute the task. The examiner may not repeat the subject's answers. If the subject asks what her/his last given answer was or what number (s)he must subtract from his/her answer, the examiner responds by repeating the instructions if not already done so.

Scoring: This item is scored out of 3 points. Give no (0) points for no correct subtractions, 1 point for one correct subtraction, 2 points for two or three correct subtractions, and 3 points if the subject successfully makes four or five correct subtractions. Each subtraction is evaluated independently; that is, if the subject responds with an incorrect number but continues to correctly subtract 7 from it, each correct subtraction is counted. For example, a subject may respond "52 - 45 - 38 - 31 – 24" where the "52" is incorrect, but all subsequent numbers are subtracted correctly. This is one error and the task would be given a score of 3.

7. Sentence repetition:

Administration: The examiner gives the following instructions: "*I am going to read you a sentence. Repeat it after me, exactly as I say it* [pause]: ***The child walked his dog in the park after midnight*.**" Following the response, say: "*Now I am going to read you another sentence. Repeat it after me, exactly as I say it* [pause]: ***The artist finished his painting at the right moment for the exhibition.***"

Scoring: One point is allocated for each sentence correctly repeated. Repetitions must be exact. Be alert for omissions (e.g., omitting "right"), substitutions/additions (e.g., substituting "after" for "at"), grammar errors/altering plurals (e.g. "his painting" for "his paintings"), etc.

8. Verbal fluency:

Confidential © 2017 Clintec All Rights Reserved

Administration: The examiner gives the following instructions: "*Now, I want you to tell me as many words as you can think of that begin with the letter B. I will tell you to stop after one minute. Proper nouns, numbers, and different forms of a verb are not permitted. Are you ready?* [Pause] [Time for 60 sec.] *Stop*." If the subject names two consecutive words that begin with another letter of the alphabet, the examiner repeats the target letter if the instructions have not yet been repeated.

Scoring: One point is allocated if the subject generates 11 words or more in 60 seconds. The examiner records the subject's responses in the margins or on the back of the test sheet.

9. Abstraction:

Administration: The examiner asks the subject to explain what each pair of words has in common, starting with the example: "*I will give you two words and I would like you to tell me to what category they belong to* [pause]: *an orange and a banana*." If the subject responds correctly the examiner replies: ''*Yes, both items are part of the category Fruits*.'' If the subject answers in a concrete manner, the examiner gives one additional prompt: "*Tell me another category to which these items belong to*." If the subject does not give the appropriate response (*fruits*), the examiner says: "*Yes, and they also both belong to the category Fruits*." No additional instructions or clarifications are given. After the practice trial, the examiner says: "*Now, a hammer and a screwdriver*." Following the response, the examiner administers the second trial by saying: "*Now, matches and a lamp*." A prompt (one for the entire abstraction section) may be given if none was used during the example.

Scoring: Only the last two pairs are scored. One point is given for each pair correctly answered. The following responses are acceptable:

- hammer- screwdriver = tools, carpentry, construction, work instruments,
- matches- lamp = light, lighting, illumination

The following responses are not acceptable:

- hammer- screwdriver = instruments, have handles, metallic objects,
- matches- lamp = fire, hot objects, produce heat

10. Delayed recall:

Administration: The examiner gives the following instructions: "*I read some words to you earlier, which I asked you to remember. Tell me as many of those words as you can remember.*" The examiner makes a check mark (√) for each of the words correctly recalled spontaneously without any cues, in the allocated space.

Scoring: **One point is allocated for each word recalled freely without any cues.**

Memory index score (MIS):

Administration: Following the delayed free recall trial, the examiner provides a category (semantic) cue for each word the subject was unable to recall. Example: ''*I will give you some hints to see if it helps you remember the words, the first word was a body part*.'' If

Confidential © 2017 Clintec All Rights Reserved

the subject is unable to recall the word with the category cue, the examiner provides him/her with a multiple choice cue. Example: "*Which of the following words do you think it was, HAND, LEG, or FACE?*" All non-recalled words are prompted in this manner. The examiner identifies the words the subject was able to recall with the help of a cue (category or multiple-choice) by placing a check mark (√) in the appropriate space. The cues for each word are presented below:

**Target Word**	**Category Cue**	**Multiple Choice**
LEG	body part	Hand, leg, face (shoulder, nose)
COTTON	type of fabric	Silk, cotton, nylon (velvet, denim)
SCHOOL	type of building	School, hospital, library (church, store)
TOMATO	type of flower	Lettuce, tomato, carrot (cucumber, celery)
WHITE	color	Purple, white, green (yellow, red)

* The words in parentheses are to be used if the subject mentions one or two of the multiple choice responses during the category cuing.

Scoring: To determine the MIS (which is a sub-score), the examiner attributes points according to the type of recall (see table below). The use of cues provides clinical information on the nature of the memory deficits. For memory deficits due to retrieval failures, performance can be improved with a cue. For memory deficits due to encoding failures, performance does not improve with a cue.

**MIS scoring**				**Total**
Number of words recalled spontaneously	…	multiplied by	3	…
Number of words recalled with a category cue	…	multiplied by	2	…
Number of words recalled with a multiple choice cue	…	multiplied by	1	…
**Total MIS** (add all points)				**---/15**

11. Orientation:

Administration: The examiner gives the following instructions: "*Tell me today's date.*" If the subject does not give a complete answer, the examiner prompts accordingly by saying: "*Tell me the [year, month, exact date, and day of the week].*" Then the examiner says: "*Now, tell me the name of this place, and which city it is in.*"

Confidential © 2017 Clintec All Rights Reserved

Scoring: One point is allocated for each item correctly answered. The date and place (name of hospital, clinic, office) must be exact. No points are allocated if the subject makes an error of one day for the day and date.

TOTAL SCORE: Sum all subscores listed on the right-hand side. Add one point for subject who has 12 years or fewer of formal education, for a possible maximum of 30 points. A final total score of 26 and above is considered normal.

Confidential © 2017 Clintec All Rights Reserved

13.16 Appendix 16 Declaration of Helsinki

WORLD MEDICAL ASSOCIATION DECLARATION OF HELSINKI

Ethical Principles for Medical Research Involving Human Subjects

Adopted by the 18th WMA General Assembly, Helsinki, Finland, June 1964 and amended by the:
29th WMA General Assembly, Tokyo, Japan, October 1975
35th WMA General Assembly, Venice, Italy, October 1983
41st WMA General Assembly, Hong Kong, September 1989
48th WMA General Assembly, Somerset West, Republic of South Africa, October 1996
52nd WMA General Assembly, Edinburgh, Scotland, October 2000
53rd WMA General Assembly, Washington DC, USA, October 2002 (Note of Clarification added)
55th WMA General Assembly, Tokyo, Japan, October 2004 (Note of Clarification added)
59th WMA General Assembly, Seoul, Republic of Korea, October 2008
64th WMA General Assembly, Fortaleza, Brazil, October 2013

Preamble

1. The World Medical Association (WMA) has developed the Declaration of Helsinki as a statement of ethical principles for medical research involving human subjects, including research on identifiable human material and data.
   The Declaration is intended to be read as a whole and each of its constituent paragraphs should be applied with consideration of all other relevant paragraphs.

2. Consistent with the mandate of the WMA, the Declaration is addressed primarily to physicians. The WMA encourages others who are involved in medical research involving human subjects to adopt these principles.

General Principles

3. The Declaration of Geneva of the WMA binds the physician with the words, “The health of my patient will be my first consideration,” and the International Code of Medical Ethics declares that, “A physician shall act in the patient’s best interest when providing medical care.”

4. It is the duty of the physician to promote and safeguard the health, well-being and rights of patients, including those who are involved in medical research. The physician’s knowledge and conscience are dedicated to the fulfilment of this duty.

5. Medical progress is based on research that ultimately must include studies involving human subjects.

6. The primary purpose of medical research involving human subjects is to understand the causes, development and effects of diseases and improve preventive, diagnostic and therapeutic interventions (methods, procedures and treatments). Even the best proven interventions must be evaluated continually through research for their safety, effectiveness, efficiency, accessibility and quality.

7. Medical research is subject to ethical standards that promote and ensure respect for all human subjects and protect their health and rights.

8. While the primary purpose of medical research is to generate new knowledge, this goal can never take precedence over the rights and interests of individual research subjects.

9. It is the duty of physicians who are involved in medical research to protect the life, health, dignity, integrity, right to self-determination, privacy, and confidentiality of personal information of research subjects. The responsibility for the protection of

Confidential © 2017 Clintec All Rights Reserved

research subjects must always rest with the physician or other health care professionals and never with the research subjects, even though they have given consent.

10. Physicians must consider the ethical, legal and regulatory norms and standards for research involving human subjects in their own countries as well as applicable international norms and standards. No national or international ethical, legal or regulatory requirement should reduce or eliminate any of the protections for research subjects set forth in this Declaration.

11. Medical research should be conducted in a manner that minimises possible harm to the environment.

12. Medical research involving human subjects must be conducted only by individuals with the appropriate ethics and scientific education, training and qualifications. Research on patients or healthy volunteers requires the supervision of a competent and appropriately qualified physician or other health care professional.

13. Groups that are underrepresented in medical research should be provided appropriate access to participation in research.

14. Physicians who combine medical research with medical care should involve their patients in research only to the extent that this is justified by its potential preventive, diagnostic or therapeutic value and if the physician has good reason to believe that participation in the research study will not adversely affect the health of the patients who serve as research subjects.

15. Appropriate compensation and treatment for subjects who are harmed as a result of participating in research must be ensured.

Risks, Burdens and Benefits

16. In medical practice and in medical research, most interventions involve risks and burdens.
    Medical research involving human subjects may only be conducted if the importance of the objective outweighs the risks and burdens to the research subjects.

17. All medical research involving human subjects must be preceded by careful assessment of predictable risks and burdens to the individuals and groups involved in the research in comparison with foreseeable benefits to them and to other individuals or groups affected by the condition under investigation.
    Measures to minimise the risks must be implemented. The risks must be continuously monitored, assessed and documented by the researcher.

18. Physicians may not be involved in a research study involving human subjects unless they are confident that the risks have been adequately assessed and can be satisfactorily managed.
    When the risks are found to outweigh the potential benefits or when there is conclusive proof of definitive outcomes, physicians must assess whether to continue, modify or immediately stop the study.

Vulnerable Groups and Individuals

19. Some groups and individuals are particularly vulnerable and may have an increased likelihood of being wronged or of incurring additional harm.
    All vulnerable groups and individuals should receive specifically considered protection.

Confidential © 2017 Clintec All Rights Reserved

20. Medical research with a vulnerable group is only justified if the research is responsive to the health needs or priorities of this group and the research cannot be carried out in a non-vulnerable group. In addition, this group should stand to benefit from the knowledge, practices or interventions that result from the research.

Scientific Requirements and Research Protocols

21. Medical research involving human subjects must conform to generally accepted scientific principles, be based on a thorough knowledge of the scientific literature, other relevant sources of information, and adequate laboratory and, as appropriate, animal experimentation. The welfare of animals used for research must be respected.

22. The design and performance of each research study involving human subjects must be clearly described and justified in a research protocol.
The protocol should contain a statement of the ethical considerations involved and should indicate how the principles in this Declaration have been addressed. The protocol should include information regarding funding, sponsors, institutional affiliations, potential conflicts of interest, incentives for subjects and information regarding provisions for treating and/or compensating subjects who are harmed as a consequence of participation in the research study.
In clinical trials, the protocol must also describe appropriate arrangements for post-trial provisions.

Research Ethics Committees

23. The research protocol must be submitted for consideration, comment, guidance and approval to the concerned research ethics committee before the study begins. This committee must be transparent in its functioning, must be independent of the researcher, the sponsor and any other undue influence and must be duly qualified. It must take into consideration the laws and regulations of the country or countries in which the research is to be performed as well as applicable international norms and standards, but these must not be allowed to reduce or eliminate any of the protections for research subjects set forth in this Declaration.
The committee must have the right to monitor ongoing studies. The researcher must provide monitoring information to the committee, especially information about any serious adverse events. No amendment to the protocol may be made without consideration and approval by the committee. After the end of the study, the researchers must submit a final report to the committee containing a summary of the study's findings and conclusions.

Privacy and Confidentiality

24. Every precaution must be taken to protect the privacy of research subjects and the confidentiality of their personal information.

Informed Consent

25. Participation by individuals capable of giving informed consent as subjects in medical research must be voluntary. Although it may be appropriate to consult family members or community leaders, no individual capable of giving informed consent may be enrolled in a research study unless he or she freely agrees.

26. In medical research involving human subjects capable of giving informed consent, each potential subject must be adequately informed of the aims, methods, sources of funding, any possible conflicts of interest, institutional affiliations of the researcher, the anticipated benefits and potential risks of the study and the discomfort it may entail, post-study provisions and any other relevant aspects of the study. The potential subject must be informed of the right to refuse to participate in the study or

Confidential © 2017 Clintec All Rights Reserved

to withdraw consent to participate at any time without reprisal. Special attention should be given to the specific information needs of individual potential subjects as well as to the methods used to deliver the information.
After ensuring that the potential subject has understood the information, the physician or another appropriately qualified individual must then seek the potential subject's freely-given informed consent, preferably in writing. If the consent cannot be expressed in writing, the non-written consent must be formally documented and witnessed.
All medical research subjects should be given the option of being informed about the general outcome and results of the study.

27. When seeking informed consent for participation in a research study the physician must be particularly cautious if the potential subject is in a dependent relationship with the physician or may consent under duress. In such situations the informed consent must be sought by an appropriately qualified individual who is completely independent of this relationship.

28. For a potential research subject who is incapable of giving informed consent, the physician must seek informed consent from the legally authorised representative. These individuals must not be included in a research study that has no likelihood of benefit for them unless it is intended to promote the health of the group represented by the potential subject, the research cannot instead be performed with persons capable of providing informed consent, and the research entails only minimal risk and minimal burden.

29. When a potential research subject who is deemed incapable of giving informed consent is able to give assent to decisions about participation in research, the physician must seek that assent in addition to the consent of the legally authorised representative. The potential subject's dissent should be respected.

30. Research involving subjects who are physically or mentally incapable of giving consent, for example, unconscious patients, may be done only if the physical or mental condition that prevents giving informed consent is a necessary characteristic of the research group. In such circumstances the physician must seek informed consent from the legally authorised representative. If no such representative is available and if the research cannot be delayed, the study may proceed without informed consent provided that the specific reasons for involving subjects with a condition that renders them unable to give informed consent have been stated in the research protocol and the study has been approved by a research ethics committee. Consent to remain in the research must be obtained as soon as possible from the subject or a legally authorised representative.

31. The physician must fully inform the patient which aspects of their care are related to the research. The refusal of a patient to participate in a study or the patient's decision to withdraw from the study must never adversely affect the patient-physician relationship.

32. For medical research using identifiable human material or data, such as research on material or data contained in biobanks or similar repositories, physicians must seek informed consent for its collection, storage and/or reuse. There may be exceptional situations where consent would be impossible or impracticable to obtain for such research. In such situations the research may be done only after consideration and approval of a research ethics committee.

Use of Placebo

Confidential © 2017 Clintec All Rights Reserved

33. The benefits, risks, burdens and effectiveness of a new intervention must be tested against those of the best proven intervention(s), except in the following circumstances:
    Where no proven intervention exists, the use of placebo, or no intervention, is acceptable; or
    Where for compelling and scientifically sound methodological reasons the use of any intervention less effective than the best proven one, the use of placebo, or no intervention is necessary to determine the efficacy or safety of an intervention and the patients who receive any intervention less effective than the best proven one, placebo, or no intervention will not be subject to additional risks of serious or irreversible harm as a result of not receiving the best proven intervention.
    Extreme care must be taken to avoid abuse of this option.

Post-Trial Provisions

34. In advance of a clinical trial, sponsors, researchers and host country governments should make provisions for post-trial access for all participants who still need an intervention identified as beneficial in the trial. This information must also be disclosed to participants during the informed consent process.

Research Registration and Publication and Dissemination of Results

35. Every research study involving human subjects must be registered in a publicly accessible database before recruitment of the first subject.

36. Researchers, authors, sponsors, editors and publishers all have ethical obligations with regard to the publication and dissemination of the results of research. Researchers have a duty to make publicly available the results of their research on human subjects and are accountable for the completeness and accuracy of their reports. All parties should adhere to accepted guidelines for ethical reporting. Negative and inconclusive as well as positive results must be published or otherwise made publicly available. Sources of funding, institutional affiliations and conflicts of interest must be declared in the publication. Reports of research not in accordance with the principles of this Declaration should not be accepted for publication.

Unproven Interventions in Clinical Practice

37. In the treatment of an individual patient, where proven interventions do not exist or other known interventions have been ineffective, the physician, after seeking expert advice, with informed consent from the patient or a legally authorised representative, may use an unproven intervention if in the physician's judgement it offers hope of saving life, re-establishing health or alleviating suffering. This intervention should subsequently be made the object of research, designed to evaluate its safety and efficacy. In all cases, new information must be recorded and, where appropriate, made publicly available.

Confidential © 2017 Clintec All Rights Reserved

DocuSign Envelope ID: 9580C97D-56AD-4E50-9133-FAC5390A9286

QUANTICS
Biostatistics

BEDROC

for Douglas Pharmaceuticals Ltd.

Statistical Analysis Plan: Protocol number R107-C205

A Phase 2a Proof-of-Concept Study of R-107 for the Treatment of Refractory Major Depressive Disorder.

Version 2.0

12 November 2021

Exchange Tower, 19 Canning Street, Edinburgh EH3 8EG

DocuSign Envelope ID: 9580C97D-56AD-4E50-9133-FAC5390A9286

Contents

		Page No.
	**Signature page**	**4**
	**Section 1: Glossary of terms**	**5**
	**Section 2: Change control**	**7**
	**Section 3: Introduction**	**8**
	**Section 4: Study overview**	**9**
4.1	Study objectives	9
4.2	Study design	9
4.3	Determination of sample size	9
4.4	Treatment assignment and administration of study medication	9
4.5	Study visit schedule	11
4.6	Eligibility criteria	13
4.7	Additional data from Singapore and Taiwan	13
4.8	Changes to SAP	13
	**Section 5: Study endpoints**	**14**
5.1	Primary endpoint	14
5.2	Secondary endpoints	14
5.3	Exploratory endpoints	14
5.4	Safety endpoints	15
	**Section 6: Analysis populations**	**18**
6.1	Full analysis population	18
6.2	Per protocol population	18
6.3	Safety population	19
6.4	Enrolled population	19
6.5	Use of study populations	19
	**Section 7: General points for statistical analysis**	**20**
7.1	General methods	20
7.2	Definition of baseline	20

DocuSign Envelope ID: 9580C97D-56AD-4E50-9133-FAC5390A9286

7.3 Withdrawals and missing data 20

Section 8: Disposition and baseline characteristics 22

8.1 Subject disposition and withdrawals from treatment 22

8.2 Demographics and baseline characteristics 22

Section 9: Compliance with study product 24

Section 10: Analysis of study endpoints 26

10.1 Primary endpoint 26

10.2 Secondary endpoints 29

10.3 Exploratory endpoints 31

10.4 Safety endpoints 34

Section 11: List of tables, listings, and figures 47

References 53

Signature page

**Protocol Number:**	*R107-C205*
**Protocol Title:**	*A Phase 2a Proof-of-Concept Study of R-107 for the Treatment of Refractory Major Depressive Disorder.*
**Protocol Version (Date):**	6.0 (20 November 2020) [Singapore and Taiwan v6.1 20 November 2020]
**SAP Version:**	2.0
**SAP Date:**	12 November 2021

Quantics Consulting Limited
Daniel James
2021-11-12 14:28Z

Written by: ____________________ Date: __________

Daniel James, Director of Clinical Statistics

Quantics Consulting Ltd.

Quantics Consulting Limited
Ann Yellowlees
2021-11-12 16:51Z

Reviewed by: ____________________ Date: __________

Ann Yellowlees, Director

Quantics Consulting Ltd.

DocuSigned by:
Blair Dickman
3D5E41B77B7545B...

Approved by: ____________________ Date: 15-Nov-2021

Blair Dickman, Senior Clinical Research Manager

Douglas Pharmaceuticals Ltd.

DocuSigned by:
Jason Long
F231D0A84EB64D2...

Approved by: ____________________ Date: 15-Nov-2021

Jason Long, Clinical Research Physician

Douglas Pharmaceuticals Ltd.

Section 1: Glossary of terms

Abbreviation	Description
AE	Adverse event
ANCOVA	Analysis of covariance
BPIC-SS	Bladder Pain/Interstitial Cystitis Symptom Score
BPRS (5 items)	Brief Psychiatric Rating Scale Positive Symptom subscale
CADSS	Clinician-Administered Dissociative States Scale
CGI-S	Clinician Global impression – Severity Score
ECG	Electrocardiogram
eCRF	Electronic case report form
FA	Full analysis
ICH	International Conference on Harmonisation of Technical Requirements for Registration of Pharmaceuticals for Human Use
ITT	Intention to treat
IMP	Investigational medicinal product
LOCF	Last observation carried forward
MADRS	Montgomery-Asberg Depression Rating Scale
MAR	Missing at random
MDD	Major depressive disorder
MI	Multiple imputation
MMRM	Mixed Model Repeated Measures
MoCA	Montreal Cognitive Assessment
PGI-S	Patient Global Impression – Severity Score
PK	Pharmacokinetic
PP	Per protocol
SAE	Serious adverse event
SAP	Statistical analysis plan

SD	Standard deviation
TEAE	Treatment emergent adverse event
TRD	Treatment resistance in major depression
WOCBP	Women of childbearing potential
WSAS	Work and Social Adjustment Scale

DocuSign Envelope ID: 9580C97D-56AD-4E50-9133-FAC5390A9286

Section 2: Change control

Version	Changes
2.0	Added:<br>- Schedule of study visits tables from protocol<br>- Subgroup analyses for enrichment and randomised phase data added following sponsor request – section 10.3.4

DocuSign Envelope ID: 9580C97D-56AD-4E50-9133-FAC5390A9286

Section 3: Introduction

The purpose of this statistical analysis plan (SAP) is, as per the ICH E9 guideline and addendum [1, 2], to provide a technical and detailed elaboration of the analysis described in the study protocol [3], and to include detailed procedures for executing the statistical analysis of the primary, secondary and exploratory endpoints. The aim of the SAP is to ensure that the data listings, summary tables and figures which will be produced, and the statistical methodologies that will be used, are complete, and appropriate to allow valid conclusions regarding the study objectives.

Any amendments to the SAP will be made prior to database lock.

Any additional analyses not described in the final SAP or deviations from the final SAP will be documented in the clinical study report.

Quantics Consulting Limited will perform the statistical analyses and are responsible for the production and quality control of all tables, listings, and figures.

Section 4: Study overview

4.1 Study objectives

The primary objective of the study is to evaluate the efficacy of extended release (ER) R-107 tablets (30 mg, 60 mg, 120 mg, 180 mg) as measured by the change in Montgomery-Asberg Depression Rating Scale (MADRS) score from baseline (Day 1) to Day 92 in subjects with Diagnostic and Statistical Manual of Mental Disorders, 5th edition (DSM-5) Treatment-Resistant Depression (TRD) who have responded to 1 week's treatment with R-107 120 mg tablets.

4.2 Study design

This is a multi-centre, phase 2a study, which incorporates a 1-week enrichment open-label phase followed by a randomised, double-blind, placebo-controlled treatment phase, to investigate R-107 (30 mg, 60 mg, 120 mg or 180 mg) versus placebo in TRD subjects who respond to the 1-week enrichment open-label phase.

4.3 Determination of sample size

The sample size calculation is based on superiority of R-107 to placebo by a magnitude of 6 MADRS units, using a standard deviation of change in MADRS of 7.5 units, a two-sided Type 1 error of 0.05, and a power of 80%. Assuming a closed testing procedure whereby each dose group is compared with the placebo group in descending order, 26 subjects per group are required to achieve this. Allowing for a 13% drop-out rate and an attrition rate of 25% during the enrichment open-label phase, a total of around 200 subjects will be required initially in order to randomise 150 subjects to five treatment groups at the start of the double-blind randomised treatment phase.

4.4 Treatment assignment and administration of study medication

Subjects who meet the eligibility criteria will receive R-107 120 mg once a day for five days during the enrichment open-label phase, followed by two days of no treatment. Amongst subjects who respond to the treatment during the enrichment open-label phase, more than 150 will be randomised for the double-blind randomised treatment phase for 12 weeks. They will be randomised on a 1:1:1:1:1 basis, to receive Placebo, R-107 30 mg, R-107 60 mg, R-107 120 mg, or R-107 180 mg twice weekly. Subjects will be blinded to their group allocation.

Treatment groups will be stratified by study site. There are 15 sites in total, and each site is expected to enrol 13-14 patients in the open-label phase and randomise 10 patients in the double-blind phase.

The study medication is self-administered orally in the form of tablets. During the open-label phase, all medication will be taken at the site. During the double-blind phase, medication will be dispensed at six study visits and at two pharmacy visits. During these six study visits, one dose will be self-administered at the site, and subsequent doses will be taken by the patient at home. All medication received at pharmacy visits will be taken at home.

4.5 Study visit schedule

Figure 1 and Figure 2 show the study schedule for the enrichment and randomised phases.

Figure 1: Study Schedule – Part 1 (Enrichment Open-Label R-107 120 mg QD x 5D)

	Screening	Treatment		Early Termination (a)
		Baseline	Dosing	
	Day -28 to -1	Day 1	Day 2-5	
Informed consent	X			
Inclusion/exclusion criteria	X	X		
Demographics and medical history	X			
Medication history	X			
Physical examination	X			
Vital signs (b)	X	X	X	X
Weight, height (c)	X	X		X
Concomitant medications (d)	X	X	X	X
Concurrent medical conditions	X	X		
Clinical laboratory tests (e)	X	X		X
HIV/Hepatitis panel	X			
Serum Pregnancy test (f)	X			
Urine Pregnancy test (f)		X		
FSH (g)	X			
Urine drug screen/Alcohol breath test	X	X		
Safety ECG (h)	X	X		X
MINI	X			
MSM	X			
MoCA (i)	X			X
MADRS	X	X[m]	X[o]	X
CADSS	X	X[n]	X	X
eC-SSRS (j)	X	X[m]		X
BPIC-SS	X	X[m]		X
PGI-I	X	X[m]		X
PGI-S	X	X[m]		X
CGI-S	X	X[m]		X
WSAS	X	X[m]		X
EQ-5D-5L	X	X[m]		X
BPRS+	X	X[n]		X
Verbal Fluency		X[m]		X
SDMT		X[m]		X
Study Drug Dosing		X	X	
PTE assessment (k)	X	X		
AE assessment (l)		X	X	X

(a) Conduct procedures for subjects discontinued early.
(b) Vital signs (oral temperature, pulse rate [radial], respiratory rate, blood oxygen saturation and BP) will be obtained at screening, during each treatment period prior to and 30 minutes after dosing, and Early Termination, as appropriate.
(c) Height collected only at screening.
(d) Record all ongoing medications and non-pharmacologic therapies.
(e) Clinical laboratory tests (haematology, serum chemistry, urinalysis) will be collected at screening, Day 1 and 8, and Early Termination, as appropriate.
(f) For subjects of childbearing potential only.
(g) An FSH level will be obtained for post-menopausal subjects (defined as continuous amenorrhea >12 months and not surgically sterile).
(h) Safety ECG will be measured by standard stationary 12-lead ECG machines. ECG will be recorded at screening, Day 1 (pre-dose [within 30 minutes prior to dosing], and at Day 8, and Early Termination, as appropriate.
(i) The administration sequence of the MoCA assessment will be from Version 8.1 to Version 8.2 to Version 8.3, back to Version 8.1 and so on. The version of the MoCA assessment used at the specified study visit should differ from and will depend on the last version of MoCA administered to the subject.
(j) eC-SSRS screening version to be used at screening and "since last visit" version for all others.
(k) Pre-treatment events (PTEs) will be collected from signing of informed consent until dosing on Day 1 of the first treatment period.
(l) Any event after dosing on Day 1 of the first treatment period will be captured as an AE.

DocuSign Envelope ID: 9580C97D-56AD-4E50-9133-FAC5390A9286

(m) Questionnaires will be completed pre-dose on Day 1 visit to ensure a valid baseline measurement prior to any drug dosing.
(n) CADSS and BPRS+ assessments will be performed pre-dose and 2 hours after dosing
(o) MADRS to be completed on Day 5 only

Figure 2: Study Schedule – Part 2 (Randomisation, double-blind treatments)

	Randomisation	Treatment							Early Termination (a)	Safety Follow-up (g) (±5)
		Dosing					Check-out			
	Day 8	Day 9	Day 15, 22, 29	Day 36	Day 64 (±5)	Day 65	Day 92 (±5)	Day 93		
Physical examination	X		X	X	X		X		X	X
Vital signs (b)	X		X	X	X		X		X	X
Weight			X	X	X		X		X	X
Concomitant medications (c)	X		X	X	X		X		X	X
Clinical laboratory tests (d)	X		X	X	X		X		X	X
Urine Pregnancy test (e)					X		X		X	X
Safety ECG (f)	X			X	X		X		X	X
MoCA (k)	X			X	X		X		X	X
MADRS	X		X	X	X		X		X	X
CADSS	X		X	X	X		X		X	X
eC-SSRS	X		X	X	X		X		X	X
BPIC-SS	X		X	X	X		X		X	X
PGI-I	X		X	X	X		X		X	X
PGI-S	X		X	X	X		X		X	X
CGI-S	X		X	X	X		X		X	X
WSAS	X		X	X	X		X		X	X
EQ-5D-5L	X		X	X	X		X		X	X
BPRS+	X		X	X	X		X		X	X
Verbal Fluency	X		X	X	X		X		X	X
SDMT	X		X	X	X		X		X	X
Study Drug Dosing and Dispensing (j)	X		X	X	X		X			
AE assessment	X		X	X	X		X		X	X
PK sample collection	X[i]	X[h]			X[i]	X[h]	X[i]	X[h]		

(a) Conduct procedures for subjects discontinued early.
(b) Vital signs (oral temperature, pulse rate [radial], respiratory rate, blood oxygen saturation, and BP) will be obtained at Enrolment, during each treatment period prior to and 30 minutes after dosing, and Early Termination, as appropriate.
(c) Record all ongoing medications including non-pharmacologic therapies.
(d) Clinical laboratory tests (haematology, serum chemistry, urinalysis) will be collected at Enrolment, during each treatment period, and Early Termination, as appropriate.
(e) For subjects of childbearing potential only.
(f) Safety ECG will be measured by standard stationary 12-lead ECG machines pre-dose on Days 8, 36, 64, 92, at follow-up safety visit and Early Termination. ECG will also be measured 4 hours post-dose on Days 8, 64 and 92. This will be completed in conjunction with PK sample collections.
(g) A safety follow-up visit to be conducted 28 (±5) days after the subject's last dose of study drug.
(h) PK 24hr sample collection.
(i) PK plasma samples to be collected pre-dose and 4hrs post-dose.
(j) Reconciliation of drugs at each visit. There is a window of +24 hours for a missed dose in the Part 2. Subjects will be asked to return used and unused bottles of drugs given at prior visit to ensure subject compliance. Subjects will have scheduled Pharmacy Visits on Day 50 and Day 78 for study drug dosing and dispensing.
(k) The administration sequence of the MoCA assessment will be from Version 8.1 to Version 8.2 to Version 8.3, back to Version 8.1 and so on. The version of the MoCA assessment used at the specified study visit should differ from and will depend on the last version of MoCA administered to the subject.

QUANTICS

4.6 Eligibility criteria

The eligibility criteria are described in the study protocol [3].

4.7 Additional data from Singapore and Taiwan

Subjects from sites in Singapore and Taiwan will have additional measurements to the ones shown in the schedule (Section 4.5). The additional data will not be summarised in tables. However, they will be contained in all relevant listings.

4.8 Changes to SAP

Any deviations from this SAP will be fully documented in the clinical study report.

Section 5: Study endpoints

5.1 Primary endpoint

The primary endpoint is:

- Change in Montgomery-Asberg Depression Rating Scale (MADRS) total score from baseline (Day 1) to Day 92.

5.2 Secondary endpoints

The secondary endpoints are:

- Change in MADRS total score from baseline (Day 1) to Day 36 and Day 64;
- Proportion of relapsed subjects (MADRS ≥ 22) at Day 36, Day 64, Day 92;
- Time to relapse (MADRS ≥ 22);
- Proportion of subjects with response at Day 36, Day 64 and Day 92, where response is defined as ≥50% reduction from baseline (Day 1) in MADRS total score;
- Proportion of subjects in remission at Day 36, Day 64 and Day 92, where remission is defined as MADRS total score ≤10;
- CGI-S score and PGI-I score at Day 92;
- Pharmacokinetics (PK) of R-107 tablets (to be covered by a separate stand-alone SAP);
- Dose-response pattern for R-107 with respect to the primary efficacy outcome (MADRS) and safety outcomes.

5.3 Exploratory endpoints

The exploratory endpoints are:

- PGI-S score at Day 92;
- EuroQol-5D, 5-level version (EQ-5D-5L) questionnaire scores at Day 92;
- EuroQol-5D: EQ VAS change from baseline to Day 92;
- WSAS score at Day 92.

5.4 Safety endpoints

The safety endpoints are:

- Part 1: Enrichment Open-label Phase
    - Adverse Events (AEs);
    - Summary of heart rate, blood pressure, respiratory rate, temperature, and blood oxygen saturation (SpO2);
    - Summary of laboratory evaluations;
    - Summary of weight and BMI.
- Part 2: Randomised Double-Blind Treatment Phase
    - AEs;
    - Summary of heart rate, blood pressure, respiratory rate, temperature, and blood oxygen saturation (SpO2) for all available time points, including change from baseline;
    - Summary of laboratory evaluations, ECG, and physical examination for all available time points, and change of continuous laboratory evaluations from baseline to Day 36, Day 64, and Day 92;
    - Summary of weight and BMI for all available time points;
    - Change of eC-SSRS Suicidal Ideation Score and Suicidal Behaviour Score from baseline to Day 8, Day 36, Day 64, and Day 92;
    - Change of BPRS (5 items) total score from baseline to Day 8, Day 36, Day 64, and Day 92;
    - Change of CADSS total score and sub-scores for amnesia, depersonalisation and derealisation from baseline to Day 8, Day 36, Day 64, and Day 92;
    - Change of BPIC-SS total score from baseline to Day 8, Day 36, Day 64, and Day 92;
    - Change of Verbal Fluency scores (category and letter) and SDMT scores from baseline to Day 92.

5.4.1 Data safety monitoring committee (DSMC) meetings

The DSMC will formally meet at the following recruitment milestones:

- When 20 patients have completed the enrichment phase
- When 50 patients have completed the enrichment phase
- When 100 patients have completed the enrichment phase
- When 150 patients have completed the enrichment phase
- Quarterly until the last patient enrolled has completed the randomised, placebo-controlled phase

The following data will be summarised in the open-session and closed-session DSMC reports:

	Open report (pooled)	Closed report (by group)
Disposition, baseline characteristics, and conduct		
Subjects screened and randomised	✓	✓
Reason for ending study drug early	✓	✓
Demographics	✓	✓
Disease characteristics	✓	✓
Safety		
Adverse events (including all, serious, leading to discontinuation, fatal, related, and events of interest)		✓
Changes in vital signs		✓
Changes in laboratory evaluations		✓
Physical examination		✓
Suicidal ideation / behaviour (eC-SSRS)		✓
Changes in scale scores (i.e. BPRS, CADSS, BPIC-SS)		✓
Changes in congnitive function (MoCA, DSMT, verbal fluency)		✓
Efficacy		

	Open report (pooled)	Closed report (by group)
MADRS		✓

As part of the DSMC data review, patients' withdrawal by treatment group will also be reviewed.

Section 6: Analysis populations

The following three populations will be used for analysis.

6.1 Full analysis population

The full analysis population will be defined as the intention-to-treat (ITT) set and will include all randomised subjects.

6.2 Per protocol population

The per protocol population will be a subset of the full analysis population and is intended to fulfil the following:

- All inclusion/exclusion criteria satisfied;
- Absence of protocol violations that are likely to affect the efficacy of treatment;
- Adequate study medication compliance;
- Adequate measurement of the primary variable.

In order to select the per protocol population, the Sponsor will receive a blinded list of subjects with the following information:

- Alcohol and drug abuse at time of screening and baseline; flagged if “current” or “former” for any alcohol or drug at any of the two time points
- MADRS total score at screening and baseline; flagged if <20
- Overall compliance; flagged if < 80%
- Compliance: maximum of returned tablets by visit during treatment time; flagged if at least 6 tablets are returned
- Early withdrawal information; flagged if the subject did not complete the study
- Protocol deviations
- Medication captured in Form “Medical History”; flagged if ATC codes start with N06A (to assess whether new antidepressants were started), or flagged if they correspond to any of the medications shown as disallowed in Section 6.2, Table 7
- Number of days between first and last total MADRS scores were recorded; flagged if at least two between Day 8 and Day 92 are missing

The Sponsor will review the list and confirm final selection criteria for the PP population. Based on the final criteria, Quantics will produce a list of subjects to be excluded from the PP population.

6.3 Safety population

6.3.1 Open-label safety population

The open-label safety population will comprise all subjects who received at least one administration of the study medication during the open-label phase.

6.3.2 Double-blind safety population

The double-blind safety population will comprise all subjects who received at least one administration of the study medication during the double-blind randomised phase. Treatment group will be the actual treatment received if randomisation was incorrectly applied.

6.4 Enrolled population

The enrolled population will comprise all subjects who signed the informed consent form.

6.5 Use of study populations

The primary endpoint will be analysed using both the FA and PP population if numbers permit. All secondary and exploratory endpoints will be analysed using the FA population.

Analyses of safety endpoints will be based on the open-label safety population and on the double-blind safety population.

Listings will be mostly based on the enrolled population, as long as data collection extends to all enrolled subjects.

Section 7: General points for statistical analysis

All statistical analyses as covered by this SAP will be performed using R version 3.4.1 or later [4] and SAS® version 9.4 [5].

7.1 General methods

All summaries and listings will be presented by treatment group. Variables will be summarised as follows.

Continuous variables:

- number of observations
- mean and standard deviation
- median, minimum, and maximum
- number of missing observations

Categorical variables:

- frequencies and percentages

7.1.1 Decimal places

- The mean and median for a set of values will be presented to 1 more decimal place than the original values.
- Standard deviations will be presented to 2 more decimal places than the original values.
- The minimum and maximum will report the same number of decimal places as the original values.
- Percentages will be displayed with 1 decimal place; percentages will not be presented when the count is zero; and 100% will be presented as an integer. Note that for categorical data, the number of patients with missing data will be included in the calculations.
- P-values will be displayed with 3 decimal places.

7.2 Definition of baseline

For the purpose of reporting, the baseline value will be defined as the value recorded on the Day 1 visit if it exists. If a value is only recorded at screening and not at Day 1, the value at screening will be taken as the baseline value. Any other value is not a baseline value.

7.3 Withdrawals and missing data

DocuSign Envelope ID: 9580C97D-56AD-4E50-9133-FAC5390A9286

7.3.1 Withdrawals

Completion of full study will be determined for the FA population using the “End of Study” form of the eCRF with value “yes” recorded in variable “Did the patient complete the study?”. If the value is not set to “yes”, the subject is considered to have withdrawn.

The extent of the follow-up for each patient (i.e. the time from randomization to end of study) will be assessed for each group using a Kaplan-Meier plot with “Withdrawal” from treatment as the event. If there are clear differences between the groups in terms of the time in study this may suggest a differential treatment effect and render the assumptions behind the proposed primary analysis (MMRM) untenable since the use of MMRM relies on certain assumptions about the missing data pattern. This is also discussed in section 6.3.2 (Missing data).

7.3.2 Missing data

Unless otherwise explicitly stated, missing data will not be imputed.

For the primary endpoint, a “last observation carried forward” (LOCF) approach will be utilised for missing day 92 MADRS scores under the assumption that this is a conservative imputation that will also ensure the main analysis of the primary endpoint is not left unanalyzable due to a high relapse rate (see also section 10.1.1). An analysis using the LOCF approach assumes that missing values are identical to the last recorded value and that no further change in any direction would be expected after that point, had the patient not withdrawn.

To further explore the impact of missing values for the primary endpoint, the mixed model repeated measures (MMRM) approach will be used for data with no imputation, which gives consistent estimates if values are missing at random (MAR). If the values are not missing at random, the impact is unclear. An analysis using the same model only with a multiple imputation (MI) approach to missing values will also be conducted if feasible. This method attempts to predict the missing values based on previously recorded data. The pattern of missing data for the primary outcome (MADRS) will be investigated by plotting mean responses over time for patients grouped by their time in trial (i.e. timing of final visit).

7.3.3 Mapping of dates to study days

If measurements are reported by date rather than day within the treatment time, the day within the treatment time will be calculated using the date of the first visit day (Day 1) as reference. If the calculated day does not correspond to any of the time slots as reported in the visit schedule (including margins), the date will be queried. If the date cannot be corrected to correspond to a time slot in the visit, the data for the visit day will be discarded.

Section 8: Disposition and baseline characteristics

8.1 Subject disposition and withdrawals from treatment

The following will be listed and tabulated over all screened subjects:

- Number of subjects screened
- Number (% of screened) of subjects withdrawing before enrichment open-label phase
- Number (% of screened) of enrolled subjects
- Number (% of screened) of subjects in open-label safety population
- Number (% of open-label safety) of subjects in FA population, by randomised treatment and total
- Number (% of open-label safety) of subjects in double-blind safety population, by actual treatment and total
- Number (% of FA population in group) of subjects in FA who complete the treatment period, by randomised treatment and total
- Number (% of FA population in group) of subjects in FA who withdraw during the treatment period, by reason of withdrawal, by randomised treatment and total
- Number (% of FA population in group) of subjects in PP population, by actual treatment and total

8.2 Demographics and baseline characteristics

The following demographic and clinical characteristics will be listed and tabulated for the FA population:

- Summary using summary statistics for continuous variables:
  - Age
  - Weight by gender
  - BMI by gender
- Summary using summary statistics for categorical variables:
  - Gender
  - Ethnicity
  - Race
  - Past/concomitant diseases or past surgeries, grouped by body system group (SOC) (yes/no)
  - Substance use (never/current/former) by substance category

- ECG (normal/abnormal and not clinically significant/abnormal and clinically significant/abnormal and clinical significance missing/unable to evaluate or missing)
- Physical examination (normal/abnormal and not clinically significant/abnormal and clinically significant/abnormal and clinical significance missing/not done or missing) by body system category

Section 9: Compliance with study product

The compliance rate (%) per subject is based on the dispensed and returned number of tablets of study product. Compliance will only be measured for the randomised double-blind treatment phase, since the administration of the study product during the open-label phase will only take place during visits to the study site.

Two bottles per week will be used, translating into six tablets per week. For time points S < T where S and (T + 1) are adjacent study days in 8/15/22/29/36/50/64/78/92, it is expected that 6/7 * (T + 1 – S) tablets are consumed provided no withdrawal happened before or at T. For study days S in 8/15/22/29/36/64/92, three of these tablets will be taken at the study site on study day S, and the rest will be dispensed. For study days S = 50 and S = 78 pharmacy visits are scheduled. No tablets will be taken on site, and all tablets will be dispensed. Dispensed/returned tablets will be recorded in form "Drug Accountability", while administered drugs at the study site will be recorded in form "Dosage".

If a "time of dosage" on the "Dosage" form is given with the date of study day S, it will be assumed that all three tablets were administered at visit S. Otherwise, it will be assumed that at visit S no tablets were administered. In particular, one cannot distinguish between missing information and "no tablets given at visit S", and missing values will be interpreted as "no tablets".

In the event of withdrawal before or at T, in the absence of a daily pattern it is unclear how many doses of study product were expected to be consumed up to the point of withdrawal. Compliance between S and T is therefore calculated as follows:

- Compliance S:T = ("Number of tablets dispensed at S" – "number of tablets returned at (T + 1)" + "number of tablets administered at S") / {6/7 * (T + 1 - S)}, if drug accountability forms are completed for both time points S and (T + 1);
- Compliance S:T = missing, if drug accountability forms are not completed for at least one of the time points S and (T + 1).

Regarding the last day of dosing, compliance is calculated as follows:

- Compliance at Day 92 is 100% if a "time of dosage" is given on the dosage form, dated with Day 92;
- Compliance at Day 92 is 0% if there is no dosage form completed for Day 92 including "time of dosage", and the subject has not withdrawn early.

Overall compliance is calculated as:

DocuSign Envelope ID: 9580C97D-56AD-4E50-9133-FAC5390A9286

- Overall compliance = (Sum of number of tablets dispensed during the trial period – sum of number of tablets returned during the trial period + sum of number of tablets administered during visits) / {6/7 * 84 + 1}, if not withdrawn early from study and all drug accountability forms are completed;
- Overall compliance = ("Sum of number of tablets dispensed before time point T" – "sum of number of tablets returned up to time point T " + "sum of number of tablets administered during visits up to time point T") / {6/7 * (T – 8) + "number of tablets expected to be administered at time point T"}, if T is the last day in the above list before or at the day of withdrawal and all drug accountability forms up to T are completed.
- Overall compliance = missing, if there is a drug accountability form prior to treatment termination (Day 92 or Early Withdrawal, whichever applies) which is not completed.

Compliance rates for adjacent study days from the list above and overall compliance rates will be listed and tabulated for the FA population.

Summary statistics for the nine separate compliance rates will be tabulated by group using summary statistics for continuous variables. In addition, the number and percentage of subjects with compliance in the following bands will be summarised for each compliance rate:

- < 80%
- 80% - ≤ 100%
- > 100%

DocuSign Envelope ID: 9580C97D-56AD-4E50-9133-FAC5390A9286

Section 10: Analysis of study endpoints

10.1 Primary endpoint

The primary efficacy endpoint is change in MADRS total score from baseline (Day 1) to Day 92.

10.1.1 Analysis of the primary endpoint

The primary endpoint will be analysed using an analysis of covariance (ANCOVA). The analysis will be based on differences in MADRS at Day 92 from baseline MADRS, with dose as factor and baseline MADRS as covariate. No values from other time points will be used for this model. This will be based on the FA population.

Missing values for the Day 92 MADRS score will be imputed using the last available MADRS score (LOCF). If it is assumed that more relapses will occur in the placebo group, and that relapsed patients would have deteriorated further had they remained in the study, then this imputation method is conservative in terms of the estimation of a treatment effect.

The primary endpoint for dose groups as stated above will be compared. The following estimated marginal means of differences and their 95% confidence interval at Day 92 will be presented:

- R-107 180 mg vs. placebo
- R-107 120 mg vs. placebo
- R-107 60 mg vs. placebo
- R-107 30 mg vs. placebo

10.1.1.1 Significance levels and multiplicity

The aim of the study is to compare R-107 with placebo. The overall significance level will be at a two-sided 5.0% level. To control family-wise type I error, a fixed sequence step-down closed test procedure will be used for hypothesis testing. Starting with the highest dose, R-107 180 mg will be compared with placebo as the first step. Only if R-107 180 mg is statistically significantly different at the two-sided 5.0% level will R-107 120 mg be compared with placebo. Only if the R-107 120 mg dose group is statistically different to placebo will R-107 60 mg be compared with placebo. Only if the R-107 60 mg dose group is statistically different to placebo will R-107 30 mg be compared with placebo. A two-sided significance level of 5.0% will be used for each comparison. The primary analysis set for this analysis is the FA population.

10.1.2 Sensitivity analyses for primary endpoint

Regarding the primary endpoint, the following sensitivity analyses will be undertaken:

1. Repeat the main analysis with the PP population instead of the FA population.
2. Analyse the MADRS scores for each patient across visits using the mixed model repeated measures (MMRM) method. It will be based on the FA population.

The details for the MMRM are as follows.

- Dependent variable: baseline MADRS – MADRS (i.e., difference of MADRS score from baseline)
- Factors:
  - Dose (categorical, fixed effect): 0/30/60/120/180
  - Time (categorical, fixed effect): Days 8/15/22/29/36/64/92
  - Interaction of dose and time (categorical, fixed effect)
  - Country (categorical, fixed effect)
  - Subject (categorical, random effect)
- Covariates:
  - Baseline MADRS
- Link function: identity
- Correlation structures: independent, AR(1), unstructured

The above specifications give rise to three models, one for each correlation structure. All three models will be run, and the models will be compared with a likelihood ratio test. The more complex model will be rejected if the p value is at least 0.05. The p values for the likelihood ratio tests for the two model comparisons (independent vs AR(1); AR(1) vs unstructured) will be reported.

The primary endpoint for dose groups as stated above will be compared. The following estimated marginal means of differences and their 95% confidence interval at Day 92 will be presented:

- R-107 180 mg vs. placebo

- R-107 120 mg vs. placebo
- R-107 60 mg vs. placebo
- R-107 30 mg vs. placebo

No imputations will be made for missing data.

Changes from day 8 will be estimated from the model and presented as in-text tables.

3. Use multiple imputation (MI) to accommodate missing values instead of dropping them. Imputations will be made for any missing data which are used in the MMRM model as described above, using all other variables of the model (dose, time, interaction of dose/time, subject, and baseline MADRS score) as predictors. It is, however, expected that only differences of MADRS scores from baseline MADRS scores are potentially missing. Predictive mean matching will be used to impute values, and in total 10 data sets with imputed data will be generated. Pooled results of the 10 datasets will be displayed.

10.2 Secondary endpoints

10.2.1 Change in MADRS total score from baseline to Day 36 and Day 64

The MMRM model as selected in the sensitivity analysis of the primary endpoint (Section 10.1.2) and the MI approach as outlined in Section 10.1.2 will be used to display this endpoint, based on the FA population. No imputations of missing values will be made.

The following estimated marginal means of differences and their 95% confidence interval at Days 36 and 64 will be presented:

1. R-107 180 mg vs. placebo
2. R-107 120 mg vs. placebo
3. R-107 60 mg vs. placebo
4. R-107 30 mg vs. placebo

10.2.2 Relapse at Days 36, 64, and 92

Fisher's exact test of proportions will be used to compare the proportions of patients in the FAS that have relapsed by each time point (Day 36, Day 64, and Day 92) for each active treatment group against the placebo group.

10.2.3 Time to relapse

Relapse is defined as a total MADRS score ≥22. Patients that do not relapse during the study, or who withdraw for other reasons are censored. Kaplan-Meier analysis will be conducted for each treatment group and a restricted mean survival time (RMST) will be estimated for time to relapse from randomization to Day 92, using the FAS. Differences in RMST for each active treatment group versus placebo will be presented.

10.2.4 Response at Days 36, 64, and 92

Response is defined as ≥50% reduction from baseline (Day 1) in MADRS total score. For each of the Days 36, 64, and 92, a binary response variable will be created for each subject, indicating whether the subject was a responder at that point in time. Withdrawn subjects or subjects with missing values will be counted as non-responders. The number and proportion of responses for each treatment group will be tabulated for each time point (Day 36, Day 64 and Day 92). At each time point separately, the response rate for each dose of R-107 will be compared with that of placebo for all subjects in the FAS using a Fisher's exact test of proportions.

10.2.5 Remission at Days 36, 64, and 92

Remission is defined as a MADRS total score ≤10. A binary remission variable will be derived for each subject at the Day 36, Day 64 and Day 92 time points. Subjects who have withdrawn or have missing values will be regarded as failing to achieve remission. The number and proportion of subjects achieving remission for each treatment group will be tabulated for each time point (Day 36, Day 64 and Day 92). At each time point separately, the remission rate for each dose of R-107 will be compared with that of placebo using a Fisher's exact test of proportions.

10.2.6 Questionnaire data: CGI-S and PGI-I

The categorical scores from the CGI-S and PGI-I will be tabulated, showing frequency counts and percentages for the FA population at baseline (Day 1) and all other days beyond Day 1 where the data was collected. Scores for Day 92 will be assessed for treatment effects using a proportional odds model for the FA population, simultaneously for all treatment groups.

To test appropriateness for the proportional odds model, a multinomial model will be fitted as well, and a likelihood ratio test between these two models will be performed. If the p-value of the test is above 0.05, then no evidence has been found to dismiss the proportional odds model. If a proportional odds model can be fitted, but a multinomial model cannot be fitted (e.g., due to numerical instabilities), then this will also be interpreted as having found no evidence to dismiss the model.

If a proportional odds model cannot be fitted, categories will be combined, or four proportional odds models will be set up by active treatment group, comparing only one active group to placebo.

A shift table without statistical testing will be produced if a proportional odds model cannot be fitted, or if the p-value of the likelihood ratio test is 0.05 or below.

Results will be expressed as odds ratios with associated 95% confidence intervals. Odds ratios greater than one will signify that the odds of a lower score relative to a higher score (i.e. greater improvement) are higher for the active treatment than for the placebo.

10.2.7 Pharmacokinetic (PK) level of R-107

This will be covered by a stand-alone PK Statistical Analysis Plan.

10.2.8 Dose-response pattern and time-response pattern for R-107 with respect to the primary efficacy outcome (MADRS) and safety outcomes

10.2.8.1 Primary efficacy outcome

The endpoints which will be plotted for Days 8, 15, 22, 29, 36, 64, and 92 are:

1. Estimated marginal means of change in MADRS scores from baseline (see Section 10.1.1), including 95% confidence intervals (dose-response and time-response)
2. Observed MADRS scores (dose-response and time-response)

10.2.8.2 Safety outcomes

1. Vital signs (see Section 10.4.2) at pre-/post-dose at Days 8, 15, 22, 29, 36, 64, and 92, for all six categories (systolic blood pressure, diastolic blood pressure, pulse rate, temperature, respiratory rate, O2 saturation) (time-response)
2. Vital signs, change from baseline for pre-/post-dose readings at Day 92 (dose-response)
3. BPIC total score to assess the effect of dose on bladder problems at Days 8, 15, 22, 29, 36, 64, and 92 (dose-response and time-response)
4. Verbal fluency and SDMT scores at Days 8, 15, 22, 29, 36, 64, and 92 (time-response)
5. Laboratory values at Days 8, 15, 22, 29, 36, 64, and 92 (time-response)

10.3 Exploratory endpoints

10.3.1 Questionnaire data: PGI-S

The categorical scores from the PGI-S will be tabulated, showing frequency counts and percentages for the FA population at baseline (Day 1) and any other days where measurements are available. Scores for Day 92 will be assessed for treatment effects using a proportional odds model for the FA population.

The same rules for testing the appropriateness of a proportional odds model as described in Section Questionnaire data: CGI-S and PGI-I10.2.6 will apply. Results will be expressed as odds ratios with associated 95% confidence intervals. Odds ratios smaller than one will signify a higher probability for the active treatment group to belong to a lower category. A shift table without statistical testing will be produced if a proportional odds model cannot be fitted or found to be not appropriate.

10.3.2 Questionnaire data: EQ-5D-5L

The EQ-5D-5L questionnaire comprises the following descriptive dimensions: mobility, self-care, usual activities, pain/discomfort, and anxiety/depression.

The categorical scores from the descriptive dimensions will be tabulated, showing frequency counts and percentages for baseline (Day 1) and Days 15/22/29/36/64/92/120 and early termination. For non-baseline days, overall health states across dimensions will be characterised and calculated as follows:

1. Better: the health state is better in at least one dimension, and worse in no dimension when compared to baseline values
2. Worse: the health state is worse in at least one dimension, and better in no dimension when compared to baseline values
3. Same: no change in any dimension when compared to baseline values
4. Mixed: the health state is better in at least one dimension and worse in at least one dimension when compared to baseline values

In addition to the descriptive dimensions, the questionnaire contains the “EQ VAS” dimension, which measures overall health on the day on a scale from 0 to 100. This value will be tabulated, showing mean and standard deviation as well as median, minimum, and maximum for the FA population and for baseline (Day 1) and Days 8/15/22/29/36/64/92/120 and early termination. For Day 92, the changes from baseline will be assessed for treatment effects using an ANCOVA with the baseline “EQ VAS” values as covariate and treatment group as factor.

Missing values will be reported as missing, no imputation will be done.

10.3.3 Questionnaire data: WSAS

For each subject, individual scores from the WSAS will be added to an overall score. The overall scores from the WSAS will be tabulated, showing mean and standard deviation as well as median, minimum, and maximum for the FA population at baseline (Day 1) and Days 8/15/22/29/36/64/92/120 and early termination. For Day 92, scores will be assessed for treatment effects using an ANCOVA with the baseline “EQ WSAS” values as covariate and treatment group as factor.

Missing values will be reported as missing, no imputation will be done. In particular, the overall score will be missing if one or more individual scores are missing.

10.3.4 Subgroup analysis

This analysis described in this section is not defined in the study protocol, and has been added to the SAP at sponsor request.

The following subgroups will be considered:

- Sex: female, male
- Age: < 65 years, ≥ 65 years
- Background antidepressant: use, non-use – where "use" is defined as taking a medication in one or more of the following categories: selective serotonin-reuptake inhibitors, serotonin-norepinephrine reuptake inhibitors, tricyclic antidepressants, monoamine oxidase inhibitors, or other oral anti-depressants.
- Baseline body weight: low, high – where "low" is defined as being at most the median (by sex) within the relevant study population.

10.3.4.3 Enrichment phase data

For the enrichment phase data there are no assigned treatment groups, therefore comparisons will only be made between levels within each subgroup categorisation.

This will be done for the open-label safety population for the following endpoints:

- Change in MADRS from baseline to day 8 – ANCOVA model including baseline MADRS score as a covariate, and the relevant subgroup as a factor. The estimate of the effect of the factor and 95% CI will be reported
- Response rate – Fisher's exact test to compare the subgroup levels
- Remission rate – Fisher's exact test to compare the subgroup levels

For the response and remission rates, the number and proportion of responses for each category within subgroup will be tabulated, and the p-value from the Fisher's test will be reported.

No imputation will be conducted for missing values.

10.3.4.4 Randomised phase

Subgroup analysis will be performed using the FA and PP populations for the analysis of:

- change in MADRS from baseline to day 92 – using method described for the primary analysis of the primary endpoint (section 10.1.1)
- time to relapse – using the method described in section 10.2.3

For each endpoint a forest plot will be produced showing the differences between the placebo group and the 180 mg group with associated 95% confidence intervals for each subgroup.

10.4 Safety endpoints

10.4.1 Adverse events

10.4.1.1 Adverse events: enrichment open-label phase

Any AEs that occur during the open-label enrichment phase (i.e., onset on the day of the first dose intake or later, but before Day 8) will be contained in a listing.

All subjects will be receiving R-107 120 mg during this phase of the study. In order to identify any potential knock-on effect on later parts of the study, the number of AEs and patients with any AEs will be tabulated for the double-blind safety population by actual treatment group as assigned later in the randomised double-blind treatment phase.

10.4.1.2 Adverse events: randomised double-blind treatment phase

Treatment emergent adverse events (TEAEs) in this section will be defined as adverse events that occurred on or after the day of first intake of the randomised study product, up to Day 120, or which occurred during the open-label phase but became more severe during the double-blind phase. For TEAEs, a summary will present the number and percentage of subjects by actual treatment group and overall with:

1. Any TEAE
2. Number of subjects reporting at least one TEAE
3. Number of subjects reporting at least one treatment-related TEAE
4. Number of subjects with TEAEs leading to discontinuation
5. Number of subjects with TEAEs leading to death
6. Number of subjects reporting at least one serious TEAE
7. Number of subjects reporting at least one serious treatment-related TEAE
8. Number of subjects by maximum severity for TEAEs
9. Number of subjects by maximum relatedness for TEAEs

A treatment-related TEAE is a TEAE with “relationship to study treatment” rated as possible, probable, or definite.

A subject with TEAEs of different severity will be summarised at the highest experienced grade of severity. Likewise, a subject with TEAEs of different relatedness to study product will be summarised at the highest experienced grade of relatedness.

A TEAE will be counted as serious if the question “Serious” in the “Adverse Events” form is answered positively.

Subjects with TEAEs leading to discontinuation will be those subjects with TEAEs who withdraw early, and the primary reason for discontinuation on the “End of study” form is given as “Adverse Event”.

Missing data will be queried. If unresolved, values will be imputed as stated in this section further below.

Missing information of intensity will be imputed with “severe”, and missing information of relatedness will be imputed with “definite”. If the start date of an AE is missing and its end date is either missing or on Day 8 or later, the event will be assumed to have started on Day 8. If the start date of an AE is missing and the end date is on Day 7 or before, the event will be assumed to have started on Day 1.

This table will be produced for the double-blind safety population, based on TEAEs starting on Day 8 or later.

In addition to the above, two more separate tables will be provided:

1. Number of subjects with TEAEs by system organ class, preferred term, and by actual treatment group, and
2. Number of subjects with TEAEs by system organ class and preferred term, by actual treatment group, and severity.

A list of subjects with TEAE will be provided, including seriousness and discontinuation flags. The lists will present values as recorded.

No statistical testing will be performed for adverse events. Kaplan-Meier plots will be produced, showing time to onset of the first TEAE by treatment group.

10.4.2 Vital signs

For the purpose of this analysis, vital signs consist of the following:

1. Pulse rate

DocuSign Envelope ID: 9580C97D-56AD-4E50-9133-FAC5390A9286

2. Systolic blood pressure
3. Diastolic blood pressure
4. Respiratory rate
5. Oral body temperature
6. Blood oxygen saturation

10.4.2.1 Vital signs: enrichment open-label phase

During the enrichment open-label phase, vital signs will be collected at Screening (one reading), Days 1 – 5 (two readings: one prior and one after dosing), and Early Termination (one reading) if appropriate.

For each of the twelve readings, vital signs will be summarised using statistics for continuous variables. Moreover, for readings at Days 2 – 5 and Early Termination the difference from the baseline value (Day 1 first reading) by subject will be summarised using statistics for continuous variables.

Statistics will be based on the open-label safety population, and no imputations of missing values will be made. Statistics by actual treatment group and overall will be produced.

10.4.2.2 Vital signs: randomised double-blind treatment phase

During the randomised double-blind treatment phase, vital signs will be collected on Days 8, 15, 22, 29, 36, 64, and 92 (two readings: one prior and one after dosing), and on Day 120 (one reading) or early termination (one reading if applicable).

For each of the sixteen readings, vital signs will be summarised using statistics for continuous variables. Moreover, the difference from the baseline value (Day 1 first reading) by subject will be summarised using statistics for continuous variables.

Statistics will be based on the double-blind safety population, and no imputations of missing values will be made. Statistics by actual treatment group will be produced. As outlined in Section 10.2.8, means of values in vital sign dimensions over time by treatment group will be plotted as well.

10.4.3 Laboratory evaluations

10.4.3.1 Laboratory evaluations: enrichment open-label phase

During the enrichment open-label phase measurements for urinalysis, haematology, and serology will be collected at Screening, Day 1, and Early Termination if applicable. If more than one reading per time point is available, the last one will be selected for analysis.

DocuSign Envelope ID: 9580C97D-56AD-4E50-9133-FAC5390A9286

For each of the three readings, measurements will be summarised using statistics for continuous and categorical variables. Moreover, for readings at Early Termination which are continuous the difference from the baseline value (Day 1 reading) by subject will be summarised using statistics for continuous variables.

Statistics will be based on the open-label safety population, and no imputations of missing values will be made. Statistics by actual treatment group and overall will be produced.

10.4.3.2 Laboratory evaluations: randomised double-blind treatment phase

During the randomised double-blind treatment phase, measurements for urinalysis, haematology, and serology will be collected on Days 8, 15, 22, 29, 36, 64, 92, and also on Day 120 or early termination (if applicable). If more than one reading per time point is available, the last one will be selected for analysis.

For each of the nine readings, measurements will be summarised using statistics for continuous variables or categorical variables as appropriate. Moreover, for all continuous measurement values the change from the baseline value (Day 1) to Day 8 and beyond by subject will be summarised using statistics for continuous variables.

Statistics will be based on the double-blind safety population, and no imputations of missing values will be made. Statistics by actual treatment group will be produced.

In addition, occurrences of significant abnormalities will be summarised by treatment group.

10.4.4 BMI

10.4.4.1 BMI: enrichment open-label phase

During the enrichment open-label phase weight will be measured at Screening, Day 1, and Early Termination if applicable. Height will be measured at Screening. Body mass index (BMI) will be derived from weight and height.

For each of the three readings, BMI will be summarised using statistics for continuous variables.

Statistics will be based on the open-label safety population, and no imputations of missing values will be made. Statistics by actual treatment group and overall will be produced.

10.4.4.2 BMI: randomised double-blind treatment phase

During the randomised double-blind treatment phase, weight will be measured on Days 15, 22, 29, 36, 64, 92, and also on Day 120 or early termination (if applicable). Body mass index (BMI) will be

QUANTICS

DocuSign Envelope ID: 9580C97D-56AD-4E50-9133-FAC5390A9286

derived from weight and height. For each of the eight readings, measurements will be summarised using statistics for continuous variables.

Statistics will be based on the double-blind safety population, and no imputations of missing values will be made. Statistics by actual treatment group will be produced.

10.4.5 Physical examination: randomised double-blind treatment phase

During the randomised double-blind treatment phase, physical examinations will take place on Days 8, 15, 22, 29, 36, 64, 92, and also on Day 120 or early termination (if applicable). Occurrences of significant abnormalities will be summarised using statistics for categorical variables.

Statistics will be based on the double-blind safety population, and no imputations of missing values will be made. Statistics by actual treatment group will be produced.

10.4.6 ECG: randomised double-blind treatment phase

For time points Day 8/36/64/92, a shift table will be produced comparing baseline categories (Day 1) to the time point.

Statistics will be based on the double-blind safety population, and no imputations of missing values will be made. Statistics by actual treatment group will be produced.

10.4.7 eC-SSRS: randomised double-blind treatment phase

Questions on the eC-SSRS are grouped as follows:

1. Suicidal ideation questions:
    a. Category 1: Wish to be dead
    b. Category 2: Non-specific active suicidal thoughts
    c. Category 3: Active suicidal ideation with any methods (not plan) without intent to act
    d. Category 4: Active suicidal ideation with some intent to act, without specific plan
    e. Category 5: Active suicidal ideation with specific plan and intent
2. Suicidal behaviour questions:
    a. Category 6: Preparatory acts or behaviour
    b. Category 7: Aborted attempt
    c. Category 8: Interrupted attempt

   d. Category 9: Actual attempt (non-fatal)

   e. Category 10: Completed suicide

3. Self-injurious behaviour without suicidal intent (yes/no)

From this, the following variables are calculated for baseline (Day 1) and for Days 36/64/92/120 and Early Termination after Day 8 if the questionnaire data is not missing:

4. **Suicidal ideation**:
   a. Equal to “yes” if at least one of the suicidal ideation questions have a “yes” answer
   b. Equal to “no” if all of the suicidal ideation questions have a “no” answer
5. **Suicidal ideation score**:
   a. Equal to 0 if all suicidal ideation questions have a “no” answer
   b. Equal to the maximum suicidal ideation category which has a “yes” answer if at least one suicidal ideation question has a “yes” answer
6. **Suicidal behaviour**:
   a. Equal to “no” if all suicidal behaviour questions have a “no” answer
   b. Equal to “yes” if at least one of the suicidal behaviour questions have a “yes” answer
7. **Suicidal ideation or behaviour**:
   a. Equal to “no” if all suicidal ideation and all suicidal behaviour questions have a “no” answer
   b. Equal to “yes” if at least one of the suicidal ideation or suicidal behaviour questions have a “yes” answer

If the eC-SSRS questionnaire is incomplete for a given time point and a given subject, all the variables will be set to missing for the time point and subject. Note that this also applies to situations where, e.g., suicidal ideation questions are answered, but suicidal behaviour information is missing.

For the randomised double-blind phase the following variables are derived, referring to **events during treatment**:

1. **Suicidal ideation during treatment**:
   a. Equal to “yes” if the suicidal ideation variable has a “yes” value for at least one of the following days: Day 36, Day 64, Day 92, and Early Termination after Day 8.

DocuSign Envelope ID: 9580C97D-56AD-4E50-9133-FAC5390A9286

b. Equal to "no" if:

i. The suicidal ideation variable has a "no" or "missing" value for all of the following days: Day 36, Day 64, Day 92, and Early Termination after Day 8, and

ii. The suicidal ideation variable is not missing for at least one of the following days: Day 36, Day 64, Day 92, and Early Termination after Day 8.

c. Equal to "missing" if all values for suicidal ideation are missing for the following days: Day 36, Day 64, Day 92, or Early Termination after Day 8.

2. **Suicidal ideation categories (categories 1 – 5) during treatment**:

a. Equal to "yes" if a "yes" answer was given for the given category and all other eC-SSRS questions were answered on at least one of the following days: Day 36, Day 64, Day 92, and Early Termination after Day 8.

b. Equal to "no" if:

i. Only "no" answers were given on any of the following days if all eC-SSRS questions were answered: Day 36, Day 64, Day 92, and Early Termination after Day 8, and

ii. At least one of the following days has a complete set of answers to all questions from the questionnaire: Day 36, Day 64, Day 92, and Early Termination after Day 8.

c. Equal to "missing" if none of the following days has a complete set of answers to all questions from the questionnaire: Day 36, Day 64, Day 92, and Early Termination after Day 8.

3. **Suicidal behaviour during treatment**:

a. Equal to "yes" if the suicidal behaviour variable has a "yes" answer for at least one of the following days: Day 36, Day 64, Day 92, and Early Termination after Day 8.

b. Equal to "no" if:

i. The suicidal behaviour variable has a "no" or "missing" value for all of the following days: Day 36, Day 64, Day 92, and Early Termination after Day 8, and

ii. The suicidal behaviour variable is not missing for at least one of the following days: Day 36, Day 64, Day 92, and Early Termination after Day 8.

c. Equal to "missing" if all values for suicidal behaviour are missing for the following days: Day 36, Day 64, Day 92, and Early Termination after Day 8.

4. **Suicidal behaviour categories (categories 6 – 10) during treatment**:

a. Equal to "yes" if a "yes" answer was given for the given category and all other eC-SSRS questions were answered on at least one of the following days: Day 36, Day 64, Day 92, and Early Termination after Day 8.

b. Equal to "no" if:

i. Only "no" answers were given on any of the following days if all eC-SSRS questions were answered: Day 36, Day 64, Day 92, and Early Termination after Day 8, and

ii. At least one of the following days has a complete set of answers to all eC-SSRS questions: Day 36, Day 64, Day 92, and Early Termination after Day 8.

c. Equal to "missing" if none of the following days has a complete set of answers to all eC-SSRS questions: Day 36, Day 64, Day 92, and Early Termination after Day 8.

5. **Suicidal ideation or behaviour during treatment**:

a. Equal to "no" if both suicidal ideation during treatment and suicidal behaviour during treatment is calculated as "no"

b. Equal to "yes" if at least one of the suicidal ideation during treatment or suicidal behaviour during treatment is calculated as "yes"

c. Equal to "missing" if the values for suicidal ideation during treatment and suicidal behaviour during treatment are missing.

6. **Self-injurious behaviour without suicidal intent during treatment**:

a. Equal to "yes" if a "yes" answer was given for "self-injurious behaviour without suicidal intent" and all other questions were answered on at least one of the following days: Day 36, Day 64, Day 92, and Early Termination after Day 8.

b. Equal to "no" if:

DocuSign Envelope ID: 9580C97D-56AD-4E50-9133-FAC5390A9286

i. Only "no" answers were given for "self-injurious behaviour without suicidal intent" on any of the following days if all eC-SSRS questions were answered: Day 36, Day 64, Day 92, and Early Termination after Day 8, and

ii. At least one of the following days has a complete set of answers to all eC-SSRS questions: Day 36, Day 64, Day 92, and Early Termination after Day 8.

c. Equal to "missing" if none of the following days has a complete set of answers to all eC-SSRS questions: Day 36, Day 64, Day 92, and Early Termination after Day 8.

For events during treatment summary statistics for categorical variables will be given by actual treatment group. Statistics will be based on the double-blind safety population, excluding subjects with missing values.

In addition to the above, further variables are derived, comparing baseline values to values during or after the randomised double-blind treatment phase. Note that the following variables are set to missing if either they cannot be calculated, or the outcome for an individual is deterministic (e.g., a state cannot be worsened). The **comparison variables** are as follows:

1. **Treatment-emergent suicidal ideation compared to baseline**:

   a. Equal to "yes" if the maximum suicidal ideation score on Days 36, 64, 92, and Early Termination after Day 8 is higher than the baseline suicidal ideation score.

   b. Equal to "no" if the maximum suicidal ideation score on Days 36, 64, 92, and Early Termination after Day 8 does not exceed the baseline ideation score.

   c. Equal to "missing" if:

      i. The baseline suicidal ideation score is missing, or

      ii. All suicidal ideation scores on Days 36, 64, 92, and Early Termination after Day 8 are missing, or

      iii. The baseline suicidal ideation score is equal to 5.

2. **Treatment-emergent serious suicidal ideation compared to baseline:**

   a. Equal to "yes" if the baseline ideation score is smaller than 4 and the maximum suicidal ideation score on Days 36, 64, 92, and Early Termination after Day 8 is at least 4.

b. Equal to “no” if the baseline ideation score is smaller than 4 and the maximum suicidal ideation score on Days 36, 64, 92, and Early Termination after Day 8 is smaller than 4.

c. Equal to “missing” if:

i. The baseline suicidal ideation score is at least 4, or

ii. The baseline suicidal ideation score is missing, or

iii. All suicidal ideation scores on Days 36, 64, 92, and Early Termination after Day 8 are missing.

3. **Emergence of serious suicidal ideation compared to baseline**:

a. Equal to “yes” if the maximum suicidal ideation score on Days 36, 64, 92, and Early Termination after Day 8 is at least 4 and the baseline suicidal ideation score is 0.

b. Equal to “no” if the maximum suicidal ideation score on Days 36, 64, 92, and Early Termination after Day 8 is 0 and the baseline ideation score is 0.

c. Equal to “missing” if:

i. The baseline suicidal ideation score is missing, or

ii. The baseline suicidal ideation score is greater than 0, or

iii. All suicidal ideation scores on Days 36, 64, 92, and Early Termination after Day 8 are missing.

4. Improvement in suicidal ideation at Day 92 compared to baseline:

a. Equal to “yes” if the suicidal ideation score on Day 92 is less than the baseline suicidal ideation score.

b. Equal to “no” if the suicidal ideation score on Day 92 is greater than or equal to the baseline suicidal ideation score.

c. Equal to “missing” if:

i. The baseline suicidal ideation score is missing, or

ii. The baseline suicidal ideation score is 0, or

iii. The suicidal ideation score on Day 92 is missing.

5. Improvement in suicidal ideation at Day 120 compared to baseline:

DocuSign Envelope ID: 9580C97D-56AD-4E50-9133-FAC5390A9286

   a. Equal to “yes” if the suicidal ideation score on Day 120 is less than the baseline suicidal ideation score.
   b. Equal to “no” if the suicidal ideation score on Day 120 is greater than or equal to the baseline suicidal ideation score.
   c. Equal to “missing” if:
      i. The baseline suicidal ideation score is missing, or
      ii. The baseline suicidal ideation score is 0, or
      iii. The suicidal ideation score on Day 120 is missing.

6. **Emergence of suicidal behaviour compared to baseline**:
   a. Equal to “yes” if suicidal behaviour during treatment is equal to “yes” and the baseline suicidal behaviour is equal to “no”.
   b. Equal to “no” if suicidal behaviour during treatment is equal to “no” and the baseline suicidal behaviour is equal to “no”.
   c. Equal to “missing” if:
      i. The baseline suicidal behaviour is missing, or
      ii. The baseline suicidal behaviour is greater than 0, or
      iii. The suicidal behaviour during treatment is missing.

For the comparison variables, summary statistics for categorical variables will be given by actual treatment group. Statistics will be based on the double-blind safety population, excluding subjects with missing values.

10.4.8 CADSS: randomised double-blind treatment phase

The CADSS questionnaire consists of 27 items, and each is scored using values 0 – 4. Only the first 19 items will be analysed. For each subject, all individual question scores from the first 19 items will be summed up to a total score.

1. Amnesia score: Sum of scores from items 14 and 15
2. Depersonalization score: sum of scores from items 3 – 7
3. Derealisation score: sum of scores from items 1, 2, 8 – 13, 16 – 19

4. Total score: sum of scores from items 1 - 19

For the baseline (Day 1 prior to dosing) and for Days 8/36/64/92, scores from the above list will be summarised using statistics for continuous variables. Moreover, the change of values from Day 8 and beyond from the baseline value (Day 1) by subject will be summarised using statistics for continuous variables.

Statistics will be based on the double-blind safety population, and no imputations of missing values will be made. In particular, a missing item will lead to a missing overall score. Statistics by actual treatment group will be produced.

10.4.9 BPRS (5 items): randomised double-blind treatment phase

Five items from the BPRS scale will be analysed, each of which are scored using values 1 – 7. For each subject, all individual question scores will be summed to a total score.

The 5 items from the BPRS are: conceptual disorganization, suspiciousness, hallucinatory behaviour, unusual thought, and grandiosity.

For the baseline (Day 1) and for Days 8/36/64/92, total scores will be summarised using statistics for continuous variables. Moreover, the change of the values from Day 8 and beyond from the baseline value (Day 1) by subject will be summarised using statistics for continuous variables.

Statistics will be based on the double-blind safety population, and no imputations of missing values will be made. In particular, a missing item will lead to a missing overall score. Statistics by actual treatment group will be produced.

10.4.10 BPIC-SS scores: randomised double-blind treatment phase

The BPIC-SS score contains eight questions with varying score ranges. For each subject, all individual question scores will be summed to a total score.

For the baseline (Day 1) and for Days 8/36/64/92, total scores will be summarised using statistics for continuous variables. Moreover, the change of the values from Day 8 and beyond from the baseline value (Day 1) by subject will be summarised using statistics for continuous variables.

Statistics will be based on the double-blind safety population, and no imputations of missing values will be made. In particular, a missing item will lead to a missing overall score. Statistics by actual treatment group and overall will be produced.

DocuSign Envelope ID: 9580C97D-56AD-4E50-9133-FAC5390A9286

10.4.11 Cognitive functioning: Verbal Fluency, SDMT and MoCA scores

Verbal fluency is measured as “category” and “letter”, and one score for each will be recorded. The SDMT measurement will be provided as one score.

The Montreal Cognitive Assessment (MoCA) assesses different cognitive domains. The result of the test will be reported as Memory Index Score and Total score.

For the baseline (Day 1) and for Days 8, 15, 22, 29, 36, 64, 92, 120 and “early termination” the above mentioned scores will be summarised using statistics for continuous variables. Moreover, the change of the values from Day 8 and beyond from the baseline value (Day 1) by subject will be summarised using statistics for continuous variables.

Statistics will be based on the double-blind safety population, and no imputations of missing values will be made. Statistics by actual treatment group will be produced.

DocuSign Envelope ID: 9580C97D-56AD-4E50-9133-FAC5390A9286

Section 11: List of tables, listings, and figures

Type	Number	Title
Table	14.1.1	Subject disposition – all screened subjects
Table	14.1.2	Demographics – full analysis population
Table	14.1.3	Medical History – full analysis population
Table	14.1.4	Substance use at baseline – full analysis population
Table	14.2.1.1	Primary endpoint, sensitivity analysis: MADRS score (Day 92) with ANCOVA – full analysis population
Table	14.2.1.2	Primary endpoint, sensitivity analysis: MADRS score (Day 92) with ANCOVA – per protocol population
Table	14.2.1.3	Primary/secondary endpoints: MADRS score (Day 92, Day 64, and Day 36) – full analysis population
Table	14.2.1.4	Primary/secondary endpoints, sensitivity analysis: MADRS score (Day 92, Day 64, and Day 32) with multiple imputations – full analysis population
Table	14.2.2.1	Secondary endpoint: Count of relapse – full analysis population
Table	14.2.2.2	Secondary endpoint: Time to relapse – full analysis population
Table	14.2.2.3	Secondary endpoint: Count of response – full analysis population
Table	14.2.2.4	Secondary endpoint: Count of remission – full analysis population
Table	14.2.2.5.1	Secondary endpoint: CGI-S, observed – full analysis population
Table	14.2.2.5.2	Secondary endpoint: CGI-S, odds ratios, Day 92 – full analysis population
Table	14.2.2.6.1	Secondary endpoint: PGI-I, observed – full analysis population
Table	14.2.2.6.2	Secondary endpoint: PGI-I, estimated probabilities, Day 92 – full analysis population
Table	14.2.2.7.1	Exploratory endpoint: PGI-S, observed – full analysis population
Table	14.2.2.7.2	Exploratory endpoint: PGI-S, estimated probabilities, Day 92 – full analysis population
Table	14.2.2.8.1	Exploratory endpoint: EQ-5D-5L dimensions – full analysis population
Table	14.2.2.8.2	Exploratory endpoint: EQ-5D-5L, overall health compared to baseline – full analysis population
Table	14.2.2.9	Exploratory endpoint: EQ VAS – full analysis population
Table	14.2.2.10	Exploratory endpoint: WSAS – full analysis population
Table	14.3.1.1	Safety endpoint: Treatment emergent adverse events, enrichment open-label phase – open-label safety population
Table	14.3.1.2	Safety endpoint: Treatment emergent adverse events, randomised double-blind phase – double-blind safety population
Table	14.3.1.3	Safety endpoint: Treatment emergent adverse events by system organ class and preferred term, randomized double-blind phase - open-label safety population
Table	14.3.1.4	Safety endpoint: Treatment emergent adverse events by system organ class and preferred term, randomized double-blind phase - double-blind safety population
Table	14.3.1.5	Safety endpoint: Treatment emergent adverse events by maximum severity, randomized double-blind phase - open-label safety population
Table	14.3.1.6	Safety endpoint: Treatment emergent adverse events by maximum severity, randomized double-blind phase - double-blind safety population
Table	14.3.2.1	Safety endpoint: Treatment emergent serious adverse events by system organ class and preferred term, randomized double-blind phase - open-label safety population

DocuSign Envelope ID: 9580C97D-56AD-4E50-9133-FAC5390A9286

Type	Number	Title
Table	14.3.2.2	Safety endpoint: Treatment emergent serious adverse events by system organ class and preferred term, randomized double-blind phase - double-blind safety population
Table	14.3.2.3	Safety endpoint: Treatment emergent adverse events leading to death by system organ class and preferred term, randomized double-blind phase - open-label safety population
Table	14.3.2.4	Safety endpoint: Treatment emergent adverse events leading to death by system organ class and preferred term, randomized double-blind phase - double-blind safety population
Table	14.3.3.1	Safety endpoint: eC-SSRS, Number of patients with suicidal ideation, suicidal behaviour, and self-injurious behaviour without suicidal intent – double-blind safety population
Table	14.3.3.2	Safety endpoint: eC-SSRS, Number of patients with suicide-related treatment-emergent events – double-blind safety population
Table	14.3.4.1	Safety endpoint: CADSS total score, randomised double-blind phase – double-blind safety population
Table	14.3.4.2	Safety endpoint: CADSS Amnesia score, randomised double-blind phase – double-blind safety population
Table	14.3.4.3	Safety endpoint: CADSS Depersonalisation score, randomised double-blind phase – double-blind safety population
Table	14.3.4.4	Safety endpoint: CADSS Derealisation score, randomised double-blind phase – double-blind safety population
Table	14.3.5.1	Safety endpoint: BPRS (5 items) total score, randomised double-blind phase - double-blind safety population
Table	14.3.5.2	Safety endpoint: BPRS Conceptual disorganization score, randomised double-blind phase - double-blind safety population
Table	14.3.5.3	Safety endpoint: BPRS Suspiciousness score, randomised double-blind phase - double-blind safety population
Table	14.3.5.4	Safety endpoint: BPRS Hallucinatory behaviour score, randomised double-blind phase - double-blind safety population
Table	14.3.5.5	Safety endpoint: BPRS Unusual thought score, randomised double-blind phase - double-blind safety population
Table	14.3.5.6	Safety endpoint: BPRS Grandiosity score, randomised double-blind phase - double-blind safety population
Table	14.3.6.1	Safety endpoint: BPIC-SS total score, randomised double-blind phase – double-blind safety population
Table	14.3.7.1	Safety endpoint: Verbal fluency - category, randomised double-blind phase – double-blind safety population
Table	14.3.7.2	Safety endpoint: Verbal fluency - letter, randomised double-blind phase – double-blind safety population
Table	14.3.8.1	Safety endpoint: SDMT, randomised double-blind phase – double-blind safety population
Table	14.3.9.1	Safety endpoint: MoCA – memory index, randomised double-blind phase – double-blind safety population
Table	14.3.9.2	Safety endpoint: MoCA – total score, randomised double-blind phase – double-blind safety population
Table	14.3.10.1.x	Safety endpoint: Vital signs - <PARAMETER> - enrichment open-label phase - open-label safety population
Table	14.3.10.2.x	Safety endpoint: Vital signs - <PARAMETER> - randomised double-blind phase – double-blind safety population

Type	Number	Title
Table	14.3.11.1.x	Safety endpoint: Urinalysis - <PARAMETER> - enrichment open-label phase - open-label safety population
Table	14.3.11.2.x	Safety endpoint: Urinalysis - <PARAMETER> - randomised double-blind phase – double-blind safety population
Table	14.3.11.3.x	Safety endpoint: Urinalysis - <PARAMETER> - clinical significance - enrichment open-label phase - open-label safety population
Table	14.3.11.4.x	Safety endpoint: Urinalysis - <PARAMETER> - clinical significance - randomised double-blind phase – double-blind safety population
Table	14.3.11.5	Safety endpoint: Urinalysis, clinical significance summary – open-label safety population
Table	14.3.12.1.x	Safety endpoint: Serum chemistry - <PARAMETER> - enrichment open-label phase - open-label safety population
Table	14.3.12.2.x	Safety endpoint: Serum chemistry - <PARAMETER> - randomised double-blind phase – double-blind safety population
Table	14.3.12.3.x	Safety endpoint: Serum chemistry - <PARAMETER> - clinical significance - enrichment open-label phase - open-label safety population
Table	14.3.12.4.x	Safety endpoint: Serum chemistry - <PARAMETER> - clinical significance - randomised double-blind phase – double-blind safety population
Table	14.3.12.5	Safety endpoint: Serum chemistry, clinical significance summary - open-label safety population
Table	14.3.13.1.x	Safety endpoint: Haematology - <PARAMETER> - enrichment open-label phase - open-label safety population
Table	14.3.13.2.x	Safety endpoint: Haematology - <PARAMETER> - randomised double-blind phase – double-blind safety population
Table	14.3.13.3.x	Safety endpoint: Haematology - <PARAMETER> - clinical significance - enrichment open-label phase - open-label safety population
Table	14.3.13.4.x	Safety endpoint: Haematology - <PARAMETER> - clinical significance - randomised double-blind phase – double-blind safety population
Table	14.3.13.5	Safety endpoint: Haematology, clinical significance summary - open-label safety population
Table	14.3.14.1	Safety endpoint: BMI, enrichment open-label phase – open-label safety population
Table	14.3.14.2	Safety endpoint: BMI, randomised double-blind phase – double-blind safety population
Table	14.3.15.1	Safety endpoint: Physical examination, baseline and randomised double-blind phase – double-blind safety population
Table	14.3.16.1	Safety endpoint: ECG, randomised double-blind phase – double-blind safety population
Table	14.3.17	Compliance – full analysis population
Table	DSMC.1	Primary/secondary endpoints, sensitivity analysis: MADRS score (Day 92, Day 64, and Day 36) relative to Day 8 – full analysis population
Table	DSMC.2	Summary at Day 8: MoCA, SDMT, verbal fluency, WSAS – FA population
Table	DSMC.3	Summary at Day 8: CGI-S, PGI-S – FA population
Listing	16.2.1.1	Subject disposition, part 1 – enrolled
Listing	16.2.1.2	Subject disposition, part 2 – enrolled
Listing	16.2.2	Protocol Deviations – enrolled
Listing	16.2.3.1	PP population selection – full analysis population
Listing	16.2.3.2	Analysis populations – all screened

Type	Number	Title
Listing	16.2.4.1	Demographics – enrolled
Listing	16.2.4.2	Medical history – enrolled
Listing	16.2.4.3	Physical examination – enrolled
Listing	16.2.4.4	Substance use – enrolled
Listing	16.2.4.5	Medication – enrolled
Listing	16.2.4.6	Serology – enrolled
Listing	16.2.5.1	Dosing, enrichment open-label phase – open-label safety
Listing	16.2.5.2	Drug administration and accountability, randomised double-blind phase – double-blind safety
Listing	16.2.5.3	Compliance, randomised double-blind phase – full analysis population
Listing	16.2.6.1	MADRS scores – enrolled
Listing	16.2.6.2	Response and remission – full analysis population
Listing	16.2.6.3	CGI-S – enrolled
Listing	16.2.6.4	PGI-I – full analysis population
Listing	16.2.6.5	PGI-S – enrolled
Listing	16.2.6.6	EQ-5D-5L – enrolled
Listing	16.2.6.7	WSAS – enrolled
Listing	16.2.7.1	Adverse events – open-label safety population without double-blind safety population
Listing	16.2.7.2	Adverse events – double-blind safety population
Listing	16.2.7.3	Serious adverse events – open-label safety population without double-blind safety population
Listing	16.2.7.4	Serious adverse events – double-blind safety population
Listing	16.2.7.5	Adverse events leading to death – open-label safety population without double-blind safety population
Listing	16.2.7.6	Adverse events leading to death – double-blind safety population
Listing	16.2.8.1	Vital signs – enrolled
Listing	16.2.8.2	Urinalysis part 1 – enrolled
Listing	16.2.8.3	Urinalysis part 2 – enrolled
Listing	16.2.8.4	Serum chemistry, part 1, values – enrolled
Listing	16.2.8.5	Serum chemistry, part 1, clinical significance – enrolled
Listing	16.2.8.6	Serum chemistry, part 2, values – enrolled
Listing	16.2.8.7	Serum chemistry, part 2, clinical significance – enrolled
Listing	16.2.8.8	Haematology, values – enrolled
Listing	16.2.8.9	Haematology, clinical significance – enrolled
Listing	16.2.8.10	Weight, BMI – enrolled
Listing	16.2.8.11	ECG – enrolled
Listing	16.2.8.12	eC-SSRS – enrolled
Listing	16.2.8.13	eC-SSRS, key for abbreviations
Listing	16.2.8.14	CADSS – enrolled
Listing	16.2.8.15	BPRS (5 items) – enrolled
Listing	16.2.8.16	BPIC-SS – enrolled
Listing	16.2.8.17	Verbal fluency, SDMT and MoCA – enrolled
Listing	16.2.8.18	Pregnancy, FSH – enrolled
Listing	16.2.8.19	MINI, MSM – all screened

Type	Number	Title
Figure	14.2.1.1	Time-response pattern, MADRS score, estimated marginal means – full analysis population
Figure	14.2.1.2	Dose-response pattern, MADRS score, estimated marginal means – full analysis population
Figure	14.2.1.3	Time-response pattern, MADRS score, observed means – full analysis population
Figure	14.2.1.4	Dose-response pattern, MADRS score, observed means – full analysis population
Figure	14.2.2	MADRS score by time in trial – full analysis population
Figure	14.2.3	Time to relapse – full analysis population
Figure	14.2.4.1	EQ-VAS scores, time-response – full analysis population
Figure	14.2.4.2	EQ-VAS scores, dose-response – full analysis population
Figure	14.3.1.1	Vital signs: Systolic blood pressure – double-blind safety population
Figure	14.3.1.2	Vital signs: Diastolic blood pressure - double-blind safety population
Figure	14.3.1.3	Vital signs: Pulse rate - double-blind safety population
Figure	14.3.1.4	Vital signs: Temperature - double-blind safety population
Figure	14.3.1.5	Vital signs: Respiratory rate - double-blind safety population
Figure	14.3.1.6	Vital signs: O2 saturation - double-blind safety population
Figure	14.3.1.7	Vital signs: Day 92, change from baseline - double-blind safety population
Figure	14.3.2.1	Haematology: Haemoglobin - double-blind safety population
Figure	14.3.2.2	Haematology: Haematocrit - double-blind safety population
Figure	14.3.2.3	Haematology: Absolute neutrophil count - double-blind safety population
Figure	14.3.2.4	Haematology: Lymphocyte count - double-blind safety population
Figure	14.3.2.5	Haematology: Monocyte count - double-blind safety population
Figure	14.3.2.6	Haematology: Eosinophil count - double-blind safety population
Figure	14.3.2.7	Haematology: Total basophil count - double-blind safety population
Figure	14.3.2.8	Haematology: Platelet count - double-blind safety population
Figure	14.3.3.1	Serum Chemistry: Sodium - double-blind safety population
Figure	14.3.3.2	Serum Chemistry: Potassium - double-blind safety population
Figure	14.3.3.3	Serum Chemistry: Chloride - double-blind safety population
Figure	14.3.3.4	Serum Chemistry: Glucose - double-blind safety population
Figure	14.3.3.5	Serum Chemistry: Uric acid - double-blind safety population
Figure	14.3.3.6	Serum Chemistry: Total protein - double-blind safety population
Figure	14.3.3.7	Serum Chemistry: Magnesium - double-blind safety population
Figure	14.3.3.8	Serum Chemistry: BUN or urea - double-blind safety population
Figure	14.3.3.9	Serum Chemistry: Creatinine - double-blind safety population
Figure	14.3.3.10	Serum Chemistry: Albumin - double-blind safety population
Figure	14.3.3.11	Serum Chemistry: Aspartate aminotransferase - double-blind safety population
Figure	14.3.3.12	Serum Chemistry: Alanine aminotransferase - double-blind safety population
Figure	14.3.3.13	Serum Chemistry: Alkaline phosphatase - double-blind safety population
Figure	14.3.3.14	Serum Chemistry: Total bilirubin - double-blind safety population
Figure	14.3.3.15	Serum Chemistry: Lactate dehydrogenase - double-blind safety population
Figure	14.3.3.16	Serum Chemistry: Ammonia - double-blind safety population
Figure	14.3.3.17	Serum Chemistry: Calcium - double-blind safety population
Figure	14.3.3.18	Serum Chemistry: Phosphorus - double-blind safety population
Figure	14.3.3.19	Serum Chemistry: Amylase - double-blind safety population
Figure	14.3.3.20	Serum Chemistry: Lipase - double-blind safety population

DocuSign Envelope ID: 9580C97D-56AD-4E50-9133-FAC5390A9286

Type	Number	Title
Figure	14.3.4	BMI - double-blind safety population
Figure	14.3.5.1	BPIC-SS: Total score, time-response - double-blind safety population
Figure	14.3.5.2	BPIC-SS: Total score, dose-response - double-blind safety population
Figure	14.3.5.3	BPIC-SS: Urination due to bladder pain - double-blind safety population
Figure	14.3.5.4	BPIC-SS: Need to urinate just after urination - double-blind safety population
Figure	14.3.5.5	BPIC-SS: Urination in order to avoid bladder pain - double-blind safety population
Figure	14.3.5.6	BPIC-SS: Bladder pressure - double-blind safety population
Figure	14.3.5.7	BPIC-SS: Bladder pain - double-blind safety population
Figure	14.3.5.8	BPIC-SS: Feeling bothered due to frequent urination during daytime - double-blind safety population
Figure	14.3.5.9	BPIC-SS: Feeling bothered due to urination during night - double-blind safety population
Figure	14.3.5.10	BPIC-SS: Worst bladder pain - double-blind safety population
Figure	14.3.6.1	Verbal fluency, category - double-blind safety population
Figure	14.3.6.2	Verbal fluency, letter - double-blind safety population
Figure	14.3.7	SDMT - double-blind safety population
Figure	14.3.8.1	WSAS, ability to work – full analysis population
Figure	14.3.8.2	WSAS, home management – full analysis population
Figure	14.3.8.3	WSAS, social leisure activities – full analysis population
Figure	14.3.8.4	WSAS, private leisure activities – full analysis population
Figure	14.3.8.5	WSAS, close relationships – full analysis population
Figure	14.3.9	Time to onset of TEAE – double-blind safety population

DocuSign Envelope ID: 9580C97D-56AD-4E50-9133-FAC5390A9286

References

1. ICH (1998). ICH Topic E9 Statistical Principles for Clinical Trials
2. ICH (2018). ICH E9 (R1) Addendum on Estimands and Sensitivity Analysis in Clinical Trials to the guideline on Statistical Principles for Clinical Trials EMA/CHMP/ICH/436221/2017
3. Douglas Pharmaceuticals Ltd. BEDROC. 26 February 2019. *A Phase 2a Proof-of-Concept Study of R-107 for the Treatment of Refractory Major Depressive Disorder.* Version 6.0 [v5.4 Singapore / Taiwan]
4. R Core Team (2013). R: *A language and environment for statistical computing*. R Foundation for Statistical Computing, Vienna, Austria.
5. SAS Institute Inc., Cary, NC (2013): SAS version 9.4

Site PIs, Bedroc Study

The Black Dog Institute, Sydney, Australia

Colleen Loo

Brain and Mind Centre, University of Sydney, Australia

Nick Glozier

Australian National University College of Health and Medicine, Canberra, Australia

Paul Fitzgerald

Lyell McEwin Hospital, Adelaide, Australia

Dennis Liu

Gold Coast University Hospital, Queensland, Australia

Shanthi Sarma

Peninsula Therapeutic & Research Group, Melbourne, Australia

Jennifer Grunfeld

Neurocentrix, Melbourne, Australia

David Barton

Ramsay Clinic Albert Road, University of Melbourne, Australia

Malcolm Hopwood

University of Otago, Dunedin, New Zealand

Paul Glue

North Shore Hospital, Auckland, New Zealand

Wayne Miles

Lakeland Clinical Trials & Anteris Clinical Research, New Zealand

Mike Williams

Southern Clinical Trials, Christchurch, New Zealand

Simon Carson

National University Hospital Singapore, Singapore

Johnson Fam

Institute of Mental Health, Singapore

Phern-Chern Tor

China Medical University Hospital, Taiwan

Hsien-Yuan Lane

Taipei Municipal Wanfang Hospital, Taiwan

Chun-Hsin Chen

Taipei Medical University Hospital, Taiwan

Yu-Jui Huang
