# Supplementary material for: Extended-release ketamine tablets for treatment-resistant depression: a randomized placebo-controlled phase 2 trial
Source: Nat Med. 2024 Jun 24;30(7):2004–9. doi: 10.1038/s41591-024-03063-x (PMC11271411; doi:10.1038/s41591-024-03063-x)
Supplement: Supplementary file 2 — Reporting Summary [file 41591_2024_3063_MOESM2_ESM.pdf]

Corresponding author(s): Paul Glue MD

Last updated by author(s): 12th April 2024

Reporting Summary

Nature Portfolio wishes to improve the reproducibility of the work that we publish. This form provides structure for consistency and transparency in reporting. For further information on Nature Portfolio policies, see our Editorial Policies and the Editorial Policy Checklist.

Statistics

For all statistical analyses, confirm that the following items are present in the figure legend, table legend, main text, or Methods section.

n/a	Confirmed	
☐	☒	The exact sample size (*n*) for each experimental group/condition, given as a discrete number and unit of measurement
☐	☒	A statement on whether measurements were taken from distinct samples or whether the same sample was measured repeatedly
☐	☒	The statistical test(s) used AND whether they are one- or two-sided<br>*Only common tests should be described solely by name; describe more complex techniques in the Methods section.*
☐	☒	A description of all covariates tested
☐	☒	A description of any assumptions or corrections, such as tests of normality and adjustment for multiple comparisons
☐	☒	A full description of the statistical parameters including central tendency (e.g. means) or other basic estimates (e.g. regression coefficient) AND variation (e.g. standard deviation) or associated estimates of uncertainty (e.g. confidence intervals)
☐	☒	For null hypothesis testing, the test statistic (e.g. *F*, *t*, *r*) with confidence intervals, effect sizes, degrees of freedom and *P* value noted<br>*Give P values as exact values whenever suitable.*
☒	☐	For Bayesian analysis, information on the choice of priors and Markov chain Monte Carlo settings
☐	☒	For hierarchical and complex designs, identification of the appropriate level for tests and full reporting of outcomes
☒	☐	Estimates of effect sizes (e.g. Cohen's *d*, Pearson's *r*), indicating how they were calculated

Our web collection on statistics for biologists contains articles on many of the points above.

Software and code

Policy information about availability of computer code

Data collection	Electronic CRF
Data analysis	SAS version 9.4

For manuscripts utilizing custom algorithms or software that are central to the research but not yet described in published literature, software must be made available to editors and reviewers. We strongly encourage code deposition in a community repository (e.g. GitHub). See the Nature Portfolio guidelines for submitting code & software for further information.

Data

Policy information about availability of data

All manuscripts must include a data availability statement. This statement should provide the following information, where applicable:

- Accession codes, unique identifiers, or web links for publicly available datasets
- A description of any restrictions on data availability
- For clinical datasets or third party data, please ensure that the statement adheres to our policy

Data will not be available until all clinical registration studies are complete

Human research participants

Policy information about studies involving human research participants and Sex and Gender in Research.

Reporting on sex and gender	91 males and 76 females were randomized
Population characteristics	Randomized participants has a diagnosis if treatment resistant depression, with MADRS scores of >/= 20 at baseline, and were responders to 5 days of R-017 dosing 120mg.day.
Recruitment	Participants were recruited at 20 outpatient psychiatric clinics in New Zealand, Australia, Taiwan and Singapore.
Ethics oversight	Ethics review was conducted by national or local ethics committees.

Note that full information on the approval of the study protocol must also be provided in the manuscript.

Field-specific reporting

Please select the one below that is the best fit for your research. If you are not sure, read the appropriate sections before making your selection.

☐ Life sciences ☒ Behavioural & social sciences ☐ Ecological, evolutionary & environmental sciences

For a reference copy of the document with all sections, see nature.com/documents/nr-reporting-summary-flat.pdf

Behavioural & social sciences study design

All studies must disclose on these points even when the disclosure is negative.

Study description	After a 5 day open-label enrichment phase, treatment responders were randomized to twice weekly doses of R-107 0, 30, 60, 120 or 180mg for 12 weeks. Quantitative data were collected..
Research sample	Adult patients with treatment-resistant DSM-5 Major Depressive DIsorder, who were responders to a 5 day open-label enrichment phase (R-107 120mg/day for 5 days). Treatment-resistant depression was selected as ketamine's activity in this population is well established.
Sampling strategy	Treatment responders to the enrichment phase were randomized 1:1:1:1:1 to double blind R-107 0, 30, 60, 120 or 180mg twice weekly. Sample size was based on statistical power assumptions, of a 6 MADRS point difference between R-107 and placebo, SD 7,5 points, 80% power and alpha of 0.05..
Data collection	Data were collected on an eCRF at clinic visits, which were intitally daily, and later at weekly intervals, The primary endpoint was the MADRS; secondary efficacy endpoints were CGI and PGI. Dissociation was monitored using the CADSS scale. Adverse events, safety laboratory tests and ECGs were also collected,The researchers were blind to treatment allocation.
Timing	Data were recorded continuously between May 2019 and August 2021.
Data exclusions	No data were excluded
Non-participation	Following randomization on Day 8, no participants were excluded
Randomization	Responders to the 5 day enrichment phase were randomized 1:1:1:1:1 to study treatments using an automated integrated web response system.

Reporting for specific materials, systems and methods

We require information from authors about some types of materials, experimental systems and methods used in many studies. Here, indicate whether each material, system or method listed is relevant to your study. If you are not sure if a list item applies to your research, read the appropriate section before selecting a response.

Materials & experimental systems

n/a	Involved in the study
☒	☐ Antibodies
☒	☐ Eukaryotic cell lines
☒	☐ Palaeontology and archaeology
☒	☐ Animals and other organisms
☐	☒ Clinical data
☒	☐ Dual use research of concern

Methods

n/a	Involved in the study
☒	☐ ChIP-seq
☒	☐ Flow cytometry
☒	☐ MRI-based neuroimaging

Clinical data

Policy information about clinical studies

All manuscripts should comply with the ICMJE guidelines for publication of clinical research and a completed CONSORT checklist must be included with all submissions.

Clinical trial registration: AN

Study protocol: Included in supplementary data

Data collection: Data were collected at daily-weekly visits to the study clinic and included mood rating data, safety laboratory tests, adverse events, ECGs, and measures of dissociation

Outcomes: Efficacy Primary: MADRS; secondary: CGI, PGI. Safety: adverse events, vital signs, ECGs, safety laboratory tests, ECGs, ratings of dissociation
